# Genotype-informed docosahexaenoic acid dosing based on the FADS Indel to reduce preterm and prolonged gestation: A secondary analysis of the ADORE randomized trial

**DOI:** 10.1371/journal.pmed.1005274

**Published:** 2026-10-08

**Authors:** Yan Ning Li, Kumar S. D. Kothapalli, Susan E. Carlson, Byron J. Gajewski, Emily A. DeFranco, J. Thomas Brenna

**Affiliations:** 1 Dell Pediatric Research Institute, Dell Medical School and Department of Nutritional Sciences, The University of Texas at Austin, Austin, Texas, United States of America; 2 University of Kansas Medical Center, Kansas City, Kansas, United States of America; 3 University of Kentucky, Lexington, Kentucky, United States of America; University Hospital Ghent: Universitair Ziekenhuis Gent, BELGIUM

## Abstract

**Background:**

Spontaneous early preterm birth (EPB < 34 weeks) is a major contributor to newborn morbidity and mortality; it depends on ancestry, and preventive measures are limited. The insertion (I) allele of the fatty acid desaturase (FADS) Indel polymorphism (rs66698963) modulates FADS1 expression, and increases arachidonic acid synthesis, enhancing parturifacient prostaglandins. Omega-3 docosahexaenoic acid (DHA) inhibits this pathway. Whether FADS Indel genotype predicts response to DHA dose in pregnancy is unknown.

**Methods and findings:**

To determine whether FADS Indel genotype modifies the effect of DHA dose on gestational length, we conducted a post hoc secondary analysis of the Assessment of DHA on Reducing Early Preterm Birth (ADORE) trial (NCT02626299). The FADS Indel, the only genetic variant measured, was selected on mechanistic grounds and genotyped blind to outcome. The analysis included all participants consenting to genetic analysis (12–20 weeks gestation) at three US medical centers. Participants received 200 or 1,000 mg/d DHA and were genotyped for FADS Indel (D/D, I/D, I/I). Primary outcomes were EPB and preterm birth (PB, <37 weeks) analyzed using treatment-specific Bayesian binomial models. Cervical length was assessed in 574 participants.

Women (*n* = 10,497) were screened for eligibility from June 8th 2016, to March 13th 2020, 1,032 (1,000 mg, *n* = 540; 200 mg, *n* = 492) completed the ADORE trial. A total of 761 (1,000 mg, *n* = 396; 200 mg, *n* = 365) are included in this secondary analysis. Participants carrying at least one FADS Indel I allele (I/I + I/D) receiving low-dose DHA had 4.3% EPB (9/214; 95% Credible Interval (CrI) [2.0%, 7.3%]) versus 0.8% in the D/D group (1/151; 95% CrI [0.04%, 2.7%]; Posterior Probability (PP) = 0.99), and 14.5% PB (31/214; 95% CrI [10.1%, 19.5%]) versus 8.7% in the D/D group (13/151; 95% CrI [4.7%, 13.6%]; PP = 0.96). High-dose DHA was associated with reduced EPB in the I-allele group (1.1%; 2/205; 95% CrI [0.2%, 2.8%]; PP = 0.99), and PB (9.3%; 19/205; 95% CrI [5.7%, 13.6%]; PP = 0.95), with little effect in the D/D group. Birth at ≥40 weeks was more frequent in the high-dose D/D group. Black women had higher rates of PB and EPB, have higher rates of the I allele, and benefited most from 1,000 mg/d dose. These dose effects credibly differ by genotype for EPB and prolonged gestation (genotype × dose interaction PP = 0.99 and 0.98). Both genotypes reduced PB similarly with high-dose DHA (PP = 0.56). Although genetic analysis was anticipated and genotyping was completed blind to outcome, this was a post hoc analysis and the genotype-by-dose comparison was not a prespecified trial aim. Some subgroups contained few PB and EPB events, so these estimates, particularly for PB, should be interpreted cautiously and confirmed prospectively.

**Conclusions:**

The FADS Indel is a functional, mechanistically grounded polymorphism whose frequency varies with race/ethnicity. Preterm and early preterm birth were lower in the I-allele group receiving high-dose DHA (1,000 mg/d), while prolonged gestation was more frequent in the D/D group receiving high-dose DHA. FADS Indel genotypes predicted the direction of the response to high-dose DHA for EPB and prolonged gestation, whereas its modest PB benefit was shared by both genotypes. Analysis by retrospectively pairing high dose to I-carrier genotype and low dose to D/D was associated with the highest proportion of births at 37–40 weeks. These findings identify the FADS Indel as a potential predictive biomarker for DHA dose response and support a genotype-informed, precision-medicine strategy to optimize gestational length by reducing preterm birth in the I-allele group and prolonged gestation in the D/D group across racially/ethnically diverse populations.

**Trial registration:**

Clinical trial identification number: NCT02626299, Date of registration: December 8, 2015, Date of Initial participant enrolled: June 8, 2016 https://clinicaltrials.gov/study/NCT02626299.

## Author summary

**Why was this study done?:**

- Globally, complications of preterm birth are the leading cause of death among children under five and a major cause of long-term disability, with disproportionate burden on Black women, and DHA supplementation has been broadly associated with lower PB and EPB rates despite known heterogeneity in response.
- The FADS Indel mechanistically raises arachidonic acid and its downstream prostaglandin PGE2, a driver of parturition that DHA antagonizes, and its frequency itself tracks ancestry.
- To our knowledge, no study exists on whether this genotype associates who benefits from higher-dose DHA.

**What did the researchers do and find?:**

- We performed a prospective-retrospective secondary/post hoc analysis of the ADORE randomized trial; 761 of 1,032 participants at three US sites were genotyped for FADS Indel and analyzed by DHA dose (200 versus 1,000 mg/d) using Bayesian binomial models.
- High-dose DHA was associated with lower early preterm birth rates in the I-allele group: 1.1% with high-dose DHA (95% CrI [0.2%, 2.8%]) versus 4.3% with low-dose DHA (95% CrI [2.0%, 7.3%]; PP = 0.99), whereas prolonged gestation was more frequent in the high-dose D/D group.
- Women of African ancestry, who carry the I allele more often, benefited most.

**What do these findings mean?:**

- The FADS Indel emerges as a candidate predictive biomarker for DHA dose–response in pregnancy, supporting genotype-informed dosing over flat recommendations.
- In this post hoc analysis, matching DHA dose to genotype was associated with the highest observed proportion of births between 37 and <40 weeks. Because this analysis was conducted after the trial and included few early preterm births in some groups, the findings should be confirmed in future studies designed specifically to evaluate genotype-guided DHA dosing.

## Abstract 3

In a secondary analysis of a trial of docosahexaenoic acid (DHA) in pregnant women, Yan Ning Li and colleagues examine whether different genotypes of the FADS indel might influence the effects of a low or high DHA dose on the timing of birth.

## Introduction

Preterm birth (PB), defined as birth before 37 weeks of gestational age, is the leading cause of mortality and morbidity in children under 5 years of age [1]. Early PB (EPB) that occurs before 34 weeks’ gestation is a particular concern for long-term morbidity, especially lasting neurological deficits [2,3]. The annual preterm birth rate in the US is near an all-time high at 10.4%, and EPB rate is at 2.16% [4]. Some races and ethnicities have higher rates of EPB and PB, including Black persons [5], and populations of South Asia, particularly India [6].

Vaginal progesterone and cervical cerclage are two widely used interventions for preventing PB and EPB [7]. Vaginal progesterone lowers PB rates in women with a mid-trimester short cervix (≤25 mm) [8], but is ineffective for the prevention of PB in those with normal cervical length regardless of prior history [9,10]. Accordingly, the current American College of Obstetricians and Gynecologists (ACOG) guidelines do not recommend vaginal progesterone use in the absence of cervical shortening, regardless of prior PB history [11]. Cervical cerclage, or cervical stitch, is a prophylactic surgical reinforcement of the cervix. ACOG supports history-indicated cerclage placement at around 12–14 weeks of gestation for women with a history suggestive of cervical insufficiency or ultrasound-indicated cerclage in the second trimester in women with prior spontaneous PB and cervical shortening before 24 weeks of gestation in the current pregnancy [12].

Supplemental intake of the omega-3 fatty acids, docosahexaenoic acid (DHA) and eicosapentaenoic acid (EPA), is the only broadly effective intervention known to reduce PB and EPB in an unselected population [13]. The mechanism of action is likely to be based on competition with arachidonic acid (ARA) signaling for inflammation or direct myometrial activity via the parturifacient prostaglandin E2 (PGE2), used clinically to induce labor. Early clinical [14] and animal studies [15] demonstrate that DHA and EPA inhibit PGE2 formation [16]. EPA and DHA may also support the production of specialized pro-resolving lipid mediators [17], though their role in parturition is uncertain. Importantly, some evidence of prolonged gestation with DHA supplementation has figured into recommendations [18].

Circulating ARA increases in response to intake of its omega-6 fatty acid precursor, linoleic acid, up to about 4% of total energy intake. In populations with industrialized food supplies, such as in the US, where linoleic acid exceeds 4% of energy, circulating ARA levels plateau. In those populations, the FADS Indel, a specific insertion-deletion (Indel) polymorphism (rs66698963) in the FADS gene cluster at 11q12-13, strongly modulates circulating ARA [19,20]. The FADS Indel genotype determines endogenous arachidonic acid (ARA) biosynthetic capacity: individuals homozygous for the deletion allele (D/D) have the lowest circulating ARA, heterozygotes (I/D) are intermediate, and those homozygous for the insertion allele (I/I) have the highest, reflecting an allele-dose effect on FADS1 expression and enzymatic conversion of the ARA precursor di-homo-gamma-linolenic acid (DGLA) to ARA. The deletion (D) allele dominates in persons of European and Chinese ancestry; however, we showed a higher prevalence of the insertion (I) allele in Africans, South Asian Indians, and US Black persons [19]. The I allele causes higher expression of FADS1, the last step in human endogenous ARA biosynthesis, resulting in higher circulating ARA and lower circulating di-homo-gamma-linolenic acid (DGLA), the immediate precursor of ARA [21]. As a result, prostaglandin levels driven by PGE2 synthesis will be higher among participants carrying at least one I allele, putatively leading to higher risks of PB and EPB.

A recently reported randomized clinical trial of DHA supplementation in pregnancy (Assessment of DHA on Reducing Early Preterm Birth or ADORE) [22] found a high probability that high-dose DHA (1,000 mg/d) reduced early preterm birth (EPB) less than 34 weeks compared to low-dose DHA (200 mg/d), particularly in participants who began the trial with low red blood cell phospholipid (RBC-PL) DHA. We used a prospective-retrospective framework in this post hoc analysis to analyze EPB and PB less than 37 weeks by DHA dose (200 or 1,000 mg/d), Indel genotypes (I/I, ID, D/D), and race (Black or Non-Black) [23,24]. Race was included in our analysis because Black persons have a higher incidence of the I allele [19]. Additionally, a planned secondary analysis of the ADORE trial found that Black participants who began the trial with higher baseline RBC-PL DHA and who received high-dose DHA had rates of EPB and PB of 2.1% and 6.4%, respectively [25], below recent US averages of 4.0% and 12.3% for Black singleton pregnancy [26]. We hypothesized that, among participants carrying the FADS Indel I allele, 1,000 mg/d DHA would be associated with lower rates of PB and EPB than 200 mg/d DHA; we also examined whether the frequency of birth at ≥40 weeks differed by genotype and dose.

## Methods

### Study design and participants

The ADORE trial was a multicenter, double-blind, randomized, superiority trial aimed to evaluate the effectiveness of high-dose (1,000 mg) DHA in reducing the rates of PB and EPB compared to a low dose (200 mg) per day, which is a standard dose in the US recommended for support of infant neural development [27]. DHA capsules were orange-flavored and dispensed in opaque bottles coded only as “black stripe” or “solid black,” to conceal dose assignment. Participants and study-team members were masked to assignment throughout the trial, except two team members who conducted the interim analyses and updated the allocation tables, and who had no contact with participants. Managing study personnel remained blinded until 30 days after the last birth [22]. The University of Kansas Medical Center granted approval under a central IRB with reliance by the other institutions (STUDY00003455) [22,27]. Clinical trial identification number: NCT02626299, Date of registration: December 8, 2015, Date of Initial participant enrolled: June 8, 2016 https://clinicaltrials.gov/study/NCT02626299

Group sizes were determined by power calculations available in the statistical analysis plan. Eleven hundred participants were recruited in three academic medical centers in Ohio and Kansas. Participants were enrolled after 12- and before 20-weeks’ gestation between June 2016 and March 2020. Women were excluded if they had multiple gestations, were <18 years old, less than 12 weeks of gestational age, or greater than 20 weeks of gestational age. Participants were randomly assigned by an electronic research tool to ingest either 200 mg/d or 1,000 mg/d DHA. An adaptive design assigned more participants to the winning dose [22]. Race and ethnicity were self-reported by participants of the ADORE trial. Options provided were: Black or African American, White, Hispanic, Asian, Native Hawaiian or other Pacific Islander, American Indian or Alaskan Native, Biracial (specify), or Other(specify). Gestational age at birth was obtained from birth records. No participant data were excluded after the initiation of data analysis. All participants gave written consent and the original consent documentation permitted the extended use of data and samples collected during the ADORE trial for secondary research.

### Procedures

A total of 492 and 540 participants (1,032 total) completed the respective arms of 200 mg and 1,000 mg/d of DHA (Fig 1). Blood was obtained from participants at baseline and at birth and separated by centrifugation into three fractions: RBCs, plasma, and white cells (buffy coat), which appear at the interface between RBCs and plasma. All three fractions were stored at −80 °C until analysis. Informed consent for genetic analysis was optional. Of the original cohort, 828 consented to a secondary study of genes related to the status of DHA. From these, 761 had complete data (gestational age, DNA for analysis, and fatty acid profile). The 761 participants comprised 365 of 492 participants (74.2%) assigned to 200 mg/d DHA and 396 of 540 (73.3%) assigned to 1,000 mg/d DHA.

**Fig 1:**
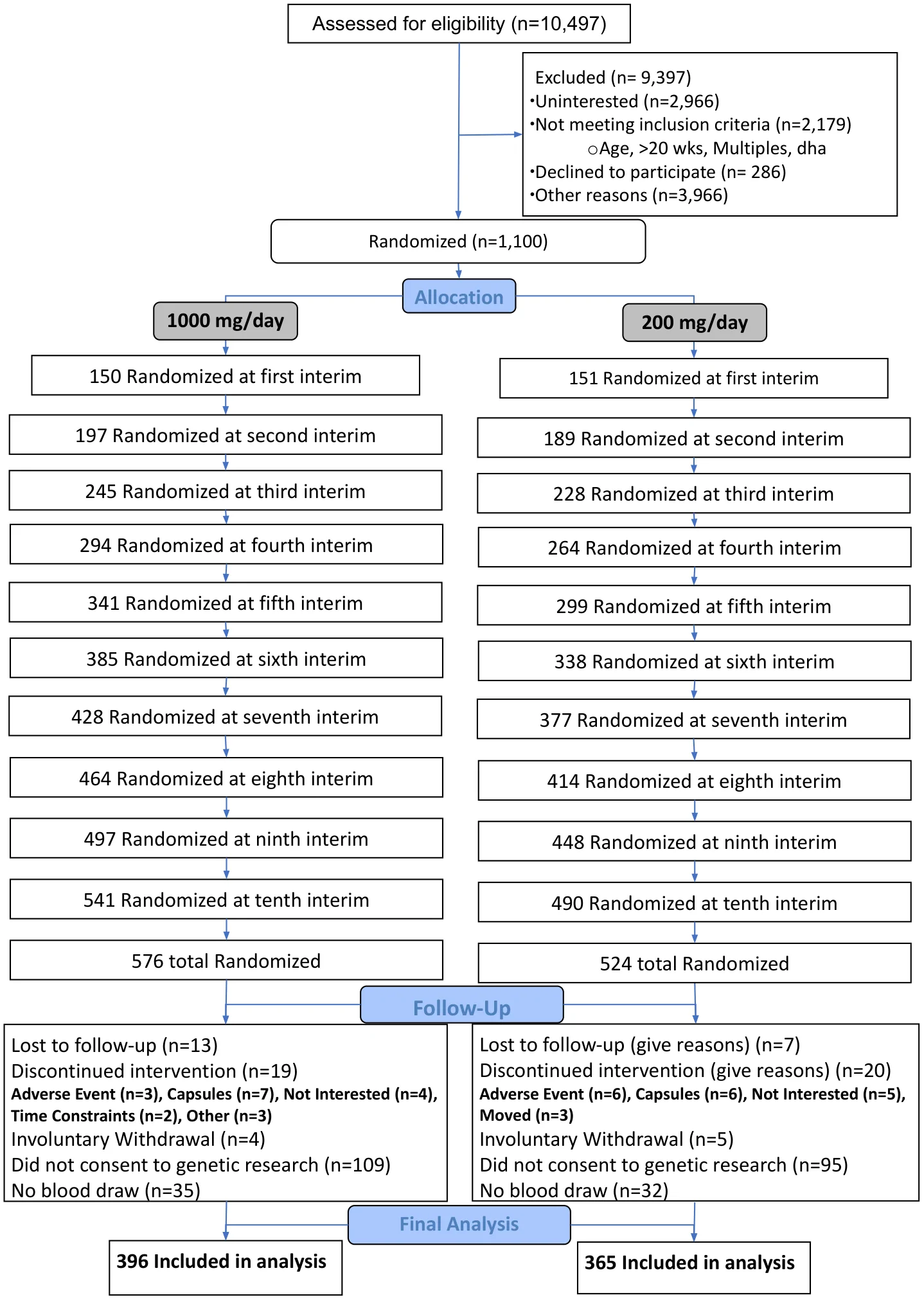
CONSORT diagram.

The white cells were transferred to the University of Texas at Austin on dry ice in late 2023 for FADS Indel genotyping. No information about participants was available to the genotyping group until after genotyping was completed. Genotypes were determined according to previous methods [19]. Briefly, genomic DNA is isolated from the white cell-enriched buffy coat and quality-checked by absorption at 260/280 nm. PCR with primers to amplify the Indel region generates 629 bp (I) or 607 bp (D) fragments. The presence of 607 bp (D/D), 607 and 629 bp (I/D), or 629 bp (I/I) is determined by gel electrophoresis.

RBC-PL fatty acids for participants of the ADORE trial were analyzed at the University of Kansas Medical Center by gas-liquid chromatography soon after the blood draw, and those determinations were used in our present analysis. The methods are published with the primary outcomes of that trial [22].

### Outcomes

The primary outcome of this secondary analysis is the effect of high-dose DHA on the PB and EPB rates of participants carrying at least one FADS Indel I allele. Prolonged gestation, defined as birth at ≥40 weeks of gestation, was assessed as a secondary outcome with respect to genotype and dose. Other secondary outcomes were the relationships between maternal race and gestational age, maternal circulating fatty acid profiles, and the association between cervical length and gestational age, all with respect to FADS Indel genotype. Adverse events were death, life-threatening events, hospital admission, incapacity or congenital anomaly, birth defect, and other adverse events related to DHA intake. These are published with the results of the ADORE trial [22].

### Statistical analysis

Bayesian statistics were modeled analogously to the treatment-specific Bayesian binomial model used for primary efficacy analysis in the original ADORE trial [22]. Bayesian non-hierarchical logistic binomial models with a weakly informative prior (a Normal prior on the log-odds with mean 0 and standard deviation 5, applied independently within each genotype and dose group and identical across all models) were fitted using the independent Bayesian design approach described in Berry and colleagues [28]. This approach avoids borrowing-induced type I error inflation when true effects differ between groups. We ran four Hamiltonian Monte Carlo chains for 4,000 iterations each, using 1,000 warm-up iterations for adaptation and retaining 12,000 post-warmup draws for inference. Convergence was confirmed for every model (potential scale reduction factor ≤1.01, effective sample sizes in the thousands, and no divergent transitions; Table A in S1 Appendix). Posterior predictive checks showed no evidence of misfit, and representative trace plots and posterior predictive checks are shown in S1 and S2 Figs. The results were insensitive to the prior: refitting under tighter and more diffuse priors (standard deviation 2.5 and 10 on the log-odds scale) changed every posterior probability by 0.05 or less (Table B in S1 Appendix), and prior-versus-posterior overlays for the smallest (I/I) subgroups confirm that the data, rather than the prior, primarily inform the estimates (S3 Fig). We evaluated the effect of DHA dose (200 mg/d versus 1,000 mg/d) on EPB and PB across FADS Indel genotypes using Bayesian logistic binomial models implemented in Stan via the rstan interface in R v4.2.3. The model provided posterior probability (PP) that 1,000 mg/d DHA reduced EPB and PB rates compared to 200 mg/d, stratified by genotype. No additional covariates were included, and no variable-selection procedure was used; the model structure was specified a priori to mirror the parent trial, and potential confounding of the non-randomized genotype comparisons was addressed through the inverse-probability-weighting sensitivity analysis described below. Missing data was minimal, all variables in the primary Bayesian models (gestational age at birth, FADS Indel genotype, DHA dose, and maternal race) were complete, so no imputation or distributional modeling of missing data was required. Potential selection bias arising from non-consent to genotyping was addressed by the inverse-probability-weighting sensitivity analysis. We further analyzed the interaction between maternal race, DHA dose, and FADS Indel genotypes, and PP that PB and EPB rates were higher in the I-allele group than in the D/D group. For the genotype contrast, participants with I/D and I/I genotypes were combined as the I-allele group to improve precision, this grouping does not imply equivalent biological responses. As a sensitivity analysis we additionally fitted a hierarchical (partial-pooling) model across the three genotypes, which produced more conservative subgroup estimates without altering the principal findings (Table C in S1 Appendix).

Posterior draws of the 200 mg/d and 1,000 mg/d treatments are binned on a common set of equal-width breaks to generate overlaid frequency histograms for visualization of the comparison of posterior distributions of participants within a genotype stratified by treatment groups (200 mg versus 1,000 mg DHA). Kaplan–Meier survival curves were generated using the survfit function in R, stratified by FADS Indel genotype and treatment groups. All figures are generated using R v4.2.3 and finalized in Inkscape 1.4.

We conducted a sensitivity analysis to assess potential bias introduced by excluding participants who did not consent or lacked genotype data using stabilized inverse probability weights (IPW) within each treatment group. The inclusion probability was modeled with logistic regression, using center, maternal race, baseline ARA/DGLA, and baseline RBC-PL DHA percent in the denominator, and center and maternal race in the numerator. Stabilized weights were truncated at the 1st–99th percentiles and incorporated into the Bayesian non-hierarchical logistic-binomial models. To assess robustness, we explored alternative denominator models (center + race; center + race + baseline ARA/DGLA + baseline RBC-PL DHA percent; center×race interaction + baseline ARA/DGLA + baseline RBC-PL DHA percent) and truncations (none, 1st–99th, 2nd–98th percentiles). A tipping point analysis was conducted through incrementally introducing selection bias on genotypes only by down-weighing the I-allele group by 1/Γ (Γ > 1) while the D/D group remained at 1. The stratum is then centered to ensure the stratum mean stayed at 1. We then conducted an outcome-tilted analysis within the I-allele group that multiplied I allele term birth by Γ, and I allele PB/EPB by 1/Γ, followed by the recentering of the stratum. The Bayesian model described above is refitted at each setting to record when the PP that PB or EPB rates are higher in the I-allele group than in the D/D group fall below 0.9 (Tables D–G in S1 Appendix).

Our reporting follows the CONSORT 2025 statement and the STROBE guidelines. Outcomes are summarized as *n*/*N* (%) with posterior means and 95% Bayesian credible intervals (CrIs). Between-group comparisons are reported as risk ratios (RRs) or fold-differences with 95% CrIs and the posterior probability (PP) of the stated direction. Secondary measures (e.g., cervical length) were compared between dose arms with a Welch two-sample *t* test, reported as the mean difference with a 95% confidence interval (CI) and a p-value. Baseline characteristics were compared across the three FADS Indel genotypes using *χ*^*2*^ tests for categorical variables and one-way analysis of variance for continuous variables to assess balance across the non-randomized genotype groups. We use Bayesian 95% CrI (credible intervals) designated in brackets with a dash throughout the text and reserve “95% CI” for this frequentist confidence interval.

### Use of AI disclosure

Artificial intelligence tools. OpenAI ChatGPT GPT-5 was used for limited assistance with developing and debugging code used in the statistical analyses. All AI-assisted code and resulting analytical outputs were reviewed and verified by a statistician/author to ensure their accuracy, integrity, and validity. The AI tool did not independently interpret the findings or determine the study conclusions. The authors retain full responsibility for the analyses and final manuscript.

## Results

Out of the 10,497 women screened for eligibility from June 8th 2016 to March 13th 2020, 3,966 were not assessed for interest or eligibility, predominantly because staffing limitations at the Ohio sites prevented the research team from approaching all potentially eligible women attending clinic. Of the remaining candidates,1,100 consented to the trial and 1,032 participants completed the ADORE trial and had birth data available. Of the 1,032 participants, 761 consented to genetic testing and had complete data on FADS Indel, RBC-PL fatty acids at baseline and delivery, gestational age at birth, and maternal race. All are included in the analyses we report here (Tables 1 and 2). EPB among the 761 participants was similar to that in the U.S., with a mean of 2.2% for singleton births (17/761) [26].

**Table 1:** Baseline characteristics of the ADORE cohort with a complete fatty acid and gestational age profile, and consented to genetic analysis.

	Total Sample (*n* = 761)	200 mg/day (*n* = 365; 48%)	1,000 mg/day (*n* = 396; 52%)
**Site, *n* (%)**			
The University of Kansas Medical Center	389 (51.1%)	192 (52.6%)	197 (49.7%)
University of Cincinnati Medical Center	183 (24%)	82 (22.5%)	101 (25.5%)
Ohio State University	189 (24.8%)	91 (24.9%)	98 (24.7%)
**Age at enrollment, yrs, mean ± SD**	30.3 ± 5.7	30.2 ± 5.7	30.4 ± 5.6
**Baseline DHA status, mean ± SD** ^**a**^	6.3 ± 1.7	6.4 ± 1.7	6.2 ± 1.7
**Pre-pregnancy BMI, kg/m** ^**2**^ **, mean ± SD**	28.1 ± 7.2	28.1 ± 7.1	28.1 ± 7.3
**Marital Status, *n* (%)**			
Unmarried/Single	253 (33.2%)	124 (34%)	129 (32.6%)
Married/Partnered	487 (64%)	232 (63.6%)	255 (64.4%)
Separated	10 (1.3%)	2 (0.5%)	8 (2%)
Divorced	11 (1.4%)	7 (1.9%)	4 (1%)
**Maternal Race, *n* (%)**			
American Indian or Alaskan Native	4 (0.5%)	2 (0.5%)	2 (0.5%)
Asian	17 (2.2%)	7 (1.9%)	10 (2.5%)
Biracial: Asian, White	4 (0.5%)	3 (0.8%)	1 (0.3%)
Biracial: Black, White	8 (1.1%)	5 (1.4%)	3 (0.8%)
Black or African American	156 (20.5%)	79 (21.6%)	77 (19.4%)
Hispanic	190 (25%)	86 (23.6%)	104 (26.3%)
Multiracial: Black, Asian, White	1 (0.1%)	1 (0.3%)	0 (0%)
Multiracial: Black, Native American, White	2 (0.3%)	0 (0%)	2 (0.5%)
Native Hawaiian or other Pacific Islander	1 (0.1%)	0 (0%)	1 (0.3%)
Other, specify	4 (0.5%)	3 (0.8%)	1 (0.3%)
White	374 (49.1%)	179 (49%)	195 (49.2%)
**Maternal Education, *n* (%)**			
Less than high school	134 (17.6%)	57 (15.6%)	77 (19.4%)
High school graduate or equivalent	161 (21.2%)	79 (21.6%)	82 (20.7%)
Some college or tech school	141 (18.5%)	67 (18.4%)	74 (18.7%)
Bachelor’s degree	167 (21.9%)	94 (25.8%)	73 (18.4%)
Master’s degree	106 (13.9%)	46 (12.6%)	60 (15.2%)
Doctorate	52 (6.8%)	22 (6%)	30 (7.6%)
**Annual Household Income, *n* (%)**			
Less than $15,000	162 (21.3%)	78 (21.4%)	84 (21.2%)
$15,000–$24,999	112 (14.7%)	49 (13.4%)	63 (15.9%)
$25,000–$49,999	134 (17.6%)	66 (18.1%)	68 (17.2%)
$50,000–$99,999	135 (17.7%)	66 (18.1%)	69 (17.4%)
$100,000–$149,999	121 (15.9%)	60 (16.4%)	61 (15.4%)
At least $150,000	79 (10.4%)	39 (10.7%)	40 (10.1%)
Unknown	18 (2.4%)	7 (1.9%)	11 (2.8%)
**Ever Smoker, yes, *n* (%)**	194 (25.5%)	92 (25.2%)	102 (25.8%)
Smoke 6mo prior, yes, *n* (%)	98 (12.9%)	53 (14.5%)	45 (11.4%)
Current smoker, yes, *n* (%)	40 (5.3%)	22 (6%)	18 (4.5%)
**Pregnancy History, *n* (%)**			
Primagravida	219 (28.8%)	107 (29.3%)	112 (28.3%)
Prior preterm birth ^b^	104 (19.2%)	52 (20.2%)	52 (18.3%)
Prior early preterm birth (<34 weeks) ^b^	45 (8.3%)	23 (8.9%)	22 (7.7%)

^a^Mean% RBC total fatty acid.

^b^Participants with a prior pregnancy.

ADORE, Assessment of DHA on Reducing Early Preterm Birth; BMI, body mass index; DHA, docosahexaenoic acid; RBC, red blood cell; SD, standard deviation.

**Table 2:** Preterm birth (PB) and early preterm birth (EPB) events overall, by treatment arm, and by FADS Indel genotype.

	*N*	PB (<37 wk), *n* (%)	EPB (<34 wk), *n* (%)
***By treatment arm***			
200 mg/d	365	44 (12.1%)	10 (2.7%)
1,000 mg/d	396	30 (7.6%)	7 (1.8%)
***By FADS Indel genotype***			
D/D	342	24 (7.0%)	6 (1.8%)
I/D	313	36 (11.5%)	7 (2.2%)
I/I	106	14 (13.2%)	4 (3.8%)
**Total**	761	74 (9.7%)	17 (2.2%)

When receiving low-dose DHA, PB and EPB rates were 1.7-fold and 5.4-fold higher, respectively, in the FADS Indel I-allele group (I/D and I/I) than in the D/D group (PB: I-allele group: 31/214, 14.5% (95% CrI [10.1%, 19.5%]); D/D: 13/151, 8.7% (95% CrI [4.7%, 13.6%]); RR 1.7 (95% CrI [0.94, 3.25]; PP = 0.96). EPB: I-allele group: 9/214, 4.3% (95% CrI [2.0%, 7.3%]); D/D: 1/151, 0.8% (95% CrI [0.04%, 2.7%]); RR 5.4 (95% CrI [1.3, 95]; PP = 0.99)) (Fig 2a).

**Fig 2:**
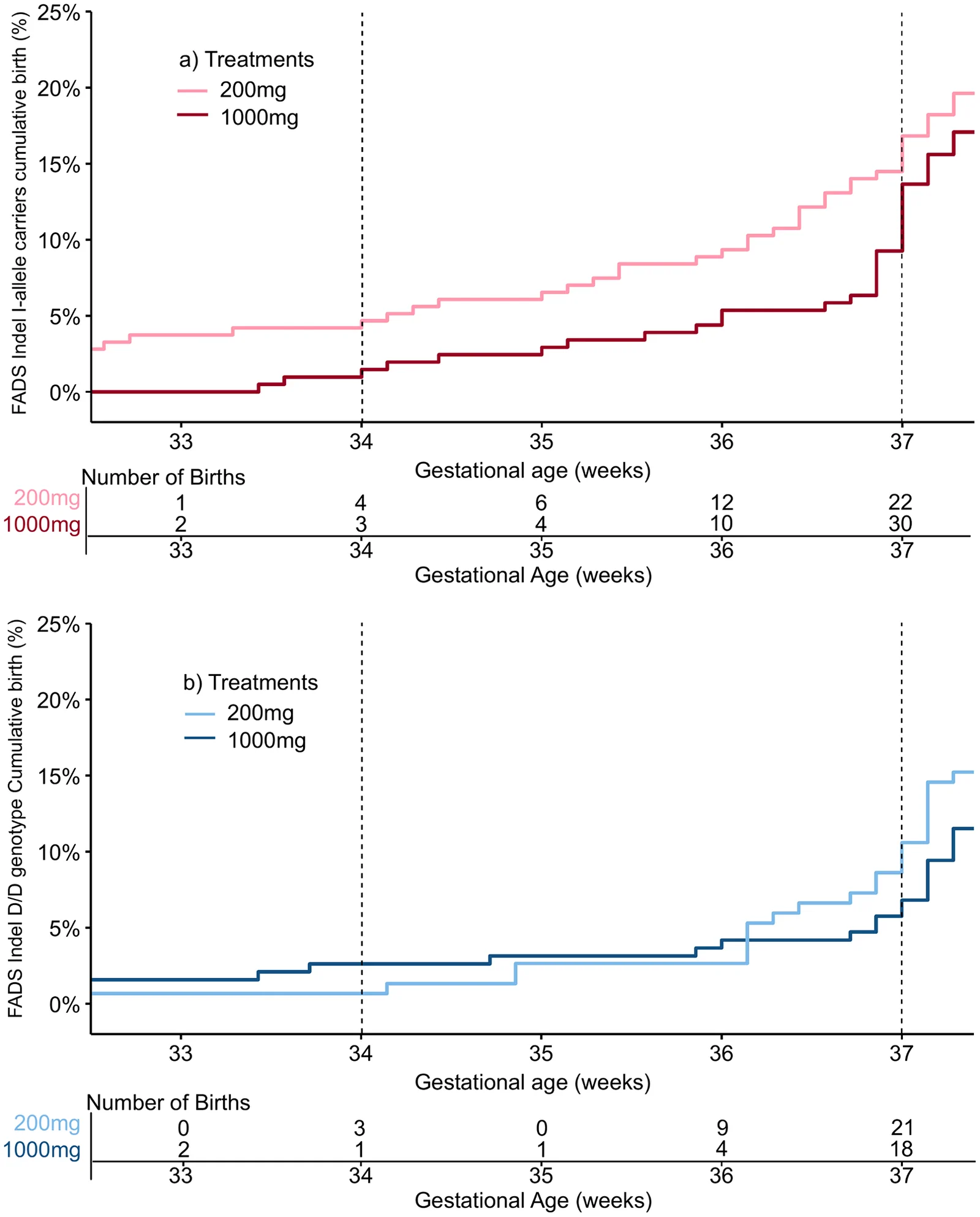
Cumulative births (%) comparing FADS Indel genotypes receiving the same dose. **(a)** On low-dose (200 mg/d) DHA, preterm birth (PB, <37 weeks) rates were 1.7-fold higher in the I-allele group (I/D + I/I) than in the D/D group. Posterior mean PB were 14.5% (95% CrI [10.1%, 19.5%])CrI for I/D + I/I, and 8.7% (95% CrI [4.7%, 13.6%])CrI for D/D, Bayesian PP = 0.96. Early preterm birth (EPB, <34 weeks) rates were 5.4-fold higher in the I-allele group than in the D/D group. Posterior mean EPB were 4.3% (95% CrI [2.0%, 7.3%])CrI for I/D + I/I and 0.8% (95% CrI [0.04%, 2.7%])CrI for D/D, with Bayesian PP = 0.99. **(b)** On high-dose (1,000 mg/d) DHA, PB rates were 1.6-fold higher in the I-allele group than in the D/D group. Posterior mean PB were 5.8% CrI(95% CrI [3.0%, 9.5%]) for D/D and 9.3% CrI(95% CrI [5.7%, 13.6%]) for I/D + I/I, PP = 0.91. EPB was greatly attenuated in the D/D group so that total EPB was a fraction of that in the low-dose group. Posterior mean EPB rates were 2.7% CrI(95% CrI [0.9%, 5.4%]) for D/D and 1.1% CrI(95% CrI [0.2%, 2.8%]) for I/D + I/I, with Bayesian PP = 0.10.

When receiving high-dose DHA, the difference in EPB between the I-allele and D/D groups was substantially attenuated relative to the low dose, whereas PB rates remained higher in the I-allele group than in the D/D group (PB: I-allele group: 19/205, 9.3% (95% CrI [5.7%, 13.6%]); D/D: 11/191, 5.8% (95% CrI [3.0%, 9.5%]); RR 1.6 (95% CrI [0.80, 3.46]; PP = 0.91). EPB: I-allele group: 2/205, 1.1% (95% CrI [0.2%, 2.8%]); D/D: 5/191, 2.7% (95% CrI [0.9%, 5.4%]); RR 0.40 (95% CrI [0.05, 1.69]; PP = 0.10)) (Fig 2b).

High-dose DHA compared to low dose DHA reduced both PB and EPB in the FADS Indel I-allele group (I/I + I/D): PB (1,000 mg/d: 19/205, 9.3% (95% CrI [5.7%, 13.6%]); 200 mg/d: 31/214, 14.5% (95% CrI [10.1%, 19.5%]); risk ratio (RR) 0.64 (95% CrI [0.36, 1.08]; PP = 0.95); 36% lower); EPB (1,000 mg/d: 2/205, 1.1% (95% CrI [0.2%, 2.8%]); 200 mg/d: 9/214, 4.3% (95% CrI [2.0%, 7.3%]); RR 0.25 (95% CrI [0.03, 0.87]; PP = 0.99); 75% lower) (Fig 3a). In individual I allele genotypes, high-dose DHA reduced PB among participants with the I/D genotype from 15.0% (24/160; 95% CrI [10.0, 21.0]) to 7.8% (12/153; 95% CrI [4.2, 12.6]), a rate below the US average of 10.4% (RR 0.53 (95% CrI [0.26, 0.98]; PP = 0.98)). PB rates did not differ by dose in the I/I group (200 mg/d: 7/54, 13.1% (95% CrI [5.6%, 23.2%]); 1,000 mg/d: 7/52, 13.6% (95% CrI [5.8%, 24.0%]); RR 1.04 (95% CrI [0.38, 2.86]; PP = 0.47)), but high-dose DHA reduced EPB by almost 3-fold (200 mg/d: 3/54, 5.6% (95% CrI [1.3%, 13.3%]); 1,000 mg/d: 1/52, 1.9% (95% CrI [0.1%, 7.5%]); RR 0.39 (95% CrI [0.02, 2.46]; PP = 0.86)). (S4 Figs, Table H in S1 Appendix, S5 Fig, Table I in S1 Appendix).

**Fig 3:**
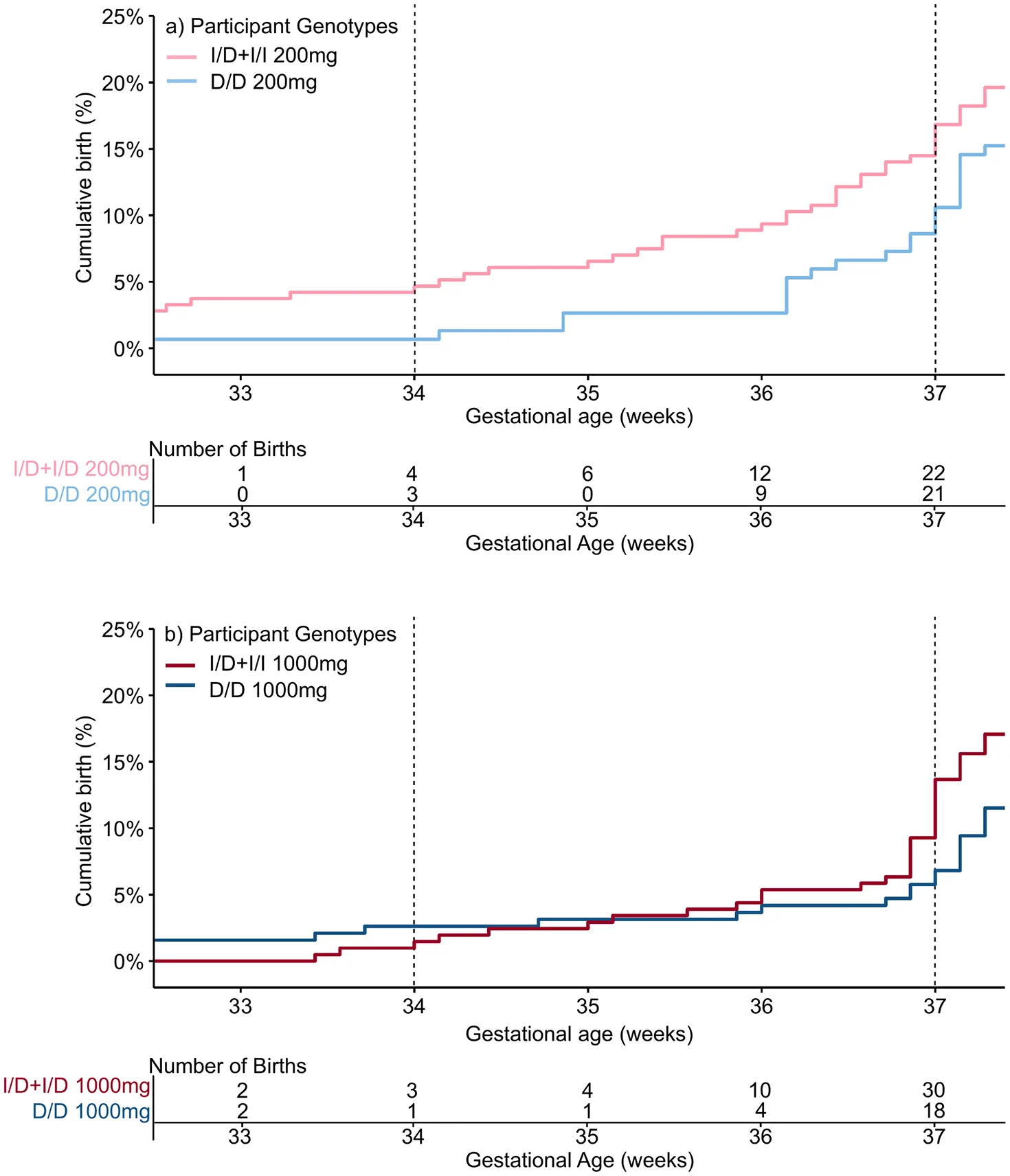
Cumulative births (%) comparing doses within FADS Indel genotypes. **(a)** In the I-allele group (I/D + I/I), participants receiving 200 mg DHA/d had more early preterm birth (EPB, <34 weeks) and preterm births (PB, <37 weeks) than participants who received 1,000 mg DHA/d. Posterior mean EPB were 1.1% CrI(95% CrI [0.2%, 2.8%]) for 1,000 mg, and 4.3% CrI(95% CrI [2.0%, 7.3%]) for 200 mg, with Bayesian PP = 0.99. Posterior mean PB were 9.3% CrI(95% CrI [5.7%, 13.6%]) for 1,000 mg, and 14.5% CrI(95% CrI [10.1%, 19.5%]) for 200 mg, with Bayesian PP = 0.95. **(b)** In the D/D group, participants receiving 200 mg of DHA/day had less EPB and more PB compared to those who received 1,000 mg. Posterior mean EPB rates were 2.7% CrI(95% CrI [0.9%, 5.4%]) for 1,000 mg, and 0.8% CrI(95% CrI [0.04%, 2.7%]) for 200 mg, with Bayesian PP = 0.07. Posterior mean PB rates were 5.8% CrI(95% CrI [3.0%, 9.5%]) for 1,000 mg, and 8.7% CrI(95% CrI [4.7%, 13.6%]) for 200 mg, with Bayesian PP = 0.85.

Participants with the D/D genotype had the lowest rates of PB among all genotypes (Table 2). High dose DHA resulted in a further small decrease in PB (1,000 mg/d: 11/191, 5.8% (95% CrI [3.0%, 9.5%]); 200 mg/d: 13/151, 8.7% (95% CrI [4.7%, 13.6%]); RR 0.67 (95% CrI [0.30, 1.45]; PP = 0.85)) (Fig 3b), but increased EPB compared to low-dose (1,000 mg/d: 5/191, 2.7% (95% CrI [0.9%, 5.4%]); 200 mg/d: 1/151, 0.8% (95% CrI [0.04%, 2.7%]); PP = 0.07). This finding is similar to a finding in the ORIP trial that high-dose DHA increased EPB in individuals entering the trial with the highest omega-3 status [29], though our results should be interpreted cautiously because of the low number of events.

On high-dose DHA, births beyond 40 weeks were 1.59-fold more frequent in the D/D group than in the I-allele group (D/D: 49/191, 25.7% (95% CrI [19.8%, 32.1%]); I-allele group: 33/205, 16.1% (95% CrI [11.4%, 21.4%]); RR 1.59 (95% CrI [1.08, 2.40]; PP = 0.99)).On low-dose DHA, births after 40 weeks did not differ between the D/D and I-allele groups: D/D: 23/151,15.3% (95% CrI [10.0%, 21.4%]); I-allele group: 38/214, 17.8% (95% CrI [13.0%, 23.1%]); RR 0.86 (95% CrI [0.52, 1.36]; PP = 0.26) (S8a, S8b, S9a, S9b and S10 Figs).

The results above show that high-dose DHA improved PB and EPB rates in the I-allele group to a greater degree than in D/D. Consistently, high-dose DHA prolonged gestation in D/D and may also result in a minor increase in D/D EPB rates.

To test directly whether the effect of DHA dose depended on FADS Indel genotype, we regrouped the randomized arms into genotype-concordant and genotype-discordant dosing; the original ADORE trial did not assign dose by genotype. We computed the posterior probability of the genotype × dose interaction from the joint posterior draws of the four independent genotype × dose arms. The dose effects credibly diverged for EPB and prolonged gestation. High-dose DHA reduced EPB in the I-allele group but increased it in the D/D group, based on a low number of events: interaction log-OR 3.17 (95% CrI [0.58, 6.27]; PP = 0.99). For prolonged gestation (≥40 weeks), high-dose DHA increased rates in the D/D group with no effect in the I-allele group, interaction log-OR 0.78 (95% CrI [0.04, 1.54]; PP = 0.98). Dose effects for PB were similar, with high-dose DHA associated with a similar reduction in both genotype groups (RR 0.64 in the I-allele group versus 0.67 in D/D), and the interaction was inconclusive: interaction log-OR 0.08 (95% CrI [−0.93, 1.12]; PP = 0.56). Genotype-informed dosing benefited all genotypes.

As expected from genotype frequencies, mean PB and EPB rates were higher among Black participants compared to non-Black participants: PB (Black: 25/156, 16.1% (95% CrI [10.8%, 22.2%]); non-Black: 49/605, 8.1% (95% CrI [6.1%, 10.4%]); RR 2.0 (95% CrI [1.24, 3.06]; PP = 0.99)) (S6 Fig, Table J in S1 Appendix); EPB (Black: 7/156, 4.5% (95% CrI [1.9%, 8.3%]); non-Black: 10/605, 1.7% (95% CrI [0.8%, 2.9%]); RR 2.7 (95% CrI [0.98, 6.93]; PP = 0.97)). Among Black participants, all cases of EPB occurred exclusively in the I-allele group (S7 Fig, Table K in S1 Appendix). Based on these observations, we evaluated the effects of matching participants in the low-ARA-synthesis D/D group and high-ARA-synthesis I-allele group with low and high doses of DHA, respectively, on pre- and post-40-week births. Consider first *mis*matched dosing: D/D 1,000 mg/d, I-allele 200 mg/d. PB rates were lower in the D/D group than in the I-allele group, and prolonged gestation was more frequent in the D/D group. PB: (D/D: 11/191, 5.8% (95% CrI [3.0%, 9.5%]); I-allele group: 31/214, 14.5% (95% CrI [10.1%, 19.5%]); RR 0.40 (95% CrI [0.19, 0.74]; PP = 0.99)). EPB: (D/D: 5/191, 2.7% (95% CrI [0.9%, 5.4%]); I-allele group: 9/214, 4.3% (95% CrI [2.0%, 7.3%]); RR 0.63 (95% CrI [0.19, 1.75]; PP = 0.81)). Prolonged gestation: D/D: 49/191, 25.7% (95% CrI [19.8%, 32.1%]); I-allele group: 38/214, 17.8% (95% CrI [13.0%, 23.1%]), RR 1.44 (95% CrI [1.00, 2.12]; PP = 0.97)) (Fig 4a). In contrast, for *matched* dosing: 200 mg/d for the D/D group, 1,000 mg/d for the I-allele group, PB and EPB were indistinguishable across genotypes and prolonged gestations did not differ. PB: D/D 13/151, 8.7% (95% CrI [4.7%, 13.6%]); I-allele group 19/205, 9.3% (95% CrI [5.7%, 13.6%]), RR 1.07 (95% CrI [0.55, 2.18]; PP = 0.58). EPB: D/D 1/151, 0.8% (95% CrI [0.04%, 2.7%]); I-allele group 2/205, 1.1% (95% CrI [0.2%, 2.8%]), RR 1.34 (95% CrI [0.15, 27.0]; PP = 0.64). Prolonged gestation: D/D: 23/151, 15.3% (95% CrI [10.0%, 21.4%]); I-allele group: 33/205, 16.1% (95% CrI [11.4%, 21.4%]), RR 0.95 (95% CrI [0.57, 1.53]; PP = 0.41) (Fig 4b).

**Fig 4:**
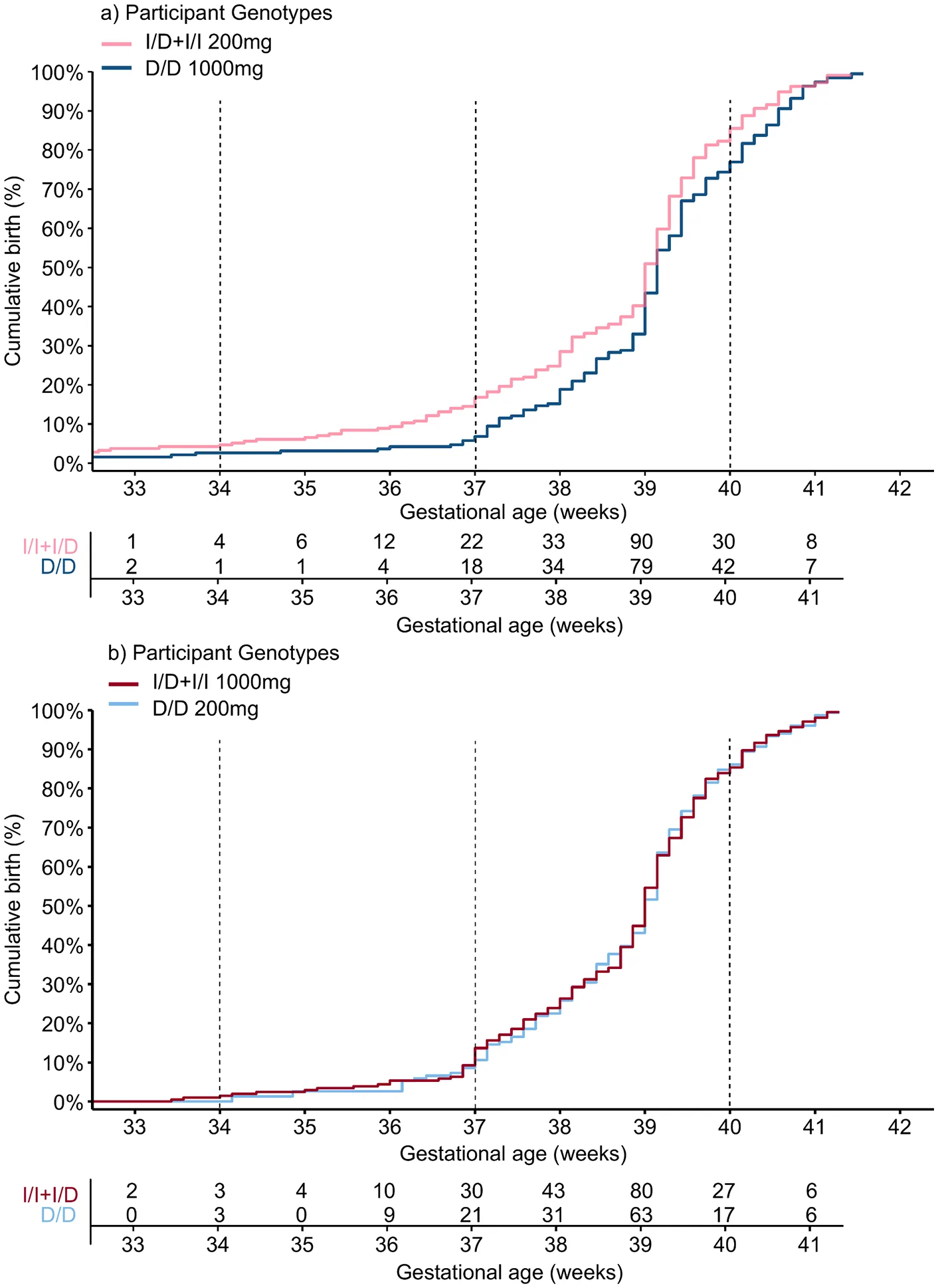
Cumulative births (%) comparing FADS Indel genotypes receiving matched, and mismatched dose. **(a)** Mismatched dose-genotype. PB and EPB rates were higher in the low-dose I-allele group than in the high-dose D/D group, whereas prolonged gestation was more frequent in the high-dose D/D group. Posterior mean PB rates were 5.8% (95% CrI [3.0%, 9.5%]) for high-dose D/D, and 14.5% (95% CrI [10.1%, 19.5%]) for the low-dose I-allele group, with Bayesian PP = 0.99. Posterior mean EPB rates were 2.7% (95% CrI [0.9%, 5.4%]) for high-dose D/D, and 4.3% (95% CrI [2.0%, 7.3%]) for the low-dose I-allele group, with Bayesian PP = 0.81. Posterior mean prolonged gestation rates were 25.7% (95% CrI [19.8%, 32.1%]) for high-dose D/D, and 17.8% (95% CrI [13.0%, 23.1%]) for the low-dose I-allele group, with Bayesian PP = 0.97. **(b)** Matched dose-genotype. Birth rates were comparable between the high-dose I-allele group and the low-dose D/D group, and prolonged gestation did not meaningfully differ. Posterior mean PB rates were 8.7% (95% CrI [4.7%, 13.6%]) for low-dose D/D, and 9.3% (95% CrI [5.7%, 13.6%]) for the high-dose I-allele group, with Bayesian PP = 0.58. Posterior mean EPB rates were 0.8% (95% CrI [0.04%, 2.7%]) for low-dose D/D, and 1.1% (95% CrI [0.2%, 2.8%]) for the high-dose I-allele group, with Bayesian PP = 0.64. Posterior mean prolonged gestation rates were 15.3% (95% CrI [10.0%, 21.4%]) for low-dose D/D, and 16.1% (95% CrI [11.4%, 21.4%]) for the high-dose I-allele group, with Bayesian PP = 0.41.

We also characterized the relationship of the FADS Indel to the ratio of ARA (product) to DGLA (the immediate precursor of ARA). The ARA-to-DGLA ratio was higher in the I-allele group, and the ratio of ARA/DGLA increased from D/D to I/D and I/I, consistent with reports from all other populations studied (Fig 5) [19,20]. When receiving 200 mg DHA/d, the ratio for I/I was 30% higher than D/D at baseline, identical to what it was at delivery. When receiving 1,000 mg DHA/d, the ratio for I/I was 37% higher than D/D at baseline, and 39% higher at delivery. In other words, DHA dose did not meaningfully affect the rate of ARA synthesis. There were, however, apparent but subtle differences in the ARA/DGLA ratio between Black participants and non-Black participants in response to DHA supplementation. As expected, the ratio of ARA to DGLA was higher in Black participants than in non-Black participants at both baseline and at birth. The ARA to DGLA ratio also appears to decline with supplementation in both Black participants and non-Black participants, except for non-Black participants with the I/I genotype, who showed a slight increase in the ratio following supplementation (Table L in S1 Appendix).

**Fig 5:**
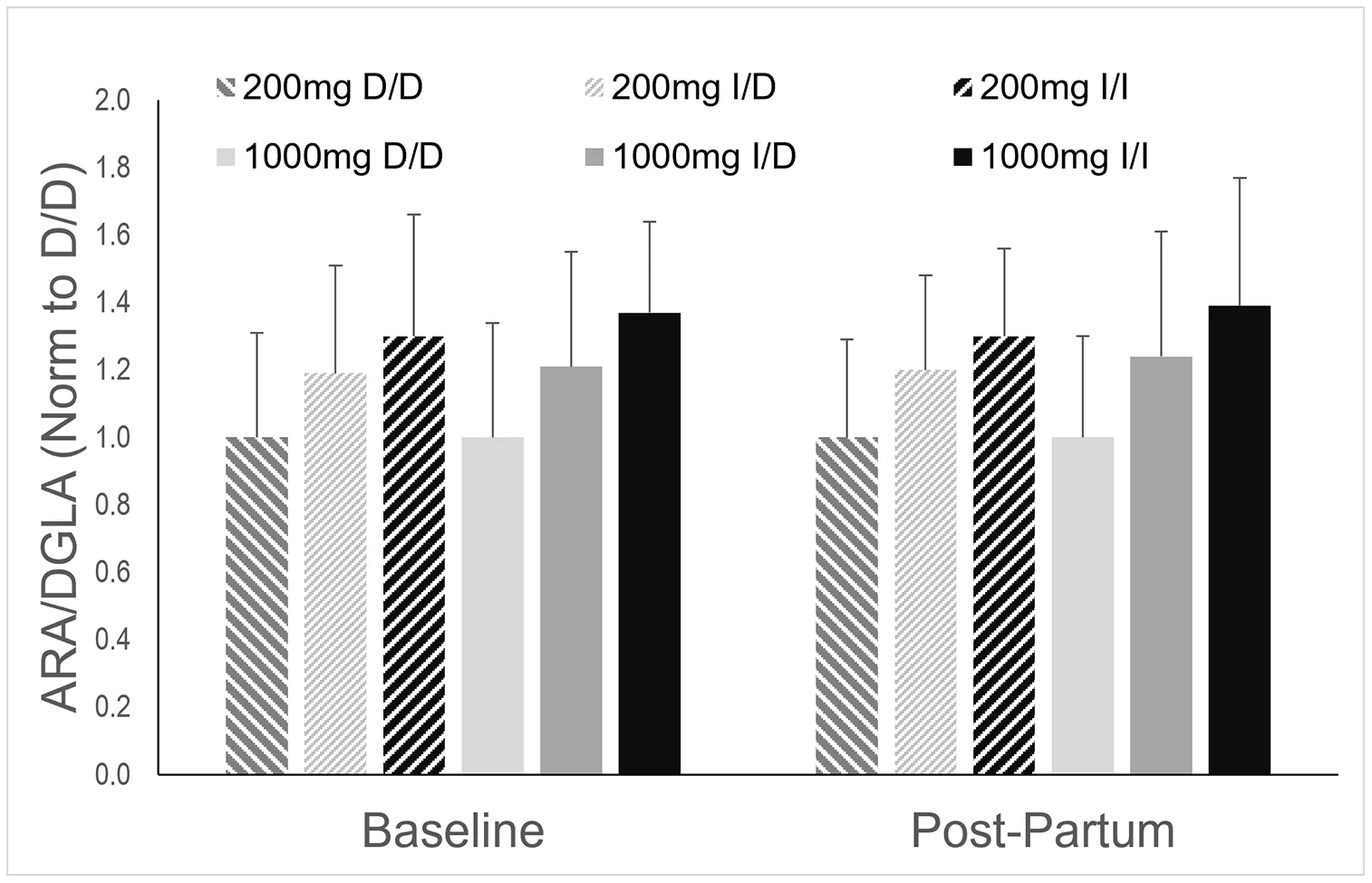
Relative circulating bioactive omega-6 fatty acids. Arachidonic acid (ARA) to di-homo-gamma-linolenic acid (DGLA) ratio by treatment and genotype, at baseline and birth.

As expected, DHA supplementation decreased the ratio of ARA to DHA between baseline and birth regardless of allele category, with a larger decrease in those receiving 1,000 mg compared to 200 mg of DHA (Table L in S1 Appendix). The ARA to DHA ratio is higher in Black than in non-Black at baseline and birth (Table L in S1 Appendix). We confirmed that Black participants in our study were far more likely to carry at least one copy of the I allele (85.3%, *n* = 133/156), with 35.9% (*n* = 56/156) exhibiting the I/I genotype. In contrast, among non-Black participants, 47.3% (*n* = 286/605) carried at least one copy of the I allele, and only 8.3% (*n* = 50/605) had the I/I genotype. The posterior probability (PP) was 1.0, indicating a clear ancestral difference (Fig 6), and US regional difference (S11 Fig) in the prevalence of both I/D and I/I genotypes. Baseline RBC-PL levels of all measured fatty acids are provided by genotype in Table M in S1 Appendix. The FADS Indel effect was confined to the n-6 LCPUFA synthesis pathway (falling DGLA with rising ARA and DTAn-6 across D/D → I/D → I/I), whereas n-3 LCPUFAs (EPA, DHA) did not meaningfully differ by genotype.

**Fig 6:**
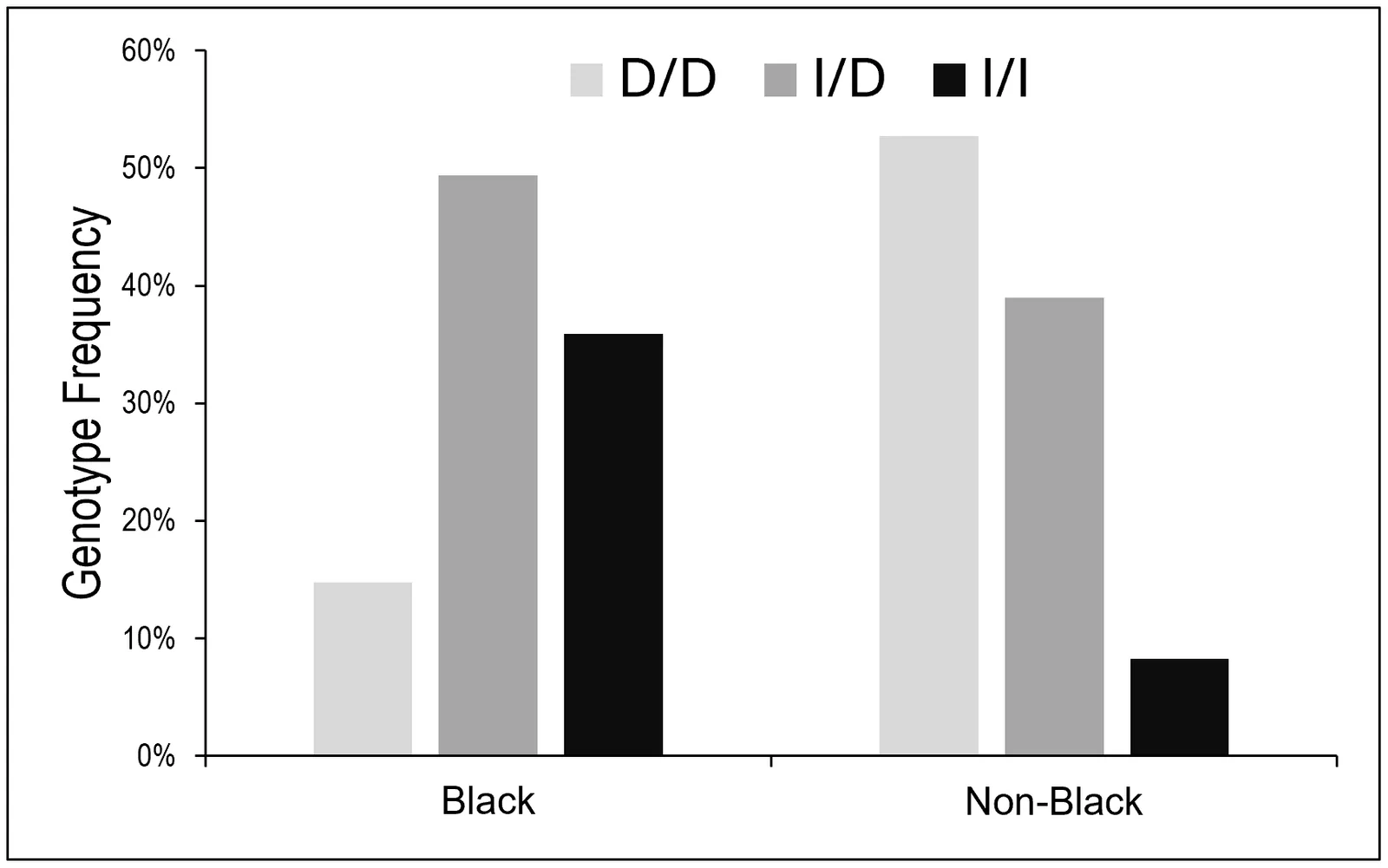
Distribution of FADS Indel genotypes from *n* = 761 ADORE participants. D/D Black: 23/156 (14.74%), D/D Non-Black: 319/605 (52.73%). I/D Black: 77/156 (49.36%), I/D Non-Black: 236/605 (39.01%). I/I Black: 56/156 (35.90%), I/I Non-Black: 50/605 (8.26%).

Cervical length data were collected between 18 and 22 weeks of gestation from 574 out of 761 participants with genetic results. Among these, 51/574 (8.9%) had PB and 11/574 (1.9%) had EPB. Eight of 574 (1.4%) had cervical lengths <25 mm. Of those who experienced PB or EPB, two cases could potentially be attributed to a short cervix: one with a cervical length of 18 mm delivered at a gestational age of 36.7 weeks. Another with a cervical length of 20 mm delivered at 33.4 weeks. Cervical length did not meaningfully differ by DHA dose: 38.2 mm at 200 mg/d versus 38.5 mm at 1,000 mg/d (mean difference, −0.3 mm; 95% CI [−1.4, 0.9]; p = 0.637; Welch two-sample t *t*est). Cervical length measurements were similar between Black participants (mean ± standard deviation, 38.9 ± 7.5 mm; 95% CI [37.5, 40.3]) and non-Black participants (mean ± standard deviation, 38.2 ± 6.8 mm; 95% CI [37.6, 38.9]), and thus it was not a major contributing factor to the higher observed EPB and PB in the Black participants.

Sensitivity analyses (Text A and Tables D–G and N in S1 Appendix) indicated that the genotype-related findings were not driven by participant selection and were directionally consistent across weighting, denominator, and truncation choices. The strength of evidence differed by outcome. In the outcome-tilted (worst-case) analysis, the early preterm birth association remained credible until a larger adverse tilt (Γ = 1.40), whereas the preterm birth association fell below a posterior probability of 0.90 with only a small tilt (Γ = 1.1, equivalent to ≈3–4 of the 31 carrier preterm birth cases) (Table N in S1 Appendix).

## Discussion

Preterm birth rates were higher in the FADS Indel I-allele group than in the D/D group and were lower with high-dose DHA (1,000 mg/d) than with low-dose DHA (200 mg/d) in the I-allele group. Separately, participants with either the I/D or I/I genotype experienced nearly 6-fold and 12-fold higher rates of EPB, respectively, compared to participants with the D/D genotype. Black participants had a higher PB and EPB, and we observed that they had a higher prevalence of the FADS Indel I allele compared to participants in all other self-reported race/ethnicity groups combined (PP > 0.999). Here, we are able to distinguish the effect of self-reported race/ethicity from genetic ancestry at the FADS Indel locus.

A 2018 Cochrane Systematic Review [13] found strong evidence that omega-3 fatty acids, particularly DHA, reduce EPB and PB. The pathophysiology leading to preterm birth often involves increased pro-inflammatory mediators, such as IL-6, IL-1β, IL-8, and TNF-α. IL-1β plays a major role in upregulating COX-2 expression, which then catalyzes the conversion of ARA to PGE2, and DGLA to PGE1. The late-gestation myometrium is abundant in prostaglandin EP1 and EP3 receptors, both of which have higher affinity for PGE2 than PGE1. The binding of these prostaglandins triggers Ca^2+^ mobilization and sensitization, resulting in forceful myometrial contractions [30]. Non-selective COX inhibitors like indomethacin and selective COX-2 inhibitors like celecoxib lower prostaglandin production, thus reducing the risk of preterm birth. We hypothesized that high-dose DHA would reduce EPB and PB in the I-allele group by balancing the excess conversion of ARA into the oxytocic PGE2, as well as conversion of DGLA into cervical effacement agent PGE1, the latter of which is administered therapeutically to induce labor as the synthetic version, dinoprostone. Our data are consistent with this mechanism.

We found in a planned secondary analysis of the ADORE trial that Black participants with the lowest rates of EPB and PB were those who began the trial with a baseline DHA level ≥6% and received high-dose DHA supplementation [25]. Their rates of EPB and PB were 2.1% and 6.4%, respectively. Considering FADS Indel genotypes alone in the current study, we find the rates of EPB in Black participants with the I/D and I/I genotypes declined from 10.5% to 2.6% and from 6.9% to 0%, respectively, with high-dose DHA (Table K in S1 Appendix); their rate of PB declined from 26.3% to 7.8% in the I/D genotype (Table J in S1 Appendix). Prior publications have focused on socioeconomic and environmental factors that are associated with racial disparities in preterm birth in the US [31]. The present study, supported by existing basic science and pharmacology, suggests a higher PB risk in US Black women in the lower half of DHA intake, driven by a higher prevalence of the FADS Indel I allele compared to those of European and other ancestries. Black participants in the ADORE trial who had high RBC-PL DHA at baseline, which is evidence of higher pre-enrollment DHA consumption, had low rates of EPB regardless of DHA dose (1.9% on 200 mg/d and 2.1% on 1,000 mg/d) [25].

Other interventions used to prevent preterm birth, such as vaginal progesterone or cerclage, are beneficial in only a small percentage of patients with applicable risk factors, such as short cervix or prior early preterm birth. However, DHA supplementation has demonstrated efficacy in preventing preterm birth when given to an unselected population, which has a larger potential benefit on a population level [13].

The ADORE trial demonstrated the efficacy of higher-dose DHA to prevent early PB among women with low DHA levels at baseline. Even if efficacy was limited to only those with low DHA status, a high proportion of participants might benefit, as 45.3% of participants in the ADORE trial had an RBC-PL DHA level lower than 6% at baseline.

Importantly, unlike invasive interventions such as cervical cerclage—which require early identification of cervical shortening and surgical placement typically in the first trimester—or vaginal progesterone, the use of which is predicated on detecting a sonographically short cervix in mid‐pregnancy, DHA supplementation is a non‐invasive, nutritional approach that applies to primary prevention in unselected populations. DHA supplementation can be implemented early in pregnancy to an unselected population as opposed to more costly screening approaches with universal ultrasound cervical screening, offering a pragmatic approach for preterm birth prevention.

Though this is a secondary analysis, these findings rest on firmer ground than observational association. Genetic analysis was anticipated in the original study design. The central contrast, high- versus low-dose DHA within a single genotype, is randomized. FADS Indel genotype is fixed at conception and balanced across dose arms (Table O in S1 Appendix), so the within-genotype dose effect is an unconfounded estimate of the intervention and is not subject to the ancestry- and socioeconomically-patterned confounding that drives preterm birth. Germline genotype is an immutable characteristic of individuals, thus there is no possible reverse causation (i.e., preterm birth causes the I allele). The upstream step, FADS Indel genotype to arachidonic acid synthesis, is consistent with several of Hill’s considerations for causation: the mechanism is a defined enzymatic reaction catalyzed by the FADS1 and FADS2 gene products [32]; arachidonic acid rises monotonically across D/D < I/D < I/I at all three study sites and matches all populations studied to date (Beijing China [21], Leeds, UK [20], Pune, India and a previous cohort in Kansas City [19]).

We measured one and only one polymorphism, selected a priori on this mechanism, with no other genetic test performed; the analysis therefore satisfies the prospective–retrospective framework under which a single, prospectively specified functional biomarker examined in archived trial specimens (retrospective) reaches an evidentiary tier above exploratory association. This is the path by which retrospective, genotype-defined subgroups of randomized trials, as with KRAS and anti-EGFR therapy, became practice-changing predictive biomarkers before prospective confirmation [23,24]. Similarly, the FADS Indel I allele distinguishes responders to EPA in colorectal polyp prevention [20]. The prolonged gestation we observe at high dose in D/D (PP = 0.99) parallel the prolonged gestation reported with omega-3 supplementation; the concurrent rise in EPB in D/D rests on few events but is directionally consistent with ORIP [29]. A randomized within-genotype effect, a replicated mechanistic gradient, controlled confounding, and independent clinical replication converge through methods whose biases do not overlap [33].

To our knowledge, the optimal dose to reduce EPB and PB has not been previously defined. We show here that 1,000 mg/d reduced PB most clearly in I/D group, with weaker and less certain evidence in the smaller I/I subgroup. Doses greater than 1,000 mg/d may be necessary to reduce the risk of PB in individuals with the highest capacity to synthesize ARA. Our data indicate that high doses are associated with prolonged gestation, as well as increased EPB in those with the D/D phenotype, based on few events. A Cochrane review similarly found prolonged gestation in studies from around the world, with FADS genotypes expected to be diverse [13]. Matching dose to genotype, in this case low-dose to DD, was associated with less prolongation of gestation in individuals who do not have excess EPB and PB risk. As to our observed increase in EPB in D/D on high-dose, the ORIP trial reported no overall reduction in EPB and a possible increase in EPB among participants with high-baseline omega-3 status with omega-3 fatty acid supplementation, which is consistent with our observation [29]. If a relatively high proportion of ORIP participants were in the D/D group, this could mute any overall benefit and increase the perceived risk of omega-3 supplementation. When the genotype is unknown, it is prudent to cease DHA supplementation at gestational age 37 weeks to reduce the risk of prolonged gestation, as indicated in a 2024 Clinical Practice Guideline [18]. Supplementation may be resumed postpartum to support maternal mental health and improve DHA transmission to the newborn.

The FADS Indel genotype prevalence varies by ancestry, which in turn implies that the results of any particular omega-3 fatty acid supplementation study will depend on the mixture of ancestries in the cohort. For example, extrapolating our results to cohorts of Black persons would reveal highly significant effects of high-dose DHA. In contrast, a study of an entirely European cohort would likely yield a much smaller effect. FADS Indel should be characterized in future studies of omega-3 supplementation.

Precision medicine posits that definable characteristics of groups or individuals, particularly genetics, guide treatment to improve medical efficacy and safety. The ADORE trial found a reduction in PB and, in those with low DHA status or intake at enrollment, a reduction in EPB. Our analysis identified both benefits and risks of high-dose DHA supplementation depending upon genotype. Participants who carry the I allele (55% of our cohort) benefited from high-dose DHA with lower rates of PB and EPB and did not have an increased risk of prolonged gestation; however, those who did not carry the I allele were at increased risk for both EPB and prolonged gestation. Our finding of increased EPB is in agreement with Simmonds and colleagues [29], whose exploratory analysis of the Australian ORIP trial found a higher risk of EPB in the group provided high-dose omega-3 fatty acid supplementation in a population that likely had a lower proportion of participants with the I allele. These findings support the use of genotype to inform DHA supplement level [34].

The same FADS Indel now linked to risk of EPB and PB was associated with response to EPA therapy in preventing recurring colorectal polyps [20]. Most clinical conditions are associated with inflammation [35], and many reports reveal benefits of omega-3 supplementation in these conditions [36]. People who synthesize more ARA are more likely to be subject to these medical issues, and plausibly could benefit from advice on increasing their omega-3 fatty acid intake. We need more population studies of this genotype.

While genetic analysis was anticipated in the original study design, a limitation of this study is that this effect-modification analysis was not among the trial’s pre-specified aims, although the FADS Indel itself was a single mechanistically motivated measurement, genotyped blind to outcome. Specific examination of the FADS Indel was not planned. However, the study tests a specific hypothesis grounded in the known relationship between the genotype at a particular locus and an underlying biochemical change, arachidonic acid levels. We measured no other genetic polymorphisms in this cohort, minimizing the risk of spurious results from multiple comparisons. The results indicate that FADS Indel genotype or a validated proxy be characterized in future studies of omega-3 supplementation, with either EPA or DHA. A further limitation is the small number of outcome events: among the 761 participants there were 74 preterm births and 17 early preterm births, and after stratification by FADS Indel genotype and DHA dose several subgroups contained few cases (as few as 1–3 early preterm birth events per genotype × dose cell). Estimates for these sparse sub-groups, particularly for early preterm birth and for the least common genotype (I/I), carry wide credible intervals and are sensitive to small perturbations, as shown in the prior-sensitivity and outcome-tilted analyses (Tables C and N in S1 Appendix). The strength of evidence also differed between outcomes: the early preterm birth association in the I-allele group was robust to worst-case outcome tilting, whereas the preterm-birth association, although directionally consistent throughout, was more fragile, falling below a posterior probability of 0.90 with only a small adverse tilt (Γ = 1.1) compared with Γ = 1.40 for early preterm birth (Table N in S1 Appendix). The early preterm birth effect therefore carries stronger evidentiary weight, and the preterm birth finding was less robust and warrants attention in replications in larger, prospectively genotyped cohorts.

We assessed potential confounding across genotype groups (Table O in S1 Appendix). The three genotypes were well balanced on the major risks that could lead to preterm birth: prior preterm birth (D/D 13.7% (95% CI [10.5, 17.8]), I/D 13.4% (95% CI [10.1, 17.6]), I/I 14.2% (95% CI [8.8, 22.0]); p = 0.98), prior early preterm birth (D/D 5.3% (95% CI [3.4, 8.2]), I/D 6.1% (95% CI [3.9, 9.3]), I/I 7.5% (95% CI [3.9, 14.2]); *p* = 0.68), maternal age (D/D 30.7 years (95% CI [30.1, 31.3]), I/D 30.1 (95% CI [29.5, 30.7]), I/I 29.6 (95% CI [28.5, 30.7]); *p* = 0.15), and baseline DHA status (D/D 6.3% (95% CI [6.1, 6.5]), I/D 6.3% (95% CI [6.1, 6.5]), I/I 6.4% (95% CI [6.1, 6.7]); *p* = 0.85) and randomized DHA dose (1,000 mg/d: D/D 55.8% (95% CI [50.5, 61.0]), I/D 48.9% (95% CI [43.4, 54.4]), I/I 49.1% (95% CI [39.7, 58.4]); *p* = 0.16). To test whether socioeconomic differences (household income, education, and marital status) explained the genotype–preterm birth association, we compared preterm birth by genotype within each factor; the higher preterm birth rate in the I-allele group relative to D/D persisted at every level of income, education, marital status, smoking, and pre-pregnancy BMI (Table P in S1 Appendix). Thus, the elevated risk in the I-allele group cannot be fully explained by these socioeconomic differences. This was most consistent for I/D, which retained adequate subgroup sizes; the I/I subgroups showed the same direction but were limited by small numbers once stratified. Because the primary comparison on DHA dose within a genotype was randomized, it is not subject to this confounding. This post-hoc analysis of the ADORE trial based on a single, mechanistically pre-specified variant genotyped blind to outcome identifies the FADS Indel as a predictive biomarker for the effect of DHA dose on preterm and early preterm birth. Preterm and early preterm birth rates were lower with high-dose DHA (1,000 mg/d) in the I-allele group; in the D/D group, the same dose was associated with prolonged gestation and lower preterm birth rates. Matching dose to genotype—high dose for the I-allele group, low dose for D/D—was associated with the highest proportion of term births. The genotype × dose interaction was credible for EPB and prolonged gestation (PP = 0.99 for EPB and PP = 0.98 for prolonged gestation) but not for PB, which benefitted all genotypes by declining similarly with high-dose DHA (PP = 0.56). The finding is grounded in a defined *cis-*regulatory mechanism by which greater ARA synthesis in the I-allele group provides more substrate for the parturifacient prostaglandin, PGE2, and it converges with the same Indel being associated with EPA response in colorectal polyp prevention [20]. As a single-trial interaction, it warrants replication in independent omega-3 supplementation trials.

## Supporting information

S1 Fig MCMC trace plots (four chains) for representative binomial models. Trace plots are shown for: **(a)** early preterm birth among Black participants with the D/D genotype in the dose-contrast model, representing a sparse stratum with zero events in both treatment arms; **(b)** preterm birth among all participants with the I/D genotype in the dose-contrast model, representing a better-powered comparison; and genotype-contrast models comparing the I-allele and D/D groups in the 200 mg arm for **(c)** preterm birth and **(d)** early preterm birth. Across all panels, the four chains overlap closely with no visible trend or drift, supporting convergence. Mixing was also acceptable in the sparse early-preterm-birth model, indicating stable sampling even under low-event conditions. Parameters are shown on the log-odds scale.
(TIF)

S2 Fig Posterior predictive checks for genotype contrasts. Posterior predictive checks are shown for genotype-specific event rates in the D/D and I-allele groups in the 200 mg/d and 1,000 mg/d DHA arms: **(a)** preterm birth (<37 weeks), 200 mg/d; **(b)** preterm birth (<37 weeks), 1,000 mg/d; **(c)** early preterm birth (<34 weeks), 200 mg/d; and **(d)** early preterm birth (<34 weeks), 1,000 mg/d. Histograms show the distribution of event rates across datasets simulated from the fitted Bayesian models, and vertical dark blue lines indicate the observed event rates. Across outcomes, treatment arms, and genotype groups, the observed event rates fall within the posterior predictive distributions, with no evidence of misfit. The discrete spikes in the early-preterm-birth panels reflect the small number of possible event counts at those low rates.
(TIF)

S3 Fig Prior versus posterior for the I/I subgroup. For each I/I stratum (preterm birth and early preterm birth at 200 and 1,000 mg/d), the diffuse Normal(0, 5^2^) prior (amber) is overlaid with the posterior (blue) on the probability scale.
(TIF)

S4 Fig Preterm birth (PB) posterior probability distributions among all participants. Stratification by **(A)** D/D, **(B)** I/D, and **(C)** I/I genotype. Posterior probability (PP) >0.50 indicates that 1,000 mg/d DHA is more effective at reducing PB compared to the 200 mg dose.
(TIF)

S5 Fig Early preterm birth (EPB) posterior probability distributions among all participants. Stratification by **(A)** D/D, **(B)** I/D, and **(C)** I/I genotype. Posterior probability (PP) >0.50 indicates that 1,000 mg/d DHA is more effective at reducing EPB compared to the 200 mg dose. PP = 0.07 indicates that for the D/D genotype there is a PP = 0.93 that 1,000 mg DHA increased the risk of EPB compared to 200 mg.
(TIF)

S6 Fig Preterm birth (PB) posterior probability distributions among Black participants. Stratification by **(A)** D/D, **(B)** I/D, and **(C)** I/I genotype. Posterior probability (PP) >0.50 indicates that 1,000 mg/d DHA is more effective at reducing PB compared to the 200 mg dose.
(TIF)

S7 Fig Early preterm birth (EPB) posterior probability distributions among Black participants. Stratification by **(A)** D/D, **(B)** I/D, and **(C)** I/I genotype. Posterior probability (PP) >0.50 indicates that 1,000 mg/d DHA is more effective at reducing EPB compared to the 200 mg dose.
(TIF)

S8 Fig Prolonged gestation (≥40 weeks) by dose within genotype. In the D/D group, participants receiving high-dose (1,000 mg/d) DHA had more prolonged gestations (≥40 weeks) than those receiving low-dose (200 mg/d) DHA (posterior mean 25.7% (95% CrI [19.7%, 32.0%]) for high-dose vs. 15.3% (95% CrI [10.0%, 21.4%]) for low-dose; Bayesian PP = 0.99). In the I-allele group, no meaningful difference in prolonged gestation rates was observed between doses (16.1% (95% CrI [11.5%, 21.4%]) for high-dose vs. 17.8% (95% CrI [13.0%, 23.2%]) for low-dose; PP = 0.33).
(TIF)

S9 Fig Prolonged gestation (≥40 weeks) by genotype within dose. On high-dose (1,000 mg/d) DHA, prolonged gestation was more frequent in the D/D group than in the I-allele group (posterior mean 25.7% (95% CrI [19.8%, 32.0%]) for D/D vs. 16.1% (95% CrI [11.5%, 21.5%]) for I/D + I/I; PP = 0.99). On low-dose (200 mg/d) DHA, no meaningful difference was observed between genotypes (15.3% (95% CrI [10.1%, 21.4%]) for D/D vs. 17.8% (95% CrI [13.3%, 23.1%]) for I/D + I/I; PP = 0.26).
(TIF)

S10 Fig Posterior distributions of prolonged gestation (delivery at ≥40 weeks) by FADS Indel genotype and DHA dose. Overlaid histograms show the posterior prolonged-birth rate for the two groups in each panel. **(A)** D/D vs. the I-allele group, 1,000 mg/d (49/191, 25.7% (95% CrI [19.8%, 32.0%]) vs. 33/205, 16.1% (95% CrI [11.5%, 21.5%]); PP = 0.99); **(B)** D/D vs. the I-allele group, 200 mg/d (23/151, 15.3% (95% CrI [10.1%, 21.4%]) vs. 38/214, 17.8% (95% CrI [13.3%, 23.1%]); PP = 0.26); **(C)** D/D, 1,000 vs. 200 mg/d (49/191, 25.7% (95% CrI [19.7%, 32.0%]) vs. 23/151, 15.3% (95% CrI [10.0%, 21.4%]); PP = 0.99); **(D)** the I-allele group, 1,000 vs. 200 mg/d (33/205, 16.1% (95% CrI [11.5%, 21.4%]) vs. 38/214, 17.8% (95% CrI [13.0%, 23.2%]); PP = 0.32). PP is the posterior probability that the first-named group has the higher prolonged-birth rate.
(TIF)

S11 Fig Distribution of FADS Indel genotypes by site. Genotype distribution at **(A)** Kansas City (*n* = 389), **(B)** Columbus, Ohio (*n* = 189), and **(C)** Cincinnati, Ohio (*n* = 183), USA.
(TIF)

S1 Appendix Supplementary tables and sensitivity analyses. This appendix contains Tables A–P, including Bayesian diagnostics, prior-sensitivity analyses, hierarchical model sensitivity analyses, IPW sensitivity analyses, genotype-selection and outcome-tilted sensitivity analyses, baseline characteristics, fatty acid composition, and socioeconomic analyses.
(DOCX)

S1 Protocol ADORE Trial Study Protocol. (PDF)

S1 Checklist STROBE Checklist. STROBE checklist (https://www.strobe-statement.org/; https://doi.org/10.1371/journal.pmed.0040296).
(DOCX)

S2 Checklist Consort Checklist. Hopewell S, Chan AW, Collins GS, Hróbjartsson A, Moher D, Schulz KF, et al. CONSORT 2025 Statement: updated guideline for reporting randomised trials. BMJ. 2025; 388:e081123. https://dx.doi.org/10.1136/bmj-2024-081123. 2025 Hopewell et al. This is an Open Access article distributed under the terms of the Creative Commons Attribution License https://creativecommons.org/licenses/by/4.0/), which permits unrestricted use, distribution, and reproduction in any medium, provided the original work is properly cited. *We strongly recommend reading this statement in conjunction with the CONSORT 2025 Explanation and Elaboration and/or the CONSORT 2025 Expanded Checklist for important clarifications on all the items. We also recommend reading relevant CONSORT extensions. See https://www.consort-spirit.org.
(PDF)

S1 File Statistical analysis plan for the ADORE trial. (PDF)

S1 Code Data Analysis Code. (DOCX)

S1 Data De-identified participant-level analytic dataset. (XLSX)

## Abbreviations

ACOG: American College of Obstetricians and Gynecologists
ADORE: Assessment of DHA on Reducing Early Preterm Birth
ARA: arachidonic acid
CI: confidence interval
CrI: Bayesian credible interval
DGLA: di-homo-gamma-linolenic acid
DHA: docosahexaenoic acid
EPA: eicosapentaenoic acid
EPB: early preterm birth
FADS: fatty acid desaturase
IPW: inverse probability weights
PB: preterm birth
PGE2: prostaglandin E2
PP: Posterior Probability
RR: risk ratio
