# Supplementary material for: Genotype-informed docosahexaenoic acid dosing based on the FADS Indel to reduce preterm and prolonged gestation: A secondary analysis of the ADORE randomized trial
Source: PLoS Med. 2026 Oct 8;23(10):e1005274. doi: 10.1371/journal.pmed.1005274 (PMC13660990; doi:10.1371/journal.pmed.1005274)
Supplement: Supplementary file 1 [file pmed.1005274.s013.pdf]

INTRODUCTION

We appreciate reviewer comments and have made changes to strengthen the proposal. We first address issues raised in the Resume and Summary of Discussion then other comments in the separate reviewers' comments. Additions to the revised proposal are denoted by a vertical line on the left side of the page.

Resume and Summary of Discussion. Two minor concerns were raised. First, there was concern about beginning the adaptive design after only 150 subjects were enrolled. We clarify that the adaptive design begins when 150 women in each group are enrolled. Anticipating enrollment of ~25 subjects/month with birth ~6 mo after enrollment, we should have ~150 births (known primary outcome) by the time 300 subjects are enrolled. Thereafter, the adaptive design would be implemented after every additional 150 births (~ 6 month intervals).

A second concern was the limited scope of primary and secondary outcomes proposed. We have added secondary pregnancy outcomes (gestational diabetes, preeclampsia, C-section, spontaneous labor) that were evaluated in KUDOS and not influenced by DHA. However, that study was underpowered to detect an effect on these important outcomes by intent-to-treat or after controlling for potential predictor variables, including a number of potential predictor variables suggested by reviewers.

Additional Comments and Responses. We added details of recruitment and the 3 medical center clinics where subjects will be recruited (C.1.a) as well as the potential limitations of the Bayesian approach (B.2) ***(reviewer #1).*** We moved the statement that we would bank blood for potential future analysis from Innovation but left a statement about banked samples in C.2 ***(reviewer #1)*** and new planned analysis (sRAGE).
Specifically, we will examine a possible mechanism of action of DHA ***(reviewer #1)*** by measuring sRAGE, a biomarker associated with a variety of inflammatory conditions and influenced by DHA in a murine model of inflammation as a new specific aim (#3***).*** sRAGE has advantages over other biomarkers of inflammation in that samples are stable while samples are on ice and during storage at -80º C.

Reviewer #1 stated that it would have been helpful had we indicated the expected rate of prior PTB and progesterone injection in the proposed trial. This is added. The comment was linked to an absence of effect of DHA on ePTB in an earlier NICHD Network trial. We suggest that the NICHD trial favored inclusion of women with a higher risk for anatomical ePTB, whereas the positive effects to reduce ePTB in populations with a low rate of prior PTB such as our previous Phase III (KUDOS) trial and the Australian DOMInO trial are more plausibly linked to reduced inflammation.

All 3 reviewers commented on at least one variable that we plan to use as potential predictor variables in our efficacy analyses of primary and secondary outcomes including maternal nutrient intake ***(reviewers #1 and #2),*** smoking ***(reviewer #1)*** and fetal sex ***(reviewer #3)*** (see SA #2). In addition to individual nutrients that are plausibly related to PTB, we will use principal component analysis to determine patterns of nutrient intake and test these as potential predictor variables.

While smoking is associated with preterm birth, we did not find any suggestion in KUDOS that the effects of DHA to reduce ePTB were restricted to smokers, however, with the larger study we have better power to test a number of potential predictor variables including maternal smoking history ***(reviewer #1)***.

Subjects are instructed how and when to do their 3-d diet record and provided visuals of servings size. When the records are returned, they are reviewed and issues resolved by a phone call if necessary. The process ensures the most reliable reporting regardless of literacy and/or writing and reading skills ***(reviewer #1***).

We will evaluate the effectiveness of randomization according to CONSORT guidelines (C.6.a) ***(reviewer #1***).

There was concern that the dose was not consistent ***(reviewer #1*).** The comparison of 1000 mg vs 600 mg is the proposed high dose compared to KUDOS. The letter from DSM Pharmaceuticals promising capsule donation should have said 400 mg DHA/capsule and this is corrected.

We obtain maternal blood at enrollment and at time of birth (7 days was a typographical error) ***(reviewer #3).***

SPECIFIC AIMS

Preterm birth contributes to 0.5 million deliveries in the United States (1 of 8 pregnancies) and poses a huge burden on public health with costs in the billions. Of particular concern is that the rate of earliest preterm birth (<34 weeks) (ePTB) in the US has decreased little since 1990 and these births impact overall infant mortality the most, resulting in the greatest cost to society. Docosahexaenoic acid (DHA) supplementation provides a high yield, low risk provocative strategy to reduce early preterm delivery in the US by up to 75%.

We propose a Phase III Clinical Trial (randomized to low or high dose DHA, double-blind) to examine the efficacy of high dose DHA supplementation to reduce ePTB and confirm the safety of supplementing pregnant women for the last two trimesters of pregnancy with high dose DHA (1000 mg/d) compared to 200 mg/day, the amount recommended by the FAO/WHO for pregnant and lactating women. Prenatal supplements typically provide 200 mg DHA, however, there is no evidence to suggest that 200 mg DHA is effective to reduce ePTB. We propose to address a knowledge gap between what is recommended and the evidence that DHA can reduce ePTB by comparing 200 mg DHA/d to 1000 mg DHA/d, which may be an appropriate dose in the US where fish intake is low. The proposal is modeled on a previous Phase III trial by co-PI Carlson in which pregnant US women were supplemented with 600 mg DHA/d compared to a placebo (KUDOS, R01 HD047315, ClinicalTrials.gov: NCT00266825). In that trial, DHA supplementation reduced ePTB by 85%. A placebo-controlled trial conducted in Australia with 800 mg DHA/d (DOMInO trial) found a 50% reduction in ePTB. However, in both trials, this was a secondary outcome observed in a low risk pregnancy cohort. The primary aim of this proposed study is to test the hypothesis that ePTB is reduced by 1000 mg of DHA per day compared to 200 mg of DHA. The results could be used immediately to inform clinical practice on prenatal supplementation.

The major specific aims of this project are:

(1) **To determine if a supplement of 1000 mg DHA /day compared to 200 mg DHA/day during the last two trimesters of pregnancy can reduce ePTB (*primary efficacy analysis*).** Pregnant women will be randomly assigned to 2 daily capsules of algal oil (totaling 800 mg DHA) or soybean and corn oil (0 mg DHA) beginning between 12 and 20 wk gestation. Both groups will receive a commercially-available, prenatal supplement containing 200 mg DHA. Therefore, the experimental group will be given 1000 mg DHA/d and the control group 200 mg DHA/d. We will employ a novel Bayesian response adaptive randomization design that assigns more subjects to the "winning" group. Using adaptive randomization (being able to change how we assign subjects to the groups during the study based on information gained during the study) allows for substantially smaller sample sizes and provides better conclusions about the most effective treatment because it lets us change our approach or stop the study early if we find strong results before the scheduled end of the study. Using this randomization design and data from our previous trial, the study has 87% power to detect a reduction in ePTB from 4% to 1% of births with an estimated 927 subjects, trial duration of 180 wk, and 60% of the subjects in the winning group. A conventional equal randomization trial would have 87% power, but be larger (1100 subjects enrolled), slower (208 wk), and have a lower rate of subjects on the winning group (50%). The analysis will be by intent-to-treat. The design has the potential to significantly expedite the findings to the research community and clinical practice.

(2) **To conduct a pregnancy efficacy analysis controlled for potential predictor variables.** We will use prior DHA status, overall DHA intake (dietary and supplement intake), other dietary nutrients, smoking, infant gender, income, and demographic variables to determine if there are pregnancies that benefit from DHA supplementation with reduced ePTB.

(3) **To determine if DHA intake affects plasma soluble (s) RAGE concentration.** sRAGE is a competitive inhibitor of RAGE and is increased in some inflammatory states (chorioamnionitis, LPS-administration). DHA attenuates the increase in sRAGE concentration in LPS-induced inflammation in murine models. We propose to determine if the higher DHA supplementation influences either maternal or cord blood sRAGE as a possible mechanism.

(4) **To evaluate the occurrence of adverse events in women and infants in the experimental (1000 mg DHA) and control (200 mg DHA) groups**. Phase III Clinical Trials must evaluate adverse events to address whether the intervention could be used safely if it proves to be efficacious.

RESEARCH STRATEGY

A. SIGNIFICANCE

A.1. DHA intake and status of US women during pregnancy and physiological importance. DHA is a long-chain polyunsaturated fatty acid member of the n-3 (or omega-3) fatty acid family. DHA is found in animal foods with the richest sources being varieties of ocean fish (1). On average, US women consume ~60 mg DHA/day (2) and synthesize little DHA from α–linolenic acid (18:3n-3) they consume in other foods (3, 4). DHA intake among US women is lower than other Western populations (2). Two commonly used indicators of DHA status: 1) red blood cell phospholipid (RBC-PL) DHA as a percent of total membrane fatty acids (5, 6) and 2) human milk DHA as a percent of total fatty acids (7) are lower in US women than in other developed countries. For example, baseline RBC-PL-DHA means ranged from 4.3-5.0 % in our last 3 Kansas City pregnant cohorts (5, 8, 9) compared to greater than 6% RBC-PL-DHA reported by others (10-12). The US rate of PTB (<37 wk) is also higher than other developed countries (13). The NICHD Maternal Fetal Network multi-center trial finds the highest rate of PTB for women in the lowest quartile for RBC PL DHA (OR 1.45) (14). Worldwide, 24% of all preterm births occur in India, where vegetable-based diets low in DHA are common (13).

US studies typically report a mean of 0.15-0.2% DHA in human milk (7, 15, 16) in contrast to countries where ocean fish are routinely consumed (0.5 to 2.7%) (17, 18). Our pilot feasibility trial found extremely low milk DHA in women consuming a placebo, but milk DHA increased to 0.5-0.7% in the group assigned to receive a dietary supplement of 1000 mg of DHA/day (16) (**Figure 1**). Women consuming more DHA as part of their usual diet provide more DHA to their fetus during pregnancy and have higher milk DHA during lactation. It is well established that the biosynthesis of DHA from α–linolenic acid is very limited (3, 4), especially under several conditions such as caloric deprivation, protein inadequacy, and corticosteroids, which inhibit the δ6–desaturase and, therefore, DHA synthesis (19) **(Figure 2)**. In fact, there have been many attempts to increase DHA status and milk DHA by feeding α–linolenic acid without success.

Preformed dietary DHA found in marine life, algal sources, or eggs can produce immediate biologic effects. Dietary DHA also increases DHA in membranes of cells from all organs that have been studied. Higher DHA status is linked to a number of positive health outcomes including protection against cardiovascular disease (20, 21), breast cancer (22-24), and Alzheimer's disease (25-28) and more recently to resolution of inflammation (29-31) and neuroprotection (32, 33). Higher DHA status during development is linked to higher cognitive performance (34-37), lower allergy (38-41), and lower adiposity (42). <u>*To summarize, DHA is found in some foods but little is synthesized from α–linolenic acid. DHA intake and status of US women are among the lowest in the world*</u>*.*

Figure 1.

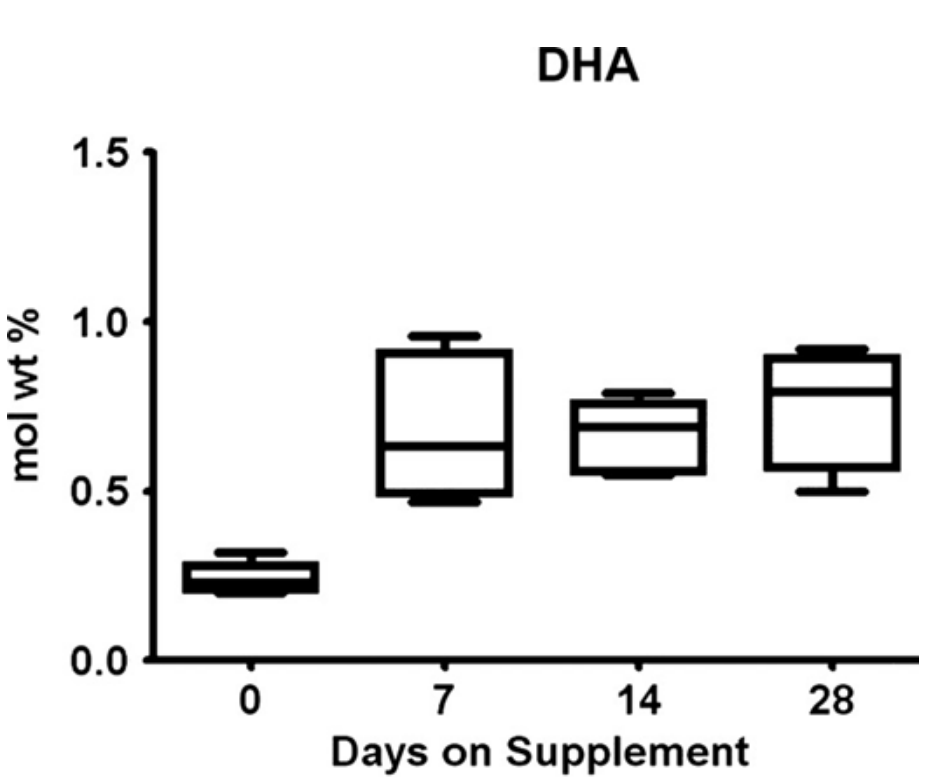

Figure 2.

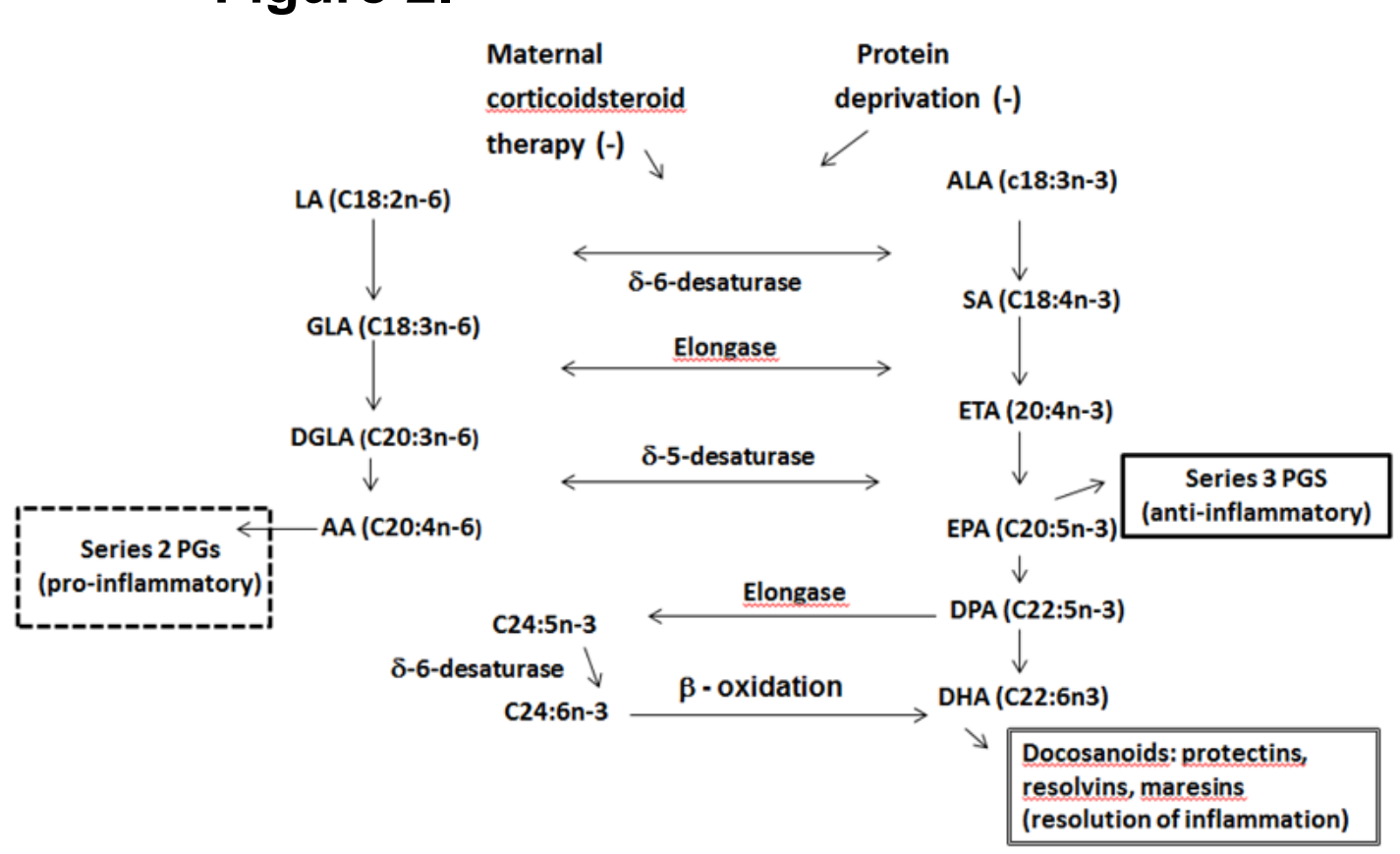

A.2. Effects of DHA and EPA supplementation on gestation duration, preterm birth, ePTB and VLBW. DHA status in pregnancy was first linked to longer gestation, higher birth weight, and less PTB by early studies of Olsen and collaborators (43, 44) after they observed longer gestation among the Faroe Islanders who consumed higher DHA and EPA compared to the Danes (43). Three recent systematic reviews address the question of pregnancy outcomes in randomized trials of total omega-3 LCPUFA supplementation (45-47). A 2006 Cochrane review that included 6 trials with 2755 women (45) found a 2.6 d increase in gestation duration favoring supplementation (P=0.0009). Only one review found a significant reduction in overall PTB (<37 wk) (P values=0.03-0.29 ((45-47), but all reported a reduction in ePTB with odds ratios favoring supplementation of 0.42 to 0.74. We note, however, that the ePTB findings are based on only 3 studies that provided DHA in amounts of either 800 or 2000 mg/d. Two trials conducted in Australia and Europe both found a reduction in ePTB (48, 49), however, the trial conducted in the US by the NICHD Maternal Fetal Network trial did not find a decrease in ePTB despite supplementation with 800 mg DHA/d. The absence of an effect in this trial could be linked to the fact that the trial included only women with a prior preterm birth and most likely a disproportionate number of women at anatomical risk of ePTB (all received weekly injections of progesterone) (50). In contrast, only 4.9% of women in KUDOS had a prior PTB and only 2.3% (n=7) a prior ePTB (one had a repeat ePTB). None received progesterone for short cervix. The potential of DHA to reduce ePTB is more plausibly linked to its role in reducing inflammation, a more common cause of ePTB. After the systematic reviews were published, our KUDOS trial conducted in the US and published later finds significantly less ePTB (4.8 vs 0.6%, P=0.025), fewer VLBW births (3.4 vs 0%, P=0.026) and fewer days in hospital for infants born preterm (40.8 vs 8.9 day, P=0.026) in the group supplemented with 600 mg/d DHA compared to placebo (5). *We conclude from studies conducted around the world that a high dose of DHA supplementation can reduce ePTB. No study has evaluated high dose DHA supplementation as a primary outcome to reduce ePTB, which we propose to do in a population of US women, who are known to have poor DHA status before and during pregnancy.*

A.3. Importance of reducing ePTB and what the evidence suggests may be happening with high dose DHA supplementation. Overall ePTB rates in the US for 2012 were 3.4% (51), however, the ePTB rate for non-Hispanic blacks (7.0%) is higher than for non-Hispanic whites (3.3%). ePTB compared to late PTB (≥34 wk-37 wk) carries a much greater risk of infant morbidity and mortality and costs society many more in billions of dollars. Three systematic reviews (45-47), one conducted after publication of our KUDOS trial (5) find a significant reduction in ePTB with high dose DHA supplementation. KUDOS results suggest that DHA prolongs gestation of a subset of pregnancies that would have ended with ePTB. The ratio of ePTB to overall PTB in our placebo group was 0.545 compared to 0.077 in the DHA group. We have other evidence that DHA shifts the gestational age to the right. From 336,129 single births occurring in North Carolina between 2004 and 2007 three normally distributed classes of gestational age are identified with means of 39.2, 38.2 and 33.3 wk (classes 1,2 and 3, respectively) (52). In both DOMInO and KUDOS the percentage of births in class 3 (mean 33.3 wk gestation) is significantly reduced by DHA supplementation (KUDOS shown **Figure 3**). For KUDOS, 7.1% of births in the placebo group fell into class 3 compared to 2.7% of births in the DHA supplemented group (P=0.0187). For DOMInO, 3.8% of placebo births fell in class 3 vs. 2.2% of births in the DHA/EPA-supplemented group (P=0.021). Biomarkers assessed prior to 20 wk gestation suggest that the risk of spontaneous preterm labor has an early etiology from stressors linked to inflammation particularly PTB due to prolonged rupture of membranes and chorioamnionitis (53-56). We hypothesize that DHA will prolong gestation duration for a period of time that may be sufficient for the fetus to require less medical intervention; i.e., that DHA will reduce ePTB. *Reducing ePTB would be clinically important as it is well known that ePTB compared to late PTB carries a far higher risk of neonatal morbidity and stress for families and a large societal burden for cost of neonatal intensive care, repeated re-hospitalization post-discharge and long term medical care.*

Figure 3.

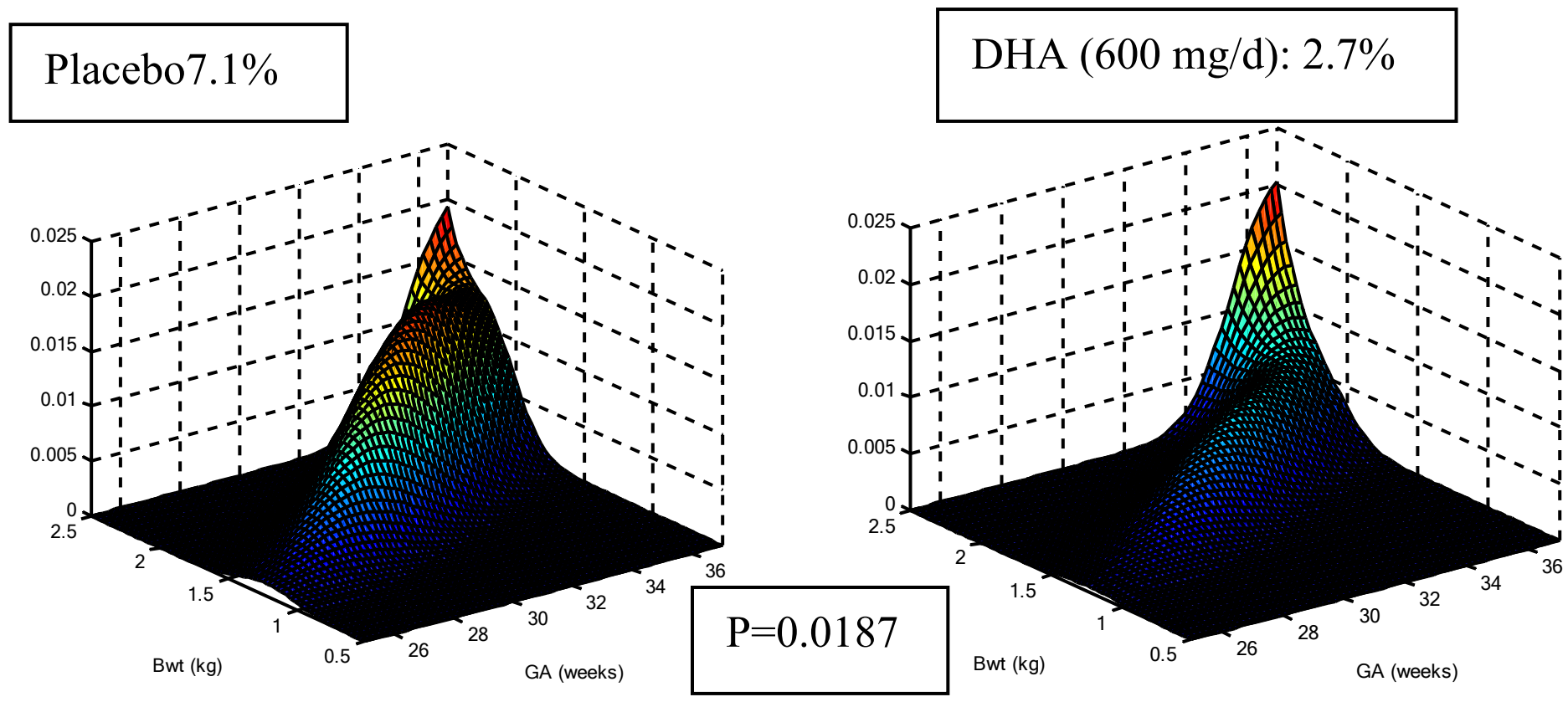

A.4. Mechanisms by which DHA might reduce ePTB. DHA unlike other omega-3 fatty acids (α–linolenic acid and EPA) uniquely modulates cell surface ligands to attenuate inflammation, demonstrated in both humans and animal models (57-61). While the mechanism of preterm parturition remains elusive and complex (62), DHA has plausible cellular effects that could modify the onset or change the timing of inflammation by antagonizing the NFkB pathway (63, 64) and promoting the activation of SIRT1 expression thereby directly impacting endothelial relaxation (65), or altering membrane fluidity (65, 66) and cell signaling (67, 68). It is now recognized that DHA released from phospholipids in cell membranes serves as a precursor for docosanoids (22 carbon mediators such as resolvins, protectins, and maresins) that are anti-inflammatory, resolve inflammation, and protect against inflammation (31, 69-73) (**Figure 2**). While it is generally believed that fish oil (a source of both DHA and EPA) increases gestation duration because EPA competes with arachidonic acid (ARA), the source of the 2-series prostaglandins E_2 and $F_{2\alpha}$ required for labor and delivery (74, 75), we provided only DHA in KUDOS. Moreover, as indicated above, the effect of DHA supplementation in both KUDOS and DOMInO is only significant in the class of births that delivers early; i.e., we find no evidence that DHA increases gestation other than in a select group of pregnancies.

A recent pilot study found lower preterm PROM in frequent fish eaters randomly supplemented with only 100 mg DHA/d (76) further linking DHA intake to reduced inflammation. We speculate that pregnancies at risk for ePTB include a disproportionate number with inflammation. sRAGE is positively linked to chorioamnionitis and preterm labor (59) and LPS-induced inflammation in a murine model (77)]. In the latter example, the increase in sRAGE was attenuated by DHA supplementation. sRAGE is negatively associated with IL-6, sepsis and FIO2 requirements in PT cohorts (54, 59, 78), however, sRAGE has not been compared in term and preterm pregnancy. We propose to determine if the higher DHA supplementation influences either maternal or cord blood sRAGE as a possible mechanism of DHA.

A.5. DHA supplement - rationale for doses. The Institute of Medicine does not set a DRI for DHA in pregnancy, however, the FAO/WHO and expert groups suggest an intake of 200 mg of DHA per day for pregnancy and lactation (79-81). Many US prenatal vitamins now contain 200 mg of DHA, and 15% of women in KUDOS took a DHA supplement. We propose to compare this low recommended dose of DHA (control group) to a high dose DHA supplement (experimental group). We chose 1000 mg as a high dose because doses up to 2000 mg/d of DHA have reduced ePTB in populations that consume more DHA than in the US (47-49). More DHA may be optimal in the US where intake and status at baseline are low. Trials providing less than 600 mg DHA have not found a reduction in ePTB (82, 83). DHA is a nutrient and intake and status are inherently variable. Women with low DHA status may require more DHA to reach an intake to reduce ePTB. All nutrients have an intake that is optimal for a given outcome and the ability to improve that outcome with supplementation is based on an individual's status at baseline **(Figure 4).** Overall KUDOS subjects had a very low DHA status (mean RBC-PL-DHA = 4.3%) consistent with DHA deficiency (84). We find 3 clusters in the DHA supplemented group of KUDOS **(Figure 5)**. Cluster 1 includes the majority of the DHA group and is exceedingly deficient with a mean baseline RBC-PL-DHA = 3.5%.

Figure 4.

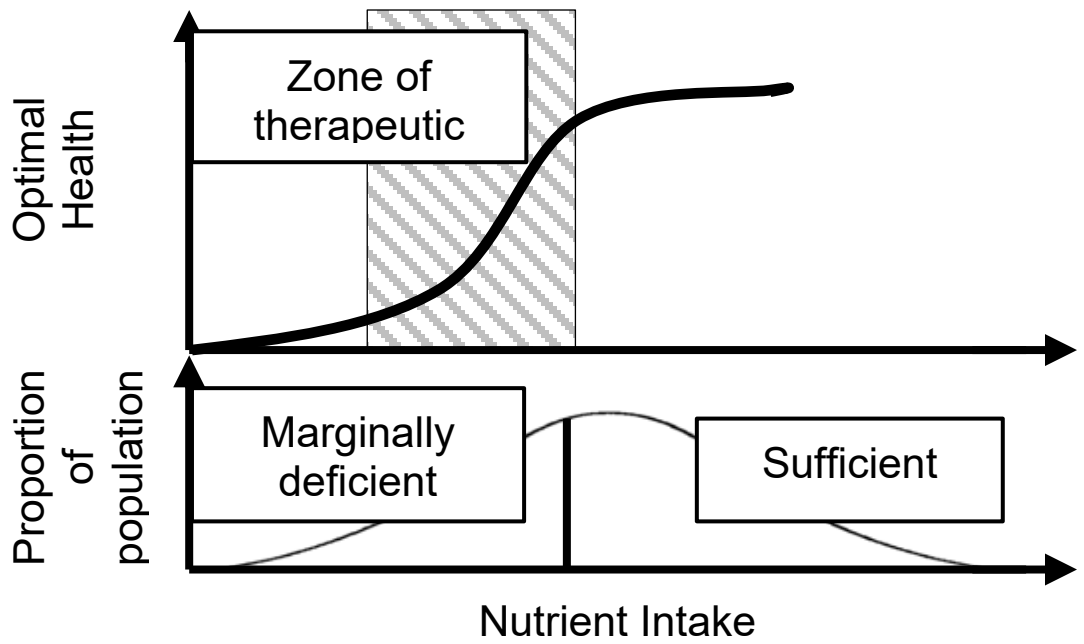

Figure 5.

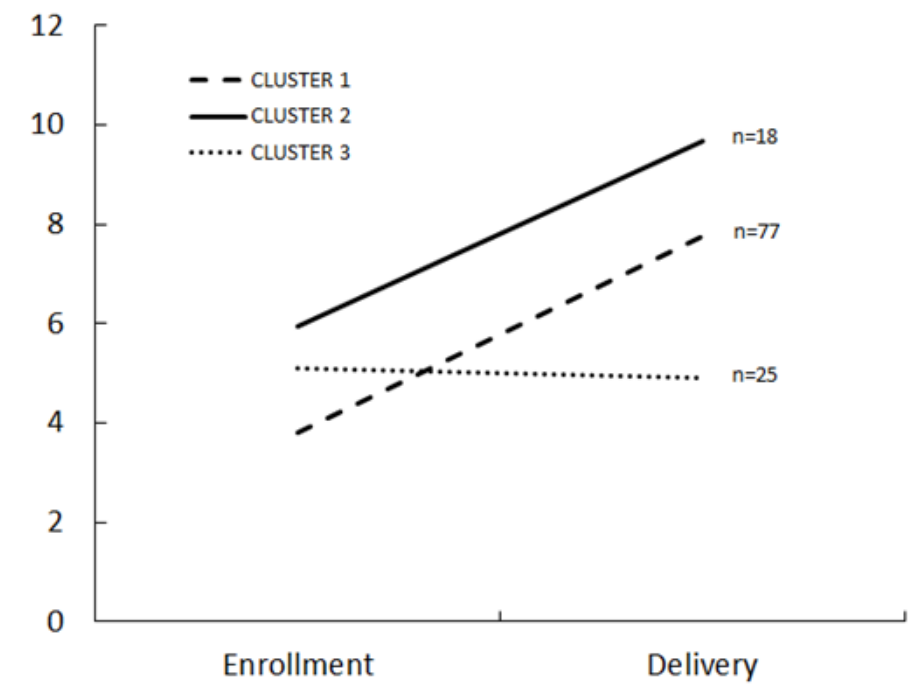

B. INNOVATION

B.1. Nutritional Approach to Reduce ePTB and VLBW. There is currently no accepted method for predicting pregnancies that will end in ePTB and no known treatment to prevent the occurrence of ePTB with the exception of progesterone therapy for women with cervical lengths of ≤20 mm (85, 86). In contrast, the US KUDOS and the larger DOMInO trial conducted in Australia excluded only pregnant women with serious maternal illness and found a significant reduction in early preterm birth with 600 mg/d DHA (n=301, 0.6% vs. 4.8% of births) and 800 mg/d DHA (n=2399, 1.1% vs 2.2% of births), respectively. As mentioned above (A.4), the presumed effect of DHA is reduction/ resolution of inflammation, a more common cause of ePTB than short cervix. Even in the NICHD trial, women with the lowest DHA blood concentrations were more likely to have a recurrent PTB (87). In both KUDOS and DOMInO, the incidence of early preterm births in the placebo group was similar to the incidence within the country. These trials and two systematic reviews suggest that a high dose supplement of DHA could effectively reduce ePTB. We feel such an approach could be particularly useful in pregnant US women whose DHA status is among the lowest of any population in the world. It is well known that 500-1000 mg DHA/d in adults improves clinical biomarkers of inflammation, systemic vascular changes, and endothelial cell function while low DHA intake shifts immune homeostasis towards a more pro-inflammatory response. We propose to compare 1000 mg DHA to 200 mg DHA/d. The lower amount of DHA is found in many currently available prenatal supplements, however, the studies that have found an effect of DHA on early preterm birth have provided between 600 and 900 mg/d (5, 48, 49).

B.2. Novel randomization approach. The Bayesian adaptive randomization design allows the trial to be stopped when a probability of 0.99 is reached that the groups are different. We employ a more conservative prediction for reduction in early preterm birth than we observed in our earlier trial (from 4% to 1%) and estimate that the study could be stopped a year earlier than planned and the results put into practice immediately. Another advantage is that more women would be assigned to the winning strategy during the trial. Security, logistics, and work required to build and evaluate designs are potential pitfalls for Bayesian response adaptive randomization. This can be minimized by limiting interim analysis results given to site PIs. The latter two are dealt with by proper budgeting for statistical labor and the core support from the institution (88) (We have properly budgeted limited for statistical support and have had institutional support from the University of Kansas Medical Center Biostatistics core for time to design this study (see C.2.a and C.3.).

C. APPROACH

C.1. Overview. *The primary purpose of this study is to determine if DHA supplements totaling 1000 mg/d compared to 200 mg/d during the last two trimesters of pregnancy can reduce ePTB (Specific Aim 1) and to conduct a secondary pregnancy efficacy analysis to determine if there is a subset of pregnancies most likely to*

benefit from DHA supplementation (Specific Aim 2). To obtain information about the effects of DHA on inflammation, we will measure sRAGE, which is plausibly influenced by DHA supplementation based on a murine model of LPS-induced inflammation (Specific Aim 3). We will perform a prospective randomized comparative effectiveness adaptive design study with pregnant women to enhance efficiency of the trial and expedite results to the research community and to guide clinical practice. First analysis will be by intent-to-treat. Safety data will be collected as required for Phase III RCTs (Specific Aim 4). We will bank maternal and cord blood to be available for future evaluations.

C.1.a. Subject recruitment. Women who are 18 years of age and older and are in their 12th to 20th week of gestation will be eligible for enrollment. In addition to outpatient clinics associated with the 3 participating centers (University of Cincinnati, The Ohio State University and the University of Kansas Medical Center), recruitment sites will be added as necessary. Sites may include but are not limited to other area or reginal hospital systems, free standing clinics and health departments. Additionally we may display approved advertisements in community settings, on social media outlets and by word of mouth. In January 2019, the Johnson County Health Department in Olathe, KS was added as an enrollment site with the University of Kansas Medical Center staff conducting all study-associated research activities. *The estimated date of delivery (EDD) determined by the 2014 ACOG guidelines will be fixed as the EDD for assessment of gestation duration in the study.* Women must be able to read or orally understand the study in English or Spanish and sign an informed consent form. The consent form will be in both English and Spanish and translators will be available to obtain informed consent for potential subjects who are not fluent in English. We will also have fluent Spanish speakers available to call these subjects. They must agree at enrollment to consume the capsules assigned them from then until they are delivered.

The goal is equal enrollment at the 3 centers with up to 400 subjects enrolled at each center (depending upon the dictates of the Bayesian response adaptive randomization). We expect to enroll 2 subjects/week at each of the 3 centers (6 subjects/week total) and anticipate losing 11%. Even if it is necessary to have 1100 enrolled, enrollment can still be completed by 4.5 years. For our Phase III trial (KUDOS) at the University of Kansas Medical Center we enrolled 350 subjects in 42 months despite a relatively slow enrollment in the first 6 months of the study.

Women expecting multiple infants will be excluded, because multiple fetuses increase risk of preterm and low birth weight delivery for reasons other than hypothesized reduction in inflammation with DHA. Availability by telephone is necessary for optimal coordination in both phases of the study. We routinely obtain additional phone numbers of friends and family who have a stable address. The complete inclusion and exclusion criteria are illustrated in Table 1.

Table 1: Inclusion and Exclusion Criteria

Inclusion Criteria

1. Pregnant females 18.0 years and older 12 to 20 weeks gestation at study entry
2. Agree to consume study capsules and a typical prenatal supplement of 200 mg DHA
3. Available by telephone
4. Able to speak and read in either English or Spanish language

Exclusion Criteria

1. Less than 18 years of age at enrollment
2. Expecting multiple infants
3. Gestational age at baseline <12 weeks or >20 weeks
4. Unable or unwilling to agree to consume capsules until delivery
5. Unwilling to discontinue use of another prenatal supplement that contains ≥200mg DHA per day
6. Women with allergy to any component of DHA product (including algae), soybean oil or corn oil

C.1.b. Placebo and DHA supplementation. A marine algae oil source of DHA (DSM, Columbia, MD) will be provided in capsules. Specific capsules to be used are equivalent to Spring Valley Algal-900 DHA Dietary Supplement Softgels, 450/mg per capsule. The algal oil capsules in this study provides 800 mg DHA in 2 1-g

capsules and the higher DHA group of subjects will be asked to consume 2 capsules per day. The placebo control group will receive 2 1-g capsules containing half soybean oil and half corn oil. The soybean and corn oil combination does not contain DHA. Two capsules provide 80 mg of α-linolenic acid, a precursor of DHA. On average, US adults consume ~1000 mg/d of α-linolenic acid but can make only about ~40 mg DHA/day. Both capsules will be prepared and provided with orange flavor to mask the taste if there is eructation. The placebo and masked DHA capsules will be provided in bottles of 100 capsules (a supply for 50 days).The capsules will be donated by DSM (see letter of support). DSM will also donate 200 mg DHA capsules to both groups for daily use. These are already available commercially as a prenatal supplement under several product names. The 200 mg capsules will be provided in bottles of 135 capsules (135-day supply) and will be marked with an expiration date. Other fatty acids found in the capsules do not contribute significantly to the amounts in the diets of US women. DHA is the only fatty acid expected to change in the RBC-phospholipids (PL) of the supplemented group. Capsule compliance was excellent in our KUDOS trial, on average 74% of capsules were consumed.

C.1.c. Capsule records and accountability. The Investigational Pharmacy at The University of Cincinnati will mail capsules to each enrolled subject on a regular schedule until they give birth; and the bottles and any remaining capsules will be returned by mail to the Investigational Pharmacy in a self-addressed envelope provided with the capsule mailing, the remaining capsules counted, the number of capsules remaining recorded and the capsules destroyed. Records of capsules mailed to and received back from subjects will be entered into the study database with a flag to investigators at the subject's study site. Investigators at each site will review the database and contact subjects who do not return their capsule bottle. Study personnel will contact the subject by telephone early within the first month and approximately monthly thereafter to determine if there are any problems and encourage compliance. The study investigator, study site staff and subjects will not know which capsules are being consumed by each patient. The Investigational Pharmacy at the University of Cincinnati will receive all bottles of capsules directly from DSM, Columbia, MD and will maintain packing receipts for study products.

C.2. Experimental Design and Study Population. We propose a randomized, double-blind, controlled Phase III Clinical Trial of DHA supplementation during the last two trimesters of pregnancy. *The study is designed to determine as a primary outcome if supplementation with 1000 mg DHA compared to 200 mg DHA/d during the last 2 trimesters of pregnancy can reduce ePTB (<34 wk).* Subjects will be randomized to consume algal oil as a source of DHA (800 mg DHA in 2 capsules) or placebo oil (2 capsules of half soybean oil and half corn oil, without DHA). ALL subjects would receive in addition a prenatal supplement with 200 mg DHA and be required not to use another prenatal with DHA. Capsules will be provided from study enrollment until delivery. Each subject will be provided with 2 capsules of their assigned oil per day.

Subjects will be approached in a clinic at the study sites if a preliminary evaluation obtained shows they meet inclusion and exclusion criteria. Subjects who consent to the study will be asked to provide blood on the day they are enrolled for fatty acid analysis of RBC-PL fatty acids, sRAGE and to bank plasma, serum and white cells for future nutrient, genetic and biomarker analysis. Subjects will complete either the National Cancer Institute Diet History Questionnaire (DHQ-II) or three separate 24-hour dietary recalls to obtain information regarding maternal dietary intake. The DHQ-II and 24-hour recalls will be reviewed by study staff and subjects will receive a phone call if clarification is needed. Additional questions will be asked regarding nutritional supplement intake prior to and during pregnancy. Reproductive, smoking and alcohol history will also be obtained. Maternal blood will be obtained during the hospitalization for delivery and cord blood will be collected at birth. Maternal and cord RBCs will be analyzed for PL fatty acids. Maternal and cord blood samples will be stored and batched for analysis of sRAGE. The remaining RBC, plasma, serum and buffy coat will be stored at -80 degrees for possible future nutrient, genetic and biomarker analysis. The process to determine the number of subjects enrolled in each group, details of the randomization process, and the rules for terminating the study are detailed in **C.2.a.**

In an effort to engage underrepresented minorities and better understand the impact of acculturation on their nutritional literacy and status, we plan to oversample Spanish-speaking and Latino participants in Kansas City. Specific questionnaires regarding their acculturation and nutrition literacy will be assessed by bilingual

staff in a culturally appropriate manner.

Subjects will be contacted as needed (approximately monthly) throughout the pregnancy. During the phone calls, subjects will be asked questions about their capsule consumption and adverse experiences and these will be recorded. The calls will also serve as an opportunity to remind subject of requirements concerning acceptance and return of capsules, as well as obtain 24-hour dietary intake if applicable. Additional contacts by telephone or letter may be made to ensure compliance or retention. The prenatal clinic and hospital record after delivery will be the source document for safety and efficacy outcomes and analysis will be those defined and described in following sections.

C.2.a. Bayesian Adaptive Design. We chose this design with efficiency in mind. There is broad acceptance that Bayesian Adaptive Designs save time and money and lead to more ethical studies (89). The time is right for the use of Bayesian Adaptive Designs in comparative effectiveness clinical trials. Both the Patient Centered Outcomes Research Institute (PCORI), a leader in comparative effectiveness research, and the FDA have adopted policies/guidelines encouraging their use (90). Using adaptive randomization (being able to change how we assign patients to the drugs during the study based on information gained during the study) allows for substantially smaller sample sizes and provides better conclusions about what group is the most effective, because it allows changes to our approach or to stop the study early if strong results are found before the scheduled end of the study (89). We conducted extensive trial simulations comparing different designs measuring the resources (time and number of patients required) and the ability to draw important conclusions about relative efficacy of the two groups and selected the proposed design as the most effective and efficient. The following sections focus on different issues and detail how we determined power, sample size, and duration of this trial.

C.2.a.1. Summary of the Bayesian Adaptive Design

- ❖ We will begin interim analysis once 150 subjects have been enrolled in each group
- ❖ Thereafter, interims will occur every 13 wks with data used on all births (intent-to-treat)
- ❖ There are many parameters that go into a Bayesian Adaptive Design and here are the highlights
- ❖ After 800 subjects have been enrolled, at each interim:
    - Stop for success if probability(group *j* is best) > 0.99 for either group
    - Update allocation probabilities based on information weighting
- ❖ A maximum of 1100 subjects will be enrolled with an estimated accrual of 6 subjects/wk (~2/wk/site)

C.2.a.2. Statistical Model. The statistical model will evaluate final determination of which group is "best." This is referred to as the group having the lowest rate of early preterm births. For this study the number of preterm births is modeled with a binomial distribution. For the jth group the number of early preterm births, Y_j, is modeled conditional on the number of births, n_j, as a binomial distribution $Y_j \sim \text{Binom}(n_j, \theta_j)$. θ_j is the rate of early preterm births (ePTB) of which we provide "weakly informative" priors, $\text{logit}(\theta_j) \sim N(-3.5, 1.5^2)$. This prior not only provides a design Type I error rate of 5% (see below), it is also very diffuse since the point estimate of θ_j is 2.9% ePTB and 95% interval 0.16%-36%. This is very, very spread out. Using the data and the prior probabilities, we then use Markov Chain Monte Carlo computations to obtain the Bayesian posterior distributions of θ_j respectively for each group as well as calculating probability (group *j* is best).

C.2.a.3. Response Adaptive Randomization. After 150 subjects are enrolled in each group the next round of subjects are randomized using a formula that takes advantage of the information gained from our analyses up to that point. Next subjects are randomly allocated to be enrolled in the jth group proportional to $V_j = [(\text{prob(group } j \text{ is best)} \times \text{Var}(\theta_j)/(n_j+1)]^{1/4}$.This formula assigns more patients to the most promising group. <u>The study remains blinded throughout.</u> In response to reviewer concern that adjustment after 150 enrollments in each group would be too soon, Dr. Gajewski explored an alternative adaptive design beginning after 300 enrollments in each group. In the original protocol 1200 births were assumed and the sample size changed from 938 with 60% in the winning group to 940 with 56% in the winning groups. The average recruitment length changed from 184 to 183 weeks. We chose to stay with adjustment after 150 enrollments in each group because it resulted in more subjects on the better dose than the later adjustment.

C.2.a.4.

Power, Sample Size, Trial Duration, and Allocation. For the purposes of this investigation we looked at several virtual (or "pretend") responses to determine the power, sample size, time (duration), and subject allocation needed for our study. We created several scenarios for ePTB rates. We performed five sets of trial simulations based on the various combinations of response shown in Table 4. Each set involved 1000 trial simulations. We highlight two scenarios. The first uses a slightly more conservative result than the KUDOS trial to predict what we believe is the most likely response **(scenario #1 in Table 2)**. If there is a best group in terms of ePTB rate, we estimated (identified) that 85% of the simulated trials had early success and 2% had late success. This trial scenario had 87% power and the sample size of this trial scenario was on average 927 (59% of these in the winning group). The average length of this trial scenario was 180 weeks. While a conventional equal randomization trial would have 89% power, it would be larger (1100 subjects), slower (209 weeks), and have a lower rate of subjects on the winning group (50%). The second is the highly unlikely scenario that serves as our null hypothesis **(scenario #5 in Table 2)**. In this scenario there is no difference in ePTB between the groups. Therefore, the extent to which this scenario is "successful" actually reflects our Type I error rate. For this scenario, we estimated (identified) that 5% of the simulated trials had early success, 0% late success. Thus this trial scenario produced an appropriate expected Type I error (α=5%). The sample size of this scenario on average was 1092 subjects (equally allocated across groups). The average length of the trials under this scenario was 208 weeks.

Table 2. Simulated Trial Operating Characteristics.

Scenario	%. Finish Early	%. Finish Late	Power	Mean Subjects	%Group 1	%Group 2	Mean Trial (Weeks)
#1. very likely (4 vs 1%)*	85%	2%	87%	927	41%	59%	180
#2. likely (3 vs 0.5%)	85%	2%	87%	927	42%	58%	180
#3. unlikely (3 vs 1%)	57%	2%	59%	1002	44%	56%	193
#4. very unlikely (3 vs 2%)	14%	1%	15%	1078	47%	53%	206
#5. no difference (3 vs 3%)	5%	0%	5%	1092	50%	50%	208

*Based on our planned enrollment and US 2012 ePTB rates of black and white pregnancies, we anticipate 4.1% ePTB in the control group.

C.2.b. Randomization. Pregnant women will be randomized to one of two arms (groups) with a maximum number of pregnant women n_{max} = 1100 enrollments with 5% expected dropout. Each study site location will have a separate randomization code. Using a Bayesian Adaptive Design, at each interim analysis a decision will be made. Depending upon the birth outcome the randomization structure will be updated. The primary endpoint, percentage of ePTB, is used to drive the adaptive randomization. After we have 150 women in each group enrolled the data will be analyzed and an updated randomization schedule will be used. The arm that looks to be the best will get more pregnant women allocated to it in this subsequent randomization. A new adaptive randomization schedule will be updated every 13 weeks, using up to date outcome data, until the trial is stopped.

C.2.b.1. Implementation. An initial allocation table would be generated (Master allocation table) with all the factors being considered (i.e. maximum sample size, endpoint etc…), this allocation table would be attached to our eResearch tool (91). Once every patient gets an enrolled status they would be assigned to an ARM (Arm A, Arm B) using the randomization module within eResearch. After enrolling 150 patients, the latest data from the system would be analyzed to generate a new allocation table; this new table would be appended (ignoring all the unused assignments on the allocation table) to the existing allocation table that is already being used, this process would be repeated at every 13 weeks, until we reach the end of the trial.

C.2.b.2. Randomizing a Patient in eResearch

- Subject demographics are entered
- Subject is attached to study (at this point patient status could be screening or pre-screening)
- Once the subject is eligible to be enrolled in the study and is ready for randomization enrolled status should be added, which will trigger the randomization and randomize the patient to a particular arm
- Once enrolled, the system automatically assigns a study ID obtained from our initial allocation table

C.2.c. Attrition. If a patient withdraws from the study prematurely, the assessments described at delivery that apply will be obtained if available and the subject has not requested her data not be obtained. This, and the requirement to obtain medical records for adverse events during pregnancy and following birth of the infant, will be explicit in the consent form subject to any provisos made by the Central IRB and HIPAA. If the subject is withdrawn due to an adverse event(s), the patient will be monitored until the adverse event has resolved or until the event is determined to be due to a stable or chronic condition. The reason for patient discontinuation will be documented. If a patient withdraws from the study, the patient's study number will not be reassigned.

C.2.d. Study Termination. <u>The study will be stopped when there is a Pr>0.99 that the groups are different or terminate when 1100 women have been randomized .</u> The DSMB may terminate the study if in the opinion of the safety monitor there is a determination of unexpected, significant, or unacceptable risk to the patient, however, this is not anticipated given that safety concerns have not arisen in other clinical trials in pregnant women that provided large amounts of DHA. Any action taken to suspend or terminate the project by the DSMB will be reported to the Central IRB and the NIH Office of Sponsored Projects and the program director at NIH.

C.2.e. Blood collection. Maternal blood samples are collected by venipuncture at enrollment and during the antenatal hospitalization when delivery is imminent or as soon as possible after delivery if the antenatal sample is not obtained. On both occasions, two 4-ml potassium–EDTA (lavender top) tubes will be obtained (BD Vacutainer, Franklin Lakes, NJ), placed on ice immediately and processed within 24 hours. At KUMC, additional one 7-ml potassium-EDTA (lavender top) tube and one 5-ml serum (red top) tube will be collected at enrollment. Plasma, buffycoat and anticoagulated RBCs will be separated by centrifugation (3000xg, 10 minutes, 4°C). Cord blood will be obtained at the time of birth in two 4-ml potassium-EDTA tubes and processed similar to the maternal samples. All samples will be stored under nitrogen at −80°C for planned fatty acid and sRAGE analysis or banked for future evaluations.

Fatty acid analysis. Fatty acid content in red blood cells (RBC) will be analyzed by gas chromatography. Briefly, an aliquot of packed RBCs is extracted with organic solvents and the dried under a nitrogen stream before transmethyation with boron trifluoride-methanol (5). The fatty acid methyl esters are extracted into organic solvent, dried under a nitrogen stream and reconstituted in dicholoromethane for analysis on a gas chromatograph with flame-ionization detection equipped with an autosampler using a fused silica capillary column (SP2560, 100m x 0.25mm id x 0.25um film thickness). Helium is used as the carrier gas. Fatty acid analyses will be completed at the University of Kansas Medical Center RBC-DHA and other fatty acids are reported as weight percent of total fatty acids. RBC-DHA can also be used to evaluate compliance.

sRAGE analysis. sRAGE will be determined at Nationwide Children's Hospital in Columbus, OH on batched samples using ELISA-based format (MesoScale Discovery, Rockville MD) according to the protocols of the manufacturer.

Cell free RNAs. Plasma obtained at enrollment and at delivery from all three sites will be used to determine a priori risk of experiencing spontaneous preterm birth by measuring a panel of cell free RNAs. The 4 markers used in this panel appear in *in vitro* studies to alter myometrial quiescence. In validation studies of maternal samples from pregnant women at gestational ages similar to the current study, the spontaneous preterm labor panel has a sensitivity of 100%, specificity of 95%, positive and negative predictive values of

95% and 100% respectively for spontaneous preterm birth less than 32 weeks gestation. Should the administration of DHA alter the predicted spontaneous preterm birth rate in participants with an abnormal panel, it would provide insight as to the mechanisms by which DHA might work.

Banked Sample: At KUMC, one additional 7-ml potassium-EDTA (lavender top) tube and one 5-ml serum (red top) tube will be collected at enrollment for storage and possible future nutrient and biomarker analysis.

C.2.f. Dietary and Nutrient Intake. Either The National Cancer Institute Diet History Questionnaire (DHQ-II) or three separate 24-hour dietary recalls will be used to collect information on subjects' dietary intake. A DHA specific questionnaire will be administered to all participants.

National Cancer Institute Diet History Questionnaire DHQ-II The DHQ-II is the current validated version of a food frequency and portion questionnaire intended for use in an English-speaking population. The database associated with the DHQ-II is based on the National Health and Nutrition Examination Surveys (NHANES) data collection from 2001-2006. English-speaking participants will complete the DHQ-II at enrollment using electronic/tablet entry. Paper copies will be available for participants that reject electronic entry. The study teams at each site will provide instruction as to how to complete the questionnaire and review DHQ-II results for completeness. Additional study staff may follow up with the study participant by phone after enrollment if there are concerns regarding missing data, inconsistences, etc.

The project will be registered at the NCI website and all electronic data will be stored securely under the PI user name and password until study end. Data will be analyzed using the Diet-Calc software. The DHQ-II provides data for 176 nutrients, dietary constituents and food groups.

24-hour Dietary Recall. For participants who speak Spanish, or who live in the Kansas City area and self-identify as Latino ethnicity, three 24-hour dietary recalls will be collected by trained bilingual staff either in person at the baseline visit, or over the phone. We will use Nutrition Data System for Research (NDSR). NDSR which is a computer-based software application developed at the University of Minnesota Nutrition Coordinating Center (NCC) that facilitates the collection of recalls in a standardized fashion (92). Dietary intake data gathered by interview is governed by a multiple-pass interview approach. In the case of this trial, three distinct passes provide multiple opportunities for the participant to recall food intake. The first pass involves obtaining from the participant a listing of all foods and beverages consumed in the previous 24 hours. This listing is reviewed with the participant for completeness and correctness and the interviewer then collects detailed information about each reported food and beverage, including the amount consumed and method of preparation (second pass). In the final pass, the interviewer then probes for commonly forgotten foods and reviews for completeness and correctness.

Dietary supplement use will be assessed in conjunction with collection of 24-hour dietary recalls using the Dietary Supplement Assessment Module included in NDSR. Use of all types of dietary supplements and non-prescription antacids are queried in the module. The Nutrition Coordinating Center (NCC) Food and Nutrient Database serves as the source of food composition information in NDSR. This database includes over 18,000 foods including 8,000 brand name products. Ingredient choices and preparation methods provide more than 160,000 food variants. Values for 165 nutrient, nutrient ratios and other food components are generated from the database. The USDA Nutrient Data Laboratory is the primary source of nutrient values and nutrient composition. These values are supplemented by food manufacturers' information and data available in the scientific literature. Standardized, published imputation procedures are applied to minimize missing values.

DHA Food Frequency Questionnaire (DHA FFQ). This questionnaire includes targeted questions to assess the intake of omega-3 fatty acids accurately (93). Results of the questionnaire will be entered into the CRIS database.

C.2.g. Nutrition Literacy. At enrollment or a subsequent study visit, a subset of subjects of Latino ethnicity in Kansas City area, who provide additional consent, will complete the Nutrition Literacy (NLit) questionnaire, a

multiple choice questionnaire that measures their nutrition knowledge (94). The 66-item questionnaire will be administered using electronic/tablet entry, or by paper for participants that reject electronic entry. Five domains of nutrition knowledge are assessed with the NLit, including: nutrition and health, macronutrients, household food measurements, food label and numeracy, food groups, and consumer skills. Subjects will be scored as low or high nutrition literacy level. Two questions related to access to nutrition information will also be included: has the participant had a consultation with registered dietitian (yes/no), and where does the participant most often access nutrition information (television, internet, magazines, friends/family, doctor, other).

C.2.h. Acculturation. Because acculturation indirectly impacts the health of minorities, acculturation proxy questions and the General Acculturation Index (GAI) will be administered at enrollment by electronic/tablet entry or by paper to a subset of subjects of Latino ethnicity in Kansas City who provide additional consent (95, 96). Two questions will be used as acculturation proxies: place of birth and time living in the US. For the place of birth we will include all the 20 countries of Latin America, plus the US and ‘other’. For the time living in the US, three options will be available (0 to 5 years, 5 to 10 years, 10 years or more). The GAI will be used as a unidimensional measure of acculturation. The scale includes five questions about reading/speaking language preferences, country where participant grew up, ethnicity of friends, and ethnic pride. Answers will be measured on a 5-point Likert scale and then averaged. The final acculturation score will range from 1 to 5.

C.2.i. Mediators of Diet Quality and Nutrition Literacy. To assess the relationship between diet quality and nutrition literacy we will evaluate food security, theory of planned behavior (TPB) and quality of life among a subset of English and Spanish-speaking Latinas who agree to participate. We hypothesize these measures will be important predictors of nutrition literacy and diet quality.

- Food Security: Midway through pregnancy we will administer the USDA Household Food Security survey (97) by phone. The 18-item questionnaire is available in both English and Spanish and is well validated.
- Theory of Planned Behavior: At enrollment we will administer the Shah et al. theory of planned behavior survey (98) to evaluate perceived behavioral control. The 12-item questionnaire is available in both English and Spanish.
- Quality of Life: At enrollment we will administer the CDC HRQOL-4 “Healthy Days Measure” (99). The 4-item questionnaire is available in both English and Spanish and is well validated.

C.2.i. Optional Urine collection. One non-sterile urine sample (minimum collection of 8 mL) in a 4 oz. specimen collection container will be obtained between 12-20 weeks gestation and again during the 3rd trimester among a subsample of women who consent and are available in our primary hospital clinics. The levels of endocrine disrupting chemicals (EDC) will be quantified. We will explore if the presence of EDCs in maternal urine differs between the treatment groups. The sample will be divided into four 2.0 mL cryovials using a disposable transfer pipette. Cryovials will be labeled and frozen at -80°C until analysis.

C.3. Data Collection and Integrity. Phase III Clinical Trials have a formal system for data collection and scrutiny in accordance with Good Clinical Practices (GCP) and conform with the regulatory requirement(s) (100). Data collection and entry for all aspects of the study will be performed by persons at each site. Study staff will adhere to trial-specific Stand Operating Procedures (SOPs) approved by trial PIs. Clinical teams at each site will maintain essential source documents including clinical and hospital reports. An electronic case report form that includes historical information obtained from subjects not in the hospital or clinical record (e.g., detailed smoking history, alcohol use – number and type of alcoholic beverages before and during pregnancy using the Nutrition Educators of Health Professionals tool and DHA FFQ obtained at study entry) will be maintained as the primary source document for this information. Whenever possible site staff will use a two-pass approach to confirm accuracy of social/historical information not in the hospital or clinical record to ensure accuracy (i.e., staff will review data entry with subjects in person or by phone interview). One hundred percent of data for the primary outcome and safety (mother and newborn) and a portion of other data collected will be double-checked by the clinical teams after the data are entered into the eResearch by reviewing source

documentation. The proportion of secondary outcome data will be determined by the PIs based on the actual incidence of errors observed in data entry. These master files will be established for each subject and maintained for the duration of the trial and retained according to the appropriate regulations.

Accuracy, completeness, legibility and timeliness of the data reported in the patient's electronic case report form will be assured by the PI. Source documentation supporting the case report form data will document the dates and details of study procedures, adverse events and patient status. Any discrepancies will be explained by a note to the individual file and changes or corrections to the electronic case report form will be dated, initialed and explained (if necessary). Original data will not be obscured.

The analyst in Biostatistics (Brown) will work with the study PIs to ensure data validity and accuracy by performing edit, logic and range checks on the study database and sending queries for resolution to the clinical team. Once all database queries have been resolved by the clinical trams, she will be responsible for creating data sets for analysis (interim and final). In collaboration with Director of Research Information Technology (Mudaranthakam), Brown will finalize the study binder, which will contain copies of the annotated project Case Report Forms, the final data dictionary, and copies of the electronic data files. Ms. Brown will perform the Bayesian adaptive design modeling and provide Mr. Mudaranthakam updated randomization for the study. Ms. Brown will perform initial database restructuring in preparation for analysis by Dr. Gajewski, and she will also conduct initial analyses under the direction of Dr. Gajewski.

Mr. Mudaranthakam and Ms. Brown will build the study database and electronic case report forms into our web-based data management system, eResearch. They will be responsible for training study personnel across all sites on entering data into this system and will provide ongoing training and help desk support over the entire study time-period defined in the protocol. Mr. Mudaranthakam will also provide custom coding during the first two years of the study in order to provide the adaptive design randomization to each of the study sites utilizing the eResearch system.

Using the interim data and the Bayesian Adaptive Design formulas, Dr. Gajewski would define the parameters to generate the initial allocation table; this allocation table is associated with study and loaded into the oracle database which acts as the backend for the eResearch.

C.4. Primary Efficacy Outcomes

- Early preterm delivery (ePTB, <34 wk gestation) based on ACOG guidelines

C.5. Secondary Efficacy Outcomes

- VLBW (<1500 g) and low birth weight (<2500 g) as recorded in hospital record
- Subject DHA status (RBC-PL-DHA) at enrollment and birth; fetal DHA status at birth from cord blood
- Gestational age (days) at delivery based on EDD in clinic record recorded following ultrasound on or before ~14 wk gestation
- Birth weight (g), length (cm) and head circumference (cm) at delivery as recorded in hospital record
- Pre-term birth (<37 weeks) based on ACOG guidelines
- Pregnancy outcomes: gestational diabetes, pre-eclampsia, C-section, spontaneous or induced labor, occurrence and reason for non-routine hospitalization

C.6. Statistical Methods.

C.6.a. Evaluation of effectiveness of randomization. We will evaluate if the randomization was effective by ensuring that the groups are similar at baseline (pre-randomization variables) for all such variables listed in C.6.d and will be presented in a table per CONSORT guidelines (101).

C.6.b. Primary and Secondary Pregnancy Efficacy Analysis (Specific Aim 1). The pregnancy analysis has two overall phases. The first phase of analysis (primary) investigates, using Bayesian posterior probability (group *j* is best, whether there is a simple difference between groups (no adjustment of predictor variables). A similar continuous Bayesian model will test sRAGE differences between groups. The second phase of analysis (secondary) uses Bayesian multiple logistic and continuous regression for detailed investigation as to why there are differences between groups.

All analyses will be conducted under intent-to-treat principles. Subjects will remain in the group for which

they are randomized regardless of compliance. The consent form will state that medical records may be obtained from the clinic and hospital (for mother and baby) unless a specific request in writing disallowing us to obtain records is received. Nevertheless, we do anticipate some missing data. To handle missing data, logistic regression will evaluate missing data patterns as a function of subject demographics. The two categories will be "drop-out" and "did not drop out." This analysis will help us understand the missing data pattern (missing at random, missing completely at random).

C.6.c. Pregnancy Efficacy Analysis According to Intent-to-treat Principles. In the first phase of analysis, we test the differences between groups for the primary efficacy outcome (ePTB). The approach is repeated for secondary outcomes using Bayesian logistic and continuous regression (VLBW (<1.5 kg), maternal RBC PL DHA) with no covariates.

C.6.d. Pregnancy Efficacy Analysis Controlled for Potential Predictor Variables. The goal here is to find out how well DHA supplementation predicts our primary and secondary efficacy outcomes after controlling for potential predictor variables. In the second phase of analysis, using multiple linear regression (logistic and continuous), we explore the relationships among predictor variables for regression (listed in C.6.e) and all primary and secondary pregnancy outcomes (C.4, C.5). <u>The predictor variables represent five general classes: overall DHA intake, diet (intake of nutrients and foods analyzed by principal component analysis), environment, subject demographics, and maternal medical history</u>. For exploratory purposes, the most important relationship is between the DHA dose and pregnancy outcomes. Notice that instead of a grouping variable representing groups, we utilize DHA in the form of several predictor variables, depending on their source.

- ❖ A final exploratory analysis investigates the impact of capsule intake on outcome as mediated by maternal RBC-PL DHA. Using a reasonable set of predictor variables from the regressions above, we will run two sets of regressions for each outcome variable. This will allow maternal RBC DHA to be a mediator. First we regress the RBC-PL DHA level on all appropriate predictor variables (as above). Then we will run a regression of outcome variables on RBC-PL DHA level and all other appropriate predictor variables (as above but with RBC DHA added). In this way, we are running a path analytic model where we can obtain direct effects of variables and indirect effects of variables through mediator plasma level.
- ❖ In all regression analyses we will investigate local model adequacy by exploring standardized residuals and leverage points via Cook's distance. Possible co-linearity among predictor variables will examined with Pearson's correlation coefficient and variance inflation factors (VIF). Scatter plots and histograms will also be used to investigate the adequacy of the model assumptions.
- ❖ Of substantive interest in the regression analysis is that race is one of the predictor variables. Since we anticipate 22.5% of the subjects to be of African descent, we can test whether efficacy of pregnancy outcomes is different for women of African descent relative to other races.
- ❖ For regression analysis purposes, the pregnancy outcomes are separated into two classes of variables, either continuous or dichotomous. For the continuous variables, Bayesian regression based on the normal distribution will be utilized. For the dichotomous variables, logistic regression will be utilized.
- ❖ For all outcomes, we set the DHA dose as a predictor variable and then fit all possible subsets of the other predictor variables to explore, for the particular pregnancy outcome, which model is the best. We will utilize a global fit index called Deviance Information Criteria (DIC) to determine which variables to keep in the final model. The DIC is very general and can be used for normal and logistic regression analyses.

C.6.e. Potential Predictor Variables for Regression Analysis

<u>Class 1: Overall DHA intake</u>

- ❖ DHA dose (capsules taken multiplied by the DHA in the type of capsule consumed)
- ❖ maternal RBC-PL DHA level at enrollment and delivery (g DHA per 100 g total fatty acids) by chromatographic analysis (C.2.e)
- ❖ cord RBC DHA at birth by chromatographic analysis (C.2.e)

- estimated DHA intake at enrollment from DHA FFQ, and frequency/amount of consumption of food and supplement sources containing DHA (see C.2.f)

Class 2: Diet

- estimated DHA intake at enrollment from DHA FFQ, and frequency/amount of consumption of food and supplement sources containing DHA (see C.2.f)
- intake of other nutrients or foods, e.g., macronutrient quantity or quality, micronutrient quantity at enrollment

Class 3: Exposure to environment

- tobacco exposure prior to and during pregnancy by subject report
- alcohol intake prior to and during pregnancy by subject report defined as standard drinks/day (Nutrition Educators of Health Professionals Teaching Tool)
- measurement of endocrine disrupting chemicals (EDCs) in urine at 12-20 weeks and during the 3rd trimester.

Class 4: Subject demographics

- marital status by subject report
- household income by subject report
- insurance type (private, public, uninsured) by review of clinic/hospital record
- maternal and paternal education by subject report
- maternal age at enrollment (years) from DOB listed in clinic/hospital record
- maternal and paternal race/ethnicity from clinic record or subject report
- fetal sex

Class 5: Maternal medical history

- BMI calculated from self-reported pre-pregnancy weight or measured 1st prenatal clinic weight record and measured height
- gestational weight gain (last clinic visit in pounds minus pre-pregnancy weight or 1st measured weight)
- gestational age at enrollment (days) calculated from EDD (based on ACOG guidelines)
- reproductive history
- characteristics of previous pregnancies (early preterm birth, pre-eclampsia, gestational diabetes)
- blood pressure throughout pregnancy
- iron status / hemoglobin at enrollment and mid-pregnancy
- cervical length between 18-22 weeks gestation
- estimated blood loss at delivery
- infant APGAR scores
- meconium in amniotic fluid
- evidence of illicit drug use from clinic/hospital record

C.6.f. Adverse Events Analyses. Fisher's exact test will be used to compare the incidence of maternal and infant adverse events between treatment groups. All p values will be evaluated at the $\alpha = 0.05$ level. No adjustment for multiple comparisons will be made.

C.7. Adverse Events and Safety Monitoring

C.7.a. Definitions of an Adverse Event. An adverse event (AE) is any reaction, side effect or other undesirable event that occurs in conjunction with the use of the test product, whether or not the event is directly related to the test product. New and/or worsening signs and symptoms of underlying or emerging disease will be recorded as an adverse event if they might be clinically or scientifically related to DHA intake. Additionally, any patient complaint reported as possibly related to treatment will be recorded as an adverse event.

C.7.b. Definition of a Serious Adverse Event. Any adverse event that results in the following is considered a serious adverse event (SAE): 1) death; 2) a life-threatening event; 3) inpatient hospitalization or prolonging of an existing hospitalization; 4) a persistent or significant disability/incapacity or 5) a congenital anomaly/birth defect. Medical events that may not result in death, be life-threatening, or require hospitalization may be considered an SAE when, based upon appropriate medical judgment, they may jeopardize the patient and may require medical or surgical intervention to prevent one of the outcomes listed above.

C.7.c. Categories of Adverse Events. Adverse event collection is limited to four categories. 1) SAEs, 2) patient-reported complaints reported as possibly or definitely related to treatment, 3) sign and symptoms that might be clinically or scientifically related to DHA-intake and 4) other important medical events that do not result in SAE, but may jeopardize the participant and may require medical or surgical intervention to prevent an SAE. Events that are common to pregnancy or lack clinical/scientific significance to DHA will not be recorded* *(change in protocol a result of May 2017 decision by PI team, DSMB and medical monitor. Approved by IRB in version 0.11).

C.7.d. Timeline of Adverse Event Collection and Review. Adverse events, that meet one of the four categories in C.7.c will be collected from enrollment through 30-days postpartum, or until the infant is discharged from the hospital. AEs will be identified from medical records and by periodic phone interview when the study coordinator calls to check for supplement compliance and tolerance. All adverse events will be documented in eResearch and reviewed by site or trial PIs weekly. Only site and trial PIs will assign attribution. Immediate attention will be given to events that are (or are suspected to be) serious, unexpected, and related or probably related. Reportable adverse events (i.e. events that are unexpected and related or probably reported) will be provided to the Central IRB and DSMB chair within 5 working days. Deaths that occur within 30 days of the last dose of the study drug will be reported to the Central IRB by phone or email within 24 hours of notification to the PI or research team, followed by a report to the IRB within 5 working days.

C.7.d. Safety Monitoring. A DSMB composed of two neonatologists, one pediatric pharmacologist and one pediatric epidemiologist will meet yearly and generate a report to the PIs and IRB. Dr. Daniel Robinson, a neonatologist, will serve as the medical monitor and will chair DSMB discussions. He will receive all reportable adverse events (i.e., events that are unexpected *and* related or probably related) within 5 working days after the investigators learn of the event. He will also be provided a complete list of all AEs by the study analyst (Brown) prior to each DSMB meeting. Dr. Robinson may request the actual product assigned to an individual if needed. After all data for the study have been monitored, entered, cleaned and locked, a safety report will be generated by the study analyst with input from Dr. Robinson to generate the safety report for publication.

C.8. Potential Limitations. We do not anticipate any major potential limitations as we developed strategies to successfully minimize issues that arose in the KUDOS trial. For example, in the KUDOS trial, we encountered a problem that we had not anticipated, i.e., the profusion of prenatal supplements with DHA ranging from 100 to 300 mg/dose that became available during the trial. Consequently this was not an exclusion criterion and 15% of women in the study (both those assigned to DHA and to placebo) took DHA from one of these sources as well as their assigned capsules (placebo or DHA). We propose to provide all women with the same low dose supplement of DHA (200 mg, an amount also recommended by WHO/FAO, and exclude women who take any other DHA supplement. *In KUDOS, the intent-to-treat analysis showed a benefit of 600 mg/d DHA to reduce ePTB compared to the placebo group despite intake of additional DHA by some subjects suggesting that a low-dose supplement in the placebo group will have little or no effect on ePTB.*

We anticipate that some women will not return their capsules for easy evaluation of compliance. Again we have developed a strategy to minimize this: We ask about intake in our monthly calls and keep individual records that can be used to compare to the number returned and used to estimate compliance if they are not returned. Women are given $50 at enrollment and $50 at delivery for their time and trouble, including compliance with returning their capsule bottles and unused capsules and calling when they are admitted to the hospital for delivery.

We anticipate some telephones being disconnected and lack of compliance to take prenatal supplements.

We obtain additional telephone numbers from subjects for relatives and friends. Women in KUDOS reported regular prenatal vitamin intake (99%) and were not averse to consuming additional capsules. Phone call and visits when subjects are in clinic encourage compliance and rapport with subjects before delivery.

C.9. Study Timeline (Planned Bayesian Adaptive Design) in contrast to conventional randomization.

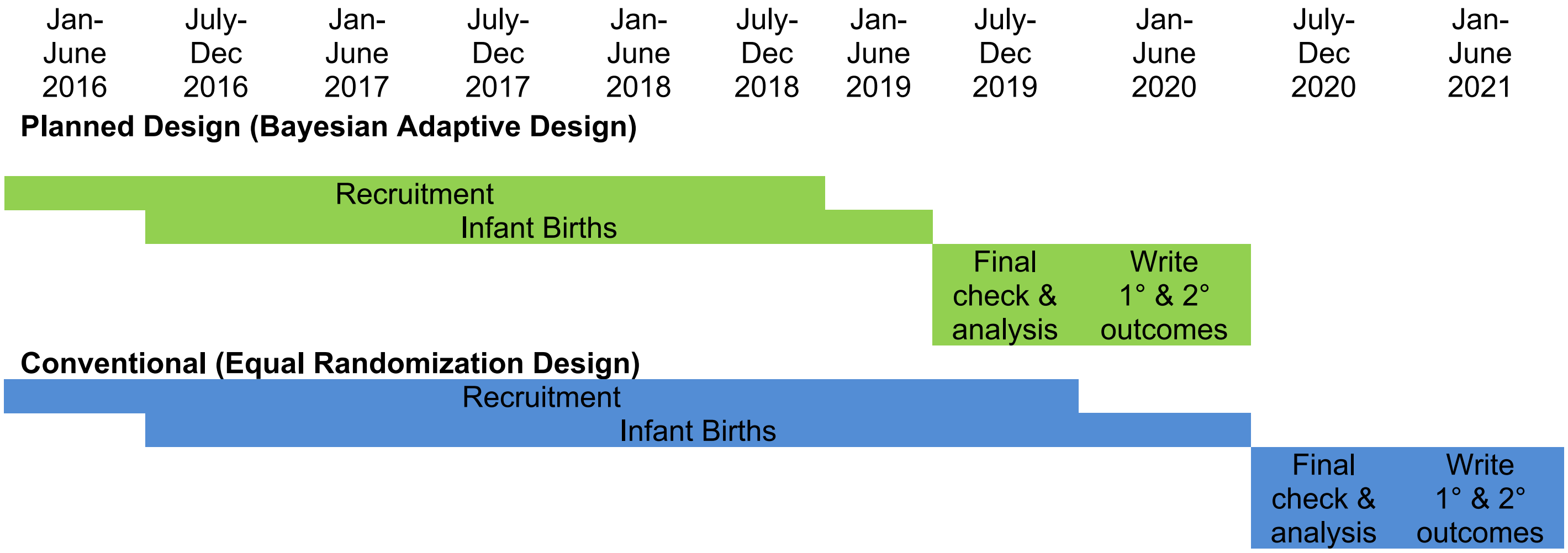

PROTECTION OF HUMAN SUBJECTS

The University of Kansas Medical Center, University of Cincinnati, Ohio State University and Nationwide Children's IRBs will review and approve this study. The University of Kansas Medical Center has agreed to serve as the central IRB for this research project. The project is registered with ClinicalTrials.gov (NCT02626299). Consent forms and HIPPA disclosure information per IRBs are given to all subjects and their signatures witnessed by research staff. The consent forms include a description of the study, nature of the data collection and potential benefits from continuing in the extended assessment phase of the RCT. Subjects are told that their usual care would not be changed, withdrawn, or reduced if they chose to withdraw from the study at any time. The research team personnel will abide by all tenets of the Universities confidentiality polices, as well as the Privacy Protection for Research Subjects. All research staff will remain current in their NIH required Human Subjects protection and HIPAA certification.

In order to maintain patient privacy, all case report forms, study reports and communications identify the patient by the assigned patient number. Data monitors and auditors from the IRBs, and regulatory authorities have access to the patient's original medical records for verification of data gathered on the case report forms and to audit the data collection process. Subjects are made aware of persons who may see their protected health information in the informed consent document and may choose not to enroll in the study based on the information provided them in accord with 2003 HIPAA regulations. The patient's confidentiality is maintained and is not made publicly available to the extent permitted by the applicable laws and regulations.

All IRB correspondence and documentation related to the study including reports to and from the study DSMB will be kept up-to-date at each site by the site study coordinators under the supervision of Carlson (University of Kansas Medical Center) or DeFranco (University of Cincinnati) and Cackovic (Ohio State University). Dr. Valentine will provide oversite at both the University of Cincinnati and Ohio State University. Adverse events will be uploaded as soon as they are found to eResearch system built under the supervision of Dr. Gajewski and both site and trial PIs will have the ability to review and assess attribution. The medical monitor and DSMB will have access to adverse event reports.

1. Human Subjects' Involvement and Characteristics

The study population for this proposal will be women between 12 and 20 wk gestation with a singleton pregnancy. . Smoking is relatively common among women in our populations. We know that smoking is related

to the primary and secondary outcomes proposed (including preterm birth and lower birth weight) and analysis of efficacy will be evaluated for potential predictor variables including smoking and nutrient intake. We obtain detailed information on smoking in all of our studies so that we may calculate total use (pack years) as well as use of smoking before, during and after pregnancy.

2. Sources of Materials

Research material or data collected will be from subject interviews, medical records (with IRB and privacy board approval and patient permission), and blood analysis. All data will be gathered for the explicit purposes of this study using procedures to ensure confidentiality. All data will be identified by a code number only. All subjects will be encouraged to contact the Human Subjects' Committee with any concerns about the study. The study will remain blinded to all study personnel until all data for the initial trial are collected, cleaned and locked. Only the Investigational Pharmacy and data analyst have the actual study assignment of each subject enrolled. All subjects are identified in data files by a code number only. Paper files are kept in locked file cabinets in rooms that are locked each evening and in an area that is restricted to study personnel. Data files are password protected.

3. Potential Risks

No appreciable risk of physical, psychological, social, legal or other harm is expected. DHA is a nutrient and the amount of DHA to be provided (1000 mg) is similar to the amount of DHA and EPA provided in DOMInO (900 mg) and less than the amount provided in several randomized trials conducted in pregnant women in Europe (2.0-2.2 g/d). None of these trials has found any major safety issue. Risks of gastrointestinal upset and bleeding have been documented with omega 3 studies using a fish source of a supplement that often contains the long chain fatty acid -eicosapentaenoic acid (EPA) – we minimize this risk by using a plant source of DHA which contains no EPA. In our previous trial published in Breastfeeding Medicine we did not have these side effects using this supplement. In addition an RCT done in Kansas used 600 mg of this DHA supplement also did not see these risks. We will obtain medical records and additionally ask women to report any burping, gas, diarrhea, constipation, or heartburn. The risk to the fetus and neonate is negligible as this is a nutrient normally preferentially transferred in utero across the placenta and via the mammary gland for eye and brain development.

As this study is a trial of a readily available over the counter nutritional supplement that has been taken and studied at 200 mg and 1000 mg doses we do not anticipate any problems or adverse events. We also completed a pilot trial on postpartum women that reported no adverse events on 1000 mg of algal DHA. No fish product is involved and no allergies have been reported.

Blood will need to be drawn for research and may be associated with pain and the usual risks of bloodletting.

4. Adequacy of Protection Against Risks

a. Patient recruitment and informed consent. We will have a Central IRB at the University of Kansas Medical Center. All recruitment, consent, and data forms for the study proposal will be approved prior to any enrollment. Informed consent for continued testing is obtained by a trained research team member with NIH-approved Human Subjects' protection certification. Consent includes the standard elements: a study description, the potential risks, benefits and options for non-participation. All subjects are informed they are free to withdraw from the study without changes in their usual care. Consent is documented as a signed form and will be kept in a locked file at the study office for each site. The study is conducted in accordance with ethical principles founded in the Declaration of Helsinki. The IRB review includes a review of all appropriate study documentation in order to safeguard the rights, safety and well-being of the subjects. The protocol, informed consent, written information given to the subjects, safety updates, annual progress reports, and any revisions to these documents are provided to the IRB by the principal investigators. The method of obtaining and documenting the informed consent and the contents of the consent will comply with GCP and all applicable regulatory requirement(s).

b. Protection against risk. Adverse events will be collected for 30 days after the woman gives birth, or until the infant is discharged from the hospital with clinic and hospital records as the source documents. The study on which this one is based (KUDOS) did not identify any safety concerns associated with DHA supplementation using a dose of 600 mg/d of DHA, however, we propose to provide more DHA (1000 mg) daily for the last two trimesters. Subjects will be asked to report immediately to the study coordinator any health problems they might incur and to call the site PI or the study coordinator at their site at any time if they have questions or concerns. In such an event, it is the PI or study coordinator responsibility to answer those concerns honestly and to reiterate to the subject that they should continue in the trial only if they feel entirely comfortable with it. Both Carlson and Valentine have done past trials with pregnant women. Subjects who choose to withdraw from the study will be reassured if they indicate the desire to withdraw.

Subjects are protected against the risk of breaking confidentiality by decoupling of names from databases. Paper documents (e.g., signed consent forms) are stored separately in locked file cabinets. Only selected members of the research staff have access to the subjects' data.

Subject's informed consent includes the HIPAA compliance documentation approved by the Central IRB. The research team personnel abide by all tenets of the University confidentiality polices as well as the Privacy Protection for Research Subjects.

Study personnel will identify subjects who might be eligible under a waiver issued by the IRB to look only at medical information related to inclusion/exclusion criteria for the study. No personal health information will be recorded until consent for the study is obtained in writing.

Women will be paid $50 after delivery if they are compliant in mailing back unused capsules or empty capsule bottles. Based on our previous experience, this incentive will be sufficient to encourage compliance with bottle return.

5. Potential Benefits of the Proposed Research to the Subjects and Others

This study could result in outcomes which contribute to new knowledge about the effects of prenatal DHA supplementation on gestation duration and/or pregnancies most likely to benefit.

6. Importance of the Knowledge to be Gained

Early preterm birth is associated with significant morbidity and mortality and cost to both families and society. It is a factor in up to 5% of US births and higher still in less advantaged groups within the US This would be the first study in the US to test as a primary hypothesis that DHA can reduce early preterm birth using a study powered to answer this question. A similar study is underway in Australia, based on a secondary finding from the DOMInO trial that 800 mg DHA + 100 mg EPA reduced early preterm delivery from 2.2 to 1.1% of births. Australia has a much lower rate of early preterm birth and there may be different etiological issues that contribute compared to the US so we believe it is important to study this problem in the US.

7. Data Safety and Monitoring Plan

A Data Safety Monitoring Board (DSMB) will monitor the study for safety. Dr. Ardythe Morrow (Professor in Biostatistics and Epidemiology Center at CCHMC), Dr. Kurt Schibler (Neonatologist and The Medical Director of Clinical research for the Neonatal Netwrok) and Alexander Vinks PhD (Pharmacology expert) will be the members of the DSMB in addition to Dr. Daniel Robinson, as the medical monitor. The DSMB will provide and independent review of the ongoing data, patient reports, adverse events at least yearly, making their deliberation about safety using data of adverse events from all 3 recruitment sites. Through its reviews of the study, the DSMB will determine whether cumulative data indicates the need to change the research design, to modify information presented to participants, or to terminate the project. Any action taken to suspend or terminate the project will be reported to the Central IRB, NIH Office of Sponsored Projects and the program director at NIH. The DSMB and medical monitor will evaluate the final study manuscript(s) and final reports to assure results are fairly presented and conclusions are appropriate.

8. Anticipated Areas of Difficulty

The major area of concern is capsule compliance and return of capsules. However, we discovered in KUDOS that a financial incentive for compliance with capsule return at delivery was quite successful. We will only enroll subjects who have a telephone where they can regularly be reached.

9. Clinicaltrials.gov Registration
We will ensure that the clinical trial is registered on clinicaltrials.gov.

10. Statistical Analysis Plan Modifications
Because of emerging statistical methodology and data released from Australia we have modified our analytic plan, of note: 1) we use a mixture of normal distributions to improve power for detecting differences in early preterm birth rates across groups; and 2) added an analysis that estimates and attributes the interaction between low and high levels of blood level of DHA at baseline and arm and its impact on early preterm birth. Please see the added statistical analysis plan (SAP).

INCLUSION OF WOMEN AND MINORITIES

All subjects are women. A significant proportion of the sample is of African-American descent or Hispanic ethnicity. We will make a concerted effort to enroll Hispanic women in our centers by using Spanish translators and translated informed consents to include subjects who are interested.

Special populations: pregnant women and infants. The research proposal meets the guidelines for studies of vulnerable populations outlined in 45CFR46. In particular, the research has minimal risk but holds the prospect of direct benefit to pregnant women and their infants.

INCLUSION OF CHILDREN

Infants born to pregnant women will all be included in this research. We will obtain their medical records and use these for some secondary outcomes and to monitor safety; cord blood will be available to measure sRAGE and banked for future genetic and biochemical studies.

MULTIPLE PI LEADERSHIP PLAN

Overview

The submission of this proposal with Drs. Susan E. Carlson, Christina Valentine and Byron Gajewski as principal investigators represents a unique collaboration of three scientists with a strong interest in the nutrient DHA but representing different key disciplines and experiences to work together to conduct a clinical trial that requires several clinical sites to meet the planned recruitment goal needed to test the hypothesis that DHA can reduce early preterm birth (ePTB). We embrace the CTSA program concept that it is important to insure "that all translational science is performed in the context of collaborative team science and that shared leadership roles are the norm throughout the entire translational science process" (http://www.ncats.nih.gov/CTSA-IOM-WG-Draft-Report.pdf).

Dr. Carlson is a nutritionist who has conducted NIH funded (NICHD, NEI) randomized clinical trials with DHA supplementation in preterm and term infants and pregnant women since 1986. From 1983 to 1997, she was on the faculty in several Departments of Pediatrics: University of South Florida, University of Mississippi Medical Center (UMMC) and University of Tennessee-Memphis. At both UMMC and UT-Memphis, she was in the Division of Newborn Medicine. In 1997 she moved to the University of Missouri-Kansas City (UMKC) and in 1999 to the University of Kansas Medical Center (KUMC) where she currently serves as a Professor in the Departments of Dietetics and Nutrition and Pediatrics and retains an appointment as Professor of Obstetrics and Gynecology at the UMKC. Dr. Carlson and Dr. Byron Gajewski have collaborated on an NIH funded trial (R01 DHA and Pregnancy Outcome) that has just started the 8th year of funding. The second 5 year funding cycle targets long term cognitive development of children born to women in the clinical trial. The two are also working in collaboration with the PIs from the DOMInO trial in Australia to determine women who most

benefited from DHA supplementation to reduced ePTB in that trial; and have generated preliminary data shared in this proposal.

Dr. Valentine is a neonatologist who initially trained as a dietitian who has conducted 2 trials in maternal dietary DHA and human milk composition that have an FDA IND. She has worked in the field of neonatology as a physician since 2002 and has a goal of improving perinatal nutrition. She is a co-investigator on an NIH funded R01 trial from the Office of Dietary Supplementation to examine the inflammatory homeostasis of preterm infants after maternal DHA supplementation. She was previously at Nationwide Children's Hospital and The Ohio State for 9 years and maintains a strong collaboration with her previous mentor Dr. Lynette Rogers who is the PI on the current R01. Dr. Valentine was an Assistant Professor in the Department of Pediatrics at Cincinnati Children's Hospital until last fall when she became the Medical Director at Mead Johnson Nutrition. She has an appointment at the University of Cincinnati, continues to live in Cincinnati, and has 20% effort donated to the study by Mead Johnson Nutrition (see letter from Dr. Colin Rudolph) to fulfill her role as coordinator of efforts in Ohio (University of Cincinnati and Ohio State University).

Dr. Gajewski is a co-investigator with Drs. Carlson and Colombo on their R01. For the current proposal, Dr. Gajewski's role as a PI is to design and govern the Bayesian Adaptive Design. He has expertise in the design and implementation of Bayesian adaptive designs. He has published new Bayesian clinical trials methodology in a top tier biostatistics journal (Statistics in Medicine), of which one was quoted in NHLBI's RFA-HL-08-013. He has also published two papers showcasing novel Bayesian predictors of clinical trials accrual with co-PI Carlson. He was also successful in gaining PCORI (CER-1306-02496) funding using a novel Bayesian adaptive design. His experienced team in Biostatistics will also set up and manage a common secure data entry system for the 3 study sites and be responsible for generating the initial and subsequent (adaptive) randomizations that will be used by the Investigational Pharmacy at the University of Cincinnati to allocate the supplement to subjects at all 3 sites. He will be responsible for the randomization, setting up a common secure data entry system for the 3 proposed study sites (University of Cincinnati, Ohio State University, and the University of Kansas Medical Center) and for managing the adaptive design. With his Co-PIs, Carlson and Valentine, he will be primarily responsible for the statistical analysis for the study

Communication

We will set up a teleconference at least once per month (and Skype calls more frequently initially and as needed) to discuss any issues of recruitment and retention at any site. The teleconference will include the PIs (Valentine, Gajewski, Carlson) and the key personnel at UC, OSU and Nationwide (DeFranco, Cackovic Rogers) and study coordinators at each site (Kerling, Lehman, Armond). Because we have already conducted a nearly identical intervention at Kansas City, we do not anticipate a lot of new issues. We have developed a system and understand the workload engendered by the proposed study and so could be helpful in advising Dr. Valentine what will be needed for the two Ohio sites. The teleconferences will be used to review and provide updates on enrollment, testing, and data collection over the previous month provided by the leaders of each of the teams. Carlson, Valentine and Gajewski will communicate at least weekly through email or telephone conversation. Kansas City and Cincinnati are only ~90 minutes apart by air. The PIs will meet face-to-face 6 months into the recruitment and yearly or as needed after that. The PIs have already developed open channels of dialogue that are exercised frequently in the writing of this proposal. Bridge lines, video conferencing, e-mail and shared digital access systems are available as needed.

Conflict Resolution

Given that Valentine's, Carlson's and Gajewski's spheres of expertise are disparate (i.e., they have unique roles and knowledge they bring to this proposal and team), intellectual disagreements are unlikely to arise. Dr. Gajewski and Dr. Carlson have worked closely and effectively for the last 11 years. If there is uncertainty with respect to scientific issues, however, the PIs have enough common experience that they should be able to explain the matters at hand to one another. Even in the best collaborations, it is sometimes desirable to seek out external opinions to guide decisions. Again all PIs have significant experience with other teams that should carry over. In addition, Dr. Carlson has a long history of mentoring faculty who are earlier in their careers. Her primary interest is that this trial be completed to determine if the low DHA intake by US

adults, particularly women of childbearing age, is a factor in early preterm birth that can be mitigated by increasing DHA intake during pregnancy. She is not motivated by any new career goals.

Actual conflict is not anticipated given that both PIs have a long history of collaboration, cooperation, and compromise with other teams. However, should they occur, we expect that they will be resolved through a significant effort on the part of all 3 PIs with the conduct of the project's science maintained as the highest priority. If a dispute arises with no immediate resolution, an online or in person meeting of the entire executive research team for this project (Valentine, Gajewski, and Carlson) will be called, issues will be described and the committee will make a majority decision that will be fully binding. In the writing of the grant, we have also had significant input and support from Drs. Buhimschi (Dr. Cackovic as of November 2018) and Weiner, and our own team members (Rogers for Valentine, Colombo for Carlson) themselves experienced senior investigators. In the event it is difficult to reach a decision among the PIs, we would involve our full teams at all sites to resolve any difficult issues.

Data Access, Dissemination, and Authorship

The data generated by this project will be entered at the individual site into a system developed and managed by Dr. Gajewski's team in Biostatistics at KUMC. The data will be fully accessible to the other PIs at all times. KUMC maintains an elaborate and secure digital networks for storage and remote access of data through encrypted and password-protected servers. Typically, with longitudinal projects like this one, decisions as to how and when to publish empirical reports is a difficult one. To resolve this issue, the PIs will map out a preliminary dissemination plan that is principled yet flexible enough to allow for the clearest manner of presenting the results, and to determine the extent of authorship. This has been a satisfactory mechanism for Drs. Carlson and Gajewski (and Dr. John Colombo) to address the issue of publication without conflict in their ongoing R01 at the University of Kansas Medical Center and the University of Kansas.

Responsibilities and Management Plan

A flowchart for management of the proposed project is shown below. The chart is designed primarily to show the nature of the organization of the teams and the lines of communication rather than the status of individuals within the teams. Carlson will interface with a study coordinator at KUMC and Valentine with the study coordinators responsible for managing both the Cincinnati and Columbus, OH sites. Monthly conference calls will take place among the PIs to resolve any issues. These meetings may occur more frequently especially at the beginning of the trial. Dr. Valentine directs the oversight in Ohio and the Lab Technician (Augustine), who is responsible for fatty acid analysis at Nationwide Children's. The Investigational pharmacy at University Hospital will be the site for supplement distribution and return of unused capsules from all three study sites: KUMC, OSU and UC.

SITE TEAMS AND LINES OF COMMUNICATION FLOWCHART

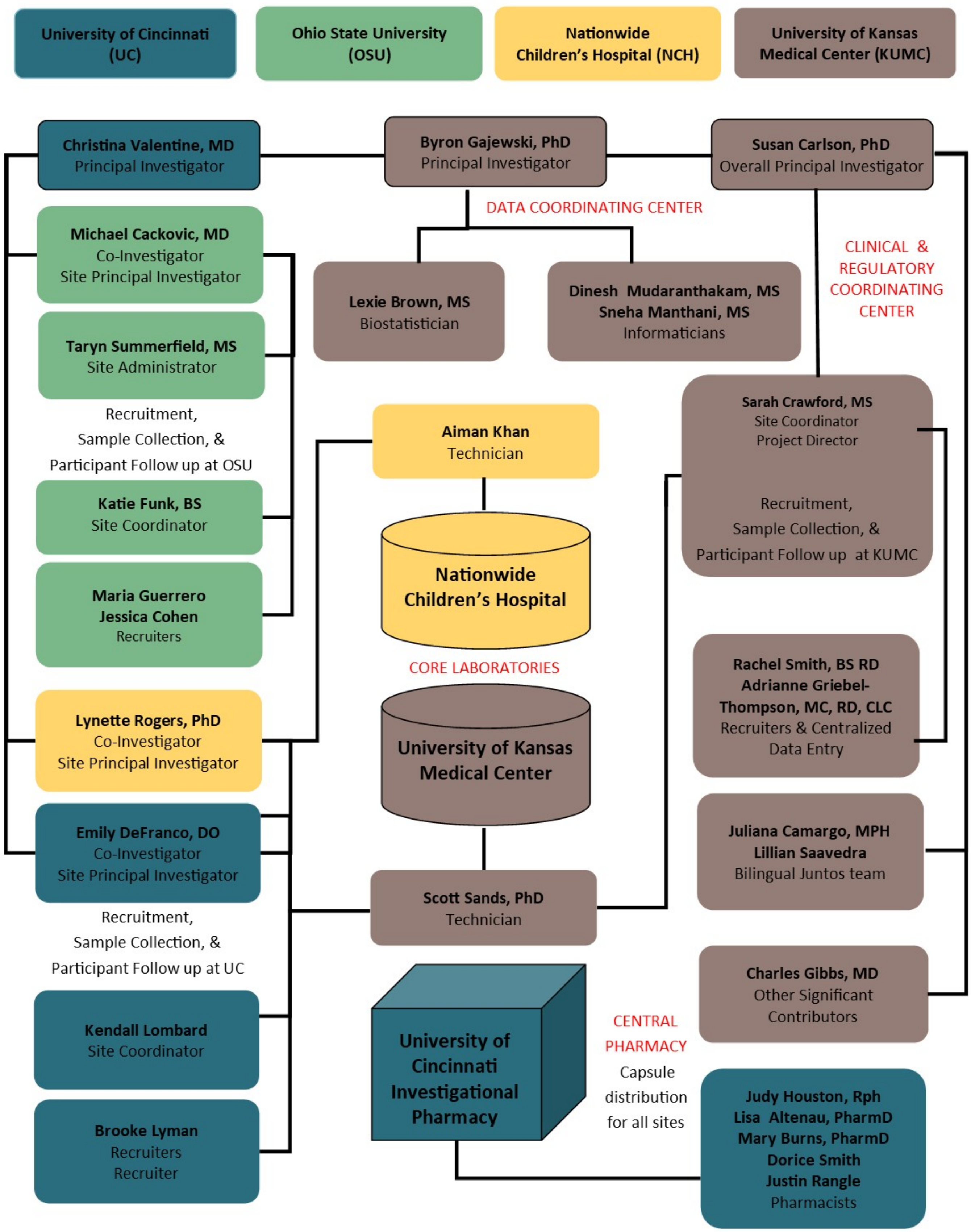

BIBLIOGRAPHY

1. Nesheim M, Yaktine, AL. Seafood Chioces Balancing Benefits and Risks. Washington, DC: The National Academies of Press; 2007.
2. Brenna JT, Lapillonne A. Background paper on fat and fatty acid requirements during pregnancy and lactation. Annals of nutrition & metabolism. 2009;55(1-3):97-122. Epub 2009/09/16. doi: 10.1159/000228998. PubMed PMID: 19752538.
3. Emken EA, Adlof RO, Gulley RM. Dietary linoleic acid influences desaturation and acylation of deuterium-labeled linoleic and linolenic acids in young adult males. Biochimica et biophysica acta. 1994;1213(3):277-88. Epub 1994/08/04. PubMed PMID: 7914092.
4. Burdge GC. Metabolism of alpha-linolenic acid in humans. Prostaglandins, leukotrienes, and essential fatty acids. 2006;75(3):161-8. Epub 2006/07/11. doi: 10.1016/j.plefa.2006.05.013. PubMed PMID: 16828546.
5. Carlson SE, Colombo J, Gajewski BJ, Gustafson KM, Mundy D, Yeast J, Georgieff MK, Markley LA, Kerling EH, Shaddy DJ. DHA supplementation and pregnancy outcomes. Am J Clin Nutr. 2013;97(4):808-15. Epub 2013/02/22. doi: 10.3945/ajcn.112.050021. PubMed PMID: 23426033 PMCID: PMCPmc3607655.
6. Parra MS, Schnaas L, Meydani M, Perroni E, Martinez S, Romieu I. Erythrocyte cell membrane phospholipid levels compared against reported dietary intakes of polyunsaturated fatty acids in pregnant Mexican women. Public health nutrition. 2002;5(6a):931-7. Epub 2003/03/14. doi: 10.1079/phn2002381. PubMed PMID: 12633518.
7. Yuhas R, Pramuk K, Lien EL. Human milk fatty acid composition from nine countries varies most in DHA. Lipids. 2006;41(9):851-8. Epub 2006/12/13. PubMed PMID: 17152922.
8. Gustafson KM, Carlson SE, Colombo J, Yeh HW, Shaddy DJ, Li S, Kerling EH. Effects of docosahexaenoic acid supplementation during pregnancy on fetal heart rate and variability: a randomized clinical trial. Prostaglandins, leukotrienes, and essential fatty acids. 2013;88(5):331-8. Epub 2013/02/26. doi: 10.1016/j.plefa.2013.01.009. PubMed PMID: 23433688 PMCID: PMCPmc3734850.
9. Smuts CM, Huang M, Mundy D, Plasse T, Major S, Carlson SE. A randomized trial of docosahexaenoic acid supplementation during the third trimester of pregnancy. Obstetrics and gynecology. 2003;101(3):469-79. Epub 2003/03/15. PubMed PMID: 12636950.
10. Lakin V, Haggarty P, Abramovich DR, Ashton J, Moffat CF, McNeill G, Danielian PJ, Grubb D. Dietary intake and tissue concentration of fatty acids in omnivore, vegetarian and diabetic pregnancy. Prostaglandins, leukotrienes, and essential fatty acids. 1998;59(3):209-20. Epub 1998/12/09. PubMed PMID: 9844995.
11. Otto SJ, van Houwelingen AC, Badart-Smook A, Hornstra G. Changes in the maternal essential fatty acid profile during early pregnancy and the relation of the profile to diet. The American journal of clinical nutrition. 2001;73(2):302-7. Epub 2001/02/07. PubMed PMID: 11157328.
12. Kuipers RS, Luxwolda MF, Sango WS, Kwesigabo G, Dijck-Brouwer DA, Muskiet FA. Postdelivery changes in maternal and infant erythrocyte fatty acids in 3 populations differing in fresh water fish intakes. Prostaglandins, leukotrienes, and essential fatty acids. 2011;85(6):387-97. Epub 2011/09/16. doi: 10.1016/j.plefa.2011.06.004. PubMed PMID: 21917436.
13. March of Dimes PMNCH, Save the Children, WHO;. Born Too Soon: The Global Action Report on Preterm Birth. Eds CP Howson, MV Kinney, JE Lawn. World Health Organization. Geneva: 2012.
14. Harper M. 454: Low maternal omega-3 levels prior to 22 weeks' gestation are associated with preterm delivery and low fish intake. American journal of obstetrics and gynecology. 2009;201(6, Supplement):S172. doi: http://dx.doi.org/10.1016/j.ajog.2009.10.620.
15. Valentine CJ, Morrow G, Fernandez S, Gulati P, Bartholomew D, Long D, Welty SE, Morrow AL, Rogers LK. Docosahexaenoic Acid and Amino Acid Contents in Pasteurized Donor Milk are Low for Preterm Infants. The Journal of pediatrics. 2010;157(6):906-10. Epub 2010/09/21. doi: 10.1016/j.jpeds.2010.06.017. PubMed PMID: 20850762.
16. Valentine CJ, Morrow G, Pennell M, Morrow AL, Hodge A, Haban-Bartz A, Collins K, Rogers LK. Randomized controlled trial of docosahexaenoic acid supplementation in midwestern U.S. human milk donors. Breastfeeding medicine : the official journal of the Academy of Breastfeeding Medicine. 2013;8(1):86-91. Epub 2012/05/10. doi: 10.1089/bfm.2011.0126. PubMed PMID: 22568471 PMCID: PMCPmc3566653.
17. Helland IB, Saarem K, Saugstad OD, Drevon CA. Fatty acid composition in maternal milk and plasma during supplementation with cod liver oil. European journal of clinical nutrition. 1998;52(11):839-45. Epub 1998/12/10. PubMed PMID: 9846598.
18. Connor C, McGinness M, Mammen J, Ranallo L, Lafaver S, Klemp J, Fan F, Mahnken J. Axillary reverse mapping: a prospective study in women with clinically node negative and node positive breast cancer. Annals of surgical oncology. 2013;20(10):3303-7. Epub 2013/08/27. doi: 10.1245/s10434-013-3113-4. PubMed PMID: 23975287.
19. Valentine CJ. Maternal dietary DHA supplementation to improve inflammatory outcomes in the preterm infant. Advances in nutrition (Bethesda, Md). 2012;3(3):370-6. Epub 2012/05/16. doi: 10.3945/an.111.001248. PubMed PMID: 22585914 PMCID: PMCPmc3649472.
20. Kromhout D, de Goede J. Update on cardiometabolic health effects of omega-3 fatty acids. Current opinion in lipidology.

2014;25(1):85-90. Epub 2013/12/19. doi: 10.1097/mol.0000000000000041. PubMed PMID: 24345990.
21. Harris WS, Dayspring TD, Moran TJ. Omega-3 fatty acids and cardiovascular disease: new developments and applications. Postgraduate medicine. 2013;125(6):100-13. Epub 2013/11/10. doi: 10.3810/pgm.2013.11.2717. PubMed PMID: 24200766.
22. Jiang W, Zhu Z, McGinley JN, El Bayoumy K, Manni A, Thompson HJ. Identification of a molecular signature underlying inhibition of mammary carcinoma growth by dietary N-3 fatty acids. Cancer research. 2012;72(15):3795-806. Epub 2012/06/02. doi: 10.1158/0008-5472.can-12-1047. PubMed PMID: 22651929.
23. MacLennan MB, Clarke SE, Perez K, Wood GA, Muller WJ, Kang JX, Ma DW. Mammary tumor development is directly inhibited by lifelong n-3 polyunsaturated fatty acids. The Journal of nutritional biochemistry. 2013;24(1):388-95. Epub 2012/10/03. doi: 10.1016/j.jnutbio.2012.08.002. PubMed PMID: 23026490.
24. Bougnoux P, Hajjaji N, Maheo K, Couet C, Chevalier S. Fatty acids and breast cancer: sensitization to treatments and prevention of metastatic re-growth. Progress in lipid research. 2010;49(1):76-86. Epub 2009/09/01. doi: 10.1016/j.plipres.2009.08.003. PubMed PMID: 19715726.
25. Lopez LB, Kritz-Silverstein D, Barrett Connor E. High dietary and plasma levels of the omega-3 fatty acid docosahexaenoic acid are associated with decreased dementia risk: the Rancho Bernardo study. The journal of nutrition, health & aging. 2011;15(1):25-31. Epub 2011/01/27. PubMed PMID: 21267518.
26. Zhao Y, Calon F, Julien C, Winkler JW, Petasis NA, Lukiw WJ, Bazan NG. Docosahexaenoic acid-derived neuroprotectin D1 induces neuronal survival via secretase- and PPARgamma-mediated mechanisms in Alzheimer's disease models. PloS one. 2011;6(1):e15816. Epub 2011/01/20. doi: 10.1371/journal.pone.0015816. PubMed PMID: 21246057 PMCID: PMCPmc3016440.
27. Yurko-Mauro K, McCarthy D, Rom D, Nelson EB, Ryan AS, Blackwell A, Salem N, Jr., Stedman M. Beneficial effects of docosahexaenoic acid on cognition in age-related cognitive decline. Alzheimer's & dementia : the journal of the Alzheimer's Association. 2010;6(6):456-64. Epub 2010/05/04. doi: 10.1016/j.jalz.2010.01.013. PubMed PMID: 20434961.
28. Hjorth E, Zhu M, Toro VC, Vedin I, Palmblad J, Cederholm T, Freund-Levi Y, Faxen-Irving G, Wahlund LO, Basun H, Eriksdotter M, Schultzberg M. Omega-3 fatty acids enhance phagocytosis of Alzheimer's disease-related amyloid-beta42 by human microglia and decrease inflammatory markers. Journal of Alzheimer's disease : JAD. 2013;35(4):697-713. Epub 2013/03/14. doi: 10.3233/jad-130131. PubMed PMID: 23481688.
29. Calder PC. n-3 fatty acids, inflammation and immunity: new mechanisms to explain old actions. The Proceedings of the Nutrition Society. 2013;72(3):326-36. Epub 2013/05/15. doi: 10.1017/s0029665113001031. PubMed PMID: 23668691.
30. Mas E, Croft KD, Zahra P, Barden A, Mori TA. Resolvins D1, D2, and other mediators of self-limited resolution of inflammation in human blood following n-3 fatty acid supplementation. Clinical chemistry. 2012;58(10):1476-84. Epub 2012/08/23. doi: 10.1373/clinchem.2012.190199. PubMed PMID: 22912397.
31. Ji RR, Xu ZZ, Strichartz G, Serhan CN. Emerging roles of resolvins in the resolution of inflammation and pain. Trends in neurosciences. 2011;34(11):599-609. Epub 2011/10/04. doi: 10.1016/j.tins.2011.08.005. PubMed PMID: 21963090 PMCID: PMCPmc3200462.
32. Hong SH, Belayev L, Khoutorova L, Obenaus A, Bazan NG. Docosahexaenoic acid confers enduring neuroprotection in experimental stroke. Journal of the neurological sciences. 2014;338(1-2):135-41. Epub 2014/01/18. doi: 10.1016/j.jns.2013.12.033. PubMed PMID: 24433927 PMCID: PMCPmc3943637.
33. Borre YE, Panagaki T, Koelink PJ, Morgan ME, Hendriksen H, Garssen J, Kraneveld AD, Olivier B, Oosting RS. Neuroprotective and cognitive enhancing effects of a multi-targeted food intervention in an animal model of neurodegeneration and depression. Neuropharmacology. 2014;79:738-49. Epub 2013/11/30. doi: 10.1016/j.neuropharm.2013.11.009. PubMed PMID: 24286859.
34. Colombo J, Carlson SE, Cheatham CL, Shaddy DJ, Kerling EH, Thodosoff JM, Gustafson KM, Brez C. Long-term effects of LCPUFA supplementation on childhood cognitive outcomes. The American journal of clinical nutrition. 2013;98(2):403-12. Epub 2013/06/28. doi: 10.3945/ajcn.112.040766. PubMed PMID: 23803884 PMCID: PMCPmc3712550.
35. Willatts P, Forsyth S, Agostoni C, Casaer P, Riva E, Boehm G. Effects of long-chain PUFA supplementation in infant formula on cognitive function in later childhood. The American journal of clinical nutrition. 2013;98(2):536s-42s. Epub 2013/06/21. doi: 10.3945/ajcn.112.038612. PubMed PMID: 23783296.
36. Jacobson JL, Jacobson SW, Muckle G, Kaplan-Estrin M, Ayotte P, Dewailly E. Beneficial effects of a polyunsaturated fatty acid on infant development: evidence from the inuit of arctic Quebec. The Journal of pediatrics. 2008;152(3):356-64. Epub 2008/02/19. doi: 10.1016/j.jpeds.2007.07.008. PubMed PMID: 18280840.
37. Helland IB, Smith L, Saarem K, Saugstad OD, Drevon CA. Maternal supplementation with very-long-chain n-3 fatty acids during pregnancy and lactation augments children's IQ at 4 years of age. Pediatrics. 2003;111(1):e39-44. Epub 2003/01/02. PubMed PMID: 12509593.
38. Birch EE, Khoury JC, Berseth CL, Castaneda YS, Couch JM, Bean J, Tamer R, Harris CL, Mitmesser SH, Scalabrin DM. The impact of early nutrition on incidence of allergic manifestations and common respiratory illnesses in children. The Journal of pediatrics. 2010;156(6):902-6, 6.e1. Epub 2010/03/17. doi: 10.1016/j.jpeds.2010.01.002. PubMed PMID: 20227721.
39. Miyake Y, Tanaka K, Okubo H, Sasaki S, Arakawa M. Maternal fat intake during pregnancy and wheeze and eczema in Japanese infants: the Kyushu Okinawa Maternal and Child Health Study. Annals of epidemiology. 2013;23(11):674-80. Epub

2013/10/08. doi: 10.1016/j.annepidem.2013.08.004. PubMed PMID: 24094480.
40. Montes R, Chisaguano AM, Castellote AI, Morales E, Sunyer J, Lopez-Sabater MC. Fatty-acid composition of maternal and umbilical cord plasma and early childhood atopic eczema in a Spanish cohort. European journal of clinical nutrition. 2013;67(6):658-63. Epub 2013/04/04. doi: 10.1038/ejcn.2013.68. PubMed PMID: 23549201.
41. Pike KC, Calder PC, Inskip HM, Robinson SM, Roberts GC, Cooper C, Godfrey KM, Lucas JS. Maternal plasma phosphatidylcholine fatty acids and atopy and wheeze in the offspring at age of 6 years. Clinical & developmental immunology. 2012;2012:474613. Epub 2012/10/11. doi: 10.1155/2012/474613. PubMed PMID: 23049600 PMCID: PMCPmc3463812.
42. Moon RJ, Harvey NC, Robinson SM, Ntani G, Davies JH, Inskip HM, Godfrey KM, Dennison EM, Calder PC, Cooper C. Maternal plasma polyunsaturated fatty acid status in late pregnancy is associated with offspring body composition in childhood. The Journal of clinical endocrinology and metabolism. 2013;98(1):299-307. Epub 2012/11/20. doi: 10.1210/jc.2012-2482. PubMed PMID: 23162098 PMCID: PMCPmc3604685.
43. Olsen SF, Hansen HS, Jensen B, Sorensen TI. Pregnancy duration and the ratio of long-chain n-3 fatty acids to arachidonic acid in erythrocytes from Faroese women. Journal of internal medicine Supplement. 1989;731:185-9. Epub 1989/01/01. PubMed PMID: 2706041.
44. Olsen SF, Sorensen JD, Secher NJ, Hedegaard M, Henriksen TB, Hansen HS, Grant A. Randomised controlled trial of effect of fish-oil supplementation on pregnancy duration. Lancet. 1992;339(8800):1003-7. Epub 1992/04/25. PubMed PMID: 1349049.
45. Makrides M, Duley L, Olsen SF. Marine oil, and other prostaglandin precursor, supplementation for pregnancy uncomplicated by pre-eclampsia or intrauterine growth restriction. The Cochrane database of systematic reviews. 2006(3):Cd003402. Epub 2006/07/21. doi: 10.1002/14651858.CD003402.pub2. PubMed PMID: 16856006.
46. Imhoff-Kunsch B, Briggs V, Goldenberg T, Ramakrishnan U. Effect of n-3 long-chain polyunsaturated fatty acid intake during pregnancy on maternal, infant, and child health outcomes: a systematic review. Paediatric and perinatal epidemiology. 2012;26 Suppl 1:91-107. Epub 2012/07/07. doi: 10.1111/j.1365-3016.2012.01292.x. PubMed PMID: 22742604.
47. Kar S, Wong M, Rogozinska E, Thangaratinam S. Effects of omega-3 fatty acids in prevention of early preterm delivery: a systematic review and meta-analysis of randomized studies. European journal of obstetrics, gynecology, and reproductive biology. 2016;198:40-6. Epub 2016/01/17. doi: 10.1016/j.ejogrb.2015.11.033. PubMed PMID: 26773247.
48. Makrides M, Gibson RA, McPhee AJ, Yelland L, Quinlivan J, Ryan P. Effect of DHA supplementation during pregnancy on maternal depression and neurodevelopment of young children: a randomized controlled trial. JAMA : the journal of the American Medical Association. 2010;304(15):1675-83. Epub 2010/10/21. doi: 10.1001/jama.2010.1507. PubMed PMID: 20959577.
49. Olsen SF, Secher NJ, Tabor A, Weber T, Walker JJ, Gluud C. Randomised clinical trials of fish oil supplementation in high risk pregnancies. Fish Oil Trials In Pregnancy (FOTIP) Team. BJOG : an international journal of obstetrics and gynaecology. 2000;107(3):382-95. Epub 2000/03/31. PubMed PMID: 10740336.
50. Harper M, Thom E, Klebanoff MA, Thorp J, Jr., Sorokin Y, Varner MW, Wapner RJ, Caritis SN, Iams JD, Carpenter MW, Peaceman AM, Mercer BM, Sciscione A, Rouse DJ, Ramin SM, Anderson GD. Omega-3 fatty acid supplementation to prevent recurrent preterm birth: a randomized controlled trial. Obstetrics and gynecology. 2010;115(2 Pt 1):234-42. Epub 2010/01/23. doi: 10.1097/AOG.0b013e3181cbd60e. PubMed PMID: 20093894.
51. Martin J, Hamilton, BE, Osterman, MJ, Curtin, SC, Mathers, TJ. Births: Final Data for 2012. National vital statistics reports; vol 62 no 9. Hyattsville, MD: National Center for Health Statistics 2013. Available from: http://www.cdc.gov/nchs/data/nvsr/nvsr62/nvsr62_09.pdf.
52. Schwartz SL, Gelfand AE, Miranda ML. Joint Bayesian analysis of birthweight and censored gestational age using finite mixture models. Statistics in medicine. 2010;29(16):1710-23. Epub 2010/06/25. doi: 10.1002/sim.3900. PubMed PMID: 20575047.
53. Lockwood CJ, Kuczynski E. Markers of risk for preterm delivery. Journal of perinatal medicine. 1999;27(1):5-20. Epub 1999/05/27. doi: 10.1515/jpm.1999.001. PubMed PMID: 10343929.
54. Buhimschi CS, Baumbusch MA, Dulay AT, Oliver EA, Lee S, Zhao G, Bhandari V, Ehrenkranz RA, Weiner CP, Madri JA, Buhimschi IA. Characterization of RAGE, HMGB1, and S100beta in inflammation-induced preterm birth and fetal tissue injury. The American journal of pathology. 2009;175(3):958-75. Epub 2009/08/15. doi: 10.2353/ajpath.2009.090156. PubMed PMID: 19679874 PMCID: PMCPmc2731116.
55. Weiner CP, Mason CW, Dong Y, Buhimschi IA, Swaan PW, Buhimschi CS. Human effector/initiator gene sets that regulate myometrial contractility during term and preterm labor. American journal of obstetrics and gynecology. 2010;202(5):474.e1-20. Epub 2010/05/11. doi: 10.1016/j.ajog.2010.02.034. PubMed PMID: 20452493 PMCID: PMCPmc2867841.
56. Defranco EA, Jacobs TS, Plunkett J, Chaudhari BP, Huettner PC, Muglia LJ. Placental pathologic aberrations in cases of familial idiopathic spontaneous preterm birth. Placenta. 2011;32(5):386-90. Epub 2011/03/29. doi: 10.1016/j.placenta.2011.02.010. PubMed PMID: 21440297.
57. Rizza S, Tesauro M, Cardillo C, Galli A, Iantorno M, Gigli F, Sbraccia P, Federici M, Quon MJ, Lauro D. Fish oil supplementation improves endothelial function in normoglycemic offspring of patients with type 2 diabetes. Atherosclerosis. 2009;206(2):569-74. Epub 2009/04/28. doi: 10.1016/j.atherosclerosis.2009.03.006. PubMed PMID: 19394939 PMCID: PMCPmc2772138.
58. Calder PC. The role of marine omega-3 (n-3) fatty acids in inflammatory processes, atherosclerosis and plaque stability.

Molecular nutrition & food research. 2012;56(7):1073-80. Epub 2012/07/05. doi: 10.1002/mnfr.201100710. PubMed PMID: 22760980.
59. Rogers LK, Graf AE, Bhatia A, Leonhart KL, Oza-Frank R. Associations between maternal and infant morbidities and sRAGE within the first week of life in extremely preterm infants. PloS one. 2013;8(12):e82537. Epub 2013/12/11. doi: 10.1371/journal.pone.0082537. PubMed PMID: 24324804 PMCID: PMCPmc3855742.
60. Rogers LK, Valentine CJ, Keim SA. DHA supplementation: current implications in pregnancy and childhood. Pharmacological research : the official journal of the Italian Pharmacological Society. 2013;70(1):13-9. Epub 2012/12/26. doi: 10.1016/j.phrs.2012.12.003. PubMed PMID: 23266567 PMCID: PMCPmc3602397.
61. Matesanz N, Park G, McAllister H, Leahey W, Devine A, McVeigh GE, Gardiner TA, McDonald DM. Docosahexaenoic acid improves the nitroso-redox balance and reduces VEGF-mediated angiogenic signaling in microvascular endothelial cells. Investigative ophthalmology & visual science. 2010;51(12):6815-25. Epub 2010/08/13. doi: 10.1167/iovs.10-5339. PubMed PMID: 20702831 PMCID: PMCPmc3055780.
62. Muglia LJ, Katz M. The enigma of spontaneous preterm birth. The New England journal of medicine. 2010;362(6):529-35. Epub 2010/02/12. doi: 10.1056/NEJMra0904308. PubMed PMID: 20147718.
63. Calder PC. Immunomodulation by omega-3 fatty acids. Prostaglandins, leukotrienes, and essential fatty acids. 2007;77(5-6):327-35. Epub 2007/11/23. doi: 10.1016/j.plefa.2007.10.015. PubMed PMID: 18032006.
64. Lindstrom TM, Bennett PR. The role of nuclear factor kappa B in human labour. Reproduction (Cambridge, England). 2005;130(5):569-81. Epub 2005/11/03. doi: 10.1530/rep.1.00197. PubMed PMID: 16264088.
65. Jung SB, Kwon SK, Kwon M, Nagar H, Jeon BH, Irani K, Yoon SH, Kim CS. Docosahexaenoic acid improves vascular function via up-regulation of SIRT1 expression in endothelial cells. Biochemical and biophysical research communications. 2013;437(1):114-9. Epub 2013/06/29. doi: 10.1016/j.bbrc.2013.06.049. PubMed PMID: 23806688.
66. Xue B, Yang Z, Wang X, Shi H. Omega-3 polyunsaturated fatty acids antagonize macrophage inflammation via activation of AMPK/SIRT1 pathway. PloS one. 2012;7(10):e45990. Epub 2012/10/17. doi: 10.1371/journal.pone.0045990. PubMed PMID: 23071533 PMCID: PMCPmc3465287.
67. Kim PY, Zhong M, Kim YS, Sanborn BM, Allen KG. Long chain polyunsaturated fatty acids alter oxytocin signaling and receptor density in cultured pregnant human myometrial smooth muscle cells. PloS one. 2012;7(7):e41708. Epub 2012/08/01. doi: 10.1371/journal.pone.0041708. PubMed PMID: 22848573 PMCID: PMCPmc3406084.
68. Stillwell W, Shaikh SR, Zerouga M, Siddiqui R, Wassall SR. Docosahexaenoic acid affects cell signaling by altering lipid rafts. Reproduction, nutrition, development. 2005;45(5):559-79. Epub 2005/09/29. doi: 10.1051/rnd:2005046. PubMed PMID: 16188208.
69. Calder PC. Omega-3 fatty acids and inflammatory processes. Nutrients. 2010;2(3):355-74. Epub 2010/03/01. doi: 10.3390/nu2030355. PubMed PMID: 22254027 PMCID: PMCPmc3257651.
70. Hong S, Gronert K, Devchand PR, Moussignac RL, Serhan CN. Novel docosatrienes and 17S-resolvins generated from docosahexaenoic acid in murine brain, human blood, and glial cells. Autacoids in anti-inflammation. The Journal of biological chemistry. 2003;278(17):14677-87. Epub 2003/02/19. doi: 10.1074/jbc.M300218200. PubMed PMID: 12590139.
71. Serhan CN, Hong S, Gronert K, Colgan SP, Devchand PR, Mirick G, Moussignac RL. Resolvins: a family of bioactive products of omega-3 fatty acid transformation circuits initiated by aspirin treatment that counter proinflammation signals. The Journal of experimental medicine. 2002;196(8):1025-37. Epub 2002/10/23. PubMed PMID: 12391014 PMCID: PMCPmc2194036.
72. Maskrey BH, Megson IL, Rossi AG, Whitfield PD. Emerging importance of omega-3 fatty acids in the innate immune response: molecular mechanisms and lipidomic strategies for their analysis. Molecular nutrition & food research. 2013;57(8):1390-400. Epub 2013/02/19. doi: 10.1002/mnfr.201200723. PubMed PMID: 23417926.
73. Serhan CN, Dalli J, Karamnov S, Choi A, Park CK, Xu ZZ, Ji RR, Zhu M, Petasis NA. Macrophage proresolving mediator maresin 1 stimulates tissue regeneration and controls pain. FASEB journal : official publication of the Federation of American Societies for Experimental Biology. 2012;26(4):1755-65. Epub 2012/01/19. doi: 10.1096/fj.11-201442. PubMed PMID: 22253477 PMCID: PMCPmc3316905.
74. Karim SM. Prostaglandins as abortifacients. The New England journal of medicine. 1971;285(27):1534-5. Epub 1971/12/30. doi: 10.1056/nejm197112302852712. PubMed PMID: 5127146.
75. Norwitz ER, Robinson JN, Challis JR. The control of labor. The New England journal of medicine. 1999;341(9):660-6. Epub 1999/08/26. doi: 10.1056/nejm199908263410906. PubMed PMID: 10460818.
76. Pietrantoni E, Del Chierico F, Rigon G, Vernocchi P, Salvatori G, Manco M, Signore F, Putignani L. Docosahexaenoic Acid Supplementation during Pregnancy: A Potential Tool to Prevent Membrane Rupture and Preterm Labor. International journal of molecular sciences. 2014;15(5):8024-36. Epub 2014/05/09. doi: 10.3390/ijms15058024. PubMed PMID: 24810692.
77. Velten M, Britt RD, Jr., Heyob KM, Tipple TE, Rogers LK. Maternal dietary docosahexaenoic acid supplementation attenuates fetal growth restriction and enhances pulmonary function in a newborn mouse model of perinatal inflammation. The Journal of nutrition. 2014;144(3):258-66. Epub 2014/01/24. doi: 10.3945/jn.113.179259. PubMed PMID: 24453131 PMCID: PMCPmc3927543.
78. Bastek JA, Brown AG, Foreman MN, McShea MA, Anglim LM, Adamczak JE, Elovitz MA. The soluble receptor for

advanced glycation end products can prospectively identify patients at greatest risk for preterm birth. The journal of maternal-fetal & neonatal medicine : the official journal of the European Association of Perinatal Medicine, the Federation of Asia and Oceania Perinatal Societies, the International Society of Perinatal Obstet. 2012;25(9):1762-8. Epub 2012/02/22. doi: 10.3109/14767058.2012.663825. PubMed PMID: 22339587.

79. Simopoulos AP, Leaf A, Salem N, Jr. Workshop statement on the essentiality of and recommended dietary intakes for Omega-6 and Omega-3 fatty acids. Prostaglandins, leukotrienes, and essential fatty acids. 2000;63(3):119-21. Epub 2000/09/19. doi: 10.1054/plef.2000.0176. PubMed PMID: 10991764.

80. Fats and oils in human nutrition. Report of a joint expert consultation. Food and Agriculture Organization of the United Nations and the World Health Organization. FAO food and nutrition paper. 1994;57:i-xix, 1-147. Epub 1994/01/01. PubMed PMID: 7641870.

81. Koletzko B, Cetin I, Brenna JT. Dietary fat intakes for pregnant and lactating women. The British journal of nutrition. 2007;98(5):873-7. Epub 2007/08/11. doi: 10.1017/s0007114507764747. PubMed PMID: 17688705.

82. Smuts CM, Borod E, Peeples JM, Carlson SE. High-DHA eggs: feasibility as a means to enhance circulating DHA in mother and infant. Lipids. 2003;38(4):407-14. Epub 2003/07/10. PubMed PMID: 12848286.

83. Ramakrishnan U, Stein AD, Parra-Cabrera S, Wang M, Imhoff-Kunsch B, Juarez-Marquez S, Rivera J, Martorell R. Effects of docosahexaenoic acid supplementation during pregnancy on gestational age and size at birth: randomized, double-blind, placebo-controlled trial in Mexico. Food and nutrition bulletin. 2010;31(2 Suppl):S108-16. Epub 2010/08/19. PubMed PMID: 20715595.

84. Mulder KA, King DJ, Innis SM. Omega-3 fatty acid deficiency in infants before birth identified using a randomized trial of maternal DHA supplementation in pregnancy. PloS one. 2014;9(1):e83764. Epub 2014/01/16. doi: 10.1371/journal.pone.0083764. PubMed PMID: 24427279 PMCID: PMCPmc3888379.

85. DeFranco EA, O'Brien JM, Adair CD, Lewis DF, Hall DR, Fusey S, Soma-Pillay P, Porter K, How H, Schakis R, Eller D, Trivedi Y, Vanburen G, Khandelwal M, Trofatter K, Vidyadhari D, Vijayaraghavan J, Weeks J, Dattel B, Newton E, Chazotte C, Valenzuela G, Calda P, Bsharat M, Creasy GW. Vaginal progesterone is associated with a decrease in risk for early preterm birth and improved neonatal outcome in women with a short cervix: a secondary analysis from a randomized, double-blind, placebo-controlled trial. Ultrasound in obstetrics & gynecology : the official journal of the International Society of Ultrasound in Obstetrics and Gynecology. 2007;30(5):697-705. Epub 2007/09/28. doi: 10.1002/uog.5159. PubMed PMID: 17899571.

86. Iams JD. Identification of candidates for progesterone: why, who, how, and when? Obstetrics and gynecology. 2014;123(6):1317-26. Epub 2014/05/09. doi: 10.1097/aog.0000000000000276. PubMed PMID: 24807317.

87. Klebanoff MA, Harper M, Lai Y, Thorp J, Jr., Sorokin Y, Varner MW, Wapner RJ, Caritis SN, Iams JD, Carpenter MW, Peaceman AM, Mercer BM, Sciscione A, Rouse DJ, Ramin SM, Anderson GD. Fish consumption, erythrocyte fatty acids, and preterm birth. Obstetrics and gynecology. 2011;117(5):1071-7. Epub 2011/04/22. doi: 10.1097/AOG.0b013e31821645dc. PubMed PMID: 21508745 PMCID: PMCPmc3754827.

88. Berry DA. Adaptive clinical trials: the promise and the caution. Journal of clinical oncology : official journal of the American Society of Clinical Oncology. 2011;29(6):606-9. Epub 2010/12/22. doi: 10.1200/jco.2010.32.2685. PubMed PMID: 21172875.

89. Berry S, Carlin, BP, Lee, JJ, Muller, P. Bayesian Adaptive Methods for Clinical Trials. Boca Raton, FL: CRC Press; 2011.

90. Patient Centered Outcomes Research Instistitue. PCORI Methodology Standards 2012 [cited 2013 June 17]. Available from: http://www.pcori.org/assets/PCORI-Methodology-Standards.pdf.

91. McIlwain J. Data Standards Harmonization: A look at the past, present, and future of interoperability across the clinical research spectrum: Advanstar Communications; May 1, 2010. Available from: http://www.appliedclinicaltrialsonline.com/appliedclinicaltrials/CRO%2FSponsor/Data-Standards-Harmonization/ArticleStandard/Article/detail/668519.

92. Nutrition Coordinating Center. Information for Grant Writers 2016 [cited 2016 November 01]. Available from: http://www.ncc.umn.edu/products/information-for-grant-writers/.

93. Kuratko C. Food-frequency questionnaire for assessing long-chain omega-3 fatty-acid intake: Re: Assessing long-chain omega-3 polyunsaturated fatty acids: a tailored food-frequency questionnaire is better. Nutrition (Burbank, Los Angeles County, Calif). 2013;29(5):807-8. Epub 2013/04/16. doi: 10.1016/j.nut.2012.10.013. PubMed PMID: 23582079.

94. Gibbs HD, Ellerbeck EF, Befort C, Gajewski B, Kennett AR, Yu Q, Christifano D, Sullivan DK. Measuring Nutrition Literacy in Breast Cancer Patients: Development of a Novel Instrument. Journal of cancer education : the official journal of the American Association for Cancer Education. 2016;31(3):493-9. Epub 2015/05/09. doi: 10.1007/s13187-015-0851-y. PubMed PMID: 25952941 PMCID: PMCPmc4639469.

95. Balcazar H, Krull JL, Peterson G. Acculturation and family functioning are related to health risks among pregnant Mexican American women. Behavioral medicine (Washington, DC). 2001;27(2):62-70. Epub 2002/01/05. doi: 10.1080/08964280109595772. PubMed PMID: 11763826.

96. Perez-Escamilla R, Song DS, Taylor CA, Mejia A, Melgar-Quinonez H, Balcazar HG, Anders RL, Segura-Perez S, Duarte-Gardea MO, Ibarra JM. Place of residence modifies the association between acculturation and dietary tools knowledge among Latina WIC participants: a multi-state study. Journal of immigrant and minority health. 2011;13(2):299-308. Epub 2010/06/16. doi: 10.1007/s10903-010-9360-6. PubMed PMID: 20549357 PMCID: PMCPmc3050110.

97. USDA Economic Research Service. U.S. Household Food Security Survey Module 2012 [cited 2017 April 5th]. Available from: https://www.ers.usda.gov/topics/food-nutrition-assistance/food-security-in-the-us/survey-tools/#household.

98. Shah MK, Kieffer EC, Choi H, Schumann C, Heisler M. Mediators and Moderators of the Effectiveness of a Community Health Worker Intervention That Improved Dietary Outcomes in Pregnant Latino Women. Health education & behavior : the official publication of the Society for Public Health Education. 2015;42(5):593-603. Epub 2015/02/01. doi: 10.1177/1090198114568307. PubMed PMID: 25636316 PMCID: PMCPmc4520781.

99. Centers for Disease Control and Prevention. Healthy Days Core Module (CDC HRQOL-4) 1993 [cited 2017 April 5th]. Available from: https://www.cdc.gov/hrqol/hrqol14_measure.htm#1.

100. International Conference of Harmonisation of Technical Requirements for Registration of Pharmaceuticals for Human Use, Guidelines for Good Clinical Practice 1996. Available from: http://www.ich.org/fileadmin/Public_Web_Site/ICH_Products/Guidelines/Efficacy/E6_R1/Step4/E6_R1__Guideline.pdf.

101. Moher D, Hopewell S, Schulz KF, Montori V, Gotzsche PC, Devereaux PJ, Elbourne D, Egger M, Altman DG. CONSORT 2010 explanation and elaboration: updated guidelines for reporting parallel group randomised trials. BMJ (Clinical research ed). 2010;340:c869. Epub 2010/03/25. doi: 10.1136/bmj.c869. PubMed PMID: 20332511 PMCID: PMCPmc2844943.
