# Supplementary material for: Genotype-informed docosahexaenoic acid dosing based on the FADS Indel to reduce preterm and prolonged gestation: A secondary analysis of the ADORE randomized trial
Source: PLoS Med. 2026 Oct 8;23(10):e1005274. doi: 10.1371/journal.pmed.1005274 (PMC13660990; doi:10.1371/journal.pmed.1005274)
Supplement: Supplementary file 3 [file pmed.1005274.s016.pdf]

Assessment of DHA on Early Preterm birth: Statistical Analysis Plan

PIs: Carlson, Valentine, Gajewski

April 30, 2014

July 6, 2020: Modifications Made During the Study

1. Table of Contents

2. Introduction and Aims 3

3. Aim 1: Determine if a supplement of 1000 mg DHA /day compared to 200 mg DHA/day during the last two trimesters of pregnancy can reduce ePTB (*primary efficacy analysis*) 3

3.1. Primary Endpoint 3

3.2. Treatment Arms 3

3.3. Statistical Modeling 3

3.4. Final Endpoint Model 4

3.4.1. Evaluation of Posterior Estimates 4

3.4.2. Quantities of Interest 4

3.4.3. Target Doses 4

3.4.4. Decision Quantities 5

3.5. Conventions for Missing Data 5

3.6. Study Design 5

3.7. Timing of Interim Analyses 5

3.8. Response Adaptive Randomization 5

3.9. Criteria for Stopping Accrual 6

3.9.1. Stopping for Expected Futility 6

3.9.2. Stopping for Expected Success 6

3.10. Final Evaluation Criteria 6

3.11. Simulation Scenarios 6

3.12. Virtual Subject Response Profiles 6

3.13. Accrual Profiles 7

3.14. Dropout Profiles 7

3.15. Operating Characteristics 7

3.16. [Modifications to Primary Plan, April 7, 2020] Operating Characteristics 8

In March of 2020 the trial suspended enrollment due to the COVID-19 pandemic. Since the trial was so close to finishing its final year of funding and we had enrolled n=1100 participants it was decided to cease enrollment here and perform follow-up. 8

4. Aim 2: To conduct a pregnancy efficacy analysis controlled for potential predictor variables. 9

5. Aim 3: Determine if DHA intake affects plasma soluble (s) RAGE concentration. 12

6. Aim 4: Evaluate the occurrence of adverse events in women and infants in the experimental (1000 mg DHA) and control (200 mg DHA) groups 12

Definitions of an Adverse Event. An adverse event (AE) is any reaction, side effect or other undesirable event that occurs in conjunction with the use of the test product, whether or not the event is directly related to the test product. New and/or worsening signs and symptoms of underlying or emerging disease will be recorded as an adverse event if they might be clinically or scientifically related to DHA intake. Additionally, any patient complaint reported as possibly related to treatment will be recorded as an adverse event. 13

Definition of a Serious Adverse Event. Any adverse event that results in the following is considered a serious adverse event (SAE): 1) death; 2) a life-threatening event; 3) inpatient hospitalization or prolonging of an existing hospitalization; 4) a persistent or significant disability/incapacity or 5) a congenital anomaly/birth defect. Medical events that may not result in death, be life-threatening, or require hospitalization may be considered an SAE when, based upon appropriate medical judgment, they may jeopardize the patient and may require medical or surgical intervention to prevent one of the outcomes listed above. 13

6.1. Bayesian Monitoring 14

6.2. Final Bayesian Hierarchical Monitoring (w/contributions from Yang Wang) 17

Priors and Likelihood Functions 17

Simulation results for Example Data 17

7. Secondary Analyses for Aim 1: 23

7.1. Determine if a supplement of 1000 mg DHA /day compared to 200 mg DHA/day during the last two trimesters of pregnancy can reduce ePTB 23

7.2. Determine if a supplement of 1000 mg DHA /day compared to 200 mg DHA/day during the last two trimesters of pregnancy can reduce ePTB but varies across (RBC-PL-DHA) at Enrollment (<6% versus >= 6%) (TTE Model help by Fengming Tang) 24

7.3. Determine if a supplement of 1000 mg DHA /day compared to 200 mg DHA/day during the last two trimesters of pregnancy can reduce ePTB but varies across time ("drift analysis") (TTE Model help by Fengming Tang) 25

7.4. Determine if a supplement of 1000 mg DHA /day compared to 200 mg DHA/day during the last two trimesters of pregnancy can reduce ePTB but varies across time ("compliance model") 26

8. Secondary Analyses for Aim 4: NICU Stays Across Arms 27

2. Introduction and Aims

This statistical analysis plan (SAP) is for the ADORE trial and describes it as a phase III clinical trial adaptive design to Aim 1) determine if a supplement of 1000 mg DHA /day compared to 200 mg DHA/day during the last two trimesters of pregnancy can reduce ePTB (*primary efficacy analysis*). Other goals of this phase III study is to Aim 2) To conduct a pregnancy efficacy analysis controlled for potential predictor variables; Aim 3) determine if DHA intake affects plasma soluble (s) RAGE concentration; and Aim 4) evaluate the occurrence of adverse events in women and infants in the experimental (1000 mg DHA) and control (200 mg DHA) groups.

This document describes the features of the primary analysis for aim 1 including simulated design, the statistical models, decision rules, and simulation scenarios as input into the FACTS (Fixed and Adaptive Clinical Trial Simulator) software. A small set of operating characteristics for the simulations is also summarized.

3. Aim 1: Determine if a supplement of 1000 mg DHA /day compared to 200 mg DHA/day during the last two trimesters of pregnancy can reduce ePTB (*primary efficacy analysis*)

3.1. Primary Endpoint

The primary endpoint is Dichotomous (<34 weeks gestation age at birth), an assumption for the simulation is that this is measured at 26 weeks after enrollment. Lower response corresponds to subject improvement.

3.2. Treatment Arms

The trial will enroll up to a maximum of 1200 births, randomized among 2 arms. We have 2 treatment arms which we label generically by their arm index as: $d = 1, 2$. The first arm is 200 mg/day of DHA and the second is 1,000 mg/day of DHA.

3.3. Statistical Modeling

This section describes the statistical modeling used in the design. The modeling is Bayesian in nature.

3.4. Final Endpoint Model

Let Y_i be the primary outcome measured at 26 weeks for the ith subject. We model the outcomes as

$$Y_i \sim \text{Bernoulli}(P_{d_i})$$

where P_d is the underlying response rate for arm d. We transform the response rates onto the log-odds scale to allow modeling on a continuous scale:

$$\theta_d = \log\left(\frac{P_d}{1 - P_d}\right).$$

The mean response is modeled independently for each dose as:

$$\theta_1 \sim N(-3.4761, 1.5^2),$$

$$\theta_2 \sim N(-3.4761, 1.5^2).$$

Thus, θ_d for each dose is estimated separately using only data from that dose.

3.4.1. Evaluation of Posterior Estimates

The Bayesian final endpoint model is fitted to the data at each update. The posterior is calculated as:

$$p(\omega|Y) \propto \prod_{i=1}^{n} p\,(y_i|\varphi)p(\varphi)$$

where φ is the set of parameters for the final endpoint model, $p(\varphi)$ is the prior for those parameters, y_i is the final response for each subject, and n is the number of subjects. The posterior is evaluated using MCMC with individual parameters updated by Metropolis Hastings (or Gibbs sampling where possible), using only the y_i data available at the time of the update.

3.4.2. Quantities of Interest

We define a number of quantities that will be tracked and may be used to make decisions during the trial.

3.4.3. Target Doses

We define the following target dose:

- The maximum effective dose (d_{max}) is the dose with the greatest treatment effect (difference from control). For each dose, we calculate the probability of being the d_{max}:

$$Pr(Max).$$

3.4.4. Decision Quantities

Throughout the trial, decisions may be based on the following quantities:

- $Pr(Max)$ for $d =$ greatest $Pr(Max)$

3.5. Conventions for Missing Data

For any subject whose final endpoint is unknown due to drop out, the final outcome will be multiply imputed from the Bayesian model.

3.6. Study Design

3.7. Timing of Interim Analyses

The first interim will occur after 300 subjects have enrolled into the trial. Subsequent interims will be conducted every 13 weeks and will continue beyond full accrual. Since interims are defined by calendar time, the total number of planned interims, I, is random and will depend on the rate at which subjects accrue to the trial.

3.8. Response Adaptive Randomization

There will be an initial burn-in period in which subjects are allocated with ratio 150:150 among the arms. After this initial burn-in period, adaptive randomization will begin, with the goal of preferentially allocating subjects to the doses that appear more promising.
For the remaining doses, we recompute the randomization probabilities as follows. First we calculate:

The response adaptive allocation uses the following weights:

$$V_d = \left[\frac{Pr(Max)Var(\theta_d)}{n_d + 1}\right]^{1/4}$$

(where $Var(\theta_d)$ is the posterior variance of the mean response and n_d is the current number of subjects allocated to dose d).

The randomization probabilities for the adaptively allocated arms will be updated at each interim. They will be weighted according to the V_d and the weights will be renormalized to sum to 1.

To avoid assigning subjects to a dose with a minimal chance of being the best dose, any probability less than 0.05 is set to zero at that interim and the resulting probability is reallocated among the remaining doses. In this manner, a dose may be temporarily dropped but may be re-introduced if the adaptive randomization probability increases at subsequent interims.

3.9. Criteria for Stopping Accrual

3.9.1. Stopping for Expected Futility

No early stopping criteria for futility have been defined for this trial.

3.9.2. Stopping for Expected Success

For interim 1-I, the trial may stop accrual for expected success if all of the following criteria are satisfied:

- $Pr(Max) > 0.99$ for $d =$ greatest $Pr(Max)$

If a success stopping rule is met at an interim analysis, then a final analysis will be conducted after all currently enrolled subjects have been followed to their final endpoint.

3.10. Final Evaluation Criteria

At the final analysis, the trial will be considered successful if all of the following criteria are satisfied:

- $Pr(Max) > 0.99$ for $d =$ greatest $Pr(Max)$

No final futility criteria have been defined for this trial.

3.11. Simulation Scenarios

We evaluate the proposed design through trial simulation. We hypothesize several possible underlying truths for the mean response, as well as for trial execution variables such as accrual and dropout. For each of these scenarios, we generate data according to those truths and run through the design as specified above. We repeat this process to create multiple ``virtual trials" and we track the behavior of each trial. In this section, we describe the the parameters used to generate the virtual subject-level data.

3.12. Virtual Subject Response Profiles

We consider 5 profiles for which subject outcomes for the final endpoint are simulated to have response rates as shown in Table-1.

Table-1: Virtual subject response rates

VSR	d_1	d_2
Expected effect 3 .5	0.03	0.005
Expected effect 3 1	0.03	0.01
Expected effect 3 2	0.03	0.02
Expected effect 4 1	0.04	0.01
No effect .03	0.03	0.03

3.13. Accrual Profiles

We simulate the random arrival of subjects into the trial from a Poisson process with the mean weekly rates specified in Table-2. Within each accrual profile, there may be differential recruitment rates over time and across regions. Thus for each region, we specify:

- the mean number of subjects per week at peak accrual,
- the start date (in weeks from the start of the trial),
- whether the region will have a ramp up phase, and if so, when the ramp up will be complete, and
- whether the region will have a ramp down phase, and if so, when the ramp down will begin and when it will be complete.
  Ramp up and ramp down define simple linear increases and decreases in the mean recruitment rate from the start to the end of the ramp. Thus some simulated trials recruit more quickly than this and some more slowly.

Table-2: Accrual Profiles

Profile Name	Region Index	Peak Rate	Start Week	Ramp Up	Ramp up Complete	Ramp Down	Start Ramp Down	Ramp Down Complete
Accrual 9 per week total	1	6	0	NA	NA	NA	NA	NA

3.14. Dropout Profiles

We assume no dropouts for the purpose of this simulation.

3.15. Operating Characteristics

Power, Sample Size, Trial Duration, and Allocation. For the purposes of this investigation we looked at several virtual (or "pretend") responses to determine the power, sample size, time (duration), and subject allocation needed for our study. We created several scenarios for ePTB rates. We performed five sets of trial simulations based on the various combinations of response shown in Table 2. Each set involved 100 trial simulations. We highlight two scenarios. The first uses a slightly more conservative result than the KUDOS trial to predict what we believe is the most likely response **(scenario #1 in Table 2)**. If there is a best group in terms of ePTB rate, we estimated (identified) that 82% of the simulated trials had early success and 8% had late success. This trial scenario had 90% power and the sample size of this trial scenario was on average 938 (60% of these in the winning group). The average length of this trial scenario was 184 weeks. While a

conventional equal randomization trial would have 90% power, it would be larger (1200 subjects), slower (230 weeks), and have a lower rate of subjects on the winning group (50%). The second is the highly unlikely scenario that serves as our null hypothesis **(scenario #5 in Table 3).** In this scenario there is no difference in ePTB between the groups. Therefore, the extent to which this scenario is "successful" actually reflects our Type I error rate. For this scenario, we estimated (identified) that 5% of the simulated trials had early success, 0% late success. Thus this trial scenario produced an appropriate expected Type I error (α=5%). The sample size of this scenario on average was 1188 subjects (equally allocated across groups). The average length of the trials under this scenario was 231 weeks.

Table 3. Simulated Trial Operating Characteristics.

Scenario	%. Finish Early	%. Finish Late	Power	Mean Subjects	%Group 1	%Group 2	Mean Trial (Weeks)
#1. very likely (4 vs 1%)*	82%	8%	90%	938	40%	60%	184
#2. likely (3 vs 0.5%)	84%	7%	91%	934	41%	59%	184
#3. unlikely (3 vs 1%)	52%	11%	63%	1046	44%	56%	204
#4. very unlikely (3 vs 2%)	23%	4%	27%	1142	46%	54%	221
#5. no difference (3 vs 3%)	5%	0%	5%	1188	51%	49%	231

*Based on our planned enrollment and US 2012 ePTB rates of black and white pregnancies, we anticipate 4.1% ePTB in the control group.

3.16. [Modifications to Primary Plan, April 7, 2020] Operating Characteristics

In March of 2020 the trial suspended enrollment due to the COVID-19 pandemic. Since the trial was so close to finishing its final year of funding and we had enrolled n=1100 participants it was decided to cease enrollment here and perform follow-up.

The changes are a revision assuming 1100 enrolled with 5% dropout.

Power, Sample Size, Trial Duration, and Allocation. For the purposes of this investigation we looked at several virtual (or "pretend") responses to determine the power, sample size, time (duration), and subject allocation needed for our study. We created

several scenarios for ePTB rates. We performed five sets of trial simulations based on the various combinations of response shown in Table 4. Each set involved 1000 trial simulations. We highlight two scenarios. The first uses a slightly more conservative result than the KUDOS trial to predict what we believe is the most likely response **(scenario #1 in Table 4)**. If there is a best group in terms of ePTB rate, we estimated (identified) that 85% of the simulated trials had early success and 2% had late success. This trial scenario had 87% power and the sample size of this trial scenario was on average 927 (59% of these in the winning group). The average length of this trial scenario was 180 weeks. While a conventional equal randomization trial would have 89% power, it would be larger (1100 subjects), slower (209 weeks), and have a lower rate of subjects on the winning group (50%). The second is the highly unlikely scenario that serves as our null hypothesis **(scenario #5 in Table).** In this scenario there is no difference in ePTB between the groups. Therefore, the extent to which this scenario is "successful" actually reflects our Type I error rate. For this scenario, we estimated (identified) that 5% of the simulated trials had early success, 0% late success. Thus this trial scenario produced an appropriate expected Type I error (α=5%). The sample size of this scenario on average was 1092 subjects (equally allocated across groups). The average length of the trials under this scenario was 208 weeks.

Table 4. Simulated Trial Operating Characteristics.

Scenario	%. Finish Early	%. Finish Late	Power	Mean Subjects	%Group 1	%Group 2	Mean Trial (Weeks)
#1. very likely (4 vs 1%)*	85%	2%	87%	927	41%	59%	180
#2. likely (3 vs 0.5%)	85%	2%	87%	927	42%	58%	180
#3. unlikely (3 vs 1%)	57%	2%	59%	1002	44%	56%	193
#4. very unlikely (3 vs 2%)	14%	1%	15%	1078	47%	53%	206
#5. no difference (3 vs 3%)	5%	0%	5%	1092	50%	50%	208

*Based on our planned enrollment and US 2012 ePTB rates of black and white pregnancies, we anticipate 4.1% ePTB in the control group.

4. Aim 2: To conduct a pregnancy efficacy analysis controlled for potential predictor variables.

Secondary Efficacy Outcomes

- VLBW (<1500 g) and low birth weight (<2500 g) as recorded in hospital record
- Subject DHA status (RBC-PL-DHA) at enrollment and birth; fetal DHA status at birth from cord blood
- Gestational age (days) at delivery based on EDD in clinic record recorded following ultrasound on or before ~14 wk gestation
- Birth weight (g), length (cm) and head circumference (cm) at delivery as recorded in hospital record
- Pre-term birth (<37 weeks) based on ACOG guidelines
- Pregnancy outcomes: gestational diabetes, pre-eclampsia, C-section, spontaneous or induced labor, occurrence and reason for non-routine hospitalization

This phase of analysis (secondary) uses Bayesian multiple logistic and continuous regression for detailed investigation as to why there are differences between groups.

Pregnancy Efficacy Analysis According to Intent-to-treat Principles. In the first phase of analysis, we tested the differences between groups for the primary efficacy outcome (ePTB). The approach is repeated for secondary outcomes using Bayesian logistic and continuous regression (VLBW (<1.5 kg), maternal RBC PL DHA) with no covariates.

Pregnancy Efficacy Analysis Controlled for Potential Predictor Variables. The goal here is to find out how well DHA supplementation predicts our primary and secondary efficacy outcomes after controlling for potential predictor variables. In the second phase of analysis, using multiple linear regression (logistic and continuous), we explore the relationships among predictor variables for regression and all primary and secondary pregnancy outcomes. The predictor variables represent five general classes: overall DHA intake, diet (intake of nutrients and foods analyzed by principal component analysis), environment, subject demographics, and maternal medical history. For exploratory purposes, the most important relationship is between the DHA dose and pregnancy outcomes. Notice that instead of a grouping variable representing groups, we utilize DHA in the form of several predictor variables, depending on their source.

- A final exploratory analysis investigates the impact of capsule intake on outcome as mediated by maternal RBC-PL DHA. Using a reasonable set of predictor variables from the regressions above, we will run two sets of regressions for each outcome variable. This will allow maternal RBC DHA to be a mediator. First we regress the RBC-PL DHA level on all appropriate predictor variables (as above). Then we will run a regression of outcome variables on RBC-PL DHA level and all other appropriate predictor variables (as above but with RBC DHA added). In this way, we are running a path analytic model where we can obtain direct effects of variables and indirect effects of variables through mediator plasma level.
- In all regression analyses we will investigate local model adequacy by exploring standardized residuals and leverage points via Cook's distance. Possible co-linearity among predictor variables will examined with Pearson's correlation coefficient and

variance inflation factors (VIF). Scatter plots and histograms will also be used to investigate the adequacy of the model assumptions.

- Of substantive interest in the regression analysis is that race is one of the predictor variables. Since we anticipate 22.5% of the subjects to be of African descent, we can test whether efficacy of pregnancy outcomes is different for women of African descent relative to other races.
- For regression analysis purposes, the pregnancy outcomes are separated into two classes of variables, either continuous or dichotomous. For the continuous variables, Bayesian regression based on the normal distribution will be utilized. For the dichotomous variables, logistic regression will be utilized.
- For all outcomes, we set the DHA dose as a predictor variable and then fit all possible subsets of the other predictor variables to explore, for the particular pregnancy outcome, which model is the best. We will utilize a global fit index called Deviance Information Criteria (DIC) to determine which variables to keep in the final model. The DIC is very general and can be used for normal and logistic regression analyses.

Potential Predictor Variables for Regression Analysis

Class 1: Overall DHA intake

- DHA dose (capsules taken multiplied by the DHA in the type of capsule consumed)
- maternal RBC-PL DHA level at enrollment and delivery (g DHA per 100 g total fatty acids) by chromatographic analysis (C.2.e)
- cord RBC DHA at birth by chromatographic analysis (C.2.e)
- estimated DHA intake at enrollment from DHA FFQ, and frequency/amount of consumption of food and supplement sources containing DHA (see C.2.f)

Class 2: Diet

- estimated DHA intake at enrollment from DHA FFQ, and frequency/amount of consumption of food and supplement sources containing DHA (see C.2.f)
- intake of other nutrients or foods, e.g., macronutrient quantity or quality, micronutrient quantity at enrollment

Class 3: Exposure to environment

- tobacco exposure prior to and during pregnancy by subject report
- alcohol intake prior to and during pregnancy by subject report defined as standard drinks/day (Nutrition Educators of Health Professionals Teaching Tool)
- measurement of endocrine disrupting chemicals (EDCs) in urine at 12-20 weeks and during the 3rd trimester.

Class 4: Subject demographics

- marital status by subject report
- household income by subject report
- insurance type (private, public, uninsured) by review of clinic/hospital record
- maternal and paternal education by subject report
- maternal age at enrollment (years) from DOB listed in clinic/hospital record

- maternal and paternal race/ethnicity from clinic record or subject report
- fetal sex

Class 5: Maternal medical history

- BMI calculated from self-reported pre-pregnancy weight or measured 1st prenatal clinic weight record and measured height
- gestational weight gain (last clinic visit in pounds minus pre-pregnancy weight or 1st measured weight)
- gestational age at enrollment (days) calculated from EDD (based on ACOG guidelines)
- reproductive history
- characteristics of previous pregnancies (early preterm birth, pre-eclampsia, gestational diabetes)
- blood pressure throughout pregnancy
- iron status / hemoglobin at enrollment and mid-pregnancy
- cervical length between 18-22 weeks gestation
- estimated blood loss at delivery
- infant APGAR scores
- meconium in amniotic fluid
- evidence of illicit drug use from clinic/hospital record

5. Aim 3: Determine if DHA intake affects plasma soluble (s) RAGE concentration.

sRAGE is a competitive inhibitor of RAGE and is increased in some inflammatory states (chorioamnionitis, LPS-administration). DHA attenuates the increase in sRAGE concentration in LPS-induced inflammation in murine models. We propose to determine if the higher DHA supplementation influences either maternal or cord blood sRAGE as a possible mechanism.

sRAGE analysis. sRAGE will be determined at Nationwide Children's Hospital in Columbus, OH on batched samples using ELISA-based format (MesoScale Discovery, Rockville MD) according to the protocols of the manufacturer.

A continuous Bayesian model will test sRAGE differences between groups.

6. Aim 4: Evaluate the occurrence of adverse events in women and infants in the experimental (1000 mg DHA) and control (200 mg DHA) groups

Adverse Events Analyses. Fisher's exact test will be used to compare the incidence of maternal and infant adverse events between treatment groups. All *p* values will be evaluated at the $\alpha = 0.05$ level. No adjustment for multiple comparisons will be made. In

order to incorporate Bayesian modeling and reduce the false discovery of signals please see the alternatives to Fisher's exact testing below.

Adverse Events and Safety Monitoring

Definitions of an Adverse Event. An adverse event (AE) is any reaction, side effect or other undesirable event that occurs in conjunction with the use of the test product, whether or not the event is directly related to the test product. New and/or worsening signs and symptoms of underlying or emerging disease will be recorded as an adverse event if they might be clinically or scientifically related to DHA intake. Additionally, any patient complaint reported as possibly related to treatment will be recorded as an adverse event.

Definition of a Serious Adverse Event. Any adverse event that results in the following is considered a serious adverse event (SAE): 1) death; 2) a life-threatening event; 3) inpatient hospitalization or prolonging of an existing hospitalization; 4) a persistent or significant disability/incapacity or 5) a congenital anomaly/birth defect. Medical events that may not result in death, be life-threatening, or require hospitalization may be considered an SAE when, based upon appropriate medical judgment, they may jeopardize the patient and may require medical or surgical intervention to prevent one of the outcomes listed above.

Categories of Adverse Events. Adverse event collection is limited to four categories. 1) SAEs, 2) patient-reported complaints reported as possibly or definitely related to treatment, 3) sign and symptoms that might be clinically or scientifically related to DHA-intake and 4) other important medical events that do not result in SAE, but may jeopardize the participant and may require medical or surgical intervention to prevent an SAE. Events that are common to pregnancy or lack clinical/scientific significance to DHA will not be recorded* *(change in protocol a result of May 2017 decision by PI team, DSMB and medical monitor. Approved by IRB in version 0.11).

Timeline of Adverse Event Collection and Review. Adverse events, that meet one of the four categories in C.7.c will be collected from enrollment through 30-days postpartum, or until the infant is discharged from the hospital. AEs will be identified from medical records and by periodic phone interview when the study coordinator calls to check for supplement compliance and tolerance. All adverse events will be documented in eResearch and reviewed by site or trial PIs weekly. Only site and trial PIs will assign attribution. Immediate attention will be given to events that are (or are suspected to be) serious, unexpected, and related or probably related. Reportable adverse events (i.e. events that are unexpected and related or probably reported) will be provided to the Central IRB and DSMB chair within 5 working days. Deaths that occur within 30 days of the last dose of the study drug will be reported to the Central IRB by phone or email within 24 hours of notification to the PI or research team, followed by a report to the IRB within 5 working days.

Safety Monitoring. A DSMB composed of two neonatologists, one pediatric pharmacologist and one pediatric epidemiologist will meet yearly and generate a report to the PIs and IRB. Dr. Daniel Robinson, a neonatologist, will serve as the medical monitor and will chair DSMB discussions. He will receive all reportable adverse events (i.e., events that are unexpected *and* related or probably related) within 5 working days after the investigators learn of the event. He will also be provided a complete list of all AEs by the study analyst (Brown) prior to each DSMB meeting. Dr. Robinson may request the actual product assigned to an individual if needed. After all data for the study have been monitored, entered, cleaned and locked, a safety report will be generated by the study analyst with input from Dr. Robinson to generate the safety report for publication.

6.1. Bayesian Monitoring

At each of the interim analyses we calculate the posterior distribution one arm has higher rate of subjects with AEs as well as total number of AEs for each model. Here is the basic Rcode for the respective models.

```
###ADORE_SAE
########This function calcualtes the probability tx A has higher SAE rate than tx B
######## 7/26/2018
######## Byron J. Gajewski
######## See protocol for more information

#######Inputs:
#yA= number of SUBJECTS with with at least 1 SAE TREATMENT A
#nA=number enrolled TREATMENT A
#yB= number of SUBJECTS with with at least 1  SAEs TREATMENT B
#nB=number enrolled TREATMENT B
#S=Number of simulation iterarions

#######Outputs:
#PBbest=probability TREATMENT B higher rate than TREATMENT A
#########Function
ADORE_SAE_BySub<-function(yA,nA,yB,nB,S)
{
 ######Proportional posterior (last part is the Jacobian)
 logpost<-function(y,n,P)
 {
  dbinom(y,n,P,log=TRUE)+dnorm(log(P/(1-P)),0,10,log=TRUE)-log(P)-log((1-P))
 }
 ####tx A
 P=runif(S)
 q=exp(logpost(yA,nA,P))/dunif(P)
 q=q/sum(q)
 ###Draws from control parameter
```

```
 thetaA=sample(P,S,replace=TRUE,prob=q)

 ####tx B



 P=runif(S)
 q=exp(logpost(yB,nB,P))/dunif(P)
 q=q/sum(q)
 ###Draws from treatment parameter
 thetaB=sample(P,S,replace=TRUE,prob=q)

 ########

 ####Best arm
 PBhigher=mean(thetaB>=thetaA)

 ###Mean of parameters
 MthetaA=mean(thetaA)
 MthetaB=mean(thetaB)

 ###Variance of parameters
 VthetaA=var(thetaA)
 VthetaB=var(thetaB)


 return(c(PBhigher,MthetaA,MthetaB,VthetaA,VthetaB))
}

####Example (AE example)
##Table 8 (4th interim)
###ADORE_SAE_BySub(yA=176,nA=294,yB=176,nB=264,S=1000000)
```

```
###ADORE_SAE

########This function calculates the probability tx A has higher SAE rate than tx B

######## 7/26/2018
######## Byron J. Gajewski

######## See protocol for more information

#######Inputs:
#yA=TOTAL number of SAEs TREATMENT A
#nA=number enrolled TREATMENT A
```

```
#yB=TOTAL number of SAEs TREATMENT B
#nB=number enrolled TREATMENT B
#S=Number of simulation iterarions

#######Outputs:
#PBbest=probability TREATMENT B higher rate than TREATMENT A


#########Function

ADORE_SAE<-function(yA,nA,yB,nB,S)
{


####A
  ###Draws from treatment parameter
thetaA=rgamma(10000,yA+.01,nA+.1)### Prior SAE is .01, prior sample size .1


####B

###Draws from treatment parameter
thetaB=rgamma(10000,yB+.01,nB+.1)### Prior SAE is .01, prior sample size .1

########

####higher rate SAE
PBhigher=mean(thetaB>=thetaA)


###mean of parameters
MthetaA=mean(thetaA)
MthetaB=mean(thetaB)
###variance of parameters
VthetaA=var(thetaA)
VthetaB=var(thetaB)

return(c(PBhigher,MthetaA,MthetaB,VthetaA,VthetaB))
}

####Example
###ADORE_SAE(yA=51,nA=294,yB=83,nB=264,S=1000000)
```

These posterior probabilities are calculated and monitored for the following tables:

Type of Adverse Event by Treatment Group in Mother's (AE and SAE).

Type of Adverse Event by Treatment Group in Infants (AE and SAE).

6.2. Final Bayesian Hierarchical Monitoring (w/contributions from Yang Wang)

In this model, we will fit the multilevel SAEs model by using ADORE "Incidence of Serious Adverse Event by Body System Across Treatment Groups in Mother's" and will be replicated for "Infants" to see if there is a safety signal.

Priors and Likelihood Functions

For b = 1,2,...,B and j = 1,2,...,k_b, let Y_{bj} and X_{bj} be the number of subjects with an AE with PT j under system organ class SOC b in treatment and placebo groups. N_t and N_c are the number of subjects in treatment and control groups, respectively.

Assuming a binomial likelihood for the AE counts, i.e., Y_{bj} ~Binom(N_t,b_j) and X_{bj} ~Binom(N_c,c_{bj}) where t_{bj} and c_{bj} are the probability of AE for PT j and SOC b in treatment and control groups, respectively. Considering a logistic regression mean structure: logit(c_{bj})=log(c_{bj}/(1−c_{bj})=Υ_{bj}; logit(t_{bj})= Υ_{bj} + θ_{bj}. Note that θ_{bj} =log$\frac{t_{bj}(1-c_{bj})}{c_{bj}(1-t_{bj})}$ is the logarithm of odds ratio (log-OR).

The priors are:
The stage 1 prior distributions are

$$\Upsilon_{bj} \sim N(\mu_{\Upsilon b}, \sigma^2_{\Upsilon b}) \quad \text{and} \quad \theta_{bj} \sim N(\mu_{\theta b}, \sigma^2_{\theta b}).$$

The stage 2 prior distributions are

$$\mu_{\Upsilon b} \sim N(\mu_{\Upsilon 0}, \tau^2_{\Upsilon 0}) \qquad \sigma^2_{\Upsilon b} \sim IG(\alpha_\Upsilon, \beta_\Upsilon)$$
$$\mu_{\theta b} \sim N(\mu_{\theta 0}, \tau^2_{\theta 0}) \qquad \sigma^2_{\theta b} \sim IG(\alpha_\theta, \beta_\theta)$$

The 3 stage prior distributions are

$$\mu_{\Upsilon 0} \sim N(\mu_{\Upsilon 00}, \tau^2_{\Upsilon 00}) \qquad \tau^2_{\Upsilon 0} \sim IG(\alpha_{\Upsilon 00}, \beta_{\Upsilon 00})$$
$$\mu_{\theta 0} \sim N(\mu_{\theta 00}, \tau^2_{\theta 00}) \qquad \tau^2_{\theta 0} \sim IG(\alpha_{\theta 00}, \beta_{\theta 00})$$

The hyperparameters $\mu_{\Upsilon 00}$, $\tau^2_{\Upsilon 00}$, $\mu_{\theta 00}$, $\tau^2_{\theta 00}$, $\alpha_{\Upsilon 00}$, $\beta_{\Upsilon 00}$, $\alpha_{\theta 00}$, $\beta_{\theta 00}$, α_Υ, β_Υ, α_θ, β_θ are considered fixed constants. In the simulation, the following values as Xia's paper are used: $\mu_{\Upsilon 00} = \mu_{\theta 00} = 0$, $\tau^2_{\Upsilon 00} = \tau^2_{\theta 00} = 10$, $\alpha_{\Upsilon 00} = \alpha_{\theta 00} = \alpha_\Upsilon = \alpha_\theta = 3$, $\beta_{\Upsilon 00} = \beta_{\theta 00} = \beta_\Upsilon = \beta_\theta = 1$.

Simulation results for Example Data
Results from multilevel SAEs model:

Simulation was done in OpenBug. Results of Odds-Ratio is as following.

	mean	sd	MC_error	val2.5pc	median	val97.5pc
OR[1,1]	0.5706	0.4493	0.001717	0.07492	0.4585	1.724
OR[1,2]	0.9525	0.6163	0.002285	0.2449	0.804	2.529
OR[1,3]	0.5345	0.4113	0.001635	0.07069	0.4343	1.588
OR[1,4]	1.368	0.7339	0.002959	0.4647	1.203	3.228
OR[2,1]	0.4457	0.4197	0.00176	0.05009	0.3296	1.533
OR[2,2]	0.4444	0.4189	0.001756	0.04968	0.3294	1.517
OR[3,1]	0.5045	0.4625	0.002074	0.06246	0.3776	1.688
OR[3,2]	0.5035	0.4591	0.002043	0.06299	0.3782	1.68
OR[3,3]	0.7287	0.7095	0.002853	0.1058	0.5369	2.498
OR[4,1]	1.169	1.127	0.004743	0.1946	0.866	3.949
OR[4,2]	0.6169	0.5646	0.002367	0.07599	0.4634	2.068
OR[5,1]	0.5015	0.3376	0.001279	0.09981	0.4244	1.364
OR[5,2]	0.6275	0.4085	0.001458	0.149	0.5327	1.669
OR[5,3]	0.7195	0.518	0.001794	0.1648	0.5938	2.019
OR[5,4]	0.5296	0.369	0.001326	0.1041	0.4434	1.466
OR[5,5]	0.9957	0.6644	0.002279	0.272	0.8303	2.712
OR[5,6]	0.719	0.5155	0.001712	0.165	0.5931	2.01
OR[5,7]	0.9448	0.5112	0.001793	0.313	0.8307	2.246
OR[5,8]	0.462	0.2431	9.15E-4	0.136	0.4142	1.064
OR[5,9]	0.6603	0.3914	0.001388	0.1832	0.5738	1.652
OR[6,1]	0.4483	0.4279	0.001822	0.05006	0.3314	1.542
OR[6,2]	0.4473	0.4257	0.001766	0.05068	0.3299	1.537
OR[7,1]	0.7625	0.6128	0.002391	0.144	0.6007	2.337
OR[7,2]	0.6031	0.4223	0.001746	0.1256	0.4993	1.699
OR[7,3]	0.7171	0.5524	0.002218	0.14	0.5736	2.137
OR[7,4]	0.7186	0.5574	0.002168	0.1384	0.575	2.138
OR[8,1]	0.6784	0.5239	0.002272	0.13	0.5425	2.036
OR[8,2]	0.4928	0.3812	0.001736	0.07572	0.3962	1.479
OR[8,3]	0.9953	0.8435	0.003579	0.1955	0.7685	3.14
OR[8,4]	0.7636	0.6407	0.002623	0.1377	0.5937	2.4
OR[8,5]	0.5409	0.4396	0.001895	0.08039	0.4266	1.678

It can be seen that only OR[1,4] and OR[4,1] are greater than 1. If we use 1.2 as cutoff for significant AE, only OR[1,4] will be considered as significant AE. If we use 1.5 as cutoff, there is no significant AE.

DIC of the model is 191.8. Please see following.

	Dbar	Dhat	DIC	pD
X	93.04	80.51	105.6	12.52
Y	73.69	61.14	86.24	12.55
total	166.7	141.7	191.8	25.08

Using R code Appendix, predicted X and Y can be obtained as following (left).

b	j	Ypred	Xpred
1	1	2	1
1	2	2	0
1	3	1	5
1	4	8	6
2	1	0	0
2	2	0	0
3	1	0	3
3	2	0	0
3	3	1	0
4	1	1	1
4	2	0	2
5	1	1	5
5	2	2	0
5	3	1	1
5	4	1	0
5	5	0	1
5	6	2	2
5	7	2	5
5	8	3	4
5	9	0	1
6	1	1	1
6	2	0	2
7	1	1	2
7	2	5	2
7	3	2	3
7	4	0	2
8	1	2	4
8	2	2	2
8	3	1	3
8	4	0	2
8	5	0	0

Y_{bj}	Rate	X_{bj}	Rate
0	0.000	1	0.003
3	0.009	3	0.009
0	0.000	2	0.006
8	0.025	6	0.019
0	0.000	1	0.003
0	0.000	1	0.003
0	0.000	1	0.003
0	0.000	1	0.003
1	0.003	0	0.000
2	0.006	0	0.000
0	0.000	1	0.003
0	0.000	2	0.006
1	0.003	2	0.006
1	0.003	0	0.000
0	0.000	1	0.003
3	0.009	1	0.003
1	0.003	0	0.000
5	0.016	5	0.016
2	0.006	10	0.031
2	0.006	4	0.013
0	0.000	1	0.003
0	0.000	1	0.003
1	0.003	0	0.000
1	0.003	4	0.013
1	0.003	1	0.003
1	0.003	1	0.003
1	0.003	2	0.006
0	0.000	3	0.009
2	0.006	0	0.000
1	0.003	0	0.000
0	0.000	1	0.003

Comparing with the data we have (right), it looks consistent.

The box plots from multilevel SAE model and Beta Binomial are as following.

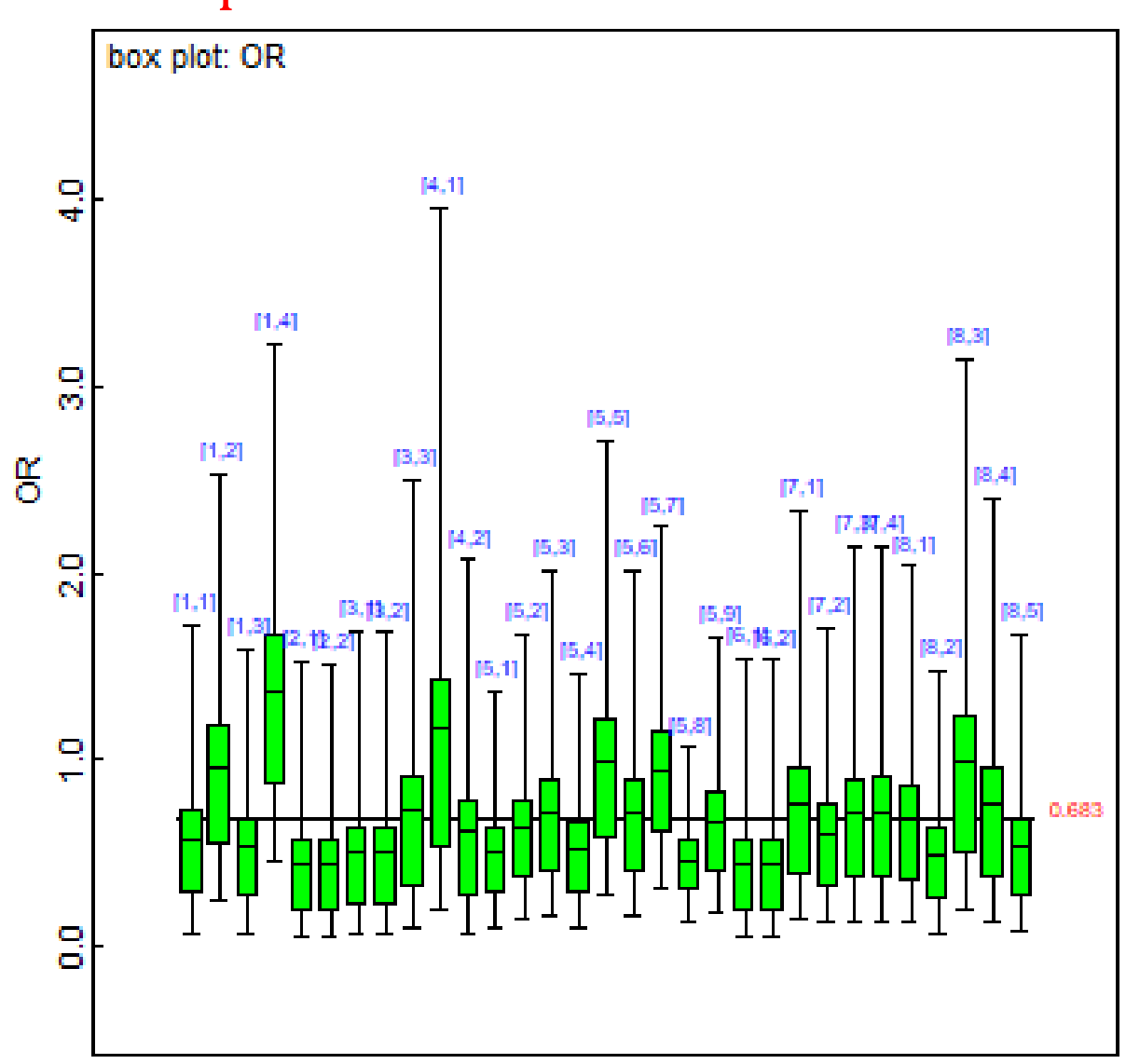

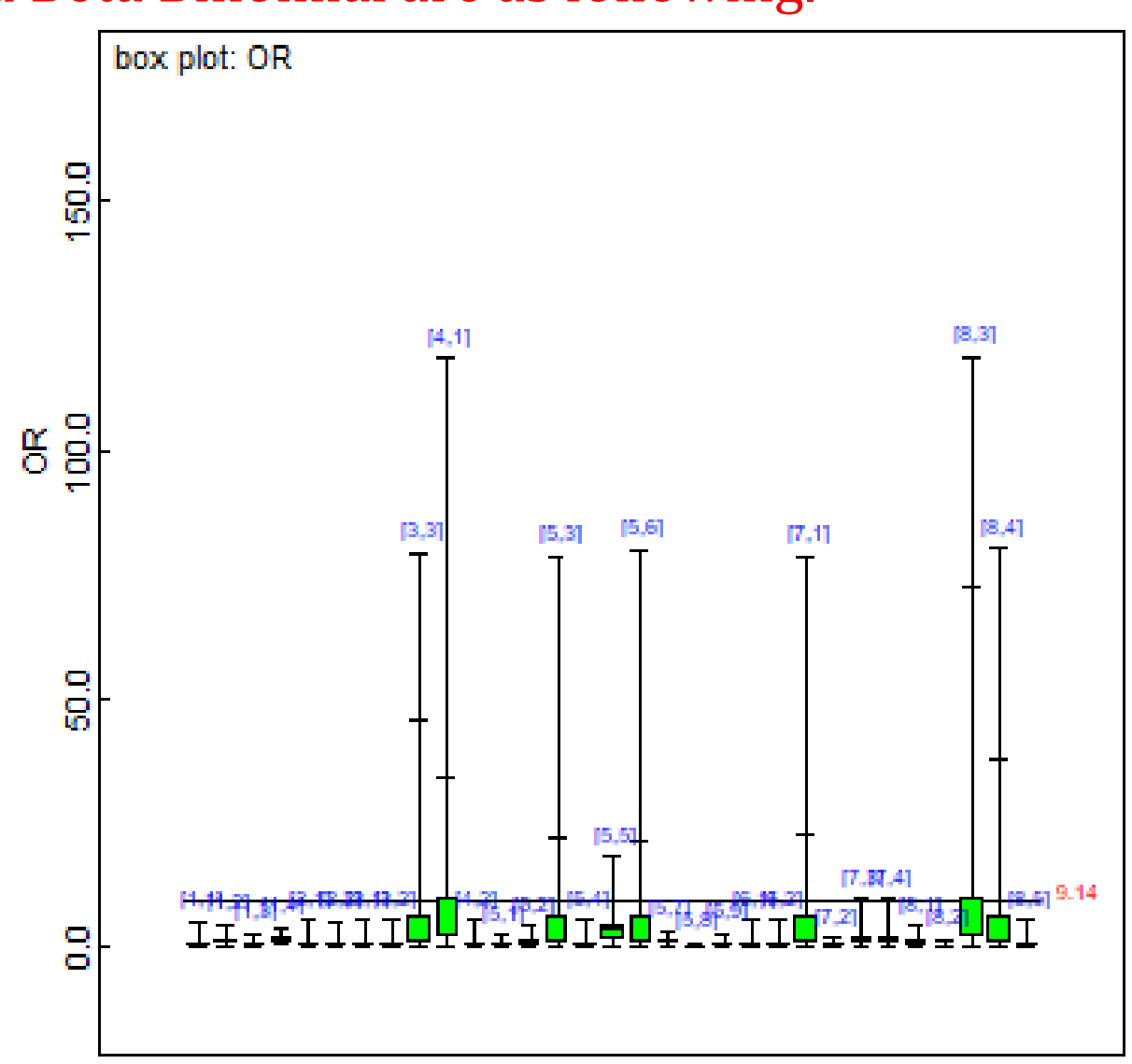

Results from one level Beta Binomial SAEs model :

	mean	sd	MC_error	val2.5pc	median	val97.5pc
OR[1,1]	0.9863	3.164	0.006507	0.01273	0.4098	5.239
OR[1,2]	1.339	1.257	0.002606	0.2247	0.9996	4.496
OR[1,3]	0.4982	0.8738	0.001749	0.00836	0.2585	2.429
OR[1,4]	1.513	0.8871	0.001846	0.4718	1.306	3.778
OR[2,1]	1.008	3.472	0.006883	0.01264	0.4139	5.331
OR[2,2]	0.9911	2.964	0.005884	0.01264	0.4126	5.26
OR[3,1]	1.012	4.422	0.00913	0.01256	0.4142	5.349
OR[3,2]	1.005	3.122	0.006431	0.01253	0.4163	5.362
OR[3,3]	45.83	11470.0	22.92	0.1857	2.412	79.2
OR[4,1]	34.4	1192.0	2.402	0.4154	3.896	118.2
OR[4,2]	1.005	3.221	0.006717	0.01291	0.4125	5.363
OR[5,1]	0.4985	0.8676	0.001705	0.008337	0.2582	2.41
OR[5,2]	0.9986	1.375	0.002893	0.07139	0.6247	4.149
OR[5,3]	22.29	1009.0	1.993	0.1863	2.415	78.55
OR[5,4]	1.027	9.335	0.01857	0.01273	0.4136	5.396
OR[5,5]	4.036	9.976	0.02027	0.3932	2.205	18.1
OR[5,6]	21.12	572.7	1.21	0.1862	2.418	79.84
OR[5,7]	1.203	0.8247	0.00168	0.2997	0.9997	3.302
OR[5,8]	0.293	0.2053	4.071E-4	0.05085	0.2439	0.8219
OR[5,9]	0.7479	0.6627	0.001328	0.1083	0.5694	2.446
OR[6,1]	1.0	3.58	0.006915	0.01267	0.4136	5.356
OR[6,2]	1.007	3.539	0.007289	0.01291	0.4131	5.353
OR[7,1]	22.31	1185.0	2.349	0.186	2.414	78.67
OR[7,2]	0.4968	0.4989	0.001075	0.04444	0.3557	1.79
OR[7,3]	2.02	6.883	0.01362	0.1028	1.001	9.669
OR[7,4]	2.002	6.699	0.01347	0.1038	1.003	9.602
OR[8,1]	0.9974	1.445	0.002867	0.07248	0.625	4.161
OR[8,2]	0.331	0.472	9.592E-4	0.006254	0.1869	1.51
OR[8,3]	72.56	20680.0	41.32	0.409	3.873	118.6
OR[8,4]	37.75	5099.0	10.18	0.1871	2.424	80.04
OR[8,5]	1.002	3.842	0.008058	0.01254	0.4132	5.323

It can be seen that from one level Beta binomial model, it can be seen that we have quite a few Odds-Ratio greater than 1. There are even some AEs having OR greater than 20. So from this model, more AEs are identified as significant and multiplicity was not well considered by this model.

The DIC is as following 217.5.

	Dbar	Dhat	DIC	pD
X	100.5	84.66	116.3	15.82
Y	89.58	77.93	101.2	11.65
total	190.1	162.6	217.5	27.48

Comparing the two models by using DIC, we can tell that the multilevel SAE model is better. Also, from the results, it can be seen that the multilevel SAE model well considered the multiplicity and not as many SAEs were considered as significant as the Beta binomial model.

In a nut shell, the multilevel SAE model is much better than the regular Beta binomial model and has a more reasonable filter for identifying significance of SAEs.

```
OpenBug code for multilevel SAE model:
## Data example:
list(Nae = 31, Nc = 320, Nt = 320, B = 8, b = c(1, 1, 1, 1, 2, 2, 3, 3, 3, 4, 4, 5, 5, 5, 5, 5, 5, 5, 5, 5,
6, 6, 7, 7, 7, 7, 8, 8, 8, 8, 8), j = c(1, 2, 3, 4,  1, 2, 1, 2, 3, 1, 2, 1, 2, 3, 4, 5, 6, 7, 8, 9, 1, 2, 1, 2, 3, 4,
1, 2, 3, 4, 5), Y = c( 0, 3, 0, 8, 0, 0, 0, 0, 1, 2, 0, 0, 1, 1, 0, 3, 1, 5, 2, 2, 0, 0, 1, 1, 1, 1, 1, 0, 2, 1, 0),
X = c(1, 3, 2, 6, 1, 1, 1, 1, 0, 0, 1, 2, 2, 0, 1, 1, 0, 5, 10, 4, 1, 1, 0, 4, 1, 1, 2, 3, 0, 0, 1))
model{
for (i in 1:Nae) {
X[i] ~ dbin(c[b[i], j[i]], Nc)
Y[i] ~ dbin(t[b[i], j[i]], Nt)
logit(c[b[i], j[i]]) <- gamma[b[i], j[i]]
logit(t[b[i], j[i]]) <- gamma[b[i], j[i]] + theta[b[i], j[i]]
gamma[b[i], j[i]] ~ dnorm(mu.gamma[b[i]], tau.gamma [b[i]])
theta[b[i], j[i]] ~ dnorm(mu.theta[b[i]], tau.theta[b[i]])
 OR[b[i],j[i]] <- exp(theta[b[i],j[i]] )
# SOC level parameters
for(k in 1:B){
mu.gamma[k] ~ dnorm(mu.gamma.0, tau.gamma.0)
tau.gamma[k] ~ dgamma(3,1)
mu.theta[k] ~ dnorm(mu.theta.0, tau.theta.0)
tau.theta[k] ~ dgamma(3,1)
}
# hyperpriors for gamma's;
mu.gamma.0 ~ dnorm(0, 0.1)
tau.gamma.0~ dgamma(3,1)
# hyperpriors for theta's;
mu.theta.0 ~ dnorm(0, 0.1)
tau.theta.0 ~ dgamma(3,1)
}
OpenBug code for Beta Binomial model:
## Data example:
list(Nae = 31, Nc = 320, Nt = 320, b = c(1, 1, 1, 1, 2, 2, 3, 3, 3, 4, 4, 5, 5, 5, 5, 5, 5, 5, 5, 5, 6, 6, 7,
7, 7, 7, 8, 8, 8, 8, 8), j = c(1, 2, 3, 4,  1, 2, 1, 2, 3, 1, 2, 1, 2, 3, 4, 5, 6, 7, 8, 9, 1, 2, 1, 2, 3, 4, 1, 2, 3,
4, 5), Y = c( 0, 3, 0, 8, 0, 0, 0, 0, 1, 2, 0, 0, 1, 1, 0, 3, 1, 5, 2, 2, 0, 0, 1, 1, 1, 1, 1, 0, 2, 1, 0), X = c(1,
3, 2, 6, 1, 1, 1, 1, 0, 0, 1, 2, 2, 0, 1, 1, 0, 5, 10, 4, 1, 1, 0, 4, 1, 1, 2, 3, 0, 0, 1))
model{
for (i in 1:Nae) {
X[i] ~ dbin(c[b[i], j[i]], Nc)
Y[i] ~ dbin(t[b[i], j[i]], Nt)
 OR[b[i],j[i]] <- exp(logit(t[b[i],j[i]]) -logit(c[b[i], j[i]]))
}
 # hyperpriors
for (i in 1:Nae) {
c[b[i], j[i]]~ dbeta(1, 1)
t[b[i], j[i]]~ dbeta(1, 1)
```

```
}
 }

R codes for getting predict X and Y:
library(R2WinBUGS)
dd<-list(Nae = 31, Nc = 320, Nt = 320, B = 8, b = c(1, 1, 1, 1, 2, 2, 3, 3, 3, 4, 4, 5, 5, 5, 5, 5, 5, 5,
5, 5, 6, 6, 7, 7, 7, 7, 8, 8, 8, 8, 8), j = c(1, 2, 3, 4,  1, 2, 1, 2, 3, 1, 2, 1, 2, 3, 4, 5, 6, 7, 8, 9, 1, 2, 1, 2,
3, 4, 1, 2, 3, 4, 5), Y = c( 0, 3, 0, 8, 0, 0, 0, 0, 1, 2, 0, 0, 1, 1, 0, 3, 1, 5, 2, 2, 0, 0, 1, 1, 1, 1, 1, 0, 2, 1,
0), X = c(1, 3, 2, 6, 1, 1, 1, 1, 0, 0, 1, 2, 2, 0, 1, 1, 0, 5, 10, 4, 1, 1, 0, 4, 1, 1, 2, 3, 0, 0, 1))
Nae=dd$Nae
Nc=dd$Nc
Nt=dd$Nt
B=dd$B
b=dd$b
j=dd$j
Y=dd$Y
X=dd$X
data <- list ("Nae","Nc","Nt","B","b","j","Y", "X")
final.sim = bugs(data,inits=NULL,
model.file="D:/Bayesion analysis/BIOS902 HW/Model1a.txt",
parameters=c("c","t","OR"),
n.chains=1,n.iter=100000,n.burnin=1000,n.thin=1,
bugs.directory="C:/Users/YOUNG/Desktop/winbugs143_unrestricted
(1)/winbugs14_full_patched/WinBUGS14")

print(final.sim,decimal=.0001)
print(final.sim$mean)
mean.OR=as.vector(t(final.sim$mean$OR))[1:31]
mean.c=as.vector(t(final.sim$mean$c))[1:31]
mean.t=as.vector(t(final.sim$mean$t))[1:31]

Xpred<-Ypred<-rep(NA,Nae)

for(i in 1:Nae)
{
Xpred[i]=rbinom(1,Nc,mean.c[i])
Ypred[i]=rbinom(1,Nt,mean.t[i])
}
Pred=data.frame(b,j,Ypred,Xpred)
```

7. Secondary Analyses for Aim 1:

7.1. Determine if a supplement of 1000 mg DHA /day compared to 200 mg DHA/day during the last two trimesters of pregnancy can reduce ePTB

The original ADORE trial was powered using a Bayesian model for early preterm birth dichotomous variable. To better estimate the implications of DHA, we utilize more sophisticated modeling to improve the efficiency of estimating the effect of DHA on early preterm birth.

We perform a dichotomization of continuous data (<34 weeks) but preserves the statistical power of its continuous parent.

North Carolina Detailed Birth Record (NCDBR) Database

To inform the structure of a mixture model (i.e., mixture of univariate normal, three components), we use information from the NCDBR data that had 371,924 singleton births from 2004-2006. Specifically, for the univariate formulation described above we specify component means as

$$\boldsymbol{\mu}_1 = [39.59],$$
$$\boldsymbol{\mu}_2 = [38.26], \text{ and}$$
$$\boldsymbol{\mu}_3 = [33.29].$$

Associated component covariance matrices are

$$\boldsymbol{\Sigma}_1 = 0.960,$$
$$\boldsymbol{\Sigma}_2 = 2.480, \text{ and}$$
$$\boldsymbol{\Sigma}_3 = 13.230.$$

The specific values of $\boldsymbol{\mu}_1, \boldsymbol{\mu}_2, \boldsymbol{\mu}_3, \boldsymbol{\Sigma}_1, \boldsymbol{\Sigma}_2$, and $\boldsymbol{\Sigma}_3$ come from posterior estimates obtained from the three components discovered from the model of the birth registry data.

Let $\mathbf{Y}_i$ be the joint variables for gestational age of the ith birth from ADORE. Let $\boldsymbol{\theta}$ be the parameter vector of mixture parameters for 200 mg/day and $\boldsymbol{\omega}$ be the effect comparing 200 mg/day to 1000 mg/day. Using the same logic as the historical model, we get the following model for the experimental data:

$$\boldsymbol{f}(\mathbf{Y}_i|\boldsymbol{\theta},\boldsymbol{\omega}) \sim \sum_{k=1}^{2} \frac{\exp(\theta_k + d_i\omega_k)}{\sum_{m=1}^{3}\exp(\theta_m + d_i\omega_m)} \{\phi(\mathbf{Y}_i|\boldsymbol{\mu}_k,\boldsymbol{\Sigma}_k) - \phi(\mathbf{Y}_i|\boldsymbol{\mu}_3,\boldsymbol{\Sigma}_3)\} + \phi(\mathbf{Y}_i|\boldsymbol{\mu}_3,\boldsymbol{\Sigma}_3).$$

In general, for the bth iteration, MCMC provides us a straightforward calculation for anything we desire. Since we are interested in the joint probabilities of <34 weeks, we define $\mathbf{y}^* = (34)$ and can easily get what we desire from these draws: for 200 mg/day

$p(Y < 34) = \sum_{k=1}^{2} \frac{\exp\left(\theta_k^{(b)}\right)}{\sum_{m=1}^{3} exp\left(\theta_k^{(b)}\right)} \{\Phi(\mathbf{y}^*|\boldsymbol{\mu}_\mathbf{k}, \boldsymbol{\Sigma}_\mathbf{k}) - \Phi(\mathbf{y}^*|\boldsymbol{\mu}_3, \boldsymbol{\Sigma}_3)\} + \Phi(\mathbf{y}^*|\boldsymbol{\mu}_3, \boldsymbol{\Sigma}_3)$, and 1000 mg/day

$p(Y < 34) = \sum_{k=1}^{2} \frac{\exp\left(\theta_k^{(b)} + \omega_k^{(b)}\right)}{\sum_{m=1}^{3} exp\left(\theta_m^{(b)} + \omega_m^{(b)}\right)} \{\Phi(\mathbf{y}^*|\boldsymbol{\mu}_\mathbf{k}, \boldsymbol{\Sigma}_\mathbf{k}) - \Phi(\mathbf{y}^*|\boldsymbol{\mu}_3, \boldsymbol{\Sigma}_3)\} + \Phi(\mathbf{y}^*|\boldsymbol{\mu}_3, \boldsymbol{\Sigma}_3)$,

where $\Phi(\cdot\,|\boldsymbol{\mu}_\mathbf{k}, \boldsymbol{\Sigma}_\mathbf{k})$ is the bivariate normal cumulative distribution function for k=1,2,3.

For comparison purposes we calculate the posterior distribution that DHA has a lower risk than placebo control $P\left(200\,\frac{\text{mg}}{\text{day}} \geq 1000\text{mg/day}\right)$ which is estimated the proportion of times $p\left(Y < 34\middle|\boldsymbol{\theta}^{(\mathbf{b})}\right)$ is larger or equal to $p\left(Y < 34\middle|\boldsymbol{\theta}^{(\mathbf{b})} + \boldsymbol{\omega}^{(\mathbf{b})}\right)$.

Here are the power improvements (Fengming Tang):

Scenario (p1, p2)	Power in Facts (cutoff 0.99)	Power using mixture model (calibrated, cutoff 0.96, Fixed μ *and* σ)	Power using mixture model (calibrated, cutoff 0.96, Free μ *and* σ)
0.04 vs 0.01	0.927	0.981	0.961
0.03 vs 0.005	0.919	0.975	0.947
0.03 vs 0.01	0.66	0.821	0.801
0.03 vs 0.02	0.16	0.303	0.287
0.03 vs 0.03	0.051	0.049	0.048

7.2. Determine if a supplement of 1000 mg DHA /day compared to 200 mg DHA/day during the last two trimesters of pregnancy can reduce ePTB but varies across (RBC-PL-DHA) at Enrollment (<6% versus >= 6%) (TTE Model help by Fengming Tang)

Time to event model using 3 normals mixture.

The 6% cut-off was motivated by our desire to replicate the interaction found in the ORIP study done in Australia (Simmonds et al., 2020, BJOG, DOI: 10.1111/1471-0528.16168). To compare our DHA estimate to Australia. We multiply by 0.4754. This estimate cane from a factor that Bill Harris derived to compare blood spot to RBC DHA adjusted by a factor derived from a comparison Bill and Scott did. If we take our 6% DHA at baseline and multiply by .4754 we get 2.85% in Australia, which is about the spot where the plot on the lower right panel changes risk profiles (Figure 3).

In this approach, we have four categories with doses 200+LowRBC, 1000+LowRBC, 200+highRBC, 1000+highRBC, d=1,2,3, and 4. For 200+LowRBC group (d=1), we use 3 normals mixture model as in Section 7.1.

In this case let $\theta_d = p_d(Y < 34)$ be the probability of early preterm birth from group d.

We will test if early preterm births vary across RBC, H1: $\theta_1 > \theta_2$ and $\theta_3 > \theta_4$ (e.g. early birth is riskier for lower doses). We also hypotheses that the higher dose DHA has a bigger impact for LowRBC than for highRBC, i.e. H1: $\theta_1 - \theta_2 > \theta_3 - \theta_4$.

7.3. Determine if a supplement of 1000 mg DHA /day compared to 200 mg DHA/day during the last two trimesters of pregnancy can reduce ePTB but varies across time ("drift analysis") (TTE Model help by Fengming Tang)

We will use the time to event model described in section 7.2 but with a normal dynamic linear model across time to see if there is a "drift effect." Each group (200 mg/day & 1000 mg/day will have a separate NDLM). Let θ_t^{200} be the log hazard ratio for one of 200 mg/day and let t=interim analysis time of enrollment. Let θ_t^{1000} be for 1000 mg/day. The NDLM will be modeled as $\theta_t^{200} \sim N(\theta_{t-1}^{200}, \tau^2)$, where $\theta_1^{200} = 0$ and $\tau^2 \sim IG(.01, .01)$. The model is repeated for θ_t^{1000} but θ_1^{1000} is free. We will plot 95% credible intervals for $\theta_t^{200} - \theta_t^{1000}$.

7.4. Determine if a supplement of 1000 mg DHA /day compared to 200 mg DHA/day during the last two trimesters of pregnancy can reduce ePTB but varies across time ("compliance model")

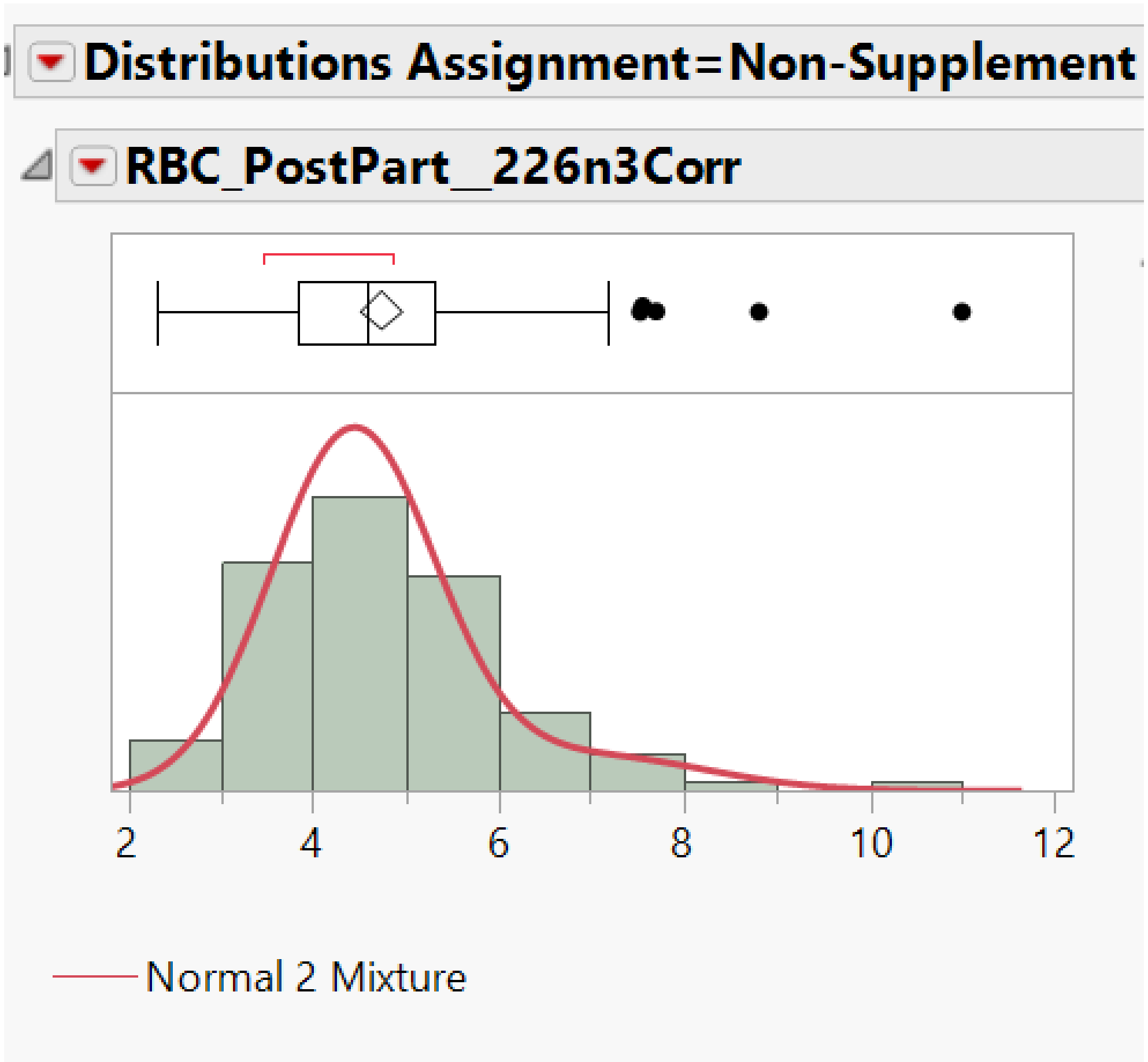

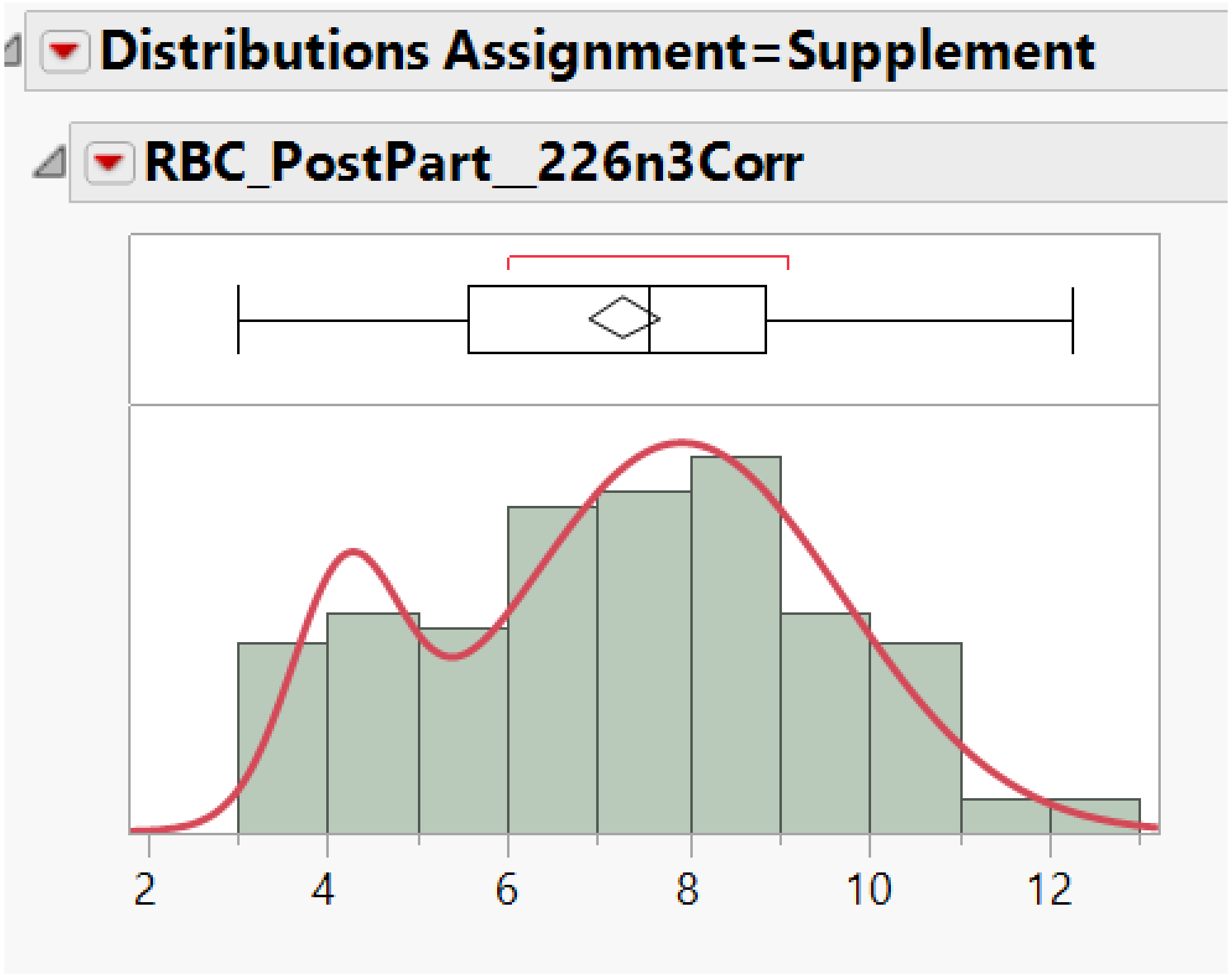

From these KUDOS plots for PP RBC DHA, we can create three subgroups. The first is non-supplement, the second is supplement (<5.5%), the third is supplement (>= 5%). The second and third groups represent "compliers" and "non-compliers." Using this definition we get the following results (one outstanding question is what to do with "missing"), for this purpose we placed "missing" in the "non-compliers" but this may need to be modified.

We will utilize a similar approach to define four groups in ADORE and analyze using the mixture model in section 7.1.

8. Secondary Analyses for Aim 4: NICU Stays Across Arms

We will define Y to be the number of days the baby was in the NICU. Y will be 0 if there is no documentation of NICU stay. We will calculate the posterior probability the NICU days are different across the two groups using a Bayesian Poisson model with flat priors.
