# Supplementary material for: Genotype-informed docosahexaenoic acid dosing based on the FADS Indel to reduce preterm and prolonged gestation: A secondary analysis of the ADORE randomized trial
Source: PLoS Med. 2026 Oct 8;23(10):e1005274. doi: 10.1371/journal.pmed.1005274 (PMC13660990; doi:10.1371/journal.pmed.1005274)
Supplement: Supplementary file 4 [file pmed.1005274.s023.pdf]

Response to reviewer

Comments #3 and #5 require no response.

Comment #1 - OK but perhaps add this clarification somewhere in the manuscript as well?

Response: We have added the following to the beginning of the results section for clarification: “Of those screened, 3,966 were categorized as excluded for 'other reasons,' a category that predominantly represents women who were not approached by study staff due to staffing limitations at the Ohio sites and were therefore not assessed for interest or eligibility.”

Comment #2 - OK, the authors have addressed this in the manuscript but not the abstract even though the response letter states exactly that - maybe just an oversight?

Response: Apologies, we have added the proposed changes from last round into the abstract.

Comment #4 - The response in the letter clarifies the issue satisfactorily, but I note that Figure 1 seems to be unchanged and still has the original order of the boxes with the original numbers rather than the reversed order and associated changes in numbers. Maybe just another oversight when the figure file was uploaded?

Response: We have uploaded the correct, updated Figure 1.

Response to editor

Comment #1 - In the Kaplan-Meier curve(s) please provide the number at risk for each time interval.

Response: We have provided all numbers at risk for each time interval beneath all our Kaplan-Meier curves. See example below

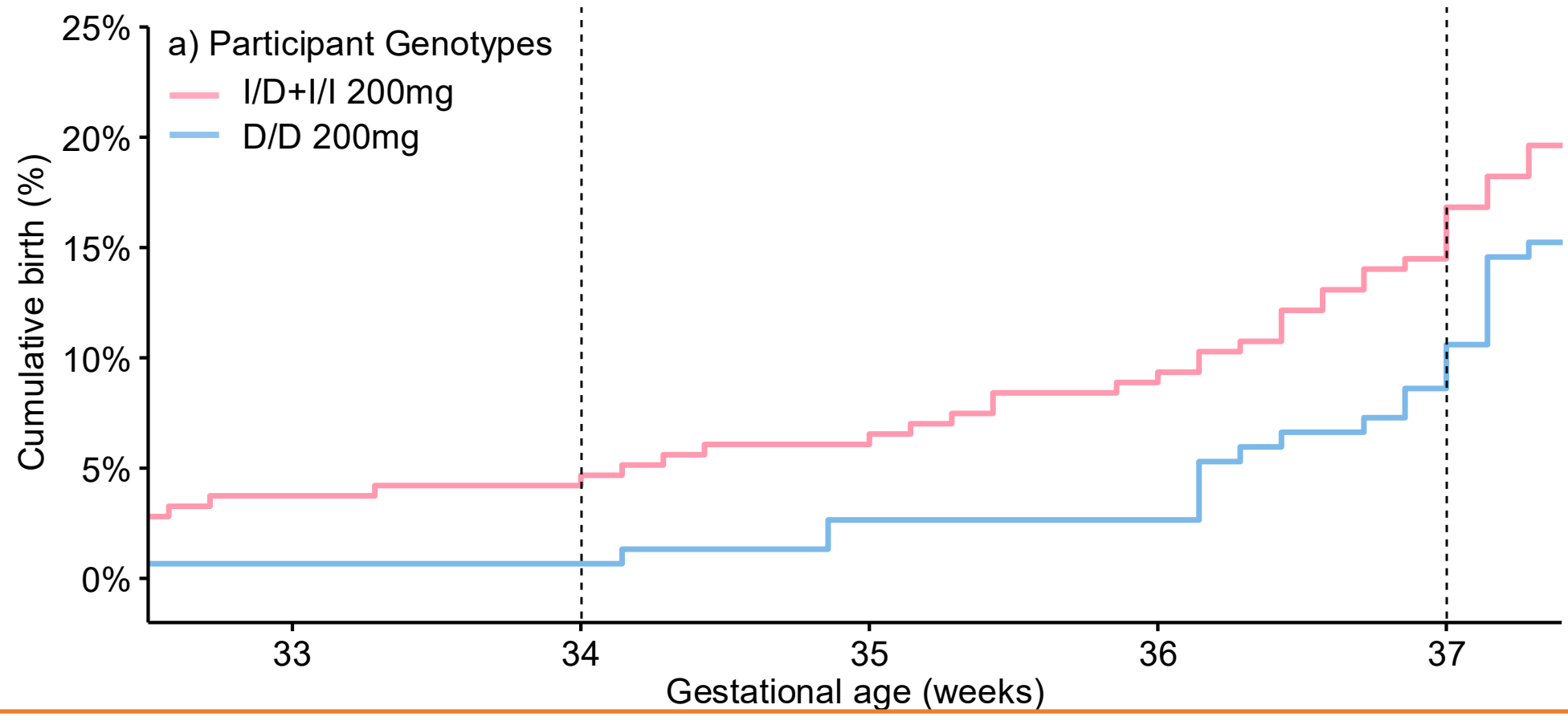

Number of Births

	33	34	35	36	37
I/D+I/D 200mg	1	4	6	12	22
D/D 200mg	0	3	0	9	21

Gestational Age (weeks)

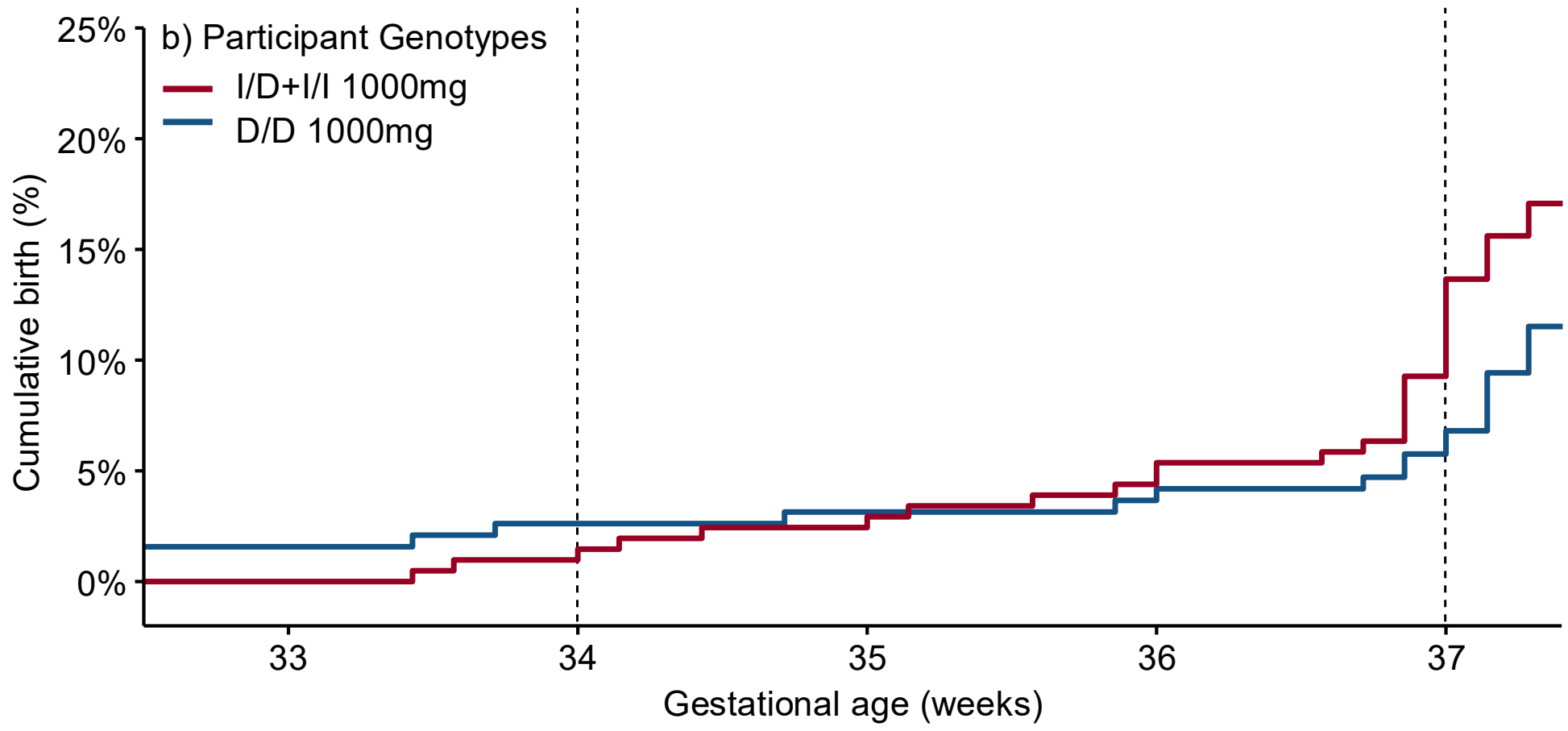

Number of Births

	33	34	35	36	37
I/D+I/D 1000mg	2	3	4	10	30
D/D 1000mg	2	1	1	4	18

Gestational Age (weeks)

Comment #2 - Please include a statement regarding use or non-use of AI tools and technologies at the end of the Methods, under the subheading: Use of AI Disclosure. Please specify the name(s) and version(s) of any tool(s) used and a description of their use, including how the AI-assisted or generated content was evaluated by authors, and a clear statement of which aspects of the study, article contents, data, or supporting files were affected or generated by AI tool usage. Please also include a statement specifying who retains responsibility for the final manuscript. See our guidelines for more information: https://journals.plos.org/plosmedicine/s/ethical-publishing-practice#loc-artificial-intelligence-tools-and-technologies.

Response: We have updated our disclosure subheading to “Use of AI Disclosure”. It is present from line 307-313, at the end of the Methods section.

Comment #3 - Please revise your Data Availability Statement in the manuscript metadata to match that in the manuscript.

Response: Our Data Availability Statements now match.
