# Supplementary material for: Genotype-informed docosahexaenoic acid dosing based on the FADS Indel to reduce preterm and prolonged gestation: A secondary analysis of the ADORE randomized trial
Source: PLoS Med. 2026 Oct 8;23(10):e1005274. doi: 10.1371/journal.pmed.1005274 (PMC13660990; doi:10.1371/journal.pmed.1005274)
Supplement: Supplementary file 5 [file pmed.1005274.s012.docx]

**S1 Appendix**

1. Table A. MCMC convergence and sampling diagnostics for the Bayesian binomial models

2. Table B. Prior-sensitivity analysis

3. Table C. Hierarchical model sensitivity analysis

4. Table D. Bayesian posteriors in the 200 mg arm: unweighted vs IPW-weighted (PB, EPB)

5. Table E. Covariate balance and composition (unweighted vs IPW-weighted): SMDs, TVD, and effective sample size

6. Table F. IPW sensitivity across denominator models and truncation

7. Table G. Genotype-selection bias tipping-point analysis (Γ down-weighting of I-carriers)

8. Table H. Overall PB (<37 weeks) by dose and allele—observed rates, posterior means (95% CrI), posterior probability

9. Table I. Overall EPB (<34 weeks) by dose and allele—observed rates, posterior means (95% CrI), posterior probability

10. Table J. Black participants—PB (<37 weeks) by dose and allele—observed rates and posterior means

11. Table K. Black participants—EPB (<34 weeks) by dose and allele—observed rates and posterior means

12. Table L. PB/EPB and ARA:DHA & ARA:DGLA ratios at baseline and birth by race, genotype, and dose

13. Table M. Baseline RBC-PL fatty acid composition (% of total fatty acids) by genotype

14. Table N. Outcome-tilted (worst-case) sensitivity analysis within I-allele carriers

15. Table O. Baseline characteristics by genotype

16. Table P. Preterm birth by socioeconomic category and genotype

**Table A.** MCMC convergence and sampling diagnostics for the Bayesian binomial models.

Each row corresponds to one fitted Bayesian binomial model (28 models in total). For each model, the reported R-hat, bulk effective sample size (bulk-ESS), and tail effective sample size (tail-ESS) are the worst-case values across the model's parameters. All models showed satisfactory convergence and sampling performance, with maximum R-hat = 1.003, minimum bulk-ESS = 3,591, minimum tail-ESS = 3,393, no divergent transitions, no maximum-treedepth saturations, and minimum E-BFMI = 0.82. Models were fitted with four Hamiltonian Monte Carlo chains (4,000 iterations; 1,000 warm-up). R-hat, potential scale reduction factor (Gelman–Rubin); bulk-ESS/tail-ESS, bulk and tail effective sample size; E-BFMI, energy Bayesian fraction of missing information; PB, preterm birth; EPB, early preterm birth; IPW, inverse-probability weighting.

| **Model** | **Outcome** | **max R-hat** | **min Bulk-ESS** | **min Tail-ESS** | **Divergences** | **E-BFMI** |
| --- | --- | --- | --- | --- | --- | --- |
| Dose contrast (200 vs 1000 mg) — D/D, all | PB (<37 wk) | 1.0005 | 6,318 | 5,550 | 0 | 0.96 |
| Dose contrast (200 vs 1000 mg) — I/D, all | PB (<37 wk) | 1.0005 | 6,515 | 5,317 | 0 | 0.95 |
| Dose contrast (200 vs 1000 mg) — I/I, all | PB (<37 wk) | 1.0006 | 5,936 | 5,490 | 0 | 1.01 |
| Dose contrast (200 vs 1000 mg) — D/D, all | EPB (<34 wk) | 1.0011 | 4,979 | 3,691 | 0 | 0.85 |
| Dose contrast (200 vs 1000 mg) — I/D, all | EPB (<34 wk) | 1.0017 | 5,374 | 4,173 | 0 | 0.95 |
| Dose contrast (200 vs 1000 mg) — I/I, all | EPB (<34 wk) | 1.0013 | 5,337 | 3,703 | 0 | 0.87 |
| Dose contrast (200 vs 1000 mg) — D/D, Black | PB (<37 wk) | 1.0010 | 4,686 | 3,514 | 0 | 0.82 |
| Dose contrast (200 vs 1000 mg) — I/D, Black | PB (<37 wk) | 1.0007 | 5,695 | 4,723 | 0 | 0.95 |
| Dose contrast (200 vs 1000 mg) — I/I, Black | PB (<37 wk) | 1.0005 | 5,918 | 4,680 | 0 | 0.96 |
| Dose contrast (200 vs 1000 mg) — D/D, Black | EPB (<34 wk) | 1.0007 | 3,591 | 3,481 | 0 | 0.92 |
| Dose contrast (200 vs 1000 mg) — I/D, Black | EPB (<34 wk) | 1.0030 | 5,062 | 3,789 | 0 | 0.88 |
| Dose contrast (200 vs 1000 mg) — I/I, Black | EPB (<34 wk) | 1.0002 | 3,898 | 3,604 | 0 | 0.87 |
| Genotype contrast (I-carrier vs D/D), 200 mg — unweighted | PB (<37 wk) | 1.0007 | 6,140 | 5,051 | 0 | 0.94 |
| Genotype contrast (I-carrier vs D/D), 200 mg — IPW-weighted | PB (<37 wk) | 1.0010 | 6,351 | 5,216 | 0 | 0.94 |
| Genotype contrast (I-carrier vs D/D), 200 mg — unweighted | EPB (<34 wk) | 1.0002 | 5,154 | 4,209 | 0 | 0.89 |
| Genotype contrast (I-carrier vs D/D), 200 mg — IPW-weighted | EPB (<34 wk) | 1.0007 | 4,552 | 3,393 | 0 | 0.90 |
| post-40-week birth (≥40 wk) — dose contrast, D/D | ≥40 wk | 1.0007 | 6,550 | 5,959 | 0 | 0.98 |
| post-40-week birth (≥40 wk) — dose contrast, I-carrier | ≥40 wk | 1.0005 | 5,943 | 4,896 | 0 | 0.95 |
| post-40-week birth (≥40 wk) — genotype contrast, 200 mg | ≥40 wk | 1.0009 | 5,923 | 4,850 | 0 | 1.00 |
| post-40-week birth (≥40 wk) — genotype contrast, 1000 mg | ≥40 wk | 1.0008 | 6,288 | 5,779 | 0 | 1.00 |
| Dose contrast (200 vs 1000 mg) — I-allele carriers (pooled) | PB (<37 wk) | 1.0008 | 5,884 | 5,396 | 0 | 0.99 |
| Dose contrast (200 vs 1000 mg) — I-allele carriers (pooled) | EPB (<34 wk) | 1.0007 | 5,018 | 4,321 | 0 | 0.92 |
| Genotype contrast (I-carrier vs D/D), 1000 mg | PB (<37 wk) | 1.0010 | 6,436 | 5,248 | 0 | 1.01 |
| Genotype contrast (I-carrier vs D/D), 1000 mg | EPB (<34 wk) | 1.0018 | 6,332 | 5,260 | 0 | 0.94 |
| Mismatched dose: D/D 1000 mg vs I-carrier 200 mg | PB (<37 wk) | 1.0006 | 6,599 | 5,814 | 0 | 0.97 |
| Mismatched dose: D/D 1000 mg vs I-carrier 200 mg | EPB (<34 wk) | 1.0006 | 5,827 | 4,881 | 0 | 0.96 |
| Matched dose: D/D 200 mg vs I-carrier 1000 mg | PB (<37 wk) | 1.0005 | 6,752 | 5,925 | 0 | 1.02 |
| Matched dose: D/D 200 mg vs I-carrier 1000 mg | EPB (<34 wk) | 1.0013 | 4,560 | 3,558 | 0 | 0.84 |
| **Summary (all 28 models):** |  | **1.0030** | **3,591** | **3,393** | **0** | **0.82**^[1]^ |

**Table B.** Prior-sensitivity analysis. Posterior probability (PP) for selected primary reported contrasts under the prior used and under a tighter and a more diffuse prior on the log-odds. N(0, σ²) denotes a Normal prior with mean 0 and variance σ² (standard deviation σ) on the logit scale.

| **Contrast (PP)** | **N(0, 2.5²)** | **N(0, 5²) *** | **N(0, 10²)** | **Max change** |
| --- | --- | --- | --- | --- |
| PB D/D dose (1000 vs 200 mg) | 0.845 | 0.846 | 0.846 | 0.001 |
| PB I/D dose (1000 vs 200 mg) | 0.977 | 0.978 | 0.978 | 0.002 |
| PB I/I dose (1000 vs 200 mg) | 0.468 | 0.469 | 0.469 | 0.000 |
| EPB D/D dose (1000 vs 200 mg) | 0.098 | 0.066 | 0.056 | 0.042 |
| EPB I/D dose (1000 vs 200 mg) | 0.965 | 0.978 | 0.982 | 0.017 |
| EPB I/I dose (1000 vs 200 mg) | 0.820 | 0.855 | 0.867 | 0.047 |
| PB I-carrier vs D/D (200 mg) | 0.957 | 0.959 | 0.960 | 0.003 |
| EPB I-carrier vs D/D (200 mg) | 0.981 | 0.990 | 0.992 | 0.011 |

** the prior used in the analysis. The largest change in any PP across the three priors is 0.05, in the sparsest early-preterm-birth strata; all well-powered contrasts change by ≤0.005. PB, preterm birth; EPB, early preterm birth.*

**Table C.** Hierarchical model sensitivity analysis.

Posterior probabilities that 1000 mg/d DHA reduced event risk compared with 200 mg/d are shown by genotype. The non-hierarchical model estimates each genotype separately, whereas the hierarchical model partially pools estimates across genotypes. PB, preterm birth; EPB, early preterm birth; DHA, docosahexaenoic acid

| Outcome | Genotype | Non-Hierarchical binomial model | Hierarchical partial-pooling model |
| --- | --- | --- | --- |
| PB (<37 wk) | D/D | 0.85 | 0.91 |
| PB (<37 wk) | I/D | 0.98 | 0.98 |
| PB (<37 wk) | I/I | 0.47 | 0.80 |
| EPB (<34 wk) | D/D | 0.07 | 0.62 |
| EPB (<34 wk) | I/D | 0.97 | 0.88 |
| EPB (<34 wk) | I/I | 0.85 | 0.82 |

**Table D**. Bayesian posteriors in each treatmentarm: unweighted vs IPW-weighted (PB, EPB). IPW, inverse-probability weighting; PB, preterm birth; EPB, early preterm birth; PP, posterior probability; ESS, effective sample size.

(a) 200 mg arm (ESS = 364.6 of 365)

| **Weighting** | **Outcome** | **PP (I allele carriers> D/D)** | **Posterior mean D/D** | **Posterior mean I-carrier** | **Posterior mean risk differences** | **Posterior risk ratio** |
| --- | --- | --- | --- | --- | --- | --- |
| Unweighted | EPB | 0.9908 | 0.0079 [0.0004, 0.0266] | 0.0427 [0.0198, 0.0732] | 0.0348 [0.0068, 0.0671] | 17.2220 [1.3455, 94.7480] |
| Unweighted | PB | 0.9597 | 0.0871 [0.0480, 0.1359] | 0.1446 [0.1008, 0.1944] | 0.0575 [-0.0078, 0.1218] | 1.7832 [0.9374, 3.1911] |
|  |  |  |  |  |  |  |
|  |  |  |  |  |  |  |
| Weighted | EPB | 0.9895 | 0.0081 [0.0004, 0.0274] | 0.0424 [0.0198, 0.0729] | 0.0342 [0.0053, 0.0669] | 20.7172 [1.2489, 102.8715] |
| Weighted | PB | 0.9614 | 0.0850 [0.0457, 0.1344] | 0.1439 [0.1002, 0.1941] | 0.0589 [-0.0062, 0.1220] | 1.8255 [0.9465, 3.3252] |

(b) 1000 mg arm (ESS = 395.9 of 396)

| **Weighting** | **Outcome** | **PP (I allele carriers> D/D)** | **Posterior mean D/D** | **Posterior mean I-carrier** | **Posterior mean risk differences** | **Posterior risk ratio** |
| --- | --- | --- | --- | --- | --- | --- |
| Unweighted | EPB | 0.0967 | 0.0272 [0.0092, 0.0543] | 0.0108 [0.0016, 0.0286] | −0.0164 [−0.0462, 0.0090] | 0.4853 [0.0534, 1.6587] |
| Unweighted | PB | 0.9107 | 0.0582 [0.0301, 0.0940] | 0.0930 [0.0564, 0.1367] | 0.0348 [−0.0168, 0.0876] | 1.7440 [0.7964, 3.4146] |
|  |  |  |  |  |  |  |
|  |  |  |  |  |  |  |
| Weighted | EPB | 0.0982 | 0.0270 [0.0090, 0.0537] | 0.0106 [0.0015, 0.0286] | −0.0164 [−0.0460, 0.0090] | 0.4909 [0.0510, 1.7341] |
| Weighted | PB | 0.9197 | 0.0573 [0.0293, 0.0949] | 0.0933 [0.0577, 0.1365] | 0.0360 [−0.0142, 0.0876] | 1.7811 [0.8292, 3.4862] |

**Table E**. Covariate balance and composition in unweighted and IPW weighted samples. Analyzed (genotyped+consented) and ESS (IPW weighted) for the 200mg arm and overall cohort. IPW, inverse-probability weighting; ESS, effective sample size; SMD, standardized mean difference; ARA, arachidonic acid; DGLA, dihomo-gamma-linolenic acid; DHA, docosahexaenoic acid.

|  | **Covariate fatty acid standardized mean differences (SMDs)** | | **Total variation distance** |
| --- | --- | --- | --- |
|  |  |  |  |
| Weighting | Baseline ARA/DGLA | Baseline %DHA | Center×Maternal race×Treatment |
| Unweighted | -0.056 | -0.045 | 0.104 |
| Weighted | -0.057 | -0.052 | 0.1056 |
|  |  |  |  |
|  | 200mg Treatment group | Total |  |
| Analyzed (S=1) | 365 | 761 |  |
| ESS (weighted) | 364.6 | 760.8 |  |

**Table F**. a) IPW sensitivity across denominator models and truncation for EPB 200mg/d. b) IPW sensitivity across denominator models and truncation for PB 200mg/d. IPW, inverse-probability weighting; EPB, early preterm birth; PB, preterm birth; PP, posterior probability; ESS, effective sample size; ARA, arachidonic acid; DGLA, dihomo-gamma-linolenic acid; DHA, docosahexaenoic acid.

| a) | **No truncation** | | | **1-99% truncation** | | | **2-98% truncation** | | |
| --- | --- | --- | --- | --- | --- | --- | --- | --- | --- |
| IPW denominator (covariates) | PP of EPB I > D/D | Posterior mean risk difference | ESS | PP of EPB I > D/D | Posterior mean risk difference | ESS | PP of EPB I > D/D | Posterior mean risk difference | ESS |
| center + maternal race | 0.9892 | 0.0341 | 365 | 0.9892 | 0.0341 | 365 | 0.9892 | 0.0341 | 365 |
|  |  |  |  |  |  |  |  |  |  |
| center + maternal race + Baseline ARA/DGLA + Baseline %DHA | 0.9862 | 0.0344 | 364.4 | 0.9908 | 0.0339 | 364.5 | 0.99 | 0.0344 | 364.5 |
|  |  |  |  |  |  |  |  |  |  |
| center x maternal race + Baseline ARA/DGLA + Baseline %DHA | 0.9872 | 0.0343 | 354 | 0.985 | 0.034 | 360.4 | 0.987 | 0.0342 | 361.1 |
|  |  |  |  |  |  |  |  |  |  |
| b) | **No truncation** | | | **1-99% truncation** | | | **2-98% truncation** | | |
| IPW denominator (covariates) | PP of PB I > D/D | Posterior mean risk difference | ESS | PP of PB I > D/D | Posterior mean risk difference | ESS | PP of PB I > D/D | Posterior mean risk difference | ESS |
| center + maternal race | 0.9673 | 0.059 | 365 | 0.9673 | 0.059 | 365 | 0.9673 | 0.059 | 365 |
|  |  |  |  |  |  |  |  |  |  |
| center + maternal race + Baseline ARA/DGLA + Baseline %DHA | 0.9645 | 0.0594 | 364.4 | 0.967 | 0.0601 | 364.5 | 0.963 | 0.0586 | 364.5 |
|  |  |  |  |  |  |  |  |  |  |
| center x maternal race + Baseline ARA/DGLA + Baseline %DHA | 0.9558 | 0.056 | 354 | 0.9495 | 0.0566 | 360.4 | 0.9548 | 0.0576 | 361.1 |

**Table G.** Genotype selection bias sensitivity tipping point analysis, Γ down-weights I-carriers by 1/Γ, “Effective % reduction” is the percent decrease in total I-carrier weight mass relative to Γ=1. ESS, effective sample size; PP, posterior probability; PB, preterm birth; EPB, early preterm birth.

|  | **PB** |  |  |  | **EPB** |  |  |  |
| --- | --- | --- | --- | --- | --- | --- | --- | --- |
| **Γ** | **ESS** | **Effective % reduction of I-carrier weight vs. baseline** | **PP of I>D/D** | **Posterior Mean** | **ESS** | **Effective % reduction of I-carrier weight vs. baseline** | **PP of I>D/D** | **Posterior Mean** |
| 1 | 364.5 | 0 | 0.9607 | 0.0590 (-0.0082–0.1219) | 364.5 | 0 | 0.988 | 0.0341 (0.0053–0.0664) |
| 1.1 | 364 | 3.2 | 0.9693 | 0.0589 (-0.0021–0.1237) | 364 | 3.2 | 0.989 | 0.0352 (0.0069–0.0694) |
| 1.25 | 361.2 | 7.6 | 0.9623 | 0.0589 (-0.0057–0.1243) | 361.2 | 7.6 | 0.9937 | 0.0352 (0.0069–0.0705) |
| 1.4 | 356.9 | 11.4 | 0.962 | 0.0589 (-0.0069–0.1259) | 356.9 | 11.4 | 0.992 | 0.0369 (0.0062–0.0714) |
| 1.5 | 353.5 | 13.7 | 0.966 | 0.0597 (-0.0039–0.1262) | 353.5 | 13.7 | 0.9923 | 0.0363 (0.0070–0.0707) |
| 1.75 | 344 | 18.8 | 0.9597 | 0.0584 (-0.0058–0.1228) | 344 | 18.8 | 0.9917 | 0.0374 (0.0083–0.0764) |
| 2 | 334 | 23.2 | 0.9677 | 0.0602 (-0.0031–0.1262) | 334 | 23.2 | 0.9937 | 0.0386 (0.0090–0.0774) |
| 3 | 297.9 | 35.6 | 0.964 | 0.0602 (-0.0054–0.1325) | 297.9 | 35.6 | 0.998 | 0.0424 (0.0098–0.0887) |
| 5 | 250.8 | 49.4 | 0.9493 | 0.0614 (-0.0112–0.1417) | 250.8 | 49.4 | 0.9957 | 0.0454 (0.0103–0.0949) |
| 7 | 222.9 | 57.1 | 0.9433 | 0.0621 (-0.0131–0.1500) | 222.9 | 57.1 | 0.9953 | 0.0459 (0.0088–0.0996) |
| 10 | 196.8 | 64.2 | 0.935 | 0.0613 (-0.0154–0.1514) | 196.8 | 64.2 | 0.9933 | 0.0466 (0.0070–0.1049) |
| 15 | 171.7 | 70.9 | 0.9083 | 0.0583 (-0.0223–0.1582) | 171.7 | 70.9 | 0.99 | 0.0461 (0.0043–0.1101) |

**Table H.** Overall observed preterm birth (PB) rate by dose and allele with posterior means, Bayesian 95% credible intervals and posterior probabilities of a dose effect

|  | **preterm birth <37 weeks, no./N (%)** | | **Posterior mean%  (95% Bayesian credible interval)^a^** | | **Bayesian posterior prob.** (1000 better than 200) |
| --- | --- | --- | --- | --- | --- |
|  | **200 mg** | **1000 mg** | **200 mg** | **1000 mg** |  |
|  |  |  |  |  |  |
| **Allele** | |  |  |  |  |
| D/D | 13/151 (8.6) | 11/191 (5.8) | 8.7 (4.7,13.6) | 5.8 (3.0,9.5) | 0.84 |
| I/D | 24/160 (15.0) | 12/153 (7.8) | 15.1 (10.1,21.0) | 7.9 (4.1,12.7) | 0.98 |
| I/I | 7/54 (13.0) | 7/52 (13.5) | 13.1 (5.8,23.3) | 13.6 (5.8,24.4) | 0.47 |
| ^a^ Bayesian logistic binomial models were fitted using the approach in Berry SM et al.^30^ This approach avoids borrowing induced type I error inflation when true effects differ between in groups. We ran four Hamiltonian Monte Carlo chains for 4,000 iterations each, using 1,000 warm up iterations for adaptation and retaining 12,000 post warmup draws for inference. | | | | | |

**Table I.** Overall observed early preterm birth (EPB) rate by dose and allele with posterior means, Bayesian 95% credible intervals and posterior probabilities of a dose effect.

|  | **Early preterm birth <34 weeks, no./N (%)** | | **Posterior mean%  (95% Bayesian credible interval)^a^** | | **Bayesian posterior prob.** (1000 better than 200) |
| --- | --- | --- | --- | --- | --- |
|  | **200 mg** | **1000 mg** | **200 mg** | **1000 mg** |  |
|  |  |  |  |  |  |
| **Allele** | |  |  |  |  |
| D/D | 1/151 (0.7) | 5/191 (2.6) | 0.8(0.04,2.7) | 2.7 (0.9,5.4) | 0.07 |
| I/D | 6/160 (3.8) | 1/153 (0.7) | 3.8(1.5,7.3) | 0. 8(0.04,2.6) | 0.98 |
| I/I | 3/54 (5.6) | 1/52 (1.9) | 5.8 (1.4,13.2) | 2.3 (0.1,7.4) | 0.86 |
| ^a^ Bayesian logistic binomial models were fitted using the approach in Berry SM et al.^30^ This approach avoids borrowing induced type I error inflation when true effects differ between in groups. We ran four Hamiltonian Monte Carlo chains for 4,000 iterations each, using 1,000 warm up iterations for adaptation and retaining 12,000 post warmup draws for inference | | | | | |

**Table J.** Black participants observed preterm birth (PB) rate by dose and allele with posterior means.

|  | **Preterm birth <37 weeks, no./N (%)** | | **Posterior mean%  (95% Bayesian credible interval)^a^** | | **Bayesian posterior prob.** (1000 better than 200) |
| --- | --- | --- | --- | --- | --- |
|  | **200 mg** | **1000 mg** | **200 mg** | **1000 mg** |  |
|  |  |  |  |  |  |
| **Allele** | |  |  |  |  |
| D/D | 1/12 (8.3) | 1/11 (9.1) | 9.3 (0.4,30.0) | 10.0 (0.5,31.5) | 0.48 |
| I/D | 10/38 (26.3) | 4/39 (10.3) | 26.6 (14.1,41.7) | 10.4 (3.1,21.8) | 0.97 |
| I/I | 5/29 (17.2) | 4/27 (14.8) | 17.7 (6.1,33.7) | 15.1 (4.7,30.2) | 0.61 |
| ^a^ Bayesian logistic binomial models were fitted using the approach in Berry SM et al.^30^ This approach avoids borrowing induced type I error inflation when true effects differ between in groups. We ran four Hamiltonian Monte Carlo chains for 4,000 iterations each, using 1,000 warm up iterations for adaptation and retaining 12,000 post warmup draws for inference | | | | | |

**Table K.** Black participants observed early preterm birth (EPB) by dose and allele with posterior means.

|  | **Early preterm birth <34 weeks, no./N (%)** | | **Posterior mean%  (95% Bayesian credible interval)^a^** | | **Bayesian posterior prob.** (1000 better than 200) |
| --- | --- | --- | --- | --- | --- |
|  | **200 mg** | **1000 mg** | **200 mg** | **1000 mg** |  |
|  |  |  |  |  |  |
| **Allele** | |  |  |  |  |
| D/D | 0/12 (0.0) | 0/11 (0.0) | 1.9 (0,12.3) | 2.0 (0,13.4) | 0.49 |
| I/D | 4/38 (10.5) | 1/39 (2.6) | 10.8 (3.3,22.0) | 3.0 (0.1,10.1) | 0.94 |
| I/I | 2/29 (6.9) | 0/27 (0.0) | 7.3 (1.0,19.1) | 1.0 (0,6.1) | 0.95 |
| ^a^ Bayesian logistic binomial models were fitted using the approach in Berry SM et al.^30^ This approach avoids borrowing induced type I error inflation when true effects differ between in groups. We ran four Hamiltonian Monte Carlo chains for 4,000 iterations each, using 1,000 warm up iterations for adaptation and retaining 12,000 post warmup draws for inference. | | | | | |

**Table L.** Observed preterm birth (PB) and early preterm birth (EPB) and arachidonic acid (ARA) to docosahexaenoic acid (DHA) or di-homo-gamma-linolenic acid (DGLA) ratio at baseline and birth by racial group (Black or Other Race), genotype (D/D, I/D, I/I) and DHA dose (200 mg or 1000 mg). 25/ 156 (16.0%) of Black participants experienced PB, compared to 49/ 605 (8.1%) of Other Races (PP=0.99), 7/ 156 (4.5%) of Black participants experienced EPB, compared to 10/ 605 (1.7%) of Other Races (PP=0.97), PP, posterior probability.

|  | **Black** | | | | | | **Other Race** | | | | | |
| --- | --- | --- | --- | --- | --- | --- | --- | --- | --- | --- | --- | --- |
|  | **D/D** | | **I/D** | | **I/I** | | **D/D** | | **I/D** | | **I/I** | |
|  | **200mg** | **1000mg** | **200mg** | **1000mg** | **200mg** | **1000mg** | **200mg** | **1000mg** | **200mg** | **1000mg** | **200mg** | **1000mg** |
|  | n=12 | n=11 | n=38 | n=39 | n=29 | n=27 | n=139 | n=180 | n=122 | n=114 | n=25 | n=25 |
|  |  |  |  |  |  |  |  |  |  |  |  |  |
| PB<34 wks | 0% | 0% | 10.5% | 2.6% | 6.9% | 0% | 0.7% | 2.8% | 1.6% | 0% | 4.0% | 4.0% |
|  |  |  |  |  |  |  |  |  |  |  |  |  |
| PB <37 wks | 8.3% | 9.1% | 26.3% | 10.3% | 17.2% | 14.8% | 8.6% | 5.6% | 11.5% | 7.0% | 8.0% | 12.0% |
|  |  |  |  |  |  |  |  |  |  |  |  |  |
| ARA/DHA Baseline | 3.5 | 3.8 | 3.5 | 3.9 | 3.5 | 3.7 | 3.1 | 3.2 | 3.2 | 3.2 | 3.1 | 3.0 |
|  |  |  |  |  |  |  |  |  |  |  |  |  |
| ARA/DHA Birth | 3.1 | 2.8 | 3.1 | 2.9 | 2.9 | 2.8 | 2.3 | 1.8 | 2.4 | 1.8 | 2.2 | 1.7 |
|  |  |  |  |  |  |  |  |  |  |  |  |  |
| ARA/DGLA Baseline | 9.1 | 11.1 | 10.9 | 10.4 | 10.7 | 11.8 | 7.8 | 7.6 | 8.9 | 9.0 | 9.6 | 9.5 |
|  |  |  |  |  |  |  |  |  |  |  |  |  |
| ARA/DGLA Birth | 8.7 | 11.0 | 9.2 | 10.2 | 8.8 | 9.9 | 7.0 | 7.1 | 8.4 | 8.5 | 9.7 | 10.1 |
|  |  |  |  |  |  |  |  |  |  |  |  |  |

**Table M. Baseline RBC-PL fatty acid composition (% of total fatty acids) by FADS Indel genotype**

| **Fatty acid** | **D/D (n=342)** | **I/D (n=313)** | **I/I (n=106)** | **All (n=761)** |
| --- | --- | --- | --- | --- |
| 14:0 | 0.30 ± 0.12 | 0.29 ± 0.12 | 0.26 ± 0.11 | 0.29 ± 0.12 |
| 16:0 | 20.67 ± 1.23 | 20.52 ± 1.22 | 20.56 ± 1.10 | 20.60 ± 1.21 |
| 17:0 | 0.26 ± 0.09 | 0.25 ± 0.09 | 0.25 ± 0.09 | 0.25 ± 0.09 |
| 18:0 | 10.75 ± 2.15 | 10.73 ± 2.29 | 10.96 ± 2.16 | 10.77 ± 2.21 |
| 20:0 | 0.09 ± 0.07 | 0.09 ± 0.06 | 0.08 ± 0.05 | 0.09 ± 0.06 |
| 22:0 | 0.11 ± 0.07 | 0.11 ± 0.06 | 0.10 ± 0.09 | 0.11 ± 0.07 |
| 24:0 | 0.22 ± 0.16 | 0.21 ± 0.15 | 0.21 ± 0.18 | 0.21 ± 0.16 |
| 14:1 | 0.18 ± 0.08 | 0.17 ± 0.09 | 0.15 ± 0.07 | 0.17 ± 0.08 |
| 16:1 | 0.28 ± 0.12 | 0.26 ± 0.11 | 0.23 ± 0.10 | 0.27 ± 0.11 |
| 18:1n7 | 1.35 ± 0.39 | 1.35 ± 0.40 | 1.36 ± 0.41 | 1.35 ± 0.40 |
| 18:1n9 | 10.29 ± 1.03 | 10.09 ± 1.11 | 9.93 ± 1.11 | 10.15 ± 1.08 |
| 24:1 | 0.21 ± 0.14 | 0.22 ± 0.15 | 0.22 ± 0.16 | 0.22 ± 0.15 |
| 16:1t | 0.11 ± 0.04 | 0.11 ± 0.04 | 0.10 ± 0.05 | 0.11 ± 0.04 |
| 18:1t | 0.50 ± 0.18 | 0.48 ± 0.23 | 0.45 ± 0.19 | 0.48 ± 0.20 |
| 18:2n6t | 0.37 ± 0.21 | 0.36 ± 0.20 | 0.34 ± 0.17 | 0.36 ± 0.20 |
| 18:2n6 (LA) | 14.15 ± 2.04 | 13.63 ± 1.83 | 13.46 ± 2.10 | 13.84 ± 1.98 |
| 18:3n6 (GLA) | 0.08 ± 0.05 | 0.08 ± 0.06 | 0.07 ± 0.03 | 0.08 ± 0.05 |
| 20:2n6 | 0.35 ± 0.08 | 0.33 ± 0.07 | 0.33 ± 0.07 | 0.34 ± 0.08 |
| 20:3n6 (DGLA) | 2.57 ± 0.68 | 2.19 ± 0.45 | 1.97 ± 0.35 | 2.33 ± 0.60 |
| 20:4n6 (ARA) | 18.65 ± 2.47 | 19.64 ± 2.72 | 19.97 ± 2.56 | 19.24 ± 2.64 |
| 22:4n6 (DTA) | 3.86 ± 0.86 | 4.03 ± 0.86 | 4.08 ± 0.90 | 3.96 ± 0.87 |
| 22:5n6 (DPA n-6) | 0.87 ± 0.24 | 0.89 ± 0.24 | 0.90 ± 0.25 | 0.88 ± 0.24 |
| 18:3n3 (ALA) | 0.23 ± 0.09 | 0.22 ± 0.09 | 0.20 ± 0.08 | 0.22 ± 0.09 |
| 20:5n3 (EPA) | 0.51 ± 0.29 | 0.52 ± 0.32 | 0.51 ± 0.32 | 0.52 ± 0.31 |
| 22:5n3 (DPA n-3) | 2.18 ± 0.42 | 2.22 ± 0.40 | 2.17 ± 0.40 | 2.19 ± 0.41 |
| 22:6n3 (DHA) | 6.25 ± 1.70 | 6.32 ± 1.72 | 6.41 ± 1.67 | 6.30 ± 1.70 |

**Table N.** Outcome tilted sensitivity (worst case scenario) analysis. Within I allele carriers, outcomes are tilted adversely (term birth × Γ; PB/EPB × 1/Γ) and weights re-centered. PB, preterm birth; EPB, early preterm birth; ESS, effective sample size; PP, posterior probability.

|  | **PB** |  |  |  | **EPB** |  |  |  |
| --- | --- | --- | --- | --- | --- | --- | --- | --- |
| **Γ** | **ESS** | **Effective % reduction of I-carrier weight vs. baseline** | **PP of I>D/D** | **Posterior Mean** | **ESS** | **Effective % reduction of I-carrier weight vs. baseline** | **PP of I>D/D** | **Posterior Mean** |
| 1 | 364.5 | 0 | 0.9607 | 0.0590 (-0.0082–0.1219) | 364.5 | 0 | 0.988 | 0.0341 (0.0053–0.0664) |
| 1.25 | 360.7 | -0.1 | 0.697 | 0.0152 (-0.0454–0.0720) | 363.3 | 0 | 0.939 | 0.0199 (-0.0073–0.0466) |
| 1.4 | 357.2 | -0.1 | 0.493 | -0.0011 (-0.0622–0.0545) | 362.2 | 0 | 0.899 | 0.0148 (-0.0108–0.0409) |
| 1.5 | 355 | -0.1 | 0.362 | -0.0103 (-0.0671–0.0463) | 361.6 | 0 | 0.8653 | 0.0118 (-0.0112–0.0364) |
| 1.75 | 350.2 | -0.1 | 0.17 | -0.0271 (-0.0856–0.0270) | 360.2 | 0 | 0.7683 | 0.0071 (-0.0163–0.0297) |
| 2 | 346.4 | -0.1 | 0.0743 | -0.0384 (-0.0944–0.0134) | 359.1 | 0 | 0.6983 | 0.0039 (-0.0175–0.0232) |
| 3 | 338 | -0.2 | 0.0043 | -0.0603 (-0.1127–-0.0137) | 356.8 | 0 | 0.4307 | -0.0018 (-0.0212–0.0138) |
| 5 | 333 | -0.2 | 0.001 | -0.0724 (-0.1225–-0.0308) | 355.5 | -0.1 | 0.2273 | -0.0050 (-0.0248–0.0080) |

**Table O. Baseline characteristics by genotype.** HS, high school; GED, General Educational Development; Hx, history; PTB, preterm birth; ePTB, early preterm birth; Tx, treatment; DHA, docosahexaenoic acid; BMI, body mass index.

|  | **Total Sample** | **D/D** | **I/D** | **I/I** |
| --- | --- | --- | --- | --- |
| **Site** | n (%) | | | |
| The University of Kansas Medical Center | 389 (51.1%) | 211 (61.7%) | 148 (47.3%) | 30 (28.3%) |
| University of Cincinnati Medical Center | 183 (24%) | 45 (13.2%) | 88 (28.1%) | 50 (47.2%) |
| Ohio State University | 189 (24.8%) | 86 (25.1%) | 77 (24.6%) | 26 (24.5%) |
| **Marital Status** |  |  |  |  |
| Unmarried/Single | 253 (33.2%) | 78 (22.8%) | 118 (37.7%) | 57 (53.8%) |
| Married/Partnered | 487 (64%) | 255 (74.6%) | 188 (60.1%) | 44 (41.5%) |
| Separated | 10 (1.3%) | 6 (1.8%) | 2 (0.6%) | 2 (1.9%) |
| Divorced | 11 (1.4%) | 3 (0.9%) | 5 (1.6%) | 3 (2.8%) |
| **Race/Ethnicity** |  |  |  |  |
| American Indian or Alaskan Native | 4 (0.5%) | 2 (0.6%) | 1 (0.3%) | 1 (0.9%) |
| Asian | 17 (2.2%) | 2 (0.6%) | 7 (2.2%) | 8 (7.5%) |
| Biracial: Asian, White | 4 (0.5%) | 1 (0.3%) | 3 (1%) | 0 (0%) |
| Biracial: Black, White | 8 (1.1%) | 5 (1.5%) | 1 (0.3%) | 2 (1.9%) |
| Black or African American | 156 (20.5%) | 23 (6.7%) | 77 (24.6%) | 56 (52.8%) |
| Hispanic | 190 (25%) | 133 (38.9%) | 50 (16%) | 7 (6.6%) |
| Multiracial: Black, Asian, White | 1 (0.1%) | 0 (0%) | 0 (0%) | 1 (0.9%) |
| Multiracial: Black, Native American, White | 2 (0.3%) | 1 (0.3%) | 1 (0.3%) | 0 (0%) |
| Native Hawaiian or other Pacific Islander | 1 (0.1%) | 1 (0.3%) | 0 (0%) | 0 (0%) |
| Other | 4 (0.5%) | 0 (0%) | 3 (1%) | 1 (0.9%) |
| White | 374 (49.1%) | 174 (50.9%) | 170 (54.3%) | 30 (28.3%) |
| **Maternal Education** |  |  |  |  |
| less than HS | 134 (17.6%) | 84 (24.6%) | 36 (11.5%) | 14 (13.2%) |
| HS grad/GED | 161 (21.2%) | 58 (17%) | 76 (24.3%) | 27 (25.5%) |
| some college or tech school | 141 (18.5%) | 50 (14.6%) | 67 (21.4%) | 24 (22.6%) |
| bachelor's degree | 167 (21.9%) | 73 (21.3%) | 69 (22%) | 25 (23.6%) |
| master's degree | 106 (13.9%) | 52 (15.2%) | 43 (13.7%) | 11 (10.4%) |
| doctorate | 52 (6.8%) | 25 (7.3%) | 22 (7%) | 5 (4.7%) |
| **Household Income** |  |  |  |  |
| unknown | 18 (2.4%) | 7 (2%) | 7 (2.2%) | 4 (3.8%) |
| <$15,000 | 162 (21.3%) | 45 (13.2%) | 84 (26.8%) | 33 (31.1%) |
| $15,000-$24,999 | 112 (14.7%) | 61 (17.8%) | 38 (12.1%) | 13 (12.3%) |
| $25,000-$49,999 | 134 (17.6%) | 72 (21.1%) | 44 (14.1%) | 18 (17%) |
| $50,000-$99,999 | 135 (17.7%) | 56 (16.4%) | 60 (19.2%) | 19 (17.9%) |
| $100,000-$150,000 | 121 (15.9%) | 62 (18.1%) | 52 (16.6%) | 7 (6.6%) |
| ≥$150,000 | 79 (10.4%) | 39 (11.4%) | 28 (8.9%) | 12 (11.3%) |
|  |  |  |  |  |
| **Ever Smoke (yes)** | 194 (25.5%) | 76 (22.2%) | 94 (30%) | 24 (22.6%) |
| smoke 6mo prior (yes) | 98 (12.9%) | 33 (9.6%) | 51 (16.3%) | 14 (13.2%) |
| current smoker (yes) | 40 (5.3%) | 10 (2.9%) | 24 (7.7%) | 6 (5.7%) |
| **Pregnancy Hx** |  |  |  |  |
| primagravida | 219 (28.8%) | 94 (27.5%) | 106 (33.9%) | 19 (17.9%) |
| prior PTB (yes) | 104 (13.7%) | 47 (13.7%) | 42 (13.4%) | 15 (14.2%) |
| prior ePTB (yes) | 45 (5.9%) | 18 (5.3%) | 19 (6.1%) | 8 (7.5%) |
| **Tx Group** |  |  |  |  |
| 200mg | 365 (48%) | 151 (44.2%) | 160 (51.1%) | 54 (50.9%) |
| 1000mg | 396 (52%) | 191 (55.8%) | 153 (48.9%) | 52 (49.1%) |
|  | mean ± SD | | | |
| Age at enrollment | 30.3 ± 5.7 | 30.7 ± 5.5 | 30.1 ± 5.8 | 29.6 ± 5.6 |
| Baseline DHA status | 6.3 ± 1.7 | 6.3 ± 1.7 | 6.3 ± 1.7 | 6.4 ± 1.7 |
| Pre-pregnancy BMI | 28.1 ± 7.2 | 27.4 ± 6.6 | 28.4 ± 7.4 | 29.7 ± 7.8 |

**Table P. Preterm birth rates by socioeconomic category and genotype**. BMI, body mass index.

|  | **D/D** | **I/D** | **I/I** |
| --- | --- | --- | --- |
| **Income <$15k** | **2/45 (4%)** | **14/84 (17%)** | **4/33 (12%)** |
| **Income ≥$15k** | **22/290 (8%)** | **22/222 (10%)** | **9/69 (13%)** |
| **Unmarried** | **8/87 (9%)** | **15/125 (12%)** | **11/62 (18%)** |
| **Married** | **16/255 (6%)** | **21/188 (11%)** | **3/44 (7%)** |
| **Education <bachelor** | **17/192 (9%)** | **24/179 (13%)** | **11/65 (17%)** |
| **Education ≥bachelor** | **7/150 (5%)** | **12/134 (9%)** | **3/41 (7%)** |
| **Ever smoker** | **7/76 (9%)** | **13/94 (14%)** | **2/24 (8%)** |
| **Never smoker** | **17/266 (6%)** | **23/219 (11%)** | **12/82 (15%)** |
| **Obese (BMI≥30)** | **8/93 (9%)** | **18/114 (16%)** | **8/48 (17%)** |
| **Non-obese** | **16/246 (7%)** | **17/192 (9%)** | **6/58 (10%)** |

**Text A**

**Sensitivity analysis**

From the representative sensitivity analysis using IPW, we found that weighing had minimal impact on the effective sample sizes in the overall group, and the 200mg/d dose group (ESS=760.8 of 761 overall, ESS=364.3 of 365 for 200mg/d). Within the 200mg/d group, the total variation distance is 0.10 in the unweighted group vs. 0.11 in the weighted group, and the standard mean differences of baseline ARA/DGLA and baseline %DHA are both <0.06. PPs are virtually identical between the unweighted and weighted group: PP=0.960 vs. PP=0.961 for PB, respectively, and PP=0.991 vs. PP=0.989, respectively, for EPB, indicating that it is unlikely to introduce biased genotype-related outcomes when only analyzing consented participants (761/ 1032). (**Tables F and G**) In addition, modification of the denominator model and truncation rules had a minimal impact on the outcome above. (**Table H**) In the tipping point analysis, progressively downweighing the I allele carriers alone did not push the PP of PB or EPB below 0.9 even at Γ=15, equivalent to removing 71% of effective weight from I allele carriers (PP=0.91 for PB, PP=0.99 for EPB) (**Table I**). The outcome-tilted analysis indicated that at Γ = 1.1, the PP for PB dropped below 0.9. This corresponds to approximately 11% fewer weighted PBs among I allele carriers, equivalent to 3–4 fewer preterm births among the 31 observed PB cases in I allele carriers, relative to term births. For EPB, the threshold occurred at Γ=1.40, implying that 49% fewer weighted I-carrier EPBs, about 4-5 of the 9 observed EPBs.

1. E-BFMI has no practical upper-bound warning threshold; concern arises for low values, typically <0.3, which indicate inefficient exploration of the marginal energy distribution. In our models, all E-BFMI values were well above this threshold, supporting adequate HMC energy exploration.
