# Supplementary material for: Genotype-informed docosahexaenoic acid dosing based on the FADS Indel to reduce preterm and prolonged gestation: A secondary analysis of the ADORE randomized trial
Source: PLoS Med. 2026 Oct 8;23(10):e1005274. doi: 10.1371/journal.pmed.1005274 (PMC13660990; doi:10.1371/journal.pmed.1005274)
Supplement: Supplementary file 6 [file pmed.1005274.s014.docx]

*Note: Paragraph numbers (¶) refer to narrative paragraphs within each section; figures and tables are embedded inline in the Results and are cited by label (e.g., Fig 2, Table 1), not counted in the ¶ numbering.*

|  | Item No | Recommendation | Location (§ / ¶) |
| --- | --- | --- | --- |
| **Title and abstract** | 1 | (*a*) Indicate the study’s design with a commonly used term in the title or the abstract | Title; Abstract—Methods. |
|  |  | (*b*) Provide in the abstract an informative and balanced summary of what was done and what was found | Abstract, ¶1–4 (Background, Methods, Results, Conclusion). |
| Introduction | | | |
| Background/rationale | 2 | Explain the scientific background and rationale for the investigation being reported | §1 Background, ¶1–5. |
| Objectives | 3 | State specific objectives, including any prespecified hypotheses | §1 Background, ¶5; Abstract—Background & Methods; §2.3 Outcomes, ¶1. |
| Methods | | | |
| Study design | 4 | Present key elements of study design early in the paper | §2.1 Study Design & Participants, ¶1; Title; Abstract—Methods; §1 Background, ¶5. |
| Setting | 5 | Describe the setting, locations, and relevant dates, including periods of recruitment, exposure, follow-up, and data collection | §2.1 Study Design & Participants, ¶1–2; §2.2 Procedures, ¶2; §3 Results, ¶1. |
| Participants | 6 | (*a*) *Cohort study*—Give the eligibility criteria, and the sources and methods of selection of participants. Describe methods of follow-up  *Case-control study*—Give the eligibility criteria, and the sources and methods of case ascertainment and control selection. Give the rationale for the choice of cases and controls  *Cross-sectional study*—Give the eligibility criteria, and the sources and methods of selection of participants | §2.1 Study Design & Participants, ¶2; §2.2 Procedures, ¶1; §3 Results, ¶1; Fig 1. [Case-control / cross-sectional: N/A.] |
|  |  | (*b*) *Cohort study*—For matched studies, give matching criteria and number of exposed and unexposed  *Case-control study*—For matched studies, give matching criteria and the number of controls per case | N/A (unmatched). Group sizes: §3 Results, ¶1; Tables 1–2. |
| Variables | 7 | Clearly define all outcomes, exposures, predictors, potential confounders, and effect modifiers. Give diagnostic criteria, if applicable | §2.3 Outcomes, ¶1; §1 Background, ¶1 & ¶4; §2.1 Study Design & Participants, ¶2; §2.2 Procedures, ¶2; §2.4 Statistical Analysis, ¶1 & ¶4; §4 Discussion, ¶14. |
| Data sources/ measurement | 8* | For each variable of interest, give sources of data and details of methods of assessment (measurement). Describe comparability of assessment methods if there is more than one group | §2.2 Procedures, ¶1–3; §2.1 Study Design & Participants, ¶2. |
| Bias | 9 | Describe any efforts to address potential sources of bias | §2.2 Procedures, ¶2; §2.1 Study Design & Participants, ¶1; §2.4 Statistical Analysis, ¶1, ¶3 & ¶4; §4 Discussion, ¶7, ¶13 & ¶14. |
| Study size | 10 | Explain how the study size was arrived at | §2.1 Study Design & Participants, ¶2; §2.2 Procedures, ¶1; §3 Results, ¶1. |
| Quantitative variables | 11 | Explain how quantitative variables were handled in the analyses. If applicable, describe which groupings were chosen and why | §2.4 Statistical Analysis, ¶1 & ¶4; §2.3 Outcomes, ¶1; §1 Background, ¶1. |
| Statistical methods | 12 | (*a*) Describe all statistical methods, including those used to control for confounding | §2.4 Statistical Analysis, ¶1, ¶2 & ¶4. |
|  |  | (*b*) Describe any methods used to examine subgroups and interactions | §2.4 Statistical Analysis, ¶1 & ¶3. |
|  |  | (*c*) Explain how missing data were addressed | §2.4 Statistical Analysis, ¶1 & ¶3; §2.2 Procedures, ¶1. |
|  |  | (*d*) *Cohort study*—If applicable, explain how loss to follow-up was addressed  *Case-control study*—If applicable, explain how matching of cases and controls was addressed  *Cross-sectional study*—If applicable, describe analytical methods taking account of sampling strategy | §2.4 Statistical Analysis, ¶1 & ¶3; §2.2 Procedures, ¶1. [Case-control / cross-sectional: N/A.] |
|  |  | (*e*) Describe any sensitivity analyses | §2.4 Statistical Analysis, ¶1 & ¶3; §3 Results, ¶12; §4 Discussion, ¶13 & ¶14. |

| Results | | | |
| --- | --- | --- | --- |
| Participants | 13* | (a) Report numbers of individuals at each stage of study—eg numbers potentially eligible, examined for eligibility, confirmed eligible, included in the study, completing follow-up, and analysed | §3 Results, ¶1; §2.2 Procedures, ¶1; Fig 1. |
|  |  | (b) Give reasons for non-participation at each stage | §2.2 Procedures, ¶1; §2.1 Study Design & Participants, ¶2; Fig 1. |
|  |  | (c) Consider use of a flow diagram | Fig 1; §2.2 Procedures, ¶1. |
| Descriptive data | 14* | (a) Give characteristics of study participants (eg demographic, clinical, social) and information on exposures and potential confounders | Tables 1–2; §3 Results, ¶1 & ¶8–10; Table S15; §4 Discussion, ¶14. |
|  |  | (b) Indicate number of participants with missing data for each variable of interest | §2.4 Statistical Analysis, ¶1 & ¶3; §2.2 Procedures, ¶1; §3 Results, ¶11. |
|  |  | (c) *Cohort study*—Summarise follow-up time (eg, average and total amount) | §2.1 Study Design & Participants, ¶2; §3 Results, ¶1; Figs 2–4. |
| Outcome data | 15* | *Cohort study*—Report numbers of outcome events or summary measures over time | §3 Results, ¶2–8 & ¶11; Table 2; Figs 2–4; §4 Discussion, ¶3. [Case-control / cross-sectional: N/A.] |
|  |  | *Case-control study—*Report numbers in each exposure category, or summary measures of exposure | N/A — cohort-type analysis (see Item 15 cohort row). |
|  |  | *Cross-sectional study—*Report numbers of outcome events or summary measures | N/A — cohort-type analysis (see Item 15 cohort row). |
| Main results | 16 | (*a*) Give unadjusted estimates and, if applicable, confounder-adjusted estimates and their precision (eg, 95% confidence interval). Make clear which confounders were adjusted for and why they were included | §3 Results, ¶2–9; §2.4 Statistical Analysis, ¶3 & ¶4; Figs 2–4; Table 2. |
|  |  | (*b*) Report category boundaries when continuous variables were categorized | §1 Background, ¶1; §2.3 Outcomes, ¶1; §3 Results, ¶6; §2.4 Statistical Analysis, ¶1 & ¶4. |
|  |  | (*c*) If relevant, consider translating estimates of relative risk into absolute risk for a meaningful time period | §3 Results, ¶2–8; Abstract—Results. |
| Other analyses | 17 | Report other analyses done—eg analyses of subgroups and interactions, and sensitivity analyses | §3 Results, ¶4, ¶6, ¶8 & ¶12; §4 Discussion, ¶3 & ¶14; §2.4 Statistical Analysis, ¶1 & ¶3. |
| Discussion | | | |
| Key results | 18 | Summarise key results with reference to study objectives | §4 Discussion, ¶1; §5 Conclusions, ¶1; Abstract—Results & Conclusion. |
| Limitations | 19 | Discuss limitations of the study, taking into account sources of potential bias or imprecision. Discuss both direction and magnitude of any potential bias | §4 Discussion, ¶13 & ¶14; §3 Results, ¶5 & ¶12. |
| Interpretation | 20 | Give a cautious overall interpretation of results considering objectives, limitations, multiplicity of analyses, results from similar studies, and other relevant evidence | §4 Discussion, ¶2, ¶3, ¶7, ¶9 & ¶11; §5 Conclusions, ¶1. |
| Generalisability | 21 | Discuss the generalisability (external validity) of the study results | §4 Discussion, ¶5–6, ¶10 & ¶12. |
| Other information | | | |
| Funding | 22 | Give the source of funding and the role of the funders for the present study and, if applicable, for the original study on which the present article is based | §6 Declarations—Funding; Authors' contributions; Title page—Funding. |
