# Supplementary material for: Genotype-informed docosahexaenoic acid dosing based on the FADS Indel to reduce preterm and prolonged gestation: A secondary analysis of the ADORE randomized trial
Source: PLoS Med. 2026 Oct 8;23(10):e1005274. doi: 10.1371/journal.pmed.1005274 (PMC13660990; doi:10.1371/journal.pmed.1005274)
Supplement: Supplementary file 7 [file pmed.1005274.s017.docx]

Our current Bayesian statistics were modeled analogously to the treatment- specific Bayesian binomial model used for primary efficacy analysis in the ADORE trial. It is a Bayesian non-hierarchical logistic-binomial model with a weakly informative prior [Normal(0,5)] on the log odds to avoid boundary issues. It estimates the PP of genotype/ treatment specific events independently within each genotype/ treatment arm, without pooling.

Example R code below:

```
library(rstan)

if (!requireNamespace("rstudioapi", quietly = TRUE)) {
  options(mc.cores = 1)
} else {
  options(mc.cores = parallel::detectCores())
}
rstan_options(auto_write = TRUE)

stan_code_nonhier <- "
data { int<lower=0> trials[2]; int<lower=0> successes[2]; }
parameters { real a; real b; }
model {
  a ~ normal(0,5);
  b ~ normal(0,5);
  successes[1] ~ binomial(trials[1], inv_logit(a));
  successes[2] ~ binomial(trials[2], inv_logit(b));
}
generated quantities {
  real p_200 = inv_logit(a);
  real p_1000 = inv_logit(b);
  real RD_200vs1000 = p_200 - p_1000;
  real RR_200vs1000 = p_200 / fmax(p_1000, 1e-12);
  int  RD_pos = RD_200vs1000 > 0;
  int  RR_gt1 = RR_200vs1000 > 1;
}
"

y_200   <- 3
n_200   <- 54
y_1000  <- 1
n_1000  <- 52

dlist <- list(
  trials    = as.integer(c(n_200, n_1000)),
  successes = as.integer(c(y_200, y_1000))
)

fit_nonhier <- stan(model_code = stan_code_nonhier, data = dlist,
                    iter = 4000, warmup = 1000, chains = 4, seed = 2025,
                    control = list(adapt_delta = 0.99, max_treedepth = 14))

print(fit_nonhier,
      pars = c("p_200","p_1000","RD_200vs1000","RR_200vs1000"),
      probs = c(0.025,0.975), digits_summary = 4)

dr <- rstan::extract(fit_nonhier)
mean(dr$RD_200vs1000 > 0)
mean(dr$RR_200vs1000 > 1)
```

Bayesian hierarchical logistic model

Example R code below:

```
library(rstan)

successes <- c(1, 3)
trials    <- c(151, 54)
n_groups  <- length(successes)
data_list <- list(n_groups = n_groups,
                  trials   = trials,
                  successes= successes)

stan_code_hier <- "
data {
  int<lower=0> n_groups;
  int<lower=0> trials[n_groups];
  int<lower=0> successes[n_groups];
}
parameters {
  real mu;
  real<lower=0> tau2;
  vector[n_groups] theta;
}
transformed parameters {
  real<lower=0> sigma = sqrt(tau2);
  real<lower=0,upper=1> p[n_groups];
  for (i in 1:n_groups)
    p[i] = inv_logit(theta[i]);
}
model {
  mu   ~ normal(0, 10);
  tau2 ~ inv_gamma(2, 0.1);
  for (i in 1:n_groups)
    theta[i] ~ normal(mu, sigma);
  for (i in 1:n_groups)
    successes[i] ~ binomial(trials[i], p[i]);
}
generated quantities {
  real posterior_prob_1000_better = step(p[2] - p[1]);
}
"

fit_hier <- stan(model_code = stan_code_hier,
                 data       = data_list,
                 iter       = 10000,
                 warmup     = 1000,
                 chains     = 4,
                 seed       = 123,
                 control    = list(adapt_delta = 0.99, max_treedepth = 20))

print(fit_hier,
      pars           = c("p[1]", "p[2]", "posterior_prob_1000_better"),
      probs          = c(0.025, 0.975),
      digits_summary = 4)
```

Sensitivity analysis:

We conducted a sensitivity analysis to assess potential bias introduced by excluding participants who did not consent or lacked genotype data using stabilized inverse probability weights (IPW) within the 200 mg treatment group. The inclusion probability was modeled with logistic regression, using center, maternal race, baseline ARA/DGLA, and baseline %DHA levels in the denominator, and center and maternal race in the numerator. Stabilized weights were truncated at the 1st–99th percentiles and incorporated into the Bayesian non-hierarchical logistic-binomial models. To assess robustness, we explored alternative denominator models (center + race; center + race + baseline ARA/DGLA + baseline %DHA; center×race interaction + baseline ARA/DGLA + baseline %DHA) and truncations (none, 1st–99th, 2nd–98th percentiles). A tipping point analysis was conducted through incrementally introducing selection bias on genotypes only by down-weighing I allele carriers by 1/Γ (Γ>1) while D/Ds remained at 1. The stratum is then centered to ensure the stratum mean stayed at 1. We then conducted an outcome tilted analysis within the I allele carriers that multiplied I allele term birth by Γ, and I allele PB/EPB by 1/ Γ, followed by the recentering of the stratum. The Bayesian model described above is refitted at each setting to record when the PP of I allele carriers are more likely to have PB or EPB compared to D/D genotypes fall below 0.9.

Full R code below:

```
knitr::opts_chunk$set(
  echo = TRUE,
  eval = FALSE
)
library(readxl)

library(dplyr)

library(rstan)

rstan_options(auto_write = TRUE)

if (!requireNamespace("rstudioapi", quietly = TRUE)) {

  options(mc.cores = 1)

} else {

  options(mc.cores = parallel::detectCores())

}

set.seed(2025)

path_xlsx  <- "C:/Users/Kevin/OneDrive - The University of Texas at Austin/1053 consent used for IPW.xlsx"

sheet_name <- "Sheet2"

align_rows <- FALSE   # set TRUE to force unweighted to use only rows that also have weights

df <- read_xlsx(path_xlsx, sheet = sheet_name) |> as.data.frame()

names(df) <- trimws(names(df))

ga_candidates <- grep("^gestAgeBirth(\\.\\.\\d+)?$", names(df), value = TRUE)

if (length(ga_candidates) == 0) stop("No gestAgeBirth column found.")

if ("gestAgeBirth" %in% ga_candidates) {

  col_ga <- "gestAgeBirth"

} else {

  get_idx <- function(x) as.integer(sub(".*\\.\\.(\\d+)$", "\\1", x))

  idx <- get_idx(ga_candidates)

  col_ga <- ga_candidates[order(idx)][1]

}

message("Using GA column: ", col_ga)

col_consent <- "Optional genetic research consent obtained"   # "Yes"/"No"

col_treat   <- "Treatment"

col_center  <- if ("Center" %in% names(df)) "Center" else if ("Center " %in% names(df)) "Center " else NA

if (is.na(col_center)) stop("Center column not found (Center / Center ).")

col_race    <- "maternal_race2"

col_geno    <- "Genotype"

col_FA1     <- "ARA/DGLA BL"

col_FA2     <- "a_2_6n3__corr_BL"   # NOTE: aOmega3 is intentionally NOT used

need <- c(col_consent, col_treat, col_center, col_race, col_ga, col_geno, col_FA1, col_FA2)

if (!all(need %in% names(df))) {

  miss <- need[!(need %in% names(df))]

  stop("Missing required columns: ", paste(miss, collapse = ", "),

       "\nAvailable: ", paste(names(df), collapse = " | "))

}

is_200mg <- function(x){

  if (is.numeric(x)) x == 200 else {

    xl <- tolower(as.character(x)); startsWith(xl, "200") | xl == "200mg" | xl == "200"

  }

}

clean_geno <- function(s){

  s <- toupper(trimws(as.character(s))); s <- gsub(" ", "", s)

  s <- gsub("^II$", "I/I", s); s <- gsub("^ID$", "I/D", s); s <- gsub("^DD$", "D/D", s); s

}

df <- df |>

  mutate(

    consent_bin = case_when(

      tolower(trimws(.data[[col_consent]])) == "yes" ~ 1L,

      tolower(trimws(.data[[col_consent]])) == "no"  ~ 0L,

      TRUE ~ NA_integer_

    ),

    geno_clean = clean_geno(.data[[col_geno]]),

    icarrier   = as.integer(geno_clean %in% c("I/I", "I/D")),

    DD         = geno_clean == "D/D",

    GA_wks     = as.numeric(.data[[col_ga]]),

    PB         = as.integer(GA_wks < 37),

    EPB        = as.integer(GA_wks < 34),

    dose200    = is_200mg(.data[[col_treat]])

  )

df$center <- factor(df[[col_center]])

df$maternal_race2 <- factor(df[[col_race]])

df$FA1 <- df[[col_FA1]]

df$FA2 <- df[[col_FA2]]

unw_set_base <- df |>

  filter(consent_bin == 1,

         dose200,

         !is.na(GA_wks),

         !is.na(geno_clean))

df200 <- df |>

  filter(dose200, !is.na(GA_wks))

den_fit <- glm(consent_bin ~ center + maternal_race2 + FA1 + FA2,

               data = df200, family = binomial())

num_fit <- glm(consent_bin ~ center + maternal_race2,

               data = df200, family = binomial())

df200 <- df200 |>

  mutate(

    p_den = plogis(predict(den_fit, newdata = df200)),

    p_num = plogis(predict(num_fit, newdata = df200)),

    ipw   = p_num / p_den

  )

lo <- quantile(df200$ipw[df200$consent_bin == 1], 0.01, na.rm = TRUE)

hi <- quantile(df200$ipw[df200$consent_bin == 1], 0.99, na.rm = TRUE)

df200 <- df200 |>

  mutate(ipw_trunc = ifelse(consent_bin == 1,

                            pmin(pmax(ipw, lo), hi),

                            NA_real_))

wt_set <- df200 |>

  filter(consent_bin == 1,

         !is.na(ipw_trunc),

         !is.na(GA_wks),

         !is.na(geno_clean))

if (align_rows) {

  key_cols <- c("Patient Study ID", "patientID", "Sample ID")

  key_cols <- key_cols[key_cols %in% names(df)]  # keep existing keys

  if (length(key_cols) == 0) {

    warning("align_rows=TRUE but no obvious ID column found; falling back to value-based match.")

    unw_set <- unw_set_base %>%

      semi_join(wt_set, by = c(col_center, col_race, col_treat, col_ga, col_geno))

  } else {

    unw_set <- unw_set_base %>%

      mutate(.idrow = do.call(paste, c(across(all_of(key_cols)), sep = "_"))) %>%

      semi_join(wt_set %>%

                  mutate(.idrow = do.call(paste, c(across(all_of(key_cols)), sep = "_"))),

                by = ".idrow") %>%

      select(-.idrow)

  }

} else {

  unw_set <- unw_set_base

}

w <- wt_set$ipw_trunc

ESS <- (sum(w)^2) / sum(w^2)

cat("\n200 mg IPW (FA1+FA2) diagnostics:  N_weighted = ", nrow(wt_set),

    " | ESS ≈ ", round(ESS, 1), "\n", sep = "")

cat("\nUNWEIGHTED (consent==Yes, 200mg) counts:\n")

cat("  PB  : D/D ", sum(unw_set$PB  == 1 & unw_set$DD),  "/", sum(unw_set$DD),

    " | I-carrier ", sum(unw_set$PB  == 1 & unw_set$icarrier == 1), "/", sum(unw_set$icarrier == 1), "\n", sep = "")

cat("  EPB : D/D ", sum(unw_set$EPB == 1 & unw_set$DD),  "/", sum(unw_set$DD),

    " | I-carrier ", sum(unw_set$EPB == 1 & unw_set$icarrier == 1), "/", sum(unw_set$icarrier == 1), "\n", sep = "")

cat("\nWEIGHTED usable counts:\n")

cat("  PB  : D/D ", sum(wt_set$PB  == 1 & wt_set$DD),  "/", sum(wt_set$DD),

    " | I-carrier ", sum(wt_set$PB  == 1 & wt_set$icarrier == 1), "/", sum(wt_set$icarrier == 1), "\n", sep = "")

cat("  EPB : D/D ", sum(wt_set$EPB == 1 & wt_set$DD),  "/", sum(wt_set$DD),

    " | I-carrier ", sum(wt_set$EPB == 1 & wt_set$icarrier == 1), "/", sum(wt_set$icarrier == 1), "\n", sep = "")

stan_code_exact_unweighted <- "

data { int<lower=1> N; int<lower=0,upper=1> y[N]; int<lower=0,upper=1> Icarrier[N]; }

parameters { real a; real b; }

model {

  a ~ normal(0, 5);

  b ~ normal(0, 5);

  for (n in 1:N) {

    real eta = a + (b - a) * Icarrier[n];

    y[n] ~ bernoulli_logit(eta);

  }

}

generated quantities {

  real p_DD_200 = inv_logit(a);

  real p_IC_200 = inv_logit(b);

  real RD_200   = p_IC_200 - p_DD_200;

  real RR_200   = p_IC_200 / fmax(p_DD_200, 1e-12);

}

"

stan_code_exact_weighted <- "

data { int<lower=1> N; int<lower=0,upper=1> y[N]; int<lower=0,upper=1> Icarrier[N]; vector<lower=0>[N] w; }

parameters { real a; real b; }

model {

  a ~ normal(0, 5);

  b ~ normal(0, 5);

  for (n in 1:N) {

    real eta = a + (b - a) * Icarrier[n];

    target += w[n] * bernoulli_logit_lpmf(y[n] | eta);

  }

}

generated quantities {

  real p_DD_200 = inv_logit(a);

  real p_IC_200 = inv_logit(b);

  real RD_200   = p_IC_200 - p_DD_200;

  real RR_200   = p_IC_200 / fmax(p_DD_200, 1e-12);

}

"

fit_unw <- function(dat, outcome_col) {

  d <- dat |>

    transmute(y = .data[[outcome_col]],

              Icarrier = as.integer(icarrier == 1)) |>

    na.omit()

  if (nrow(d) < 10) stop("Too few rows for unweighted fit.")

  sd <- list(N = nrow(d), y = d$y, Icarrier = d$Icarrier)

  fit <- rstan::stan(model_code = stan_code_exact_unweighted, data = sd,

                     iter = 4000, warmup = 1000, chains = 4, seed = 2025,

                     control = list(adapt_delta = 0.99, max_treedepth = 14))

  dr <- rstan::extract(fit)

  list(

    fit = fit,

    Pr_RD_gt0 = mean(dr$RD_200 > 0),

    Pr_RR_gt1 = mean(dr$RR_200 > 1),

    summaries = list(

      p_DD_200 = c(mean=mean(dr$p_DD_200), quantile(dr$p_DD_200, c(.025,.975))),

      p_IC_200 = c(mean=mean(dr$p_IC_200), quantile(dr$p_IC_200, c(.025,.975))),

      RD_200   = c(mean=mean(dr$RD_200),   quantile(dr$RD_200,   c(.025,.975))),

      RR_200   = c(mean=mean(dr$RR_200),   quantile(dr$RR_200,   c(.025,.975)))

    )

  )

}

fit_wt <- function(dat, outcome_col) {

  d <- dat |>

    transmute(y = .data[[outcome_col]],

              Icarrier = as.integer(icarrier == 1),

              w = as.numeric(ipw_trunc)) |>

    na.omit()

  if (nrow(d) < 10) stop("Too few rows for weighted fit.")

  ESS <- (sum(d$w)^2) / sum(d$w^2)

  cat("ESS(weighted) ≈ ", round(ESS,1), " of N=", nrow(d), "\n", sep="")

  sd <- list(N = nrow(d), y = d$y, Icarrier = d$Icarrier, w = d$w)

  fit <- rstan::stan(model_code = stan_code_exact_weighted, data = sd,

                     iter = 4000, warmup = 1000, chains = 4, seed = 2025,

                     control = list(adapt_delta = 0.99, max_treedepth = 14))

  dr <- rstan::extract(fit)

  list(

    fit = fit,

    Pr_RD_gt0 = mean(dr$RD_200 > 0),

    Pr_RR_gt1 = mean(dr$RR_200 > 1),

    summaries = list(

      p_DD_200 = c(mean=mean(dr$p_DD_200), quantile(dr$p_DD_200, c(.025,.975))),

      p_IC_200 = c(mean=mean(dr$p_IC_200), quantile(dr$p_IC_200, c(.025,.975))),

      RD_200   = c(mean=mean(dr$RD_200),   quantile(dr$RD_200,   c(.025,.975))),

      RR_200   = c(mean=mean(dr$RR_200),   quantile(dr$RR_200,   c(.025,.975)))

    )

  )

}

fmt <- function(x) sprintf("%.4f", x)

print_block <- function(res_unw, res_wt, label) {

  cat("\n==================== ", label, " (200 mg) ====================\n", sep="")

  cat("\nPosterior means [95% CrI]\n")

  cat("p_DD_200 : ", fmt(res_unw$summaries$p_DD_200[1]), " [",

      fmt(res_unw$summaries$p_DD_200[2]), ", ", fmt(res_unw$summaries$p_DD_200[3]), "]   |   ",

      fmt(res_wt$summaries$p_DD_200[1]), " [",

      fmt(res_wt$summaries$p_DD_200[2]), ", ", fmt(res_wt$summaries$p_DD_200[3]), "]\n", sep="")

  cat("p_IC_200 : ", fmt(res_unw$summaries$p_IC_200[1]), " [",

      fmt(res_unw$summaries$p_IC_200[2]), ", ", fmt(res_unw$summaries$p_IC_200[3]), "]   |   ",

      fmt(res_wt$summaries$p_IC_200[1]), " [",

      fmt(res_wt$summaries$p_IC_200[2]), ", ", fmt(res_wt$summaries$p_IC_200[3]), "]\n", sep="")

  cat("RD_200   : ", fmt(res_unw$summaries$RD_200[1]), " [",

      fmt(res_unw$summaries$RD_200[2]), ", ", fmt(res_unw$summaries$RD_200[3]), "]   |   ",

      fmt(res_wt$summaries$RD_200[1]), " [",

      fmt(res_wt$summaries$RD_200[2]), ", ", fmt(res_wt$summaries$RD_200[3]), "]\n", sep="")

  cat("RR_200   : ", fmt(res_unw$summaries$RR_200[1]), " [",

      fmt(res_unw$summaries$RR_200[2]), ", ", fmt(res_unw$summaries$RR_200[3]), "]\n", sep="")

  cat("\nPosterior probabilities\n")

  cat("Pr(RD_200 > 0):  ", fmt(res_unw$Pr_RD_gt0), "  |  ", fmt(res_wt$Pr_RD_gt0), "\n", sep="")

  cat("Pr(RR_200 > 1):  ", fmt(res_unw$Pr_RR_gt1), "  |  ", fmt(res_wt$Pr_RR_gt1), "\n", sep="")

}

res_unw_PB  <- fit_unw(unw_set, "PB")

res_wt_PB   <- fit_wt(wt_set,  "PB")

print_block(res_unw_PB, res_wt_PB, "PB")

res_unw_EPB <- fit_unw(unw_set, "EPB")

res_wt_EPB  <- fit_wt(wt_set,  "EPB")

print_block(res_unw_EPB, res_wt_EPB, "EPB")
library(readxl)

library(dplyr)

library(tidyr)

path_xlsx  <- "C:/Users/Kevin/OneDrive - The University of Texas at Austin/1053 consent used for IPW.xlsx"

sheet_name <- "Sheet2"

df <- read_xlsx(path_xlsx, sheet = sheet_name) |> as.data.frame()

names(df) <- trimws(names(df))

ga_candidates <- grep("^gestAgeBirth(\\.\\.\\d+)?$", names(df), value = TRUE)

if (length(ga_candidates) == 0) stop("No gestAgeBirth column found.")

col_ga <- if ("gestAgeBirth" %in% ga_candidates) "gestAgeBirth" else {

  get_idx <- function(x) as.integer(sub(".*\\.\\.(\\d+)$", "\\1", x))

  ga_candidates[order(get_idx(ga_candidates))][1]

}

message("Using GA column: ", col_ga)

col_consent <- if ("Optional genetic research consent obtained" %in% names(df))

  "Optional genetic research consent obtained" else

  grep("^Optional genetic research consent obtained", names(df), value=TRUE)[1]

col_center  <- if ("Center" %in% names(df)) "Center" else if ("Center " %in% names(df)) "Center " else NA

col_race    <- "maternal_race2"

col_geno    <- "Genotype"

col_treat   <- "Treatment"

col_FA1     <- "ARA/DGLA BL"

col_FA2     <- "a_2_6n3__corr_BL"

need <- c(col_ga, col_consent, col_center, col_race, col_geno, col_treat, col_FA1, col_FA2)

miss <- need[!(need %in% names(df))]

if (length(miss)) stop("Missing required columns: ", paste(miss, collapse=", "))

clean_geno <- function(s){

  s <- toupper(trimws(as.character(s))); s <- gsub(" ", "", s)

  s <- gsub("^II$","I/I",s); s <- gsub("^ID$","I/D",s); s <- gsub("^DD$","D/D",s); s

}

is_200 <- function(x){

  if (is.numeric(x)) x==200 else {

    xl <- tolower(as.character(x)); startsWith(xl,"200") | xl=="200mg" | xl=="200"

  }

}

df <- df |>

  mutate(

    GA_wks = as.numeric(.data[[col_ga]]),

    consent_bin = case_when(

      tolower(trimws(.data[[col_consent]])) == "yes" ~ 1L,

      tolower(trimws(.data[[col_consent]])) == "no"  ~ 0L,

      TRUE ~ NA_integer_

    ),

    geno_clean = clean_geno(.data[[col_geno]]),

    genotyped_bin = as.integer(!is.na(geno_clean)),

    S_included = as.integer(consent_bin==1 & genotyped_bin==1),

    dose_grp = ifelse(is_200(.data[[col_treat]]), "200mg", "1000mg"),

    center = factor(.data[[col_center]]),

    maternal_race2 = factor(.data[[col_race]]),

    FA1 = suppressWarnings(as.numeric(.data[[col_FA1]])),

    FA2 = suppressWarnings(as.numeric(.data[[col_FA2]]))

  )

target_all  <- df %>% filter(!is.na(GA_wks))

analyzed_all <- target_all %>% filter(S_included == 1)

cat("Sizes -> Target (with outcomes):", nrow(target_all),

    " | Analyzed (S=1: consented+genotyped):", nrow(analyzed_all), "\n")

build_ipw_one_arm <- function(df_arm){

  d <- df_arm %>% filter(!is.na(GA_wks))

  den_fit <- glm(S_included ~ center + maternal_race2 + FA1 + FA2,

                 data = d, family = binomial())

  num_fit <- glm(S_included ~ center + maternal_race2,

                 data = d, family = binomial())

  d <- d %>% mutate(

    p_den = plogis(predict(den_fit, newdata = d)),

    p_num = plogis(predict(num_fit, newdata = d)),

    ipw   = p_num / p_den

  )

  lo <- quantile(d$ipw[d$S_included==1], 0.01, na.rm=TRUE)

  hi <- quantile(d$ipw[d$S_included==1], 0.99, na.rm=TRUE)

  d <- d %>% mutate(ipw_trunc = ifelse(S_included==1, pmin(pmax(ipw, lo), hi), NA_real_))

  ww  <- d$ipw_trunc[!is.na(d$ipw_trunc)]

  ESS <- (sum(ww)^2) / sum(ww^2)

  list(df_arm = d, ESS = ESS)

}

arm_levels <- c("200mg","1000mg")

ipw_list <- lapply(arm_levels, function(a) {

  res <- build_ipw_one_arm(df %>% filter(dose_grp==a))

  cat(sprintf("Arm %s: N_analyzed=%d | ESS ≈ %.1f\n",

              a, sum(res$df_arm$S_included==1, na.rm=TRUE), res$ESS))

  res$df_arm$ipw_trunc

})

df$ipw_trunc <- NA_real_

for (i in seq_along(arm_levels)) {

  idx <- which(df$dose_grp==arm_levels[i] & !is.na(df$GA_wks))

  df$ipw_trunc[idx] <- ipw_list[[i]]

}

w_all <- df$ipw_trunc[which(df$S_included==1 & !is.na(df$ipw_trunc))]

ESS_all <- (sum(w_all)^2) / sum(w_all^2)

cat(sprintf("Combined (both arms): N_analyzed=%d | ESS ≈ %.1f\n",

            sum(df$S_included==1, na.rm=TRUE), ESS_all))

smd_cont <- function(x_tgt, x_anl, w_anl=NULL) {

  m1 <- mean(x_tgt, na.rm=TRUE); v1 <- var(x_tgt, na.rm=TRUE)

  if (is.null(w_anl)) {

    m2 <- mean(x_anl, na.rm=TRUE); v2 <- var(x_anl, na.rm=TRUE)

  } else {

    w <- w_anl[!is.na(x_anl)]; x2 <- x_anl[!is.na(x_anl)]

    m2 <- sum(w * x2) / sum(w)

    v2 <- sum(w * (x2 - m2)^2) / sum(w)  # method of moments

  }

  sd_pool <- sqrt((v1 + v2)/2)

  (m2 - m1) / sd_pool

}

composition_table <- function(target_df, analyzed_df, analyzed_w_df=NULL, label="OVERALL") {

  td <- target_df %>% mutate(cell = interaction(center, maternal_race2, drop=TRUE))

  ad <- analyzed_df %>% mutate(cell = interaction(center, maternal_race2, drop=TRUE))

  tab_t <- td %>% count(cell, name="N_target")

  tab_a <- ad %>% count(cell, name="N_anl")

  comp  <- full_join(tab_t, tab_a, by="cell") %>%

    mutate(across(c(N_target, N_anl), ~replace_na(., 0L))) %>%

    mutate(prop_target = N_target / sum(N_target),

           prop_anl    = N_anl / sum(N_anl),

           diff_prop   = prop_anl - prop_target)

  if (!is.null(analyzed_w_df)) {

    aw <- analyzed_w_df %>%

      mutate(cell = interaction(center, maternal_race2, drop=TRUE)) %>%

      group_by(cell) %>% summarise(W_anl = sum(ipw_trunc), .groups="drop") %>%

      mutate(prop_anl_w = W_anl / sum(W_anl))

    comp <- comp %>% left_join(aw, by="cell") %>%

      mutate(prop_anl_w = replace_na(prop_anl_w, 0),

             diff_prop_w = prop_anl_w - prop_target)

  }

stan_code_unw <- "

data { int<lower=1> N; int<lower=0,upper=1> y[N]; int<lower=0,upper=1> Icarrier[N]; }

parameters { real a; real b; }

model {

  a ~ normal(0,5);

  b ~ normal(0,5);

  for (n in 1:N) {

    real eta = a + (b - a) * Icarrier[n];

    y[n] ~ bernoulli_logit(eta);

  }

}

generated quantities {

  real p_DD  = inv_logit(a);

  real p_IC  = inv_logit(b);

  real RD    = p_IC - p_DD;

  real RR    = p_IC / fmax(p_DD, 1e-12);

  int RD_pos = RD > 0;

  int RR_gt1 = RR > 1;

}

"

stan_code_wt <- "

data { int<lower=1> N; int<lower=0,upper=1> y[N]; int<lower=0,upper=1> Icarrier[N]; vector<lower=0>[N] w; }

parameters { real a; real b; }

model {

  a ~ normal(0,5);

  b ~ normal(0,5);

  for (n in 1:N) {

    real eta = a + (b - a) * Icarrier[n];

    target += w[n] * bernoulli_logit_lpmf(y[n] | eta);

  }

}

generated quantities {

  real p_DD  = inv_logit(a);

  real p_IC  = inv_logit(b);

  real RD    = p_IC - p_DD;

  real RR    = p_IC / fmax(p_DD, 1e-12);

  int RD_pos = RD > 0;

  int RR_gt1 = RR > 1;

}

"

run_stan <- function(dat, outcome) {

  d <- dat %>% transmute(y = .data[[outcome]],

                         Icarrier = as.integer(geno_clean %in% c("I/I","I/D"))) %>%

               drop_na()

  list(N = nrow(d), y = d$y, Icarrier = d$Icarrier)

}

for (dose in c("200mg","1000mg")) {

  cat("\n================ Dose:", dose, "================\n")

  d_target <- df %>% filter(dose_grp==dose, !is.na(GA_wks))

  d_anl_unw <- d_target %>% filter(S_included==1)

  d_anl_wt  <- d_target %>% filter(S_included==1, !is.na(ipw_trunc))

  sd_unw <- run_stan(d_anl_unw, "GA_wks")     # we use GA later to derive PB/EPB

  sd_unw$y <- as.integer(d_anl_unw$GA_wks < 37)

  fit_unw <- rstan::stan(model_code = stan_code_unw, data = sd_unw,

                         iter=4000, warmup=1000, chains=4, seed=2025)

  sd_wt  <- sd_unw

  sd_wt$w <- d_anl_wt$ipw_trunc

  sd_wt$y <- as.integer(d_anl_wt$GA_wks < 37)

  sd_wt$Icarrier <- sd_unw$Icarrier

  fit_wt <- rstan::stan(model_code = stan_code_wt, data = sd_wt,

                        iter=4000, warmup=1000, chains=4, seed=2025)

  print(fit_unw, pars=c("p_DD","p_IC","RD","RR"), probs=c(.025,.975))

  print(fit_wt,  pars=c("p_DD","p_IC","RD","RR"), probs=c(.025,.975))

  cat("Pr(RD>0) unweighted:",

      mean(rstan::extract(fit_unw)$RD_pos),

      " | weighted:", mean(rstan::extract(fit_wt)$RD_pos), "\n")

  cat("Pr(RR>1)  unweighted:",

      mean(rstan::extract(fit_unw)$RR_gt1),

      " | weighted:", mean(rstan::extract(fit_wt)$RR_gt1), "\n")

}

  TV_unw <- 0.5 * sum(abs(comp$prop_anl - comp$prop_target))

  TV_w   <- if ("prop_anl_w" %in% names(comp)) 0.5 * sum(abs(comp$prop_anl_w - comp$prop_target)) else NA

  cat("\n--- Center×Race composition:", label, "---\n")

  cat("Total Variation (unweighted) =", sprintf("%.4f", TV_unw))

  if (!is.na(TV_w)) cat(" | (weighted) =", sprintf("%.4f", TV_w))

  cat("\n")

  comp_print <- comp %>%

    arrange(desc(abs(diff_prop))) %>%

    select(cell, prop_target, prop_anl, diff_prop,

           if ("prop_anl_w" %in% names(comp)) c("prop_anl_w","diff_prop_w") else NULL)

  print(head(comp_print, 10))

  SMD_FA1_unw <- smd_cont(target_df$FA1, analyzed_df$FA1, NULL)

  SMD_FA2_unw <- smd_cont(target_df$FA2, analyzed_df$FA2, NULL)

  if (!is.null(analyzed_w_df)) {

    SMD_FA1_w <- smd_cont(target_df$FA1, analyzed_w_df$FA1, analyzed_w_df$ipw_trunc)

    SMD_FA2_w <- smd_cont(target_df$FA2, analyzed_w_df$FA2, analyzed_w_df$ipw_trunc)

    cat(sprintf("FA SMDs:  unweighted  FA1=%.3f FA2=%.3f  |  weighted  FA1=%.3f FA2=%.3f\n",

                SMD_FA1_unw, SMD_FA2_unw, SMD_FA1_w, SMD_FA2_w))

  } else {

    cat(sprintf("FA SMDs (unweighted): FA1=%.3f FA2=%.3f\n", SMD_FA1_unw, SMD_FA2_unw))

  }

  invisible(comp)

}

target_overall  <- df %>% filter(!is.na(GA_wks)) %>% select(center, maternal_race2, FA1, FA2)

analyzed_overall_unw <- df %>% filter(!is.na(GA_wks), S_included==1) %>% select(center, maternal_race2, FA1, FA2)

analyzed_overall_w   <- df %>% filter(!is.na(GA_wks), S_included==1, !is.na(ipw_trunc)) %>%

  select(center, maternal_race2, FA1, FA2, ipw_trunc)

composition_table(target_overall, analyzed_overall_unw, analyzed_overall_w, label="OVERALL (both doses)")

for (d in c("200mg","1000mg")) {

  t_d  <- df %>% filter(!is.na(GA_wks), dose_grp==d) %>% select(center, maternal_race2, FA1, FA2)

  a_u  <- df %>% filter(!is.na(GA_wks), dose_grp==d, S_included==1) %>% select(center, maternal_race2, FA1, FA2)

  a_w  <- df %>% filter(!is.na(GA_wks), dose_grp==d, S_included==1, !is.na(ipw_trunc)) %>%

                 select(center, maternal_race2, FA1, FA2, ipw_trunc)

  composition_table(t_d, a_u, a_w, label=paste("Dose =", d))

}
```

```
