# Supplementary material for: Genotype-informed docosahexaenoic acid dosing based on the FADS Indel to reduce preterm and prolonged gestation: A secondary analysis of the ADORE randomized trial
Source: PLoS Med. 2026 Oct 8;23(10):e1005274. doi: 10.1371/journal.pmed.1005274 (PMC13660990; doi:10.1371/journal.pmed.1005274)
Supplement: Supplementary file 8 [file pmed.1005274.s021.docx]

*Response to Reviewers*

*Li, Kothapalli, Carlson, Gajewski, DeFranco, Brenna, PLoS Med 2026*

*Reviewer 1*

*Major Comment 1:*

*The binomial models should be described more clearly in Methods. While the outcome variables are clear and it is clear that the DHA dose exposure and FADS Indel genotype are included as predictor variables, it is unclear if any other variables such as center, age, sex etc were included. Further the model building process seems not to be described. E.g. p.8 refers to a final model - but no model selection procedure is described (incidentally this should probably be a plural given there was more than one model; e.g. one model for PB and one for EPB).*

Response:

We agree and have expanded the statistical analysis section to detail the binomial models. The analysis comprises Bayesian non-hierarchical logistic-binomial models, one per outcome (PB and EPB) within each genotype/dose contrast. These models mirror the treatment-specific Bayesian binomial model used for the primary efficacy analysis of the parent ADORE trial (Carlson et al. 2021) and its statistical analysis plan (SAP §3.4), in which the event rate for each arm is modeled independently on the log-odds (logit) scale with no pooling across arms. Each genotype- or dose-specific arm has its own intercept on the log-odds scale; no additional covariates (center, age, sex) enter the primary model, and potential confounding of the non-randomized genotype contrasts by center, maternal race, baseline ARA/DGLA, and baseline %DHA is instead addressed through the inverse probability weighting sensitivity analysis. The independent (non-pooled) binomial design, and the rationale that borrowing across groups can inflate false positives when group effects differ, follow Berry, Carlin, Lee & Müller, *Bayesian Adaptive Methods for Clinical Trials* (§6.2.2, Binomial response design; and the borrowing/false-positive caution in §4.7.1). We have corrected the in-text citation and added it to the reference list.

Reviewer 1

Major Comment 2:

*While the authors provide extensive sensitivity analyses to appraise the robustness of the results, it is a bit surprising to find no indication of any model diagnostics having been done - e.g. given that the models were fitted by MCMC, I would expect a statement on whether trace plots, potential scale reduction factors, effective sample sizes of parameters etc were checked, whether any model fit statistics were checked, whether predictive posterior checks were done etc and maybe have some of these included as supplementary materials.*

Response:

All results in the manuscript are produced by a single Bayesian binomial model, fitted independently within each genotype and dose group. For every fit, we report the convergence and sampling diagnostics recommended for Hamiltonian Monte Carlo, including the potential scale reduction factor (R-hat), bulk- and tail-effective sample sizes (ESS), divergent transitions, maximum-treedepth saturations, and the energy Bayesian fraction of missing information (E-BFMI). Model adequacy was assessed with posterior predictive checks, and model-fit statistics (WAIC and leave-one-out cross-validation) were computed for the individual-level fits. All diagnostics were generated from the fitted model objects; no change to the models or the reported results was required.

All models were fitted in Stan (via the rstan interface, R 4.2.3) using four Hamiltonian Monte Carlo chains of 4,000 iterations each (1,000 warm-up per chain; 12,000 retained draws in total), with adapt_delta = 0.99 and max_treedepth = 14. Convergence was deemed acceptable when R-hat ≤ 1.01 and bulk- and tail-ESS ≥ 400 for all parameters, with no divergent transitions, no maximum-treedepth saturations, and E-BFMI > 0.2. All models converged (maximum R-hat = 1.003; bulk- and tail-ESS > 3,300 for all parameters; zero divergent transitions and no maximum-treedepth saturations; E-BFMI ≥ 0.81), and posterior predictive checks showed no evidence of misfit; convergence was likewise confirmed across all sensitivity-grid refits (Table 1 in S1 Appendix; Figures S1 and 2).

**Table 1 in S1 Appendix. MCMC convergence and sampling diagnostics for the Bayesian binomial models.**

Each row corresponds to one fitted Bayesian binomial model (28 models in total). For each model, the reported R-hat, bulk effective sample size (bulk-ESS), and tail effective sample size (tail-ESS) are the worst-case values across the model's parameters. All models showed satisfactory convergence and sampling performance, with maximum R-hat = 1.003, minimum bulk-ESS = 3,591, minimum tail-ESS = 3,393, no divergent transitions, no maximum-treedepth saturations, and minimum E-BFMI = 0.82. Models were fitted with four Hamiltonian Monte Carlo chains (4,000 iterations; 1,000 warm-up). R-hat, potential scale reduction factor (Gelman–Rubin); bulk-ESS/tail-ESS, bulk and tail effective sample size; E-BFMI, energy Bayesian fraction of missing information; PB, preterm birth; EPB, early preterm birth; IPW, inverse-probability weighting.

| **Model** | **Outcome** | **max R-hat** | **min Bulk-ESS** | **min Tail-ESS** | **Divergences** | **E-BFMI** |
| --- | --- | --- | --- | --- | --- | --- |
| Dose contrast (200 vs 1000 mg) — D/D, all | PB (<37 wk) | 1.0005 | 6,318 | 5,550 | 0 | 0.96 |
| Dose contrast (200 vs 1000 mg) — I/D, all | PB (<37 wk) | 1.0005 | 6,515 | 5,317 | 0 | 0.95 |
| Dose contrast (200 vs 1000 mg) — I/I, all | PB (<37 wk) | 1.0006 | 5,936 | 5,490 | 0 | 1.01 |
| Dose contrast (200 vs 1000 mg) — D/D, all | EPB (<34 wk) | 1.0011 | 4,979 | 3,691 | 0 | 0.85 |
| Dose contrast (200 vs 1000 mg) — I/D, all | EPB (<34 wk) | 1.0017 | 5,374 | 4,173 | 0 | 0.95 |
| Dose contrast (200 vs 1000 mg) — I/I, all | EPB (<34 wk) | 1.0013 | 5,337 | 3,703 | 0 | 0.87 |
| Dose contrast (200 vs 1000 mg) — D/D, Black | PB (<37 wk) | 1.0010 | 4,686 | 3,514 | 0 | 0.82 |
| Dose contrast (200 vs 1000 mg) — I/D, Black | PB (<37 wk) | 1.0007 | 5,695 | 4,723 | 0 | 0.95 |
| Dose contrast (200 vs 1000 mg) — I/I, Black | PB (<37 wk) | 1.0005 | 5,918 | 4,680 | 0 | 0.96 |
| Dose contrast (200 vs 1000 mg) — D/D, Black | EPB (<34 wk) | 1.0007 | 3,591 | 3,481 | 0 | 0.92 |
| Dose contrast (200 vs 1000 mg) — I/D, Black | EPB (<34 wk) | 1.0030 | 5,062 | 3,789 | 0 | 0.88 |
| Dose contrast (200 vs 1000 mg) — I/I, Black | EPB (<34 wk) | 1.0002 | 3,898 | 3,604 | 0 | 0.87 |
| Genotype contrast (I-carrier vs D/D), 200 mg — unweighted | PB (<37 wk) | 1.0007 | 6,140 | 5,051 | 0 | 0.94 |
| Genotype contrast (I-carrier vs D/D), 200 mg — IPW-weighted | PB (<37 wk) | 1.0010 | 6,351 | 5,216 | 0 | 0.94 |
| Genotype contrast (I-carrier vs D/D), 200 mg — unweighted | EPB (<34 wk) | 1.0002 | 5,154 | 4,209 | 0 | 0.89 |
| Genotype contrast (I-carrier vs D/D), 200 mg — IPW-weighted | EPB (<34 wk) | 1.0007 | 4,552 | 3,393 | 0 | 0.90 |
| Late-term birth (>40 wk) — dose contrast, D/D | >40 wk | 1.0007 | 6,550 | 5,959 | 0 | 0.98 |
| Late-term birth (>40 wk) — dose contrast, I-carrier | >40 wk | 1.0005 | 5,943 | 4,896 | 0 | 0.95 |
| Late-term birth (>40 wk) — genotype contrast, 200 mg | >40 wk | 1.0009 | 5,923 | 4,850 | 0 | 1.00 |
| Late-term birth (>40 wk) — genotype contrast, 1000 mg | >40 wk | 1.0008 | 6,288 | 5,779 | 0 | 1.00 |
| Dose contrast (200 vs 1000 mg) — I-allele carriers (pooled) | PB (<37 wk) | 1.0008 | 5,884 | 5,396 | 0 | 0.99 |
| Dose contrast (200 vs 1000 mg) — I-allele carriers (pooled) | EPB (<34 wk) | 1.0007 | 5,018 | 4,321 | 0 | 0.92 |
| Genotype contrast (I-carrier vs D/D), 1000 mg | PB (<37 wk) | 1.0010 | 6,436 | 5,248 | 0 | 1.01 |
| Genotype contrast (I-carrier vs D/D), 1000 mg | EPB (<34 wk) | 1.0018 | 6,332 | 5,260 | 0 | 0.94 |
| Mismatched dose: D/D 1000 mg vs I-carrier 200 mg | PB (<37 wk) | 1.0006 | 6,599 | 5,814 | 0 | 0.97 |
| Mismatched dose: D/D 1000 mg vs I-carrier 200 mg | EPB (<34 wk) | 1.0006 | 5,827 | 4,881 | 0 | 0.96 |
| Matched dose: D/D 200 mg vs I-carrier 1000 mg | PB (<37 wk) | 1.0005 | 6,752 | 5,925 | 0 | 1.02 |
| Matched dose: D/D 200 mg vs I-carrier 1000 mg | EPB (<34 wk) | 1.0013 | 4,560 | 3,558 | 0 | 0.84 |
| **Summary (all 28 models):** |  | **1.0030** | **3,591** | **3,393** | **0** | **0.82** |

**Supplemental Figure 1. MCMC trace plots (four chains) for representative binomial models.**

Trace plots are shown for: (a) early preterm birth among Black D/D participants in the dose-contrast model, representing a sparse stratum with zero events in both treatment arms; (b) preterm birth among all I/D participants in the dose-contrast model, representing a better-powered comparison; and genotype-contrast models comparing I-allele carriers with D/D participants in the 200 mg arm for (c) preterm birth and (d) early preterm birth. Across all panels, the four chains overlap closely with no visible trend or drift, supporting convergence. Mixing was also acceptable in the sparse early-preterm-birth model, indicating stable sampling even under low-event conditions. Parameters are shown on the log-odds scale.

**Supplemental Figure 2. Posterior predictive checks for the genotype contrasts.**

Posterior predictive checks are shown for genotype-specific event rates among D/D participants and I-allele carriers in the 200 mg/d and 1000 mg/d DHA arms: (a) preterm birth (<37 weeks), 200 mg/d; (b) preterm birth (<37 weeks), 1000 mg/d; (c) early preterm birth (<34 weeks), 200 mg/d; and (d) early preterm birth (<34 weeks), 1000 mg/d. Histograms show the distribution of event rates across datasets simulated from the fitted Bayesian models, and vertical dark blue lines indicate the observed event rates. Across outcomes, treatment arms, and genotype groups, the observed event rates fall within the posterior predictive distributions, with no evidence of misfit. The discrete spikes in the early-preterm-birth panels reflect the small number of possible event counts at those low rates.

Reviewer 1

Specific comments 1:

*Data availability statements in the PLOS submission form and what is written on p.17 contradict each other. I note that the data were included in the reviewer submission pack.*

Response:

The data availability described in the manuscript controls.

Reviewer 1

Specific comments 2:

*The CONSORT 2025 checklist needs minor tweaks as some page numbers seem to be off by a count of 1. Further, not all points are fully compliant: 1) the item on sample size is not compliant as the manuscript simply refers to a non-publicly available Statistical Analysis Plan (I would suggest including full details in the manuscript or at least uploading the SAP to a permanent public repository and linking to it from Methods), 2) there is no description of blinding procedures, 3) missing data handling (which is straightforward in a Bayesian model as long as all variables that include missing data have distributions specified but that is not clear; note I do not refer to the sensitivity analysis for participants not wishing to be genotyped - that is very good) and 4) item 26 presentation of binary results (see also comment S4 below).*

Response:

1. We will upload the SAP for the original ADORE trial when submitting the review responses.
2. We have added description of the blinding procedure to the method section.
3. We verified the primary-model variables have zero missing, we have added a section at the end of the first statistical-analysis paragraph to clarify.
4. We have responded and edited the manuscript regarding comment S4.

Reviewer 1

Specific comments 3:

*Methods should also be more specific on reporting - e.g. mention that CONSORT2025 are followed (as they are) and explain what descriptive and analysis summaries are being reported and what other analyses were done for more simple comparisons. E.g. this would make it clear that "CI" refers generally to Bayesian confidence intervals (which some readers may know as credible intervals). Also on p.12, there seems to be a frequentist analysis that uses the same CI abbreviation for a frequentist confidence interval (for the difference in cervical length), together with a frequentist p-value for what is probably a t-test or Wilcoxon rank-sum test but this is not clear. Or was this also a Bayesian analysis and the 'p' should be a 'PP'?*

Response:

We now state in the Methods that reporting follows the CONSORT 2025 statement.

We have added a sentence specifying what is reported: descriptive results as counts and percentages (n/N, %); Bayesian model estimates as posterior mean event rates with 95% credible intervals; and between-group comparisons as risk ratios with 95% credible intervals and posterior probabilities (PP).

We have defined and relabeled Bayesian credible intervals as "CrI" throughout to remove ambiguity.

Cervical length comparisons are done with frequentist methods. Cervical length by DHA dose was compared with a Welch t-test, and the “p” is a frequentist p-value. We have clarified in the Methods that this single secondary comparison was frequentist, named the test explicitly, and labeled its interval a frequentist 95% confidence interval (distinct from the Bayesian credible intervals used elsewhere).

Reviewer 1

Specific comments 4:

*I would suggest consistently reporting results in the same way on 9-12. Currently sometimes only percentages with CIs are given and sometimes n/N are given and at other times only ratios are quoted. I would suggest consistently reporting n/N, %, 95% Bayesian CI for numbers and percentages of outcomes, and ratios, 95% Bayesian CIs, Bayesian PPs for comparisons. E.g. on p.9, as an example, report a CI for the 36% figure (which for consistency could be reported as a ratio of 0.64) and CIs for the 1.7 and 5.4-fold figures.*

Response:

We have standardized reporting throughout the Results so that every outcome is given as n/N, %, and 95% Bayesian credible interval, and every comparison as a ratio with 95% credible interval and posterior probability.

Reviewer 1

Specific comments 5:

*Avoid using the PP acronym for both posterior probability and post-partum as this is confusing. To be fair the latter only seems to be used on Figure 5 (though also in the data file). Probably the easiest is just to spell out the x-axis titles on Figure 5 (there is certainly enough space for 'baseline' and 'post-partum').*

Response:

BL and PP in figure 5 are now labeled as baseline and post-partum.

Reviewer 1

Specific comments 6:

*p.9, first sentence on the last paragraph has something missing. Perhaps it should be "carriers were at 1.7-fold and 5.4-fold greater risk than D/D"?*

Response:

The sentence is now fixed.

Reviewer 1

Specific comments 7:

*Add vertical lines to Figures 2, 3, 4, S5 and S6 to indicate the EPB (34 weeks) and PB (37 weeks) cut-offs as this will make it easier to read the Kaplan-Meier graphs. I also do not think  that Figures S5a and S6a are the best visual summaries for comparing prolonged gestation given that the curves shown are cumulative. The numbers given below each graph help with this, but the graphs themselves are not clear for comparing prolonged deliveries.*

Response:

We have added vertical lines at 34, 37 and 40wks to all the figures mentioned. We also generated additional posterior probability distribution plots (Supplemental Figure 10) like Supplemental Figure 4 for prolonged deliveries to better illustrate the comparison between genotype and doses.

**Supplemental Figure 10. Posterior distributions of prolonged birth (delivery at ≥40 weeks) by FADS Indel genotype and DHA dose.**

Overlaid histograms show the posterior prolonged-birth rate for the two groups in each panel. (A) D/D vs I-allele carriers, 1000 mg/d (49/191, 25.7% vs 33/205, 16.1%; PP = 0.99); (B) D/D vs I-allele carriers, 200 mg/d (23/151, 15.2% vs 38/214, 17.8%; PP = 0.26); (C) D/D, 1000 vs 200 mg/d (49/191, 25.7% vs 23/151, 15.2%; PP = 0.99); (D) I-allele carriers, 1000 vs 200 mg/d (33/205, 16.1% vs 38/214, 17.8%; PP = 0.32). PP is the posterior probability that the first-named group has the higher prolonged-birth rate.

Reviewer 1

Specific comments 8:

*Make some statements less definite as it implies a similar fallacy to interpreting results as taking a binary cut-off for p<0.05 in frequentist analyses. E.g. (all are just suggestions) p.10 "no effect was observed" --> "no meaningful effect was observed", p.11 "PB and EPB were indistinguishable" --> "PB and EPB were statistically indistinguishable" etc. If a hard interpretation of PPs is chosen, then the authors should make it clear what threshold of PP they use to distinguish between effects and non-effects - but personally I do not think that would be within the spirit of a nuanced Bayesian analysis.*

Response:

We have softened the interpretive language throughout to avoid implying a binary effect/no-effect cutoff.

Reviewer 1

Specific comments 9:

*The sentence "In other words, DHA dose did not affect the rate of ARA synthesis in the group overall" on p.11 is not clear unless the sentence before this is broken apart by DHA dose. Currently I do not see how a statement about D/D vs I/I genotype can lead to a conclusion about DHA dose.*

Response:

We added comparisons of D/D vs. I/I genotype’s ARA/DGLA ratio stratified by DHA dose prior to the sentence.

Reviewer 1

Specific comments 10:

*Having between a third and half of eligibility exclusions listed as 'other reasons' is not great on the CONSORT diagram. Can this be broken down a bit more?*

Response:

Yes, and in fact it was in Carlson, eClinMed 2021, but unintentionally omitted. We added them back.

Reviewer 1

Specific comments 11:

*It would be good to have early on a single table listing simply PB and EPB events overall, by arm and by genotype.*

Response:

We generated Table 2 per request.

| **Table 2. Preterm birth (PB) and early preterm birth (EPB) events overall, by treatment arm, and by FADS Indel genotype** | | | |
| --- | --- | --- | --- |
|  | **N** | **PB (<37 wk), n (%)** | **EPB (<34 wk), n (%)** |
| ***By treatment arm*** |  |  |  |
| 200 mg/d | 365 | 44 (12.1%) | 10 (2.7%) |
| 1000 mg/d | 396 | 30 (7.6%) | 7 (1.8%) |
| ***By FADS Indel genotype*** |  |  |  |
| D/D | 342 | 24 (7.0%) | 6 (1.8%) |
| I/D | 313 | 36 (11.5%) | 7 (2.2%) |
| I/I | 106 | 14 (13.2%) | 4 (3.8%) |
| **Total** | 761 | 74 (9.7%) | 17 (2.2%) |

Reviewer 1

Specific comments 12:

*Remove the word 'potential' from the first line of the last paragraph before the Conclusion on p.16.*

Response:

Word removed.

Reviewer 1

Specific comments 13:

*Add the word 'secondary' in from of 'analysis' in the first sentence of the Conclusion on p.16.*

Response:

Word added.

Reviewer 1

Specific comments 14:

*I am not sure the list of abbreviations is helpful if it is not complete - e.g. PP, CI etc are not featured.*

Response:

We have removed the list of abbreviations, but we ensured that all abbreviations used are defined at their first appearance in the manuscript.

Reviewer 1

Specific comments 15:

*Table 1 is not cross-referenced in the text.*

Response:

Table 1 is now cross referenced in the first paragraph of the results section.

Reviewer 1

Typo comments

Response:

All typos listed are fixed. PP=0.07 is correct and is not a transcription error for 0.97. D/D participants are low ARA synthesizers and therefore at intrinsically low risk of EPB, so very few EPB events occur in this genotype regardless of dose. In our cohort these few events happened to fall slightly more often in the high dose DHA arm than the low dose DHA arm (5/191 vs 1/151, thus yielding a low PP. Given the very small event count, we interpret this cautiously rather than as evidence of a meaningful increase in risk and have added this interpretation in the manuscript. Consistent with this, under a hierarchical model, the D/D EPB dose effect posterior probability moved from 0.07 to 0.62 (Table 3 in S1 Appendix), reflecting the observation to be the small event count rather than a genuine effect.

**Table 3 in S1 Appendix. Hierarchical model sensitivity analysis.**

Posterior probabilities that 1000 mg/d DHA reduced event risk compared with 200 mg/d are shown by genotype. The non-hierarchical model estimates each genotype separately, whereas the hierarchical model partially pools estimates across genotypes. PB, preterm birth; EPB, early preterm birth.

| Outcome | Genotype | Non-Hierarchical binomial model | Hierarchical partial-pooling model |
| --- | --- | --- | --- |
| PB (<37 wk) | D/D | 0.85 | 0.91 |
| PB (<37 wk) | I/D | 0.98 | 0.98 |
| PB (<37 wk) | I/I | 0.47 | 0.80 |
| EPB (<34 wk) | D/D | 0.07 | 0.62 |
| EPB (<34 wk) | I/D | 0.97 | 0.88 |
| EPB (<34 wk) | I/I | 0.85 | 0.82 |

Reviewer 2

Comment 1: *The post-hoc nature of the analysis is generally underplayed in the presentation and interpretation of the data. As this was not preplanned, the title should transparently refer to the investigation as a post-hoc over a 'secondary' analysis. Causal sounding language is used throughout (e.g. Abstract: "High-dose DHA (1000 mg/d) reduces preterm birth rate for I allele carriers but increased later term and post-term birth in DD. Matching dose to genotype yields the highest percentage of births 37-40 weeks" - should be "is associated with a reduced risk of preterm birth for I allele carriers".*

Response:

Thank you for raising this inconsistency.

Our study follows the **retrospective-prospective** model established more than a decade ago with studies in the New England Journal of Medicine and elsewhere that changed practice in oncology. Briefly, just as in our study, archived RCT samples were tested for a specific genetic polymorphism, finding that one genotype but not the other responded to treatment. We have now added a detailed explanation of this approach to the Discussion section including new references.

Briefly, while the analysis is post-hoc (done after the original study), the genotypes are randomized with respect to dose, it was driven by a specific single hypothesis and thus is not subject to multiple comparison shopping, and the genotype is not subject to reverse causation (i.e., preterm birth causes I allele).

Neverthless, we revised to use the word “association”.

Reviewer 2

Comment 2:

*Going along with the post-hoc issue, the analysis is not adequately powered to assess the full spectrum of possible genotypes with interactions and collapses heterozygotes and homozygotes for the indel as carriers. While this is common practice in the nutrigenetic literature, it is potentially obscures meaningful biological differences and serves mostly a statistical purpose to increase power. This should be more transparently handled in the analysis and enthusiasm tempered appropriately in the interpretation. For example, it is seen in Supplemental Figure 1 and 2 that there is clearer separation in the posterior distributions between the I/D 1000mg vs 200mg groups for PB and EPB outcomes than for the I/I genotype (PP=0.98&0.98, strong for I/D; PP=0.47 & 0.86, very weak to moderate for D/D). Whether there is a true biological disadvantage to carrying a single allele of the indel vs 2 copies is interesting to consider, but inseparable in this analysis from statistical
underpowering and reality of using a non-hierachical bayesian modeling approach that treats each group entirely independently when the I/I genotype is relatively small in number . The manuscript as written is presenting a bit more of a cleaner story than the data fully support.*

Response:

We agree that genotype-specific inferences, particularly for the smaller I/I subgroup and the sparse early preterm birth strata, are sensitive to model structure, and have tempered the genotype-specific language accordingly.

As answered in the response to reviewer 1 major comment 1, the primary analysis used independent binomial models, consistent with the approach taken in the primary efficacy analysis of the parent ADORE trial (Carlson et al., 2021). This non-pooled approach estimates each group's event rate on its own log-odds scale, with no borrowing across groups. For the main genotype contrast, I/D and I/I participants were grouped as I allele carriers because carrier status reflects the mechanism under study, even a single insertion allele raises FADS1 expression and circulating ARA relative to D/D, so both heterozygotes and homozygotes share the elevated ARA phenotype that distinguishes carriers from D/D. The grouping is therefore biologically motivated and provides greater precision for the carrier versus D/D contrast. A finer distinction within carriers (I/D vs I/I) is biologically plausible, but the present sample with the small I/I group is underpowered to resolve it.

As a sensitivity analysis and added to the manuscript, we fitted a hierarchical partial pooling model across the three genotypes. The overall interpretation was not materially changed: evidence for benefit of high dose DHA remained strongest at the carrier level and in the better powered I/D stratum, while estimates for the smaller I/I subgroup and the sparse early preterm birth group were more model sensitive. As expected, partial pooling mainly moderated estimates in the smallest or sparsest strata, including the I/I subgroup and the D/D early preterm birth estimate, while the better powered PB estimates and the I/D dose response pattern were comparatively stable. (Table 3 in S1 Appendix)

**Table 3 in S1 Appendix. Hierarchical model sensitivity analysis.** Posterior probabilities that 1000 mg/d DHA reduced event risk compared with 200 mg/d are shown by genotype. The non-hierarchical model estimates each genotype separately, whereas the hierarchical model partially pools estimates across genotypes. PB, preterm birth; EPB, early preterm birth.

| Outcome | Genotype | Non-Hierarchical binomial model | Hierarchical partial-pooling model |
| --- | --- | --- | --- |
| PB (<37 wk) | D/D | 0.85 | 0.91 |
| PB (<37 wk) | I/D | 0.98 | 0.98 |
| PB (<37 wk) | I/I | 0.47 | 0.8 |
| EPB (<34 wk) | D/D | 0.07 | 0.62 |
| EPB (<34 wk) | I/D | 0.97 | 0.88 |
| EPB (<34 wk) | I/I | 0.85 | 0.82 |

Reviewer 2

Comment 3:

*The findings for high dose DHA in carriers  are much more robust than for EPB and PB - the authors should explain this more clearly in their interpretation. They have correctly shown the fragility of the PB PP in Supp Table 10 with small changes to the gamma resulting in substantially reduced PP's but under interpret it.*

Response:

We have revised the manuscript in two places. First, we removed the prior statement that the results were uniformly robust and replaced it with a statement that the strength of evidence differs by outcome: in the outcome-tilted (worst-case) analysis, the early preterm birth association remained credible until a larger adverse tilt (Γ = 1.40), whereas the preterm-birth association fell below a posterior probability of 0.90 with only a small tilt (Γ = 1.1, equivalent to ≈3–4 of the 31 carrier preterm-birth cases). Second, we added a limitation explicitly stating that the early preterm birth effect carries the stronger evidentiary weight, while the preterm birth finding despite being directionally consistent, is more fragile and warrants replication in larger, prospectively genotyped cohorts.

Reviewer 2

Comment 4:

*More detail about the 'weakly informative prior' is needed to better understand the results and facilitate reproducibility; what is the distributional family, what were the parameters; what is the scale; is the prior the same across all models; how prior sensitivity was addressed; Show plots for the prior and posterior distributions, particularly for the lower power I/I subgroups.*

**Response**

**Prior specification.** Each genotype×dose arm is modeled with an independent binomial likelihood and a weakly informative Normal prior on the log-odds (logit) of the event rate. Explicitly, for genotype g and dose d:

*y*_gd_ ~ Binomial(*n*_gd_, *p*_gd_), logit(*p*_gd_) = *θ*_gd_, *θ*_gd_ ~ Normal(0, 5^2^)

*where y_gd_ is the number of events among n_gd_ participants, p_gd_ the event rate and θ_gd_ its log-odds; 5^2^ is the variance, i.e., a standard deviation of 5 on the logit scale.*

The same Normal(0, 5²) prior is used in every primary binomial model (preterm birth and early preterm birth; all genotype and dose strata; and the inverse-probability-weighted versions). It is weakly informative on the log-odds scale: a standard deviation of 5 corresponds to a 95% prior interval on the event probability of approximately (0.0001, 0.9999), essentially the entire unit interval, so it has minimal influence on the estimates in non-sparse strata. Its deliberate role is to keep the log-odds finite (prevent separation) in strata that contain zero or all events, where it exerts a modest regularizing influence; elsewhere the likelihood dominates the posterior. The prior is centered at 0 on the logit scale as a neutral default and is not intended to encode an expected event rate.

**Prior sensitivity.** We have now assessed sensitivity to this choice directly, varying the prior scale (its standard deviation) while holding the neutral center at 0. We refit every reported contrast under a tighter prior, Normal(0, 2.5²), and a more diffuse prior, Normal(0, 10²) which is a four-fold range of prior scale (Table 2 in S1 Appendix). The posterior probabilities were essentially unchanged: across all contrasts the largest change in any PP was 0.05, confined to the sparsest early-preterm-birth strata (1–5 events); the well-powered preterm-birth dose contrasts and the primary I-allele-carrier-versus-D/D genotype contrast changed by ≤0.005. No reported conclusion is altered by the choice of prior.

**Prior versus posterior in the low-power subgroups.** To show directly that the prior does not drive the sparse-subgroup estimates, Supplemental Figure 3 overlays the prior and posterior for the I/I subgroup, the smallest stratum (n = 106) and the one the reviewer specifically asked us to examine, for both outcomes and doses. In every case the posterior is far narrower than the prior and centered on the observed event rate, confirming that the data, not the prior, determines the estimate. As expected, the single sparsest cell (early preterm birth, 1000 mg/d; 1 event of 52) shows the widest posterior and the only appreciable prior influence, consistent with the wider credible interval reported for that stratum and the more cautious interpretation we have adopted for the I/I subgroup (see our response to R2-2).

**Table 2 in S1 Appendix. Prior-sensitivity analysis. Posterior probability (PP) for each reported contrast under the prior used and under a tighter and a more diffuse** prior on the log-odds. N(0, σ²) denotes a Normal prior with mean 0 and variance σ² (standard deviation σ) on the logit scale.

| **Contrast (PP)** | **N(0, 2.5²)** | **N(0, 5²) *** | **N(0, 10²)** | **Max change** |
| --- | --- | --- | --- | --- |
| PB D/D dose (1000 vs 200 mg) | 0.845 | 0.846 | 0.846 | 0.001 |
| PB I/D dose (1000 vs 200 mg) | 0.977 | 0.978 | 0.978 | 0.002 |
| PB I/I dose (1000 vs 200 mg) | 0.468 | 0.469 | 0.469 | 0.000 |
| EPB D/D dose (1000 vs 200 mg) | 0.098 | 0.066 | 0.056 | 0.042 |
| EPB I/D dose (1000 vs 200 mg) | 0.965 | 0.978 | 0.982 | 0.017 |
| EPB I/I dose (1000 vs 200 mg) | 0.820 | 0.855 | 0.867 | 0.047 |
| PB I-carrier vs D/D (200 mg) | 0.957 | 0.959 | 0.960 | 0.003 |
| EPB I-carrier vs D/D (200 mg) | 0.981 | 0.990 | 0.992 | 0.011 |

** the prior used in the analysis. The largest change in any PP across the three priors is 0.05, in the sparsest early-preterm-birth strata; all well-powered contrasts change by ≤0.005. PB, preterm birth; EPB, early preterm birth.*

**Supplemental Figure 3. Prior versus posterior for the I/I subgroup.**

For each I/I stratum (preterm birth and early preterm birth at 200 and 1000 mg/d), the diffuse Normal(0, 52) prior (amber) is overlaid with the posterior (blue) on the probability scale

Reviewer 2

Comment 5:

*It would be useful to show indel effects on a more complete spectrum of the fatty acid profile in the supplement, beyond the ARA/DGLA data already presented - this data would be useful for the field and provide information in the context of pregnancy. Previous reports from this group have highlighted impacts on LA, DGLA, ARA and DTA, as well as ALA, EPA, DHA. Previous reports on the ADORE trial have identified dose-x-status relationships to birth outcomes, and while this analysis is not powered to assess dose-x-status-x-genotype relationships fully, it would be useful for the field to see how these genotypes might relate to n3 status indicators and past trial reports.*

Response: We have added Table 13 in S1 Appendix which reports the complete baseline RBC-PL fatty acid composition (% total fatty acid) by FADS Indel genotype. We did not find any statistically meaningful differences in n-3 FA levels between the indel genotypes: baseline DHA (6.3%, 6.3%, 6.4%; p=0.68) and EPA were essentially identical. But we did observe an increase in ARA and its downstream elongation/ desaturation products (DTA, DPA n-6) from D/D to I/D to I/I, as predicted. We have also clarified that the ARA/DGLA ratio tracked genotype at both baseline and delivery and was not meaningfully altered by DHA dose.

**Supplemental Table 13. Baseline RBC-PL fatty acid composition (% of total fatty acids) by FADS Indel genotype**

| **Fatty acid** | **D/D (n=342)** | **I/D (n=313)** | **I/I (n=106)** | **All (n=761)** |
| --- | --- | --- | --- | --- |
| 14:0 | 0.30 ± 0.12 | 0.29 ± 0.12 | 0.26 ± 0.11 | 0.29 ± 0.12 |
| 16:0 | 20.67 ± 1.23 | 20.52 ± 1.22 | 20.56 ± 1.10 | 20.60 ± 1.21 |
| 17:0 | 0.26 ± 0.09 | 0.25 ± 0.09 | 0.25 ± 0.09 | 0.25 ± 0.09 |
| 18:0 | 10.75 ± 2.15 | 10.73 ± 2.29 | 10.96 ± 2.16 | 10.77 ± 2.21 |
| 20:0 | 0.09 ± 0.07 | 0.09 ± 0.06 | 0.08 ± 0.05 | 0.09 ± 0.06 |
| 22:0 | 0.11 ± 0.07 | 0.11 ± 0.06 | 0.10 ± 0.09 | 0.11 ± 0.07 |
| 24:0 | 0.22 ± 0.16 | 0.21 ± 0.15 | 0.21 ± 0.18 | 0.21 ± 0.16 |
| 14:1 | 0.18 ± 0.08 | 0.17 ± 0.09 | 0.15 ± 0.07 | 0.17 ± 0.08 |
| 16:1 | 0.28 ± 0.12 | 0.26 ± 0.11 | 0.23 ± 0.10 | 0.27 ± 0.11 |
| 18:1n7 | 1.35 ± 0.39 | 1.35 ± 0.40 | 1.36 ± 0.41 | 1.35 ± 0.40 |
| 18:1n9 | 10.29 ± 1.03 | 10.09 ± 1.11 | 9.93 ± 1.11 | 10.15 ± 1.08 |
| 24:1 | 0.21 ± 0.14 | 0.22 ± 0.15 | 0.22 ± 0.16 | 0.22 ± 0.15 |
| 16:1t | 0.11 ± 0.04 | 0.11 ± 0.04 | 0.10 ± 0.05 | 0.11 ± 0.04 |
| 18:1t | 0.50 ± 0.18 | 0.48 ± 0.23 | 0.45 ± 0.19 | 0.48 ± 0.20 |
| 18:2n6t | 0.37 ± 0.21 | 0.36 ± 0.20 | 0.34 ± 0.17 | 0.36 ± 0.20 |
| 18:2n6 (LA) | 14.15 ± 2.04 | 13.63 ± 1.83 | 13.46 ± 2.10 | 13.84 ± 1.98 |
| 18:3n6 (GLA) | 0.08 ± 0.05 | 0.08 ± 0.06 | 0.07 ± 0.03 | 0.08 ± 0.05 |
| 20:2n6 | 0.35 ± 0.08 | 0.33 ± 0.07 | 0.33 ± 0.07 | 0.34 ± 0.08 |
| 20:3n6 (DGLA) | 2.57 ± 0.68 | 2.19 ± 0.45 | 1.97 ± 0.35 | 2.33 ± 0.60 |
| 20:4n6 (ARA) | 18.65 ± 2.47 | 19.64 ± 2.72 | 19.97 ± 2.56 | 19.24 ± 2.64 |
| 22:4n6 (DTA) | 3.86 ± 0.86 | 4.03 ± 0.86 | 4.08 ± 0.90 | 3.96 ± 0.87 |
| 22:5n6 (DPA n-6) | 0.87 ± 0.24 | 0.89 ± 0.24 | 0.90 ± 0.25 | 0.88 ± 0.24 |
| 18:3n3 (ALA) | 0.23 ± 0.09 | 0.22 ± 0.09 | 0.20 ± 0.08 | 0.22 ± 0.09 |
| 20:5n3 (EPA) | 0.51 ± 0.29 | 0.52 ± 0.32 | 0.51 ± 0.32 | 0.52 ± 0.31 |
| 22:5n3 (DPA n-3) | 2.18 ± 0.42 | 2.22 ± 0.40 | 2.17 ± 0.40 | 2.19 ± 0.41 |
| 22:6n3 (DHA) | 6.25 ± 1.70 | 6.32 ± 1.72 | 6.41 ± 1.67 | 6.30 ± 1.70 |

Reviewer 2

Comment 6:

*The handling of race, ancestry and their interactions in the manuscript should be reviewed for consistency and clarity (few readers understand the difference). The language could be more careful to clearly define these, their potential overlap and differences, and clear attribution given to potential causal effects. Of note in the interpretation of this data, Supp Table 5 argues for a stronger effect of high dose DHA supplementation in indel carriers who are black than 'other' racial identities. Whether this is due to other ancestral genetics collinear with a black race or due to other environmental and life course factors unique to black individuals remains unclear but cuts against the language used by the authors that the indel is 'driving' ancestral differences in length somewhat. This could be untangled a bit more in the Discussion.*

Response:

We added a sentence that distinguishes race/ethnicity (i.e., Black) vs genetic ancestry (i.e. genotype).

Reviewer 2

Comment 7:

*"Circulating ARA increases in response to intake of its omega-6 fatty acid precursor, linoleic acid, up to about 4% of total energy intake. " - needs a citation*

Refs 19 and 20 at the end of the next sentence address this.

*Reviewer 3*

Comment 1:

*The sample size for the PB and especially EPB analyses is limited. Although 761 participants were included, there were only 17 EPB cases and 74 PB cases, if I counted correctly from the reported results. After the stratification by FADS genotypes and DHA dose, several groups contain very few cases. Therefore, the authors should interpret the findings more cautiously and add a discussion of this limitation.*

Response:

We have addressed this in two ways. In the Results, we now flag the sparsest estimates as uncertain and to be interpreted with caution (for example, the D/D early-preterm-birth comparison, based on only 1 and 5 events). We have also added a limitation paragraph noting that, after stratification by FADS Indel genotype and DHA dose, several subgroups contain very few outcome events (as few as 1–3 early preterm births per cell), so these estimates carry wide credible intervals and are sensitive to small perturbations; we therefore interpret the genotype-specific findings cautiously and note that they require confirmation in larger, prospectively genotyped cohorts.

*Reviewer 3*

Comment 2:

*Baseline characteristics should be compared across FADS genotype groups. This could either be added to the current Table 1 or provided as a separate table.*

Response:

We have added a table of baseline characteristics by FADS Indel genotype (Table 15 in S1 Appendix).

**Table 15 in S1 Appendix. Baseline characteristics by genotype**

|  | **Total Sample** | **D/D** | **I/D** | **I/I** |
| --- | --- | --- | --- | --- |
| **Site** | n (%) | | | |
| The University of Kansas Medical Center | 389 (51.1%) | 211 (61.7%) | 148 (47.3%) | 30 (28.3%) |
| University of Cincinnati Medical Center | 183 (24%) | 45 (13.2%) | 88 (28.1%) | 50 (47.2%) |
| Ohio State University | 189 (24.8%) | 86 (25.1%) | 77 (24.6%) | 26 (24.5%) |
| **Marital Status** |  |  |  |  |
| Unmarried/Single | 253 (33.2%) | 78 (22.8%) | 118 (37.7%) | 57 (53.8%) |
| Married/Partnered | 487 (64%) | 255 (74.6%) | 188 (60.1%) | 44 (41.5%) |
| Separated | 10 (1.3%) | 6 (1.8%) | 2 (0.6%) | 2 (1.9%) |
| Divorced | 11 (1.4%) | 3 (0.9%) | 5 (1.6%) | 3 (2.8%) |
| **Race/Ethnicity** |  |  |  |  |
| American Indian or Alaskan Native | 4 (0.5%) | 2 (0.6%) | 1 (0.3%) | 1 (0.9%) |
| Asian | 17 (2.2%) | 2 (0.6%) | 7 (2.2%) | 8 (7.5%) |
| Biracial: Asian, White | 4 (0.5%) | 1 (0.3%) | 3 (1%) | 0 (0%) |
| Biracial: Black, White | 8 (1.1%) | 5 (1.5%) | 1 (0.3%) | 2 (1.9%) |
| Black or African American | 156 (20.5%) | 23 (6.7%) | 77 (24.6%) | 56 (52.8%) |
| Hispanic | 190 (25%) | 133 (38.9%) | 50 (16%) | 7 (6.6%) |
| Multiracial: Black, Asian, White | 1 (0.1%) | 0 (0%) | 0 (0%) | 1 (0.9%) |
| Multiracial: Black, Native American, White | 2 (0.3%) | 1 (0.3%) | 1 (0.3%) | 0 (0%) |
| Native Hawaiian or other Pacific Islander | 1 (0.1%) | 1 (0.3%) | 0 (0%) | 0 (0%) |
| Other | 4 (0.5%) | 0 (0%) | 3 (1%) | 1 (0.9%) |
| White | 374 (49.1%) | 174 (50.9%) | 170 (54.3%) | 30 (28.3%) |
| **Maternal Education** |  |  |  |  |
| less than HS | 134 (17.6%) | 84 (24.6%) | 36 (11.5%) | 14 (13.2%) |
| HS grad/GED | 161 (21.2%) | 58 (17%) | 76 (24.3%) | 27 (25.5%) |
| some college or tech school | 141 (18.5%) | 50 (14.6%) | 67 (21.4%) | 24 (22.6%) |
| bachelor's degree | 167 (21.9%) | 73 (21.3%) | 69 (22%) | 25 (23.6%) |
| master's degree | 106 (13.9%) | 52 (15.2%) | 43 (13.7%) | 11 (10.4%) |
| doctorate | 52 (6.8%) | 25 (7.3%) | 22 (7%) | 5 (4.7%) |
| **Household Income** |  |  |  |  |
| unknown | 18 (2.4%) | 7 (2%) | 7 (2.2%) | 4 (3.8%) |
| <$15,000 | 162 (21.3%) | 45 (13.2%) | 84 (26.8%) | 33 (31.1%) |
| $15,000-$24,999 | 112 (14.7%) | 61 (17.8%) | 38 (12.1%) | 13 (12.3%) |
| $25,000-$49,999 | 134 (17.6%) | 72 (21.1%) | 44 (14.1%) | 18 (17%) |
| $50,000-$99,999 | 135 (17.7%) | 56 (16.4%) | 60 (19.2%) | 19 (17.9%) |
| $100,000-$150,000 | 121 (15.9%) | 62 (18.1%) | 52 (16.6%) | 7 (6.6%) |
| ≥$150,000 | 79 (10.4%) | 39 (11.4%) | 28 (8.9%) | 12 (11.3%) |
|  |  |  |  |  |
| **Ever Smoke (yes)** | 194 (25.5%) | 76 (22.2%) | 94 (30%) | 24 (22.6%) |
| smoke 6mo prior (yes) | 98 (12.9%) | 33 (9.6%) | 51 (16.3%) | 14 (13.2%) |
| current smoker (yes) | 40 (5.3%) | 10 (2.9%) | 24 (7.7%) | 6 (5.7%) |
| **Pregnancy Hx** |  |  |  |  |
| primagravida | 219 (28.8%) | 94 (27.5%) | 106 (33.9%) | 19 (17.9%) |
| prior PTB (yes) | 104 (13.7%) | 47 (13.7%) | 42 (13.4%) | 15 (14.2%) |
| prior ePTB (yes) | 45 (5.9%) | 18 (5.3%) | 19 (6.1%) | 8 (7.5%) |
| **Tx Group** |  |  |  |  |
| 200mg | 365 (48%) | 151 (44.2%) | 160 (51.1%) | 54 (50.9%) |
| 1000mg | 396 (52%) | 191 (55.8%) | 153 (48.9%) | 52 (49.1%) |
|  | mean ± SD | | | |
| Age at enrollment | 30.3 ± 5.7 | 30.7 ± 5.5 | 30.1 ± 5.8 | 29.6 ± 5.6 |
| Baseline DHA status | 6.3 ± 1.7 | 6.3 ± 1.7 | 6.3 ± 1.7 | 6.4 ± 1.7 |
| Pre-pregnancy BMI | 28.1 ± 7.2 | 27.4 ± 6.6 | 28.4 ± 7.4 | 29.7 ± 7.8 |

*Reviewer 3*

Comment 3:

*Since FADS genotype was not measured or used for randomization before the study, potential confounding across genotype groups needs to be assessed. Possible* *confounders should be considered in genotype analysis models.*

First, we refer the reviewer to the comments to reviewer 2 above regarding prospective-retrospective studies and also those in the text. Briefly, randomization to dose (200 or 1,000 mg/d) extend to characteristics of all participants. The FADS Indel genotypes were unknown but were randomized just as any characteristic in the new Table 15 in S1 Appendix. Inference follows that randomization. For instance, the D/D genotype was randomized to 200 and 1,000 mg/d even though it was unmeasured and thus blinded to everyone, which we further note eliminates any chance of experimenter bias.

Continuing from the last comment, we found genotype groups were similar on the major obstetric risk factors for preterm birth, including prior preterm birth (13.7%, 13.4%, and 14.2% in D/D, I/D, and I/I), prior early preterm birth, maternal age, and baseline DHA status and randomized dose. To test whether socioeconomic differences (income, education, marital status) accounted for the genotype–preterm birth association, we additionally report preterm birth by genotype within each socioeconomic category (Table 16 in S1 Appendix); I-allele carriers had higher preterm birth than D/D within every stratum, indicating the association is not fully explained by socioeconomic differences. An additional paragraph at the end of discussion is added to address this issue.

**Table 16** **in S1 Appendix. Preterm birth rates by socioeconomic category and genotype**

|  | **D/D** | **I/D** | **I/I** |
| --- | --- | --- | --- |
| **Income <$15k** | 2/45 (4%) | 14/84 (17%) | 4/33 (12%) |
| **Income ≥$15k** | 22/290 (8%) | 22/222 (10%) | 9/69 (13%) |
| **Unmarried** | 8/87 (9%) | 15/125 (12%) | 11/62 (18%) |
| **Married** | 16/255 (6%) | 21/188 (11%) | 3/44 (7%) |
| **Education <bachelor** | 17/192 (9%) | 24/179 (13%) | 11/65 (17%) |
| **Education ≥bachelor** | 7/150 (5%) | 12/134 (9%) | 3/41 (7%) |
| **Ever smoker** | 7/76 (9%) | 13/94 (14%) | 2/24 (8%) |
| **Never smoker** | 17/266 (6%) | 23/219 (11%) | 12/82 (15%) |
| **Obese (BMI≥30)** | 8/93 (9%) | 18/114 (16%) | 8/48 (17%) |
| **Non-obese** | 16/246 (7%) | 17/192 (9%) | 6/58 (10%) |

*Reviewer 3*

Comment 4:

*Based on the authors' previous study, it seems that baseline DHA levels were also significantly associated with EPB and PB rates. Did the authors investigate the associations between FADS genotype and baseline DHA, ARA/DGLA levels? It is also not clear whether baseline DHA and ARA-related measures were included in the genotype analysis models. This should be clarified.*

Response:

The association between FADS Indel genotype and baseline arachidonic-acid synthesis is shown in Figure 5 and Tables 12 and 13 in S1 Appendix: the ARA/DGLA ratio increased across D/D → I/D → I/I, confirming the expected allele-dose effect on ARA synthesis. In contrast, baseline DHA did not differ by genotype (6.3%, 6.3%, and 6.4% in D/D, I/D, and I/I; Tables 13 and 15 in S1 Appendix. Regarding the models, the primary genotype analyses were treatment-specific Bayesian binomial models fitted without covariates, baseline DHA and ARA/DGLA were therefore not included in these models, but they were incorporated in the inverse-probability-weighting sensitivity analysis (Supplemental Table 5), which balanced baseline DHA and ARA/DGLA across the analyzed sample and yielded virtually identical posterior probabilities. We have expanded the method section to explain and prove the validity of our Bayesian model in greater detail.

**Table 5** **in S1 Appendix.** Covariate balance and composition in unweighted and IPW weighted samples. Analyzed (genotyped+consented) and ESS (IPW weighted) for the 200mg arm and overall cohort.

|  | **Covariate fatty acid standardized mean differences (SMDs)** | | **Total variation distance** |
| --- | --- | --- | --- |
|  |  |  |  |
| Weighting | Baseline ARA/DGLA | Baseline %DHA | Center×Maternal race×Treatment |
| Unweighted | -0.056 | -0.045 | 0.104 |
| Weighted | -0.057 | -0.052 | 0.1056 |
|  |  |  |  |
|  | 200mg Treatment group | Total |  |
| Analyzed (S=1) | 365 | 761 |  |
| ESS (weighted) | 364.6 | 760.8 |  |

*Reviewer 3*

Comment 5:

*I would suggest toning down wording such as "genotype-directed" DHA dosing. The study supports the possibility that FADS genotype may be involved in the process in response to DHA supplementation, but the genotype-guided dosing strategy was not prospectively or functionally tested. The authors should also add a limitation stating that larger cohorts and prospective validation are needed before FADS genotype can be used as a functional biomarker to guide DHA dosing in pregnancy.*

Response:

We have softened the tone of “genotype-directed” and “genotype-guided” to “genotype-informed”. We’ve also toned down the causal phrases used in the manuscript. We also discussed related matters in the limitations section of the Discussion.

*Reviewer 3*

Minor comment 1:

*As the methods indicate that race and ethnicity were self-reported, it would be better to avoid using the term "ancestry".*

Response:

Thanks for making this distinction. Ancestry refers to genotype and genome. Ethnicity refers to self-reported race as defined by the US federal government and other entities.

*Reviewer 3*

Minor comment 2:

*The data availability statement is inconsistent. In the submission checklist, the authors state that all data are fully available without restriction. However in the manuscript it says "We are willing to share deidentified data from the study, including data from individual participants with a signed data access agreement that includes the study principal investigators and is contingent on their approval of the planned use of the data."*

Response:

We reconciled them in favor of the original manuscript.

Response to General Editorial Requests

Comment 1:

We ask every co-author listed on the manuscript to fill in a contributing author statement, making sure to declare all competing interests. If any of the co-authors have not filled in the statement, we will remind them to do so when the paper is revised. If all statements are not completed in a timely fashion this could hold up the re-review process. If new competing interests are declared later in the revision process, this may also hold up the submission. Should there be a problem getting one of your co-authors to fill in a statement we will be in contact. Please do not add or remove authors without first discussing this with the handling editor. You can see our competing interests policy here: <http://journals.plos.org/plosmedicine/s/competing-interests>.

Response:

All co-authors have completed the contributing-author statement and declared their competing interests. No authors have been added or removed. Competing interests are stated in the manuscript (Declarations – Competing interests): the authors have declared that no competing interests exist.

Comment 2:

Please upload any figures associated with your paper as individual TIF or EPS files with 300dpi resolution at resubmission; please read our figure guidelines for more information on our requirements: http://journals.plos.org/plosmedicine/s/figures. While revising your submission, we strongly recommend that you use PLOS's NAAS tool (https://ngplosjournals.pagemajik.ai/artanalysis) to test your figure files. NAAS can convert your figure files to the TIFF file type and meet basic requirements (such as print size, resolution), or provide you with a report on issues that do not meet our requirements and that NAAS cannot fix.

Response:

All figures (Figs 1–6 and S1–S11) are supplied as individual files at 300 dpi. Each figure was processed through the PLOS NAAS tool, and the NAAS-processed files are uploaded with this resubmission.

Comment 3:

Please ensure that the paper adheres to the PLOS Data Availability Policy (see http://journals.plos.org/plosmedicine/s/data-availability), which requires that all data underlying the study's findings be provided in a repository or as Supporting Information. For data residing with a third party, authors are required to provide instructions with contact information (web or email address) for obtaining the data. Please note that a study author cannot be the contact person for the data. PLOS journals do not allow statements supported by "data not shown" or "unpublished results." For such statements, authors must provide supporting data or cite public sources that include it.

Response:

The paper adheres to the policy. De-identified individual-level data underlying the findings are available on request through a non-author contact (Declarations – Data Availability). The manuscript contains no statements supported by “data not shown” or “unpublished results.”

Comment 4:

We expect all researchers with submissions to PLOS in which author-generated code underpins the findings in the manuscript to make all author-generated code available without restrictions upon publication of the work. In cases where code is central to the manuscript, we require the code to be made available as a condition of publication. Authors are responsible for ensuring that the code is reusable and well documented. Please make any custom code available, either as part of your data deposition or as a supplementary file. Please add a sentence to your data availability statement regarding any code used in the study, e.g. "The code used in the analysis is available from Github [URL] and archived in Zenodo [DOI link]" Please review our guidelines at https://journals.plos.org/plosmedicine/s/materials-software-and-code-sharing and ensure that your code is shared in a way that follows best practice and facilitates reproducibility and reuse. Because Github depositions can be readily changed or deleted, we encourage you to make a permanent DOI'd copy (e.g. in Zenodo) and provide the URL.

Response:

All author generated analysis code is provided as Supporting Information (S1 Code). We have stated this in the Data availability statement.

Comment 5:

Data Availability statement: A study author cannot be the contact person. If the data are not freely available, please describe briefly the ethical, legal, or contractual restriction that prevents you from sharing it. Please also include an appropriate contact (web or email address) for inquiries

Response:

The data contact (S. Bartlow-Robbins, sarahbartlow@utexas.edu) is not an author and will share data on request.

Comment 6:

Please include your Statistical Analysis Plan in the supplementary files.

Response: The statistical analysis plan is included as Supporting Information (S1 Statistical Analysis Plan).

Comment 7:

Please ensure that the study is reported according to the CONSORT guideline and include the completed checklist as Supporting Information. When completing the checklist, please use section and paragraph numbers, rather than page numbers. Please add the following statement, or similar, to the Methods: "This study is reported as per the CONsolidated Standards of Reporting Trials (CONSORT) guideline (S1 Checklist)."

Response:

Completed checklists are provided as S2 Checklist (CONSORT) and S1 Checklist (STROBE). We have added the sentence “Our reporting follows the CONSORT 2025 statement and the STROBE guidelines.” To the statistical analysis section of the manuscript.

Comment 8:

Abstract: Please structure your abstract using the PLOS Medicine headings (Background, Methods and Findings, Conclusions). Please combine the Methods and Findings sections into one section.

Response: We have combined the Methods and Findings sections.

Comment 9:

At this stage, we ask that you include a short, non-technical Author Summary of your research to make findings accessible to a wide audience that includes both scientists and non-scientists. The Author Summary should immediately follow the Abstract in your revised manuscript. This text is subject to editorial change and should be distinct from the scientific abstract. Ideally each sub-heading should contain 2-3 single sentence, concise bullet points containing the most salient points from your study. In the final bullet point of 'What Do These Findings Mean?', please include the main limitations of the study in non-technical language. Please see our author guidelines for more information: https://journals.plos.org/plosmedicine/s/revising-your-manuscript#loc-author-summary.

Response:

We have added a non-technical summary following the abstract and have stated the main limitation in one of the bullet points.

Comment 10:

Please express the main results with 95% CIs as well as p values. When reporting p values please report as p<0.001 and where higher as the exact p value p=0.002, for example. Throughout, suggest reporting statistical information as follows to improve clarity for the reader "22% (95% CI [13%,28%]; p</=)". Please be sure to define all numerical values at first use.

Response:

We have adhered to these guidelines. Effects are reported as posterior means with 95% credible intervals (CrI) and posterior probabilities (PP). The manuscript reserves “95% CI” for frequentist comparisons and explicitly distinguishes it from CrI. All numerical values are defined at first use.

Comment 11:

Please include page numbers and line numbers in the manuscript file. Use continuous line numbers (do not restart the numbering on each page).

Response:

The manuscript file now carries page numbers and continuous line numbers.

Comment 12:

Please cite the reference numbers in square brackets. Citations should precede punctuation.

Response:

In-text citations are given as reference numbers in square brackets placed before punctuation.

Comment 13:

Please provide titles and legends for all figures and tables (including those in Supporting Information files).

Response: Titles and legends are provided for every figure (Fig 1-6 and Fig S1–S11), and main tables in the manuscript, supporting information tables are combined as S1 Appendix Tables 1–16.

Comment 14:

Please define all abbreviations used in each figure/table (including those in Supporting Information files).

Response: Abbreviations are defined within each figure and table legend.

Comment 15:

Please consider avoiding the use of red and green in order to make your figure more accessible to those with color blindness.

Response: Main text figures are rendered in black/grey when possible, we do not use red/ green combinations in any of our figures.

Comment 16:

Please cite your Supporting Information as outlined here: <https://journals.plos.org/plosmedicine/s/supporting-information>

Response:

All Supporting Information is cited in the text in PLOS format (S1 Appendix; S1 Protocol; S1 and S2 Checklist; S1 Statistical Analysis Plan; S1 Code; Figs S1–S11), with legends listed under “Supporting Information Captions” in the main manuscript.

Comment 17:

Please ensure that journal name abbreviations match those found in the National Center for Biotechnology Information (NCBI) databases (http://www.ncbi.nlm.nih.gov/nlmcatalog/journals), and are appropriately formatted and capitalised.

Response:

Journal names have been abbreviated to match the NCBI NLM Catalog and formatted and capitalized accordingly.

Comment 18: Where website addresses are cited, please include the complete URL and specify the date of access (e.g. [accessed: 12/06/2023]).

Response:

Cited websites include the complete URL and the date of access (e.g., refs 4 and 12).

Comment 19: If the analyses were not prespecified in the RCT protocol, please define them in the Methods as post hoc. Post-hoc comparisons should be presented as hypothesis generating rather than conclusive.

Response:

We have defined the study throughout as a post-hoc secondary analysis conducted under a prospective–retrospective framework.

Comment 20:

In keeping with our commitment to Open Science, please include the study protocol document and analysis plan (including any amendments) as Supporting Information to be published with the manuscript if accepted.

Response:

The study protocol (S1 Protocol) and the analysis plan (S1 Statistical Analysis Plan) are included as Supporting Information, cited in the Methods, with legends at the end of the manuscript.

Comment: 21

Abstract: Please include the study design, population and setting, number of participants, years during which the study took place (enrollment and follow up), length of follow up, and main outcome measures.

Response: All components are now included in the abstract.

Comment 22:

In the manuscript text, please indicate: (1) the specific hypotheses you intended to test, (2) the analytical methods by which you planned to test them, (3) the analyses you actually performed, and (4) when reported analyses differ from those that were planned, transparent explanations for differences that affect the reliability of the study's results. If a reported analysis was performed based on an interesting but unanticipated pattern in the data, please be clear that the analysis was data driven.

Response: All four points above are described in detail in the manuscript.

Comment 23:

Please state in the Methods section whether the study had a prospective protocol or analysis plan. If a prospective analysis plan (from your funding proposal, IRB or other ethics committee submission, study protocol, or other planning document written before analyzing the data) was used in designing the study, please include the relevant document(s) with your revised manuscript as a Supporting Information file to be published alongside your study and cite it in the Methods section. A legend for this file should be included at the end of your manuscript. If no such document exists, please make sure that the Methods section transparently describes when analyses were planned, and when/why any data-driven changes to analyses took place. Changes in the analysis, including those made in response to peer review comments, should be identified as such in the Methods section of the paper, with rationale.

Response:

The Method section states that a prospective protocol and statistical analysis plan preceded data analysis and cite these documents, which are included as Supporting Information (S1 Protocol; S1 Statistical Analysis Plan) with end of manuscript legends. Analyses added during revision: stabilized inverse-probability weighting, a hierarchical partial-pooling model, prior-sensitivity analyses, and outcome-tilted (tipping-point) sensitivity analyses, are described in the Statistical Analysis section and identified as additions made in response to peer review, with their rationale.
