# Supplementary material for: Genotype-informed docosahexaenoic acid dosing based on the FADS Indel to reduce preterm and prolonged gestation: A secondary analysis of the ADORE randomized trial
Source: PLoS Med. 2026 Oct 8;23(10):e1005274. doi: 10.1371/journal.pmed.1005274 (PMC13660990; doi:10.1371/journal.pmed.1005274)
Supplement: Supplementary file 9 [file pmed.1005274.s022.docx]

**Point-by-Point Response to Reviewers**

**Reviewer #1**

We thank the reviewer for the thorough re-reading.

**Comment 1:** My previous comment S10 (large number of excluded participants listed only as 'other reasons') is claimed to be addressed in the authors' response letter, but Figure 1 still lists the exact same number for 'other reasons' (3,966/10,497). This is 38% of those screened, which is relevant to the generalizability argument made for supplementation in an unselected population.

**Response:** We apologize for not adequately addressing this point in the previous revision. The screening portion of Figure 1 was reproduced from the CONSORT diagram of the parent ADORE trial, including the 3,966 participants categorized as “other reasons.” Unfortunately, the original screening records do not permit this category to be retrospectively broken down into more specific reasons. We can clarify, however, that this category predominantly represents women who were not approached by study staff and therefore were not assessed for interest or eligibility. This occurred particularly at the Ohio sites, where staffing limitations sometimes prevented the research team from approaching all potentially eligible women attending clinic.

**Comment 2**

*My main new comment is something I did not fully appreciate on my first reading. The headline finding — and the claim in the title — is an interaction: that the effect of DHA dose differs by FADS Indel genotype. However, this is reported only as two separate genotype-stratified analyses, with a stronger dose effect in I-allele carriers than in D/D. The interaction itself is never estimated. While (frequentist) null hypothesis significance testing is not what the authors did here, this is nevertheless an instance of the "difference in significance is not (necessarily) a significant difference" fallacy. The authors' response to Reviewer 2's second comment touches on this tangentially, but the paper would be strengthened if the authors explained why no genotype × dose interaction was interrogated directly, and reported a posterior probability for the interaction itself. As I understand the parameterisation described in the response to my M1 comment from the previous round —
independent arm-level log-odds with no pooling across arms — this should require no new model fit. The posterior for the interaction is a contrast of draws already available: the PP that (θ_I-carrier,200 − θ_I-carrier,1000) > (θ_D/D,200 − θ_D/D,1000). This should be done for both PB and EPB outcomes, and for the post-40-week outcome if that claim is retained in the title (see comment #5 below). I appreciate that the sample size may well leave the interaction inconclusive, particularly for PB where the two genotype-specific estimates point in the same direction. An inconclusive interaction is an acceptable result and would not undermine the paper, but it would need to be reflected in the title, abstract, and Conclusions, which currently assert a genotype-dependent dose effect as established.*

**Response:** We agree, and we thank the reviewer for specifying the estimator. We computed the posterior probability of the genotype × dose interaction exactly as proposed , with no change to the model. The results are:

| **Outcome** | **Interaction log-OR [95% CrI]** | **PP (log-odds scale)** |
| --- | --- | --- |
| PB (<37 wk) | 0.08 [−0.93, 1.12] | 0.56 |
| EPB (<34 wk) | 3.17 [0.58, 6.27] | 0.99 |
| Prolonged gestation (≥40 wk) | 0.78 [0.04, 1.54] | 0.98 |

The interaction is therefore credible for EPB and prolonged gestation, and inconclusive for PB: high-dose DHA reduced PB to a similar extent in both genotype groups (RR 0.64 in I-allele carriers vs 0.67 in D/D). We have made the following changes: (1) a new Results paragraph reports the interaction for all three outcomes with log-odds ratios, 95% CrIs, and PPs; (2) the Abstract now states: These dose effects credibly differed by genotype for EPB and prolonged gestation (genotype × dose interaction PP=0.99 and 0.98). For PB, both genotypes reduced similarly when with high-dose DHA (PP=0.56); (4) the Conclusions state the same result

**Comment 3 (IPW restricted to the 200 mg arm)**

*More minor, but not clear to me why IPW was restricted to the 200 mg dose group — as Fig 1 shows, non-consent to genetic research occurred in both groups.*

**Response:** The reviewer is correct that non-consent occurred in both arms. Table 4 in S1 Appendix now reports unweighted and IPW-weighted posteriors for both treatment arms, and the Methods state that weights were constructed separately within each treatment group.

**Comment 4 (828 vs 840 at l.184)**

*l.184: not fully clear to me where the 828 comes from when looking at Figure 1; should this be 840? From the figure, 1,032 − (103 + 89) = 840 consenting to genetic research, and 840 − (41 + 38) = 761 with complete data, which matches the analysed sample exactly.*

**Response:** We thank the reviewer for checking this arithmetic carefully, and we apologize for the ambiguity. The reviewer's calculation accurately reflected Figure 1 as previously drawn; the apparent discrepancy arose because the figure applied the two post-completion exclusions in a different order than the text reports them, not from an error in either count. Verified against the source consent records: of the 1,032 participants who completed the trial with birth data available, optional genetic-research consent was obtained for 828 (431 in the 1000 mg arm; 397 in the 200 mg arm) and not obtained for 204 (109; 95). Among the 828 consenting participants, 67 (35; 32) had no blood sample available for FADS Indel genotyping, leaving 761 analyzed (396; 365): 1,032 − 204 = 828; 828 − 67 = 761. Figure 1 previously listed 'no blood draw' (n=41; n=38) before 'did not consent' (n=103; n=89); under that sequential accounting, the 12 participants (6 per arm) who had neither a blood draw nor consent were counted in the 'no blood draw' boxes, so subtracting only the consent boxes from 1,032 yields 840: the 828 consenters plus those 12. We have revised Figure 1 to apply the exclusions in the same order as the text: 'Did not consent to genetic research' (n=109; n=95), followed by 'No blood sample available for genotyping' (n=35; n=32). The per-arm analysis totals are unchanged (396 and 365), and the consent total of 828 can now be read directly from the figure. No change to the manuscript text was required.

**Comment 5 (post-40-week outcome in the title; inconsistent terminology)**

*The post-40-week outcome appears in the title but is not listed among the primary or secondary outcomes in the Outcomes section, and it is described variously as 'prolonged gestation', 'late-term' and 'post-term'. Might be best to remove it from the title if it is not a primary outcome and use consistent terminology for it throughout the manuscript.*

**Response:** We have added prolonged gestation as a secondary outcome in the outcomes section of the manuscript, we have maintained “prolonged gestation” in the title as we do consider it as an integral part of our dose matching strategy. We now consistently use prolonged gestation to refer to birth ≥40 weeks across the manuscript.

**Point by point response to editors**

**Comment 1:**

*Please remove all causal language in the manuscript, including the title, Abstract and main text.*

**Response:** We revised the title, Abstract, Author Summary, and main text to replace causal claims with associative or descriptive language.

**Comment 2**

*Please remove reference to race alone as it is a social construct. Use of 'race/ethnicity' could be used. In the Abstract, please state that Black women had higher rates of PB.*

**Response:** The Abstract now states that Black women had higher rates of PB and EPB.

**Comment 3**

*Please use patient-centered language throughout.*

**Response:** We revised the manuscript to use patient-centered language, referring to participants or women with particular characteristics rather than defining individuals by genotype, outcome, or race/ethnicity.

**Comment 4**

*Please temper claims in the Abstract related to preterm birth and ancestry, as not all forms of preterm birth have been shown to be linked to ancestry.*

**Response:** Abstract is revised and tempered.

**Comment 5**

*In the first bullet point of the Author Summary, line 58, please state if this comment related to global evidence or US-based evidence.*

**Response:** We clarified that the evidence described in the first Author Summary bullet is global.

**Comment 6**

*In the 3rd bullet point of the Author Summary, please revise the statement (e.g. replace predicts with associates), given that the data presented do not formally predict who would benefit from high dose DHA, which the framing implies.*

**Response:** We revised the third Author Summary bullet to state that FADS Indel genotype was “associated with response to higher-dose DHA,” rather than predicting who would benefit.

**Comment 7**

*Please use associative language in the Author Summary.*

**Response:** We revised the Author Summary throughout to use associative language.

**Comment 8**

*Temper line 78.*

**Response:** We tempered the statement to indicate that, in this post hoc analysis, genotype-matched dosing was associated with the highest observed proportion of births between 37 and <40 weeks.

**Comment 9 & 10**

*The Discussion is >2500 words. Please reduce length, removing any repetition and temper claims, given that the study is a post hoc analysis.*

*Please include a discussion of study limitations.*

**Response:** The discussion section now has 2404 words, with a limitation paragraph.

**Comment 11**

*Please confirm that the original consent documentation permitted extended use of the data or samples collected in the trial.*

**Response:** We confirm and have added it to the Method section.

**Comment 12**

*At this stage, we ask that you include a short, non-technical Author Summary of your research to make findings accessible to a wide audience that includes both scientists and non-scientists. The Author Summary should immediately follow the Abstract in your revised manuscript. This text is subject to editorial change and should be distinct from the scientific abstract. Ideally each sub-heading should contain 2-3 single sentence, concise bullet points containing the most salient points from your study. In the final bullet point of ‘What Do These Findings Mean?’ Please include the main limitations of the study in non-technical language.*

**Response:** We have modified the main limitation point to be more non-technical.

**Comment 13**

*Please confirm that your title complies with PLOS Medicine's style. Your title must be nondeclarative and not a question. It should begin with main concept if possible. "Effect of" should be used only if causality can be inferred, i.e., for an RCT. Please place the study design ("A randomized controlled trial," "A retrospective study," "A modelling study," etc.) in the subtitle (ie, after a colon).*

**Response:** Our title is compliant.

**Comment 14**

*Please confirm that your abstract complies with our requirements, including format (three sections: Background, Methods and Findings, and Conclusions) and providing all the information relevant to this study type.*

**Response:** We have added a limitations paragraph to the method and findings section of the abstract, it is now compliant.

**Comment 15**

*Please ensure that the Introduction ends with a clear description of the study question or hypothesis.*

**Response:** We have modified the last paragraph of the introduction to clearly describe our hypothesis.

**Comment 16**

*Please ensure that all abbreviations are defined at first use throughout the text.*

**Response:** All abbreviations are defined at first appearance.

**Comment 17**

*Please confirm that all numbers presented in the abstract are present and identical to numbers presented in the main manuscript text.*

**Response:** We have unified any discrepancies.

**Comment 18**

*Please include a statement regarding use or non-use of AI tools and technologies at the end of the Methods. Please specify the name(s) and version(s) of any tool(s) used and a description of their use, including how the AI-assisted or generated content was evaluated by authors, and a clear statement of which aspects of the study, article contents, data, or supporting files were affected or generated by AI tool usage. Please also include a statement specifying who retains responsibility for the final manuscript. See our guidelines for more information:* [*https://journals.plos.org/plosmedicine/s/ethical-publishing-practice#loc-artificial-intelligence-tools-and-technologies*](https://journals.plos.org/plosmedicine/s/ethical-publishing-practice#loc-artificial-intelligence-tools-and-technologies)*.*

**Response:** We have added a dedicated paragraph regarding AI use at the end of the methods section.

**Comment 19**

*Please review your text for claims of novelty or primacy (e.g. 'for the first time') and remove this language. In addition, please check that any use of statistical terms (such as trend or significant) are supported by the data, and if not please remove them.*

**Response:** We have removed claims of novelty or primacy.

**Comment 20**

*Please remove the 'conclusions' subheading from the discussion. Please also remove any other subheadings from the discussion.*

**Response:** Removed

**Comment 21**

*Statistical reporting: Please revise throughout the manuscript, including tables and figures.
- Please report statistical information as follows to improve clarity for the reader ""22% (95% CI [13,28]; p</=)"".
- Please separate upper and lower bounds with commas instead of hyphens as the latter can be confused with reporting of negative values.
- Please repeat statistical definitions (HR, CI etc.) for each set of parentheses."*

**Response:** We have modified all statistical reporting to the format above.

**Comment 22**

*In the last sentence of the Abstract Methods and Findings section, please describe the main limitation(s) of the study's methodology.*

**Response:** We have added a limitation paragraph.

**Comment 23**

*In the abstract, please include the important dependent variables that are adjusted for in the analyses.*

**Response:** The primary analyses were unadjusted; therefore, no covariates were included in these estimates

**Comment 24**

*In the author summary, please revise formatting and ensure you use bullet points.*

**Response:** The original author summary is in bullet points format.

**Comment 25**

*In the author summary, in the final bullet point of 'What Do These Findings Mean?', please include the main limitations of the study in non-technical language.*

**Response:** We have modified the final point to be more non-technical friendly.

**Comment 26**

*The funding statement should include: specific grant numbers, initials of authors who received each award, URLs to sponsors’ websites. Also, please state whether any sponsors or funders (other than the named authors) played any role in study design, data collection and analysis, the decision to publish, or preparation of the manuscript. If they had no role in the research, include this sentence: “The funders had no role in study design, data collection and analysis, decision to publish, or preparation of the manuscript.*

**Response:** Our current funding statement contains the complete information.

**Comment 27**

*All authors must declare their relevant competing interests per the PLOS policy, which can be seen here:* [*https://journals.plos.org/plosmedicine/s/competing-interests*](https://journals.plos.org/plosmedicine/s/competing-interests) *For authors with ties to industry, please indicate whether any of the interests has a financial stake in the results of the current study.*

**Response:** All authors declare no competing interests, as stated in the original manuscript.

**Comment 28**

*The Data Availability Statement (DAS) requires revision.*

**Response:** All relevant data is available within the manuscript and Supporting Information files. The de-identified participant-level analytic dataset underlying the reported findings is provided as S1 Data, and all author-generated analysis code is provided as S1 Code.

**Comment 29**

*Please provide titles and legends for all figures and tables (including those in Supporting Information files). Please define all acronyms used in each figure or table in its corresponding legend.*

**Response:** All current tables/ figures contain title and legends; we have added definitions to acronyms used in some tables.

**Comment 30**

*In Kaplan-Meier curve(s) please provide the number at risk for each time interval.*

**Response:** The current Kaplan-Meier curves contain a table directly beneath that shows the numbers at risk at each point.

**Comment 31**

*Please provide the unadjusted comparisons as well as the adjusted comparisons in all relevant Tables*

**Response:** The analyses reported in the relevant tables are unadjusted; therefore, corresponding adjusted comparisons are not applicable

**Comment 32**

*Please specify the variables controlled for in all relevant Tables*

**Response:** No covariates were controlled for in the tabulated primary analyses; therefore, no adjustment variables apply.

**Comment 33:**

*Please ensure that the study is reported according to the STROBE guideline, and include the completed STROBE checklist as Supporting Information.  Please add the following statement, or similar, to the Methods: ""This study is reported as per the Strengthening the Reporting of Observational Studies in Epidemiology (STROBE) guideline (S1 Checklist).*

**Response:** We completed this request during last round of revision; we will upload an updated STROBE checklist.

**Comment 34**

*Did your study have a prospective protocol or analysis plan? Please state this (either way) early in the Methods section.*

**Response:** The parent ADORE trial had a prospective protocol and statistical analysis plan. The present genotype-based analysis was post hoc and was not prespecified. This is now stated early in the Methods, and the statistical analysis plan and analysis code are provided as Supporting Information.
