# Supplementary material for: Genotype-informed docosahexaenoic acid dosing based on the FADS Indel to reduce preterm and prolonged gestation: A secondary analysis of the ADORE randomized trial
Source: PLoS Med. 2026 Oct 8;23(10):e1005274. doi: 10.1371/journal.pmed.1005274 (PMC13660990; doi:10.1371/journal.pmed.1005274)
Supplement: Peer review history

# Editor report: Decision Letter 0

**DOI:** 10.1371/journal.pmed.1005274.r001

**Authors:** Evangelia Fourli

19 Feb 2026

Dear Dr Brenna,

Thank you for submitting your manuscript entitled "High-dose DHA and preterm birth in high arachidonic acid synthesizing FADS Indel allele carriers: A randomized clinical trial" for consideration by PLOS Medicine.

Your manuscript has now been evaluated by the PLOS Medicine editorial staff, and I am writing to let you know that we would like to send your submission out for external peer review.

However, before we can send your manuscript to reviewers, we need you to complete your submission by providing the metadata that is required for full assessment. To this end, please login to Editorial Manager where you will find the paper in the 'Submissions Needing Revisions' folder on your homepage. Please click 'Revise Submission' from the Action Links and complete all additional questions in the submission questionnaire.

Moreover, the editorial team has the following requests to facilitate peer-review and improve clarity of your work. Please revise the title to reflect the methodology of the work described here, which is accurately described in the abstract, i.e. this is a secondary analysis of the ADORE trial and not an RCT itself. Furthermore, please provide a sentence long description, preferably in the introduction, of the physiological difference between the FADS genotypes included in your work for readers who are not familiar with this specific polymorphism and its effects (for example: circulating ARA is increasing in the order of D/D....).

For clinical studies, please upload a copy of your trial study protocol as a supporting information file. The study protocol should be the version submitted for approval to the institutional review board or ethics committee, should include any amendments to the study protocol, as well as the date of their approval by the institutional review or ethics committee. Please also detail any deviations from the study protocol in the Methods section of your manuscript. The editors will consider the protocol and study conduct prior to a final decision for external review.

Please re-submit your manuscript within two working days, i.e. by Feb 23 2026 11:59PM.

Login to Editorial Manager here: https://www.editorialmanager.com/pmedicine

Once your full submission is complete, your paper will undergo a series of checks in preparation for peer review. Once your manuscript has passed all checks it will be sent out for review.

Feel free to email us at plosmedicine@plos.org if you have any queries relating to your submission.

Kind regards,

Evangelia Fourli, Ph.D.

Associate Editor

PLOS Medicine

# Peer review: Decision Letter 1

**DOI:** 10.1371/journal.pmed.1005274.r002

**Authors:** Louise Gaynor-Brook

8 Jun 2026

Dear Dr Brenna,

Many thanks for submitting your manuscript "Genotype-directed docosahexaenoic acid (DHA) dosing based on the FADS Indel reduces preterm and post-40-week birth: A secondary analysis of the ADORE randomized trial" (PMEDICINE-D-26-00537R1) to PLOS Medicine. The paper has been reviewed by subject experts and a statistician; their comments are included below and can also be accessed here: [LINK]

After discussing the paper with the editorial team and an academic editor with relevant expertise, I'm pleased to invite you to revise the paper in response to the reviewers' comments. We plan to send the revised paper to some or all of the original reviewers, and may seek the advice of an additional subject matter expert in the new round of review. We cannot provide any guarantees at this stage regarding publication.

In addition to these revisions, you may need to complete some formatting changes, which you will receive in a follow up email. A member of our team will be in touch with a set of requests shortly. If you do not receive a separate email within a few days, please assume that checks have been completed, and no additional changes are required.

When you upload your revision, please include a point-by-point response that addresses all of the reviewer and editorial points, indicating the changes made in the manuscript and either an excerpt of the revised text or the location (eg: page and line number) where each change can be found. Please also be sure to check the general editorial comments at the end of this letter and include these in your point-by-point response. When you resubmit your paper, please include a clean version of the paper as the main article file and a version with changes tracked as a marked-up manuscript. It may also be helpful to check the guidelines for revised papers at http://journals.plos.org/plosmedicine/s/revising-your-manuscript for any that apply to your paper.

We ask that you submit your revision by Jun 29 2026 11:59PM. However, if this deadline is not feasible, please contact me by email, and we can discuss a suitable alternative.

Don't hesitate to contact me directly with any questions (lgaynor@plos.org).

Best regards,

Louise

Louise Gaynor-Brook, MBBS PhD

Senior Editor

PLOS Medicine

lgaynor@plos.org

-----------------------------------------------------------

Comments from the academic editor:

This study included patients from 2016 onwards: what was written in the ICF, for how long can their data be re-used?

-----------------------------------------------------------

Comments from the reviewers:

Reviewer #1: SUMMARY

The authors present a secondary analysis from the ADORE trial which compared early pre-term birth outcomes between participants randomised to standard (200mg) and high (1000mg) dose of DHA supplementation. This secondary analysis used genotyping data for the FADS Indel polymorphism to investigate whether dosing effects differ between genotypes. The authors report differing effects of the DHA dosing between genotypes and recommend prescribing DHA dosing level based on FADS Indel genotype.

PURPOSE OF REVIEW

This is a statistical review and focuses foremost on statistical rigour of the study, though I do add some less statistical comments.

OVERALL ASSESSMENT

This is a robust secondary analysis using a principled Bayesian analysis - at least as far is possible to judge from the described Methods. Findings are convincing, well reported and subjected to robust sensitivity analyses. My main comment is that the Bayesian models need to be described in more detail and that some model checks should be done and reported. Besides these, I have mostly minor comments, including on the absence of a publicly available SAP, some CONSORT2025 compliance issues and minor reporting issues.

MAJOR COMMENTS

M1. The binomial models should be described more clearly in Methods. While the outcome variables are clear and it is clear that the DHA dose exposure and FADS Indel genotype are included as predictor variables, it is unclear if any other variables such as center, age, sex etc were included. Further the model building process seems not to be described. E.g. p.8 refers to a final model - but no model selection procedure is described (incidentally this should probably be a plural given there was more than one model; e.g. one model for PB and one for EPB).

M2. While the authors provide extensive sensitivity analyses to appraise the robustness of the results, it is a bit surprising to find no indication of any model diagnostics having been done - e.g. given that the models were fitted by MCMC, I would expect a statement on whether trace plots, potential scale reduction factors, effective sample sizes of parameters etc were checked, whether any model fit statistics were checked, whether predictive posterior checks were done etc and maybe have some of these included as supplementary materials.

SPECIFIC COMMENTS

S1. Data availability statements in the PLOS submission form and what is written on p.17 contradict each other. I note that the data were included in the reviewer submission pack.

S2. The CONSORT 2025 checklist needs minor tweaks as some page numbers seem to be off by a count of 1. Further, not all points are fully compliant: 1) the item on sample size is not compliant as the manuscript simply refers to a non-publicly available Statistical Analysis Plan (I would suggest including full details in the manuscript or at least uploading the SAP to a permanent public repository and linking to it from Methods), 2) there is no description of blinding procedures, 3) missing data handling (which is straightforward in a Bayesian model as long as all variables that include missing data have distributions specified but that is not clear; note I do not refer to the sensitivity analysis for participants not wishing to be genotyped - that is very good) and 4) item 26 presentation of binary results (see also comment S4 below).

S3. Methods should also be more specific on reporting - e.g. mention that CONSORT2025 are followed (as they are) and explain what descriptive and analysis summaries are being reported and what other analyses were done for more simple comparisons. E.g. this would make it clear that "CI" refers generally to Bayesian confidence intervals (which some readers may know as credible intervals). Also on p.12, there seems to be a frequentist analysis that uses the same CI abbreviation for a frequentist confidence interval (for the difference in cervical length), together with a frequentist p-value for what is probably a t-test or Wilcoxon rank-sum test but this is not clear. Or was this also a Bayesian analysis and the 'p' should be a 'PP'?

S4. I would suggests consistently reporting results in the same way on 9-12. Currently sometimes only percentages with CIs are given and sometimes n/N are given and at other times only ratios are quoted. I would suggest consistently reporting n/N, %, 95% Bayesian CI for numbers and percentages of outcomes, and ratios, 95% Bayesian CIs, Bayesian PPs for comparisons. E.g. on p.9, as an example, report a CI for the 36% figure (which for consistency could be reported as a ratio of 0.64) and CIs for the 1.7 and 5.4-fold figures.

S5. Avoid using the PP acronym for both posterior probability and post-partum as this is confusing. To be fair the latter only seems to be used on Figure 5 (though also in the data file). Probably the easiest is just to spell out the x-axis titles on Figure 5 (there is certainly enough space for 'baseline' and 'post-partum').

S6. p.9, first sentence on the last paragraph has something missing. Perhaps it should be "carriers were at 1.7-fold and 5.4-fold greater risk than D/D"?

S7. Add vertical lines to Figures 2, 3, 4, S5 and S6 to indicate the EPB (34 weeks) and PB (37 weeks) cut-offs as this will make it easier to read the Kaplan-Meier graphs. I also do not think that Figures S5a and S6a are the best visual summaries for comparing prolonged gestation given that the curves shown are cumulative. The numbers given below each graph help with this, but the graphs themselves are not clear for comparing prolonged deliveries.

S8. Make some statements less definite as it implies a similar fallacy to interpreting results as taking a binary cut-off for p<0.05 in frequentist analyses. E.g. (all are just suggestions) p.10 "no effect was observed" --> "no meaningful effect was observed", p.11 "PB and EPB were indistinguishable" --> "PB and EPB were statistically indistinguishable" etc. If a hard interpretation of PPs is chosen, then the authors should make it clear what threshold of PP they use to distinguish between effects and non-effects - but personally I do not think that would be within the spirit of a nuanced Bayesian analysis.

S9. The sentence "In other words, DHA dose did not affect the rate of ARA synthesis in the group overall" on p.11 is not clear unless the sentence before this is broken apart by DHA dose. Currently I do not see how a statement about D/D vs I/I genotype can lead to a conclusion about DHA dose.

S10. Having between a third and half of eligibility exclusions listed as 'other reasons' is not great on the CONSORT diagram. Can this be broken down a bit more?

S11. It would be good to have early on a single table listing simply PB and EPB events overall, by arm and by genotype.

S12. Remove the word 'potential' from the first line of the last paragraph before the Conclusion on p.16.

S13. Add the word 'secondary' in from of 'analysis' in the first sentence of the Conclusion on p.16.

S14. I am not sure the list of abbreviations is helpful if it is not complete - e.g. PP, CI etc are not featured.

S15. Table 1 is not cross-referenced in the text.

TYPOS

T1. Some reference numbers are wrong. E.g. on p.8 reference 6 is for a berry et al. publication, but reference 6 does not have a Berry as the lead author. This reference seems to be number 26 instead? Likewise supplementary tables 1-4 captions refers to presumably the same Berry publication as number 23 - again, most likely number 26.

T2. p.4, l.9: reflecting a allele-dose --> reflecting an allele-dose

T3. p.9, 2nd paragraph in results, the 74% EPB figure is 76% when manually computed but this is likely just due to errors introduced when trying to reproduce this from rounded figures.

T4. p.10, 4th paragraph, PP=0.07 -- this must be incorrect; should this be 0.97?

T5. Figure 1: 'nineth' --> 'ninth' (this needs correcting twice on the Figure)

Reviewer #2: The authors present an interesting secondary analysis following up on an evolving story in the literature of an indel under strong positive selection that influences the efficiency of PUFA elongation. While adaptive in the past, this indel may be maladaptive in a modern environment where dietary precursor PUFAs are abundant. The authors examine whether this indel interacts with high vs low dose DHA supplementation in a secondary analysis of the ADORE trial, a landmark study examining dose-x-status interactions in impacting birth outcomes. The authors employ a rigorous Bayesian approach to a mechanistically guided hypothesis that is highly relevant at a time when there is enthusiasm for 'Precision Nutrition' and the evidence around N3 supplementation for prevention adverse birth outcomes is growing. The new analysis is an important story for the literature, and is likely to spur additional investigations and be highly cited. However, there are modifications necessary for this to be published in PLOS Medicine.

My comments are mostly minor and focus on interpretation and reproducibility/transparency; this is otherwise a strong manuscript. Comments:

- The post-hoc nature of the analysis is generally underplayed in the presentation and interpretation of the data. As this was not preplanned, the title should transparently refer to the investigation as a post-hoc over a 'secondary' analysis. Causal sounding language is used throughout (e.g. Abstract: "High-dose DHA (1000 mg/d) reduces preterm birth rate for I allele carriers but increased later term and post-term birth in DD. Matching dose to genotype yields the highest percentage of births 37-40 weeks" - should be "is associated with a reduced risk of preterm birth for I allele carriers".

-Going along with the post-hoc issue, the analysis is not adequately powered to assess the full spectrum of possible genotypes with interactions and collapses heterozygotes and homozygotes for the indel as carriers. While this is common practice in the nutrigenetic literature, it is potentially obscures meaningful biological differences and serves mostly a statistical purpose to increase power. This should be more transparently handled in the analysis and enthusiasm tempered appropriately in the interpretation. For example, it is seen in Supplemental Figure 1 and 2 that there is clearer separation in the posterior distributions between the I/D 1000mg vs 200mg groups for PB and EPB outcomes than for the I/I genotype (PP=0.98&0.98, strong for I/D; PP=0.47 & 0.86, very weak to moderate for D/D). Whether there is a true biological disadvantage to carrying a single allele of the indel vs 2 copies is interesting to consider, but inseparable in this analysis from statistical underpowering and reality of using a non-hierachical bayesian modeling approach that treats each group entirely independently when the I/I genotype is relatively small in number . The manuscript as written is presenting a bit more of a cleaner story than the data fully support.

- The findings for high dose DHA in carriers are much more robust than for EPB and PB - the authors should explain this more clearly in their interpretation. They have correctly shown the fragility of the PB PP in Supp Table 10 with small changes to the gamma resulting in substantially reduced PP's but underinterpret it.

-More detail about the 'weakly informative prior' is needed to better understand the results and facilitate reproducibility; what is the distributional family, what were the parameters; what is the scale; is the prior the same across all models; how prior sensitivity was addressed; Show plots for the prior and posterior distributions, particularly for the lower power I/I subgroups.

-It would be useful to show indel effects on a more complete spectrum of the fatty acid profile in the supplement, beyond the ARA/DGLA data already presented - this data would be useful for the field and provide information in the context of pregnancy. Previous reports from this group have highlighted impacts on LA, DGLA, ARA and DTA, as well as ALA, EPA, DHA. Previous reports on the ADORE trial have identified dose-x-status relationships to birth outcomes, and while this analysis is not powered to assess dose-x-status-x-genotype relationships fully, it would be useful for the field to see how these genotypes might relate to n3 status indicators and past trial reports.

-The handling of race, ancestry and their interactions in the manuscript should be reviewed for consistency and clarity (few readers understand the difference). The language could be more careful to clearly define these, their potential overlap and differences, and clear attribution given to potential causal effects. Of note in the interpretation of this data, Supp Table 5 argues for a stronger effect of high dose DHA supplementation in indel carriers who are black than 'other' racial identities. Whether this is due to other ancestral genetics collinear with a black race or due to other environmental and life course factors unique to black individuals remains unclear but cuts against the language used by teh authors that the indel is 'driving' ancestral differences in length somewhat. This could be untangled a bit more in the Discussion.

Minor:

-"Circulating ARA increases in response to intake of its omega-6 fatty acid precursor, linoleic acid, up to about 4% of total energy intake. " - needs a citation

Reviewer #3: This manuscript provides a genotype-stratified secondary analysis of the ADORE randomized DHA supplementation trial. The authors examined the key question whether the FADS Indel polymorphism (rs66698963)) is associated with varying effects of DHA dose on PB and EPB during pregnancy. Overall, the manuscript is well written, the statistical methods were appropriately conducted. The findings may help explain heterogeneity in response to DHA supplementation during pregnancy and could be relevant to precision medicine approaches for reducing PB and EPB. However, there are major concerns regarding the interpretation of genotype-specific effects. Major revision is required before the findings can be interpreted with confidence.

Major comments:

1. The sample size for the PB and especially EPB analyses is limited. Although 761 participants were included, there were only 17 EPB cases and 74 PB cases, if I counted correctly from the reported results. After the stratification by FADS genotypes and DHA dose, several groups contain very few cases. Therefore, the authors should interpret the findings more cautiously and add a discussion of this limitation.

2. Baseline characteristics should be compared across FADS genotype groups. This could either be added to the current Table 1 or provided as a separate table.

3. Since FADS genotype was not measured or used for randomization before the study, potential confounding across genotype groups needs to be assessed. Possible confounders should be considered in genotype analysis models.

4. Based on the authors' previous study, it seems that baseline DHA levels were also significantly associated with EPB and PB rates. Did the authors investigate the associations between FADS genotype and baseline DHA, ARA/DGLA levels? It is also not clear whether baseline DHA and ARA-related measures were included in the genotype analysis models. This should be clarified.

5. I would suggest toning down wording such as "genotype-directed" DHA dosing. The study supports the possibility that FADS genotype may be involved in the process in response to DHA supplementation, but the genotype-guided dosing strategy was not prospectively or functionally tested. The authors should also add a limitation stating that larger cohorts and prospective validation are needed before FADS genotype can be used as a functional biomarker to guide DHA dosing in pregnancy.

Minor comments:

1. As the methods indicate that race and ethnicity were self-reported, it would be better to avoid using the term "ancestry".

2. The data availability statement is inconsistent. In the submission checklist, the authors state that all data are fully available without restriction. However in the manuscript it says "We are willing to share deidentified data from the study, including data from individual participants with a signed data access agreement that includes the study principal investigators and is contingent on their approval of the planned use of the data."

Any attachments provided with reviews can be seen via the following link: [LINK]

------------------------------------------------------------

General editorial requests:

(Note: not all will apply to your paper, but please check each item carefully)

*We ask every co-author listed on the manuscript to fill in a contributing author statement, making sure to declare all competing interests. If any of the co-authors have not filled in the statement, we will remind them to do so when the paper is revised. If all statements are not completed in a timely fashion this could hold up the re-review process. If new competing interests are declared later in the revision process, this may also hold up the submission. Should there be a problem getting one of your co-authors to fill in a statement we will be in contact. Please do not add or remove authors without first discussing this with the handling editor. You can see our competing interests policy here: http://journals.plos.org/plosmedicine/s/competing-interests.

*Please upload any figures associated with your paper as individual TIF or EPS files with 300dpi resolution at resubmission; please read our figure guidelines for more information on our requirements: http://journals.plos.org/plosmedicine/s/figures. While revising your submission, we strongly recommend that you use PLOS's NAAS tool (https://ngplosjournals.pagemajik.ai/artanalysis) to test your figure files. NAAS can convert your figure files to the TIFF file type and meet basic requirements (such as print size, resolution), or provide you with a report on issues that do not meet our requirements and that NAAS cannot fix.

After uploading your figures to PLOS's NAAS tool - https://ngplosjournals.pagemajik.ai/artanalysis, NAAS will process the files provided and display the results in the "Uploaded Files" section of the page as the processing is complete.

If the uploaded figures meet our requirements (or NAAS is able to fix the files to meet our requirements), the figure will be marked as "fixed" above. If NAAS is unable to fix the files, a red "failed" label will appear above.

When NAAS has confirmed that the figure files meet our requirements, please download the file via the download option, and include these NAAS processed figure files when submitting your revised manuscript.

*Please ensure that the paper adheres to the PLOS Data Availability Policy (see http://journals.plos.org/plosmedicine/s/data-availability), which requires that all data underlying the study's findings be provided in a repository or as Supporting Information. For data residing with a third party, authors are required to provide instructions with contact information (web or email address) for obtaining the data. Please note that a study author cannot be the contact person for the data. PLOS journals do not allow statements supported by "data not shown" or "unpublished results." For such statements, authors must provide supporting data or cite public sources that include it.

*We expect all researchers with submissions to PLOS in which author-generated code underpins the findings in the manuscript to make all author-generated code available without restrictions upon publication of the work. In cases where code is central to the manuscript, we require the code to be made available as a condition of publication. Authors are responsible for ensuring that the code is reusable and well documented. Please make any custom code available, either as part of your data deposition or as a supplementary file. Please add a sentence to your data availability statement regarding any code used in the study, e.g. "The code used in the analysis is available from Github [URL] and archived in Zenodo [DOI link]" Please review our guidelines at https://journals.plos.org/plosmedicine/s/materials-software-and-code-sharing and ensure that your code is shared in a way that follows best practice and facilitates reproducibility and reuse. Because Github depositions can be readily changed or deleted, we encourage you to make a permanent DOI'd copy (e.g. in Zenodo) and provide the URL.

*Data Availability statement: A study author cannot be the contact person. If the data are not freely available, please describe briefly the ethical, legal, or contractual restriction that prevents you from sharing it. Please also include an appropriate contact (web or email address) for inquiries.

*Please include your Statistical Analysis Plan in the supplementary files.

*Please ensure that the study is reported according to the CONSORT guideline and include the completed checklist as Supporting Information. When completing the checklist, please use section and paragraph numbers, rather than page numbers. Please add the following statement, or similar, to the Methods: "This study is reported as per the CONsolidated Standards of Reporting Trials (CONSORT) guideline (S1 Checklist)."

FORMATTING - GENERAL

*Abstract: Please structure your abstract using the PLOS Medicine headings (Background, Methods and Findings, Conclusions). Please combine the Methods and Findings sections into one section.

*At this stage, we ask that you include a short, non-technical Author Summary of your research to make findings accessible to a wide audience that includes both scientists and non-scientists. The Author Summary should immediately follow the Abstract in your revised manuscript. This text is subject to editorial change and should be distinct from the scientific abstract. Ideally each sub-heading should contain 2-3 single sentence, concise bullet points containing the most salient points from your study. In the final bullet point of 'What Do These Findings Mean?', please include the main limitations of the study in non-technical language. Please see our author guidelines for more information: https://journals.plos.org/plosmedicine/s/revising-your-manuscript#loc-author-summary.

*Please express the main results with 95% CIs as well as p values. When reporting p values please report as p<0.001 and where higher as the exact p value p=0.002, for example. Throughout, suggest reporting statistical information as follows to improve clarity for the reader "22% (95% CI [13%,28%]; p</=)". Please be sure to define all numerical values at first use.

*Please include page numbers and line numbers in the manuscript file. Use continuous line numbers (do not restart the numbering on each page).

*Please cite the reference numbers in square brackets. Citations should precede punctuation.

FIGURES AND TABLES

*Please provide titles and legends for all figures and tables (including those in Supporting Information files).

*Please define all abbreviations used in each figure/table (including those in Supporting Information files).

*Please consider avoiding the use of red and green in order to make your figure more accessible to those with color blindness.

SUPPLEMENTARY MATERIAL

*Please note that supplementary material will be posted as supplied by the authors. Therefore, please amend it according to the relevant comments outlined here.

*Please cite your Supporting Information as outlined here: https://journals.plos.org/plosmedicine/s/supporting-information

REFERENCES

*PLOS uses the numbered citation (citation-sequence) method and first six authors, et al.

*Please ensure that journal name abbreviations match those found in the National Center for Biotechnology Information (NCBI) databases (http://www.ncbi.nlm.nih.gov/nlmcatalog/journals), and are appropriately formatted and capitalised.

*Where website addresses are cited, please include the complete URL and specify the date of access (e.g. [accessed: 12/06/2023]).

*Please also see https://journals.plos.org/plosmedicine/s/submission-guidelines#loc-references for further details on reference formatting.

STUDY TYPE-SPECIFIC REQUESTS

*If the analyses were not prespecified in the RCT protocol, please define them in the Methods as post hoc. Post-hoc comparisons should be presented as hypothesis generating rather than conclusive.

*In keeping with our commitment to Open Science, please include the study protocol document and analysis plan (including any amendments) as Supporting Information to be published with the manuscript if accepted.

*Abstract: Please include the study design, population and setting, number of participants, years during which the study took place (enrollment and follow up), length of follow up, and main outcome measures.

*In the manuscript text, please indicate: (1) the specific hypotheses you intended to test, (2) the analytical methods by which you planned to test them, (3) the analyses you actually performed, and (4) when reported analyses differ from those that were planned, transparent explanations for differences that affect the reliability of the study's results. If a reported analysis was performed based on an interesting but unanticipated pattern in the data, please be clear that the analysis was data driven.

*Please state in the Methods section whether the study had a prospective protocol or analysis plan. If a prospective analysis plan (from your funding proposal, IRB or other ethics committee submission, study protocol, or other planning document written before analyzing the data) was used in designing the study, please include the relevant document(s) with your revised manuscript as a Supporting Information file to be published alongside your study and cite it in the Methods section. A legend for this file should be included at the end of your manuscript. If no such document exists, please make sure that the Methods section transparently describes when analyses were planned, and when/why any data-driven changes to analyses took place. Changes in the analysis, including those made in response to peer review comments, should be identified as such in the Methods section of the paper, with rationale.

# Author comment: Author response to Decision Letter 2

**DOI:** 10.1371/journal.pmed.1005274.r003

7 Jul 2026

Attachment Submitted filename: Brenna-Carlson Response to Reviewers v5.docx

# Peer review: Decision Letter 2

**DOI:** 10.1371/journal.pmed.1005274.r004

**Authors:** Alison Farrell

6 Aug 2026

Dear Dr Brenna,

Many thanks for submitting your manuscript "Genotype-informed docosahexaenoic acid (DHA) dosing based on the FADS Indel reduces preterm and post-40-week birth: A secondary analysis of the ADORE randomized trial" (PMEDICINE-D-26-00537R2) to PLOS Medicine. The paper has been reviewed by subject experts and a statistician; their comments are included below and can also be accessed here: [LINK]

As you will see, the statistical reviewer continues to raise important points that must be addressed in a revised version. After discussing the paper with the editorial team and an academic editor with relevant expertise, I'm pleased to invite you to revise the paper in response to the reviewers' comments. We plan to send the revised paper to some or all of the original reviewers, and we cannot provide any guarantees at this stage regarding publication.

The following issues should also be addressed:

Please remove all causal language in the manuscript, including the title, Abstract and main text.

Please remove reference to race alone as it is a social construct. Use of 'race/ethnicity' could be used. In the Abstract, please state that Black women had higher rates of PB.

Please use patient-centered language throughout.

Please temper claims in the Abstract related to preterm birth and ancestry, as not all forms of preterm birth have been shown to be linked to ancestry.

In the first bullet point of the Author Summary, line 58, please state if this comment related to global evidence or US-based evidence.

In the 3rd bullet point of the Author Summary, please revise the statement (e.g. replace predicts with associates), given that the data presented do not formally predict who would benefit from high dose DHA, which the framing implies.

Please use associative language in the Author Summary.

Temper line 78.

The Discussion is >2500 words. Please reduce length, removing any repetition and temper claims, given that the study is a post hoc analysis.

Please include a discussion of study limitations.

Please confirm that the original consent documentation permitted extended use of the data or samples collected in the trial.

In addition to these revisions, you may need to complete some formatting changes, which you will find at the end of this letter, and additional requests that you will receive in a follow up email. A member of our team will be in touch with a set of requests shortly. If you do not receive a separate email within a few days, please assume that checks have been completed, and no additional changes are required.

Please use patient-centric language.

When you upload your revision, please include a point-by-point response that addresses all of the reviewer and editorial points, indicating the changes made in the manuscript and either an excerpt of the revised text or the location (eg: page and line number) where each change can be found. Please also be sure to check the general editorial comments at the end of this letter and include these in your point-by-point response. When you resubmit your paper, please include a clean version of the paper as the main article file and a version with changes tracked as a marked-up manuscript. It may also be helpful to check the guidelines for revised papers at http://journals.plos.org/plosmedicine/s/revising-your-manuscript for any that apply to your paper.

We ask that you submit your revision by Aug 27 2026 11:59PM. However, if this deadline is not feasible, please contact me by email, and we can discuss a suitable alternative.

Don't hesitate to contact me directly with any questions (afarrell@plos.org).

Best regards,

Alison

Alison Farrell, Ph.D.

Senior Editor

PLOS Medicine

afarrell@plos.org

-----------------------------------------------------------

Comments from the editors:

GENERAL EDITORIAL REQUESTS

"* At this stage, we ask that you include a short, non-technical Author Summary of your research to make findings accessible to a wide audience that includes both scientists and non-scientists. The Author Summary should immediately follow the Abstract in your revised manuscript. This text is subject to editorial change and should be distinct from the scientific abstract. Ideally each sub-heading should contain 2-3 single sentence, concise bullet points containing the most salient points from your study. In the final bullet point of ‘What Do These Findings Mean?’ Please include the main limitations of the study in non-technical language.

Please see our author guidelines for more information: https://journals.plos.org/plosmedicine/s/revising-your-manuscript#loc-author-summary."

* Please confirm that your title complies with PLOS Medicine's style. Your title must be nondeclarative and not a question. It should begin with main concept if possible. "Effect of" should be used only if causality can be inferred, i.e., for an RCT. Please place the study design ("A randomized controlled trial," "A retrospective study," "A modelling study," etc.) in the subtitle (ie, after a colon).

* Please confirm that your abstract complies with our requirements, including format (three sections: Background, Methods and Findings, and Conclusions) and providing all the information relevant to this study type https://journals.plos.org/plosmedicine/s/submission-guidelines#loc-abstract

* Please ensure that the Introduction ends with a clear description of the study question or hypothesis.

* Please ensure that all abbreviations are defined at first use throughout the text.

* Please confirm that all numbers presented in the abstract are present and identical to numbers presented in the main manuscript text.

GENERAL

* Please include a statement regarding use or non-use of AI tools and technologies at the end of the Methods. Please specify the name(s) and version(s) of any tool(s) used and a description of their use, including how the AI-assisted or generated content was evaluated by authors, and a clear statement of which aspects of the study, article contents, data, or supporting files were affected or generated by AI tool usage. Please also include a statement specifying who retains responsibility for the final manuscript. See our guidelines for more information: https://journals.plos.org/plosmedicine/s/ethical-publishing-practice#loc-artificial-intelligence-tools-and-technologies.

* Please review your text for claims of novelty or primacy (e.g. 'for the first time') and remove this language. In addition, please check that any use of statistical terms (such as trend or significant) are supported by the data, and if not please remove them.

* Please remove the 'conclusions' subheading from the discussion. Please also remove any other subheadings from the discussion.

"* Statistical reporting: Please revise throughout the manuscript, including tables and figures.

- Please report statistical information as follows to improve clarity for the reader ""22% (95% CI [13,28]; p</=)"".

- Please separate upper and lower bounds with commas instead of hyphens as the latter can be confused with reporting of negative values.

- Please repeat statistical definitions (HR, CI etc.) for each set of parentheses."

* In the last sentence of the Abstract Methods and Findings section, please describe the main limitation(s) of the study's methodology.

* In the abstract, please include the important dependent variables that are adjusted for in the analyses.

* In the author summary, please revise formatting and ensure you use bullet points.

* In the author summary, in the final bullet point of 'What Do These Findings Mean?', please include the main limitations of the study in non-technical language.

FUNDING STATEMENT

* The funding statement should include: specific grant numbers, initials of authors who received each award, URLs to sponsors’ websites. Also, please state whether any sponsors or funders (other than the named authors) played any role in study design, data collection and analysis, the decision to publish, or preparation of the manuscript. If they had no role in the research, include this sentence: “The funders had no role in study design, data collection and analysis, decision to publish, or preparation of the manuscript.”

COMPETING INTERESTS STATEMENT

* All authors must declare their relevant competing interests per the PLOS policy, which can be seen here: https://journals.plos.org/plosmedicine/s/competing-interests For authors with ties to industry, please indicate whether any of the interests has a financial stake in the results of the current study.

DATA AVAILABILITY

"* The Data Availability Statement (DAS) requires revision. For each data source used in your study:

a) If the data are freely or publicly available, note this and state the location of the data: within the paper, in Supporting Information files, or in a public repository (include the DOI or accession number).

b) If the data are owned by a third party but freely available upon request, please note this and state the institutional owner of the data set and contact information for data requests (institutional web or email address). Note that a study author cannot be the contact for the data.

c) If the data are not freely available, please describe briefly the ethical, legal, or contractual restriction that prevents you from sharing it. Please also include an appropriate institutional contact (institutional web or email address) for inquiries (again, this cannot be a study author).

FIGURES

* Please provide titles and legends for all figures and tables (including those in Supporting Information files). Please define all acronyms used in each figure or table in its corresponding legend.

* In Kaplan-Meier curve(s) please provide the number at risk for each time interval.

* Please provide the unadjusted comparisons as well as the adjusted comparisons in all relevant Tables

* Please specify the variables controlled for in all relevant Tables

ACKNOWLEDGMENTS

* Please include an acknowledgment of study participants.

OBSERVATIONAL, COHORT, CROSS-SECTIONAL, AND CASE CONTROL STUDIES

"* Please ensure that the study is reported according to the STROBE guideline, and include the completed STROBE checklist as Supporting Information. Please add the following statement, or similar, to the Methods: ""This study is reported as per the Strengthening the Reporting of Observational Studies in Epidemiology (STROBE) guideline (S1 Checklist).""

The STROBE guideline can be found here: http://www.equator-network.org/reporting-guidelines/strobe/

When completing the checklist, please use section and paragraph numbers, rather than page numbers."

"* Did your study have a prospective protocol or analysis plan? Please state this (either way) early in the Methods section.

a) If a prospective analysis plan (from your funding proposal, IRB or other ethics committee submission, study protocol, or other planning document written before analyzing the data) was used in designing the study, please include the relevant prospectively written document with your revised manuscript as a Supporting Information file to be published alongside your study, and cite it in the Methods section. A legend for this file should be included at the end of your manuscript.

b) If no such document exists, please make sure that the Methods section transparently describes when analyses were planned, and when/why any data-driven changes to analyses took place.

c) In either case, changes in the analysis-- including those made in response to peer review comments-- should be identified as such in the Methods section of the paper, with rationale."

* Your study is observational and therefore causality cannot be inferred. Please remove language that implies causality and refer to associations instead.

* For all observational studies, in the manuscript text, please indicate: (1) the specific hypotheses you intended to test, (2) the analytical methods by which you planned to test them, (3) the analyses you actually performed, and (4) when reported analyses differ from those that were planned, transparent explanations for differences that affect the reliability of the study's results. If a reported analysis was performed based on an interesting but unanticipated pattern in the data, please be clear that the analysis was data-driven.

-----------------------------------------------------------

Comments from the reviewers:

Reviewer #1: The authors very thoughtfully addressed most of my comments from the first round — many thanks. The current version reads very well, and the reported model diagnostics are exemplary now. The individual stratum-level estimates are well characterised and the sensitivity analyses are thorough. There is one comment that has not fully been addressed and several new ones that came up while I re-read the manuscript.

COMMENT FROM PREVIOUS ROUND REMAINING UNADDRESSED

1. My previous comment S10 (large number of excluded participants listed only as 'other reasons') is claimed to be addressed in the authors' response letter, but Figure 1 still lists the exact same number for 'other reasons' (3,966/10,497). This is 38% of those screened, which is relevant to the generalisability argument made for supplementation in an unselected population.

NEW COMMENTS

2. My main new comment is something I did not fully appreciate on my first reading. The headline finding — and the claim in the title — is an interaction: that the effect of DHA dose differs by FADS Indel genotype. However, this is reported only as two separate genotype-stratified analyses, with a stronger dose effect in I-allele carriers than in D/D. The interaction itself is never estimated. While (frequentist) null hypothesis significance testing is not what the authors did here, this is nevertheless an instance of the "difference in significance is not (necessarily) a significant difference" fallacy. The authors' response to Reviewer 2's second comment touches on this tangentially, but the paper would be strengthened if the authors explained why no genotype × dose interaction was interrogated directly, and reported a posterior probability for the interaction itself. As I understand the parameterisation described in the response to my M1 comment from the previous round — independent arm-level log-odds with no pooling across arms — this should require no new model fit. The posterior for the interaction is a contrast of draws already available: the PP that (θ_I-carrier,200 − θ_I-carrier,1000) > (θ_D/D,200 − θ_D/D,1000). This should be done for both PB and EPB outcomes, and for the post-40-week outcome if that claim is retained in the title (see comment #5 below). I appreciate that the sample size may well leave the interaction inconclusive, particularly for PB where the two genotype-specific estimates point in the same direction. An inconclusive interaction is an acceptable result and would not undermine the paper, but it would need to be reflected in the title, abstract, and Conclusions, which currently assert a genotype-dependent dose effect as established.

3. More minor, but not clear to me why IPW was restricted to the 200 mg dose group — as Fig 1 shows, non-consent to genetic research occurred in both groups.

4. l.184: not fully clear to me where the 828 comes from when looking at Figure 1; should this be 840? From the figure, 1,032 − (103 + 89) = 840 consenting to genetic research, and 840 − (41 + 38) = 761 with complete data, which matches the analysed sample exactly.

5. The post-40-week outcome appears in the title but is not listed among the primary or secondary outcomes in the Outcomes section, and it is described variously as 'prolonged gestation', 'late-term' and 'post-term'. Might be best to remove it from the title if it is not a primary outcome and use consistent terminology for it throughout the manuscript.

Reviewer #2: The authors have responded well to the reviewer comments and the manuscript is a strong addition to the literature.

Reviewer #3: The authors have addressed my concerns and I agree the manuscript to be published in its current form.

Any attachments provided with reviews can be seen via the following link: [LINK]

--------------------------------------------------------- ---

General editorial requests:

(Note: not all will apply to your paper, but please check each item carefully)

*We ask every co-author listed on the manuscript to fill in a contributing author statement, making sure to declare all competing interests. If any of the co-authors have not filled in the statement, we will remind them to do so when the paper is revised. If all statements are not completed in a timely fashion this could hold up the re-review process. If new competing interests are declared later in the revision process, this may also hold up the submission. Should there be a problem getting one of your co-authors to fill in a statement we will be in contact. Please do not add or remove authors without first discussing this with the handling editor. You can see our competing interests policy here: http://journals.plos.org/plosmedicine/s/competing-interests.

*Please upload any figures associated with your paper as individual TIF or EPS files with 300dpi resolution at resubmission; please read our figure guidelines for more information on our requirements: http://journals.plos.org/plosmedicine/s/figures. While revising your submission, we strongly recommend that you use PLOS's NAAS tool (https://ngplosjournals.pagemajik.ai/artanalysis) to test your figure files. NAAS can convert your figure files to the TIFF file type and meet basic requirements (such as print size, resolution), or provide you with a report on issues that do not meet our requirements and that NAAS cannot fix.

After uploading your figures to PLOS's NAAS tool - https://ngplosjournals.pagemajik.ai/artanalysis, NAAS will process the files provided and display the results in the "Uploaded Files" section of the page as the processing is complete.

If the uploaded figures meet our requirements (or NAAS is able to fix the files to meet our requirements), the figure will be marked as "fixed" above. If NAAS is unable to fix the files, a red "failed" label will appear above.

When NAAS has confirmed that the figure files meet our requirements, please download the file via the download option, and include these NAAS processed figure files when submitting your revised manuscript.

*Please ensure that the paper adheres to the PLOS Data Availability Policy (see http://journals.plos.org/plosmedicine/s/data-availability), which requires that all data underlying the study's findings be provided in a repository or as Supporting Information. For data residing with a third party, authors are required to provide instructions with contact information (web or email address) for obtaining the data. Please note that a study author cannot be the contact person for the data. PLOS journals do not allow statements supported by "data not shown" or "unpublished results." For such statements, authors must provide supporting data or cite public sources that include it.

*We expect all researchers with submissions to PLOS in which author-generated code underpins the findings in the manuscript to make all author-generated code available without restrictions upon publication of the work. In cases where code is central to the manuscript, we require the code to be made available as a condition of publication. Authors are responsible for ensuring that the code is reusable and well documented. Please make any custom code available, either as part of your data deposition or as a supplementary file. Please add a sentence to your data availability statement regarding any code used in the study, e.g. "The code used in the analysis is available from Github [URL] and archived in Zenodo [DOI link]" Please review our guidelines at https://journals.plos.org/plosmedicine/s/materials-software-and-code-sharing and ensure that your code is shared in a way that follows best practice and facilitates reproducibility and reuse. Because Github depositions can be readily changed or deleted, we encourage you to make a permanent DOI'd copy (e.g. in Zenodo) and provide the URL.

FORMATTING - GENERAL

*Abstract: Please structure your abstract using the PLOS Medicine headings (Background, Methods and Findings, Conclusions). Please combine the Methods and Findings sections into one section.

*At this stage, we ask that you include a short, non-technical Author Summary of your research to make findings accessible to a wide audience that includes both scientists and non-scientists. The Author Summary should immediately follow the Abstract in your revised manuscript. This text is subject to editorial change and should be distinct from the scientific abstract. Ideally each sub-heading should contain 2-3 single sentence, concise bullet points containing the most salient points from your study. In the final bullet point of 'What Do These Findings Mean?', please include the main limitations of the study in non-technical language. Please see our author guidelines for more information: https://journals.plos.org/plosmedicine/s/revising-your-manuscript#loc-author-summary.

*Please express the main results with 95% CIs as well as p values. When reporting p values please report as p<0.001 and where higher as the exact p value p=0.002, for example. Throughout, suggest reporting statistical information as follows to improve clarity for the reader "22% (95% CI [13%,28%]; p</=)". Please be sure to define all numerical values at first use.

*Please include page numbers and line numbers in the manuscript file. Use continuous line numbers (do not restart the numbering on each page).

*Please cite the reference numbers in square brackets. Citations should precede punctuation.

FIGURES AND TABLES

*Please provide titles and legends for all figures and tables (including those in Supporting Information files).

*Please define all abbreviations used in each figure/table (including those in Supporting Information files).

*Please consider avoiding the use of red and green in order to make your figure more accessible to those with color blindness.

SUPPLEMENTARY MATERIAL

*Please note that supplementary material will be posted as supplied by the authors. Therefore, please amend it according to the relevant comments outlined here.

*Please cite your Supporting Information as outlined here: https://journals.plos.org/plosmedicine/s/supporting-information

REFERENCES

*PLOS uses the numbered citation (citation-sequence) method and first six authors, et al.

*Please ensure that journal name abbreviations match those found in the National Center for Biotechnology Information (NCBI) databases (http://www.ncbi.nlm.nih.gov/nlmcatalog/journals), and are appropriately formatted and capitalised.

*Where website addresses are cited, please include the complete URL and specify the date of access (e.g. [accessed: 12/06/2023]).

*Please also see https://journals.plos.org/plosmedicine/s/submission-guidelines#loc-references for further details on reference formatting.

# Author comment: Author response to Decision Letter 3

**DOI:** 10.1371/journal.pmed.1005274.r005

24 Aug 2026

Attachment Submitted filename: Point by point response to reviewers R2 v5.docx

# Peer review: Decision Letter 3

**DOI:** 10.1371/journal.pmed.1005274.r006

**Authors:** Alison Farrell

21 Sep 2026

Dear Dr. Brenna,

Thank you very much for re-submitting your manuscript "Genotype-informed docosahexaenoic acid (DHA) dosing based on the FADS Indel to reduce preterm and prolonged gestation: A secondary analysis of the ADORE randomized trial" (PMEDICINE-D-26-00537R3) for review by PLOS Medicine.

I have discussed the paper with my colleagues and the academic editor and it was also seen again by one reviewer. The reviewer has some remaining minor revisions, detailed at the end of this email, that must be included in a revised manuscript. I am pleased to say that provided the remaining editorial and production issues are dealt with we are planning to accept the paper for publication in the journal.

The remaining issues that need to be addressed are listed at the end of this email. Any accompanying reviewer attachments can be seen via the link below. Please take these into account before resubmitting your manuscript:

[LINK]

***Please note while forming your response, if your article is accepted, you may have the opportunity to make the peer review history publicly available. The record will include editor decision letters (with reviews) and your responses to reviewer comments. If eligible, we will contact you to opt in or out.***

In revising the manuscript for further consideration here, please ensure you address the specific points made by each reviewer and the editors. In your rebuttal letter you should indicate your response to the reviewers' and editors' comments and the changes you have made in the manuscript. Please submit a clean version of the paper as the main article file. A version with changes marked must also be uploaded as a marked up manuscript file.

Please also check the guidelines for revised papers at http://journals.plos.org/plosmedicine/s/revising-your-manuscript for any that apply to your paper. If you haven't already, we ask that you provide a short, non-technical Author Summary of your research to make findings accessible to a wide audience that includes both scientists and non-scientists. The Author Summary should immediately follow the Abstract in your revised manuscript. This text is subject to editorial change and should be distinct from the scientific abstract.

In addition to these revisions, you may need to complete some formatting changes, which you will receive in a follow up email. A member of our team will be in touch with a set of requests shortly. If you do not receive a separate email within a few days, please assume that checks have been completed, and no additional changes are required.

We expect to receive your revised manuscript within 1 week. Please email us (plosmedicine@plos.org) if you have any questions or concerns.

We ask every co-author listed on the manuscript to fill in a contributing author statement. If any of the co-authors have not filled in the statement, we will remind them to do so when the paper is revised. If all statements are not completed in a timely fashion this could hold up the re-review process. Should there be a problem getting one of your co-authors to fill in a statement we will be in contact. YOU MUST NOT ADD OR REMOVE AUTHORS UNLESS YOU HAVE ALERTED THE EDITOR HANDLING THE MANUSCRIPT TO THE CHANGE AND THEY SPECIFICALLY HAVE AGREED TO IT.

Please ensure that the paper adheres to the PLOS Data Availability Policy (see http://journals.plos.org/plosmedicine/s/data-availability), which requires that all data underlying the study's findings be provided in a repository or as Supporting Information. For data residing with a third party, authors are required to provide instructions with contact information for obtaining the data. PLOS journals do not allow statements supported by "data not shown" or "unpublished results." For such statements, authors must provide supporting data or cite public sources that include it.

To enhance the reproducibility of your results, we recommend that you deposit your laboratory protocols in protocols.io, where a protocol can be assigned its own identifier (DOI) such that it can be cited independently in the future. Additionally, PLOS ONE offers an option to publish peer-reviewed clinical study protocols. Read more information on sharing protocols at https://plos.org/protocols?utm_medium=editorial-email&utm_source=authorletters&utm_campaign=protocols

Please review your reference list to ensure that it is complete and correct. If you have cited papers that have been retracted, please include the rationale for doing so in the manuscript text, or remove these references and replace them with relevant current references. Any changes to the reference list should be mentioned in the rebuttal letter that accompanies your revised manuscript.

Please note, when your manuscript is accepted, an uncorrected proof of your manuscript will be published online ahead of the final version, unless you've already opted out via the online submission form. If, for any reason, you do not want an earlier version of your manuscript published online or are unsure if you have already indicated as such, please let the journal staff know immediately at plosmedicine@plos.org.

If you have any questions in the meantime, please contact me or the journal staff on plosmedicine@plos.org.

We look forward to receiving the revised manuscript by Sep 28 2026 11:59PM.

Sincerely,

Alison Farrell, Ph.D.

Senior Editor

PLOS Medicine

plosmedicine.org

------------------------------------------------------------

Requests from Editors:

* In the Kaplan-Meier curve(s) please provide the number at risk for each time interval.

* Please include a statement regarding use or non-use of AI tools and technologies at the end of the Methods, under the subheading: Use of AI Disclosure. Please specify the name(s) and version(s) of any tool(s) used and a description of their use, including how the AI-assisted or generated content was evaluated by authors, and a clear statement of which aspects of the study, article contents, data, or supporting files were affected or generated by AI tool usage. Please also include a statement specifying who retains responsibility for the final manuscript. See our guidelines for more information: https://journals.plos.org/plosmedicine/s/ethical-publishing-practice#loc-artificial-intelligence-tools-and-technologies.

Please revise your Data Availability Statement in the manuscript metadata to match that in the manuscript.

Comments from Reviewers:

Reviewer #1: I thank the authors for their careful responses to my comments.

This is mostly there now. I cannot give it a full all-clear just yet, but I think the left over issues are editorial issues now (meaning the authors clarified the underlying issues in their responses but the manuscript does not fully reflect these yet) and do not need to come back to me for further review. Overall this is a very nice and robust paper and I congratulate the authors on their work.

Comment #1 - OK but perhaps add this clarification somewhere in the manuscript as well?

Comment #2 - OK, the authors have addressed this in the manuscript but not the abstract even though the response letter states exactly that - maybe just an oversight?

Comment #3 - OK, fully addressed.

Comment #4 - The response in the letter clarifies the issue satisfactorily, but I note that Figure 1 seems to be unchanged and still has the original order of the boxes with the original numbers rather than the reversed order and associated changes in numbers. Maybe just another oversight when the figure file was uploaded?

Comment 5 - OK, I am satisfied with the authors' response.

Any attachments provided with reviews can be seen via the following link:

[LINK]

# Author comment: Author response to Decision Letter 4

**DOI:** 10.1371/journal.pmed.1005274.r007

22 Sep 2026

Attachment Submitted filename: renamed_54554.pdf

# Editor report: Decision Letter 4

**DOI:** 10.1371/journal.pmed.1005274.r008

**Authors:** Alison Farrell

23 Sep 2026

Dear Dr Brenna,

On behalf of my colleagues and the Academic Editor, Isabelle Dehaene, I am pleased to inform you that we have agreed to publish your manuscript "Genotype-informed docosahexaenoic acid (DHA) dosing based on the FADS Indel to reduce preterm and prolonged gestation: A secondary analysis of the ADORE randomized trial" (PMEDICINE-D-26-00537R4) in PLOS Medicine.

Before your manuscript can be formally accepted you will need to complete some formatting changes, which you will receive in a follow up email. Please be aware that it may take several days for you to receive this email; during this time no action is required by you. Once you have received these formatting requests, please note that your manuscript will not be scheduled for publication until you have made the required changes.

We also ask you to make the following changes to your manuscript:

Please remove "(DHA)" from the title as is unnecessary.

Line 20, please spell out FADS, and clarify that it affects FADS1 expression.

Please add one last sentence to the Background of the Abstract explaining what the question is that led to this analysis.

In the first sentence of the Methods and Findings in the Abstract, please revise phrasing to clarify what the aim of this study is. In particular, please revise the phrasing of " used a prospective retrospective framework, with the FADS Indel pre-specified as a single mechanistic measurement and genotyped blind to outcome"

Please spell out PP and CrI in the Abstract at first use.

Line 38: revise to read: "High-dose DHA was associated with reduced EPB"

Lines 39 and 40: please delete the information already present in the preceding sentences. Line 39: delete "from 4.3% (9/214; 95% CrI [2.0%, 7.3%]) to " and Line 40, delete " from 14.5% (31/214; 95% CrI [10.1%, 19.5%]) to". Place "1/.1%" (line 39) and "9.3%" (line 40) within the adjacent bracket, separating it from the fraction with a semi-colon.

Line 56: Delete 'matching' as the trial did not match treatment to genotype.

Line 58: add 'potential' to read "a potential predictive biomarker"

Line 76: Please revise to: "To our knowledge, no study exists on whether this genotype associates with who benefits from high dose DHA."

Line 79: Please add "We performed a" to the start of the sentence.

Line 130: Define ARA at first use.

Please confirm that code generated for these analyses is included in the supplementary material or deposited in an open repository and include the DOI associated with the code in the Data Availability Statement.

Please revise to use associative language throughout the methods and remove causal inferences throughout (e.g. line 359).

As dosing was not matched to genotype in the trial, please avoid phrasing that suggests it was (e.g. sentence on line 417.

Line 429: please revise phrasing " Consider first mismatched dosing..".

Line 524: Delete "Supplemental."

Please remove claims of primacy (e.g. in the Discussion).

In the meantime, please log into Editorial Manager at http://www.editorialmanager.com/pmedicine/, click the "Update My Information" link at the top of the page, and update your user information to ensure an efficient production process.

PRESS

We frequently collaborate with press offices. If your institution or institutions have a press office, please notify them about your upcoming paper at this point, to enable them to help maximise its impact. If the press office is planning to promote your findings, we would be grateful if they could coordinate with medicinepress@plos.org. If you have not yet opted out of the early version process, we ask that you notify us immediately of any press plans so that we may do so on your behalf.

We also ask that you take this opportunity to read our Embargo Policy regarding the discussion, promotion and media coverage of work that is yet to be published by PLOS. As your manuscript is not yet published, it is bound by the conditions of our Embargo Policy. Please be aware that this policy is in place both to ensure that any press coverage of your article is fully substantiated and to provide a direct link between such coverage and the published work. For full details of our Embargo Policy, please visit http://www.plos.org/about/media-inquiries/embargo-policy/.

To enhance the reproducibility of your results, we recommend that you deposit your laboratory protocols in protocols.io, where a protocol can be assigned its own identifier (DOI) such that it can be cited independently in the future. Additionally, PLOS ONE offers an option to publish peer-reviewed clinical study protocols. Read more information on sharing protocols at https://plos.org/protocols?utm_medium=editorial-email&utm_source=authorletters&utm_campaign=protocols

Thank you again for submitting to PLOS Medicine. We look forward to publishing your paper.

Sincerely,

Alison Farrell, Ph.D.

Senior Editor

PLOS Medicine
